# Supplementary material for: The attitudes, perceptions and experiences of medical school applicants following the closure of schools and cancellation of public examinations in 2020 due to the COVID-19 pandemic: a cross-sectional questionnaire study of UK medical applicants
Source: BMJ Open. 2021 Mar 4;11(3):e044753. doi: 10.1136/bmjopen-2020-044753 (PMC7934197; doi:10.1136/bmjopen-2020-044753)
Supplement: Supplementary data [file bmjopen-2020-044753supp001.pdf]

FUNDED BY NIHR | National Institute for Health Research

the NHS needs YOU

UK Medical Applicant Cohort Study

UKMACS-COVID19

UKMACS COVID-19 questionnaire

Due to the coronavirus/COVID-19 outbreak, UK school examinations have been cancelled and alternatives for selecting medical students are being considered.

The UK Medical Applicant Cohort Study (UKMACS) is an academic research study about applying to medical school. We want to find out how you, the applicants, are reacting to these changes and what you think medical schools should do to select applicants fairly.

This questionnaire needs to be answered by **20th April 2020**.
The turnaround is very tight as we want the questionnaire to assist medical schools in making decisions on selection.

We keep your information private and confidential.

No medical school, school or university will be able to identify you from the information you give us.

The information you give us will NOT be used to make any decisions about you personally that will affect your education or career.

You are being invited to complete this questionnaire because you agreed to be contacted by us when you registered to take UCAT in 2019 and/or because you have already responded to one or more of our questionnaires.

Please note that we are interested in responses from ALL applicants to medical school, whether or not you completed any of our previous questionnaires, whether or not you have been offered a place, and whether or not you were due to take A levels/Highers/equivalents this year.

Before you proceed to the questionnaire, please download the study Information Sheet and read it so you understand what the study is about and what taking part entails.

Contact study lead Dr Katherine Woolf and the UKMACS research team on medsch.choice@ucl.ac.uk if you have any questions.

The study is led by Dr Katherine Woolf at University College London (UCL), and funded by the National Institute for Health Research (NIHR) Career Development Fellowship (Grant Reference Number CDF-2017-10-008). The views expressed are those of the authors and not necessarily those of the NIHR or the Department of Health and Social Care.

Eligibility to take part in this study

This study is for people aged 16 or over, resident in the UK or Crown Dependencies, who applied or were considering applying to study medicine in the UK for entry in 2020.

1. Please select "Yes" to indicate you are aged 16 or older, resident in the UK (or Channel Islands/Isle of Man). If you select "No" you cannot take part in this study. ✱ *Required*

- (•) Yes
- ( ) No

Consent to take part in this study

2. Please select “I consent” to each statement to show you understand and agree to taking part in the study. If you select "I do NOT consent" you may not be able to take part in the study.

	I consent	I do NOT consent
I understand my personal information will be kept private by the research team on password-protected computers or encrypted files at UCL, and will not be passed on to any commercial organisations. [‘Personal information’ means: name, questionnaire answers, or any other information of yours we have such as contact details, UCAT or other test registration ID number.]	◉	○
I agree my questionnaire answers can be linked with other personal information about me and used only for academic research, as explained in the Information Sheet.	◉	○
I understand that taking part in this study is voluntary, and I can withdraw by contacting the research team by the 22nd April 2020.	◉	○
I understand the potential risks of taking part in the study and the support I can get if needed, including who to contact with questions or to make a complaint (see Information Sheet).	◉	○

Woolf K, *et al. BMJ Open* 2021; 11:e044753. doi: 10.1136/bmjopen-2020-044753

How medical schools should select applicants in the absence of exam grades

Medical schools are currently discussing how to select applicants without examination grades. The following questions ask about a range of ways being discussed as possibilities. Please let us know how fair or unfair you think each option is. None of these options has yet been decided upon. Your responses may have an influence upon what medical schools decide to do.

Some of these questions are specific to applicants still at secondary school and others are more relevant to mature/graduate entrants. Please answer all questions as far as possible. Later on in the questionnaire we will ask about your educational background so we can factor that into our analyses.

3. Here is a list of different measures that medical schools could use to decide whether or not to accept **applicants who currently hold an offer**. For each method, please indicate how fair it is to use to accept or reject current offer holders now that exams have been cancelled. ["Calculated grades' are grades produced by examination boards based on estimated grades and rankings produced teachers using information on a student's progress. The method has been described by Ofqual for England and is likely to be broadly similar elsewhere].

More info

	Unfair: should not be used	Quite unfair: avoid if possible	Quite fair: could be used in combination with other measures	Very fair: could be used alone	Uncertain
Predicted grades declared on UCAS application.	○	○	○	○	○
Calculated grades based on mock exams, coursework etc, and awarded in place of final examination grades.	○	○	○	○	○
Exam grades taken in September 2020 (if these take place).	○	○	○	○	○
AS level/Higher grades taken in Year 12 (S5).	○	○	○	○	○
Grades in end-of-Year 12 (S5) internal school exams.	○	○	○	○	○
GCSE/Nat5 grades.	○	○	○	○	○
Grades in other qualifications (e.g. Extended Project Qualification).	○	○	○	○	○
For those in their final year at university, marks earlier in their course.	○	○	○	○	○
Score at interview (including MMI, panel, and other types of interview).	○	○	○	○	○
UCAT score.	○	○	○	○	○
BMAT score.	○	○	○	○	○
GAMSAT score (for Graduate Entry students).	○	○	○	○	○
UCAS personal statement.	○	○	○	○	○
UCAS reference from teacher.	○	○	○	○	○
Work experience declared on UCAS form.	○	○	○	○	○
Attendance at university summer school/widening access programme.	○	○	○	○	○
Personal background (e.g. giving an advantage to students from under-represented groups).	○	○	○	○	○

Woolf K, *et al. BMJ Open* 2021; 11:e044753. doi: 10.1136/bmjopen-2020-044753

3.a. If you think there are other measures that could be used by medical schools to select applicants, please explain by typing in the box:

4. The cancellation of exams could potentially result in medical schools having many more students than they had planned for. In that situation, how acceptable or unacceptable are each of the following for medical schools to do:

	Completely unacceptable	Slightly unacceptable	Neutral	Slight acceptable	Completely acceptable
Accept all applicants whose calculated grades meet the conditional offer, although it could mean fewer resources per student.					
Raise conditional offers for all applicants (e.g. an AAA offer becomes AAA*).					
Raise conditional offers for applicants with lower interview scores and/or admissions test results.					
Raise conditional offers for applicants from schools that tend to give higher predicted grades than their students typically achieve.					
Ask some applicants with offers to volunteer to defer a year.					
Require some applicants with offers to defer for a year.					

4.a. If you have any other comments about how medical schools should cope with having many more students than places, please type it into the box:

Looking to academic year 2020/2021

5. The coronavirus outbreak could have a knock-on effect for medical school selection in 2021. Please indicate how much you agree or disagree with the following statements about what medical schools should do in selection next year:

Current applicants re-applying next year should not be treated specially as that would not be fair for those applying for the first time next year.	Please select
Medical schools need to change their selection system for next year to give priority to applicants who are rejected this year.	Please select
Applicants with conditional offers rejected this year should automatically be given conditional offers next year regardless of any grades they are awarded this year.	Please select
Applicants rejected this year should be given special consideration when re-applying next year.	Please select
Applicants rejected this year should be required to take A-levels next year when they are running normally.	Please select
Applicants rejected this year should apply next year in the usual way and be considered with all other applicants.	Please select

6. It is possible that universities will not start the next academic year at the usual time in October 2020. In that case, which of the following should universities do?

- Start the academic year on time using distance learning for as long as is necessary.
- Defer the start of the academic year only when face-to-face teaching is possible.

Calculated Grades

Now that examinations have been cancelled, students will be awarded 'calculated grades'. We are interested in your opinion on this. Before giving us your opinion, please read the information below about how calculated grades will be awarded.

For each examination a student takes:

- Teachers will judge the grade a student would have got had they had taken the examination (for example A*, A, B, etc); AND where that student ranks compared to other students also getting that grade. For example, a student judged to get a B in Chemistry will be compared to all others also judged to get a B in Chemistry, with all students being ranked in order (1st, 2nd, 3rd etc). Teachers will do this after 29th May 2020.
    - Judgements will NOT be based on predicted grades submitted to UCAS. Instead they will be based on evidence such as classwork, homework, mock examinations, coursework etc.
    - Judgements are not told to students and are passed confidentially to exam boards and Ofqual (the English exam regulator).
- A final grade is then awarded by exam boards and Ofqual, based on the teacher judgement and other information about the school/college a student attends, such as how students at that school performed in previous years.

There will be an appeals process and it is possible students will be able to sit the examinations when schools open again, although that is still uncertain.

Full details of the process here https://www.gov.uk/government/publications/gcses-as-and-a-level-awarding-summer-2020. Please note the process may differ outside of England. However if you are outside of England please answer the questions as if the same arrangements were to apply to the exams you are taking.

7. Please rate how much you *agree* or *disagree* with each of the following statements about using calculated grades:

More info

	Strongly disagree	Somewhat disagree	Neutral	Somewhat agree	Strongly agree
I would prefer not to have calculated grades at all and instead take A levels (or equivalents) in September.	☐	☐	☐	☐	☐
Overall, I would prefer to withdraw entirely from calculated grades and sit exams properly next summer.	☐	☐	☐	☐	☐
The process described above is the best way to be fair to most students.	☐	☐	☐	☐	☐
The process described above ensures that schools cannot get an advantage by giving all of their students high calculated grades.	☐	☐	☐	☐	☐
I feel confident this process will result in an accurate assessment of my true abilities.	☐	☐	☐	☐	☐
It is unfair to judge students based on the work they have done since schools/colleges closed due to coronavirus.	☐	☐	☐	☐	☐
It seems unfair that my attainment at GCSE (or equivalent) is not taken into account.	☐	☐	☐	☐	☐
Many students do better than their teachers expect; calculated grades cannot take that into account.	☐	☐	☐	☐	☐
My teachers should take into account the disruption caused by coronavirus when judging grades.	☐	☐	☐	☐	☐
Calculated grades should be based only on my performance, not on how previous students at my school performed.	☐	☐	☐	☐	☐

Woolf K, *et al. BMJ Open* 2021; 11:e044753. doi: 10.1136/bmjopen-2020-044753

8. Please rate how much you *agree* or *disagree* with each of the following statements about calculated grades:

	Strongly disagree	Somewhat disagree	Neutral	Somewhat agree	Strongly agree
I am confident in my teachers' abilities at grading and ranking students.	☐	☐	☐	☐	☐
My teachers do not know enough about me to grade and rank me accurately.	☐	☐	☐	☐	☐
In large schools/colleges, it is difficult to see how teachers can rank so many students.	☐	☐	☐	☐	☐
Teachers judging grades should take into account the fact that many students do not do well in mocks but then work hard and do well in exams.	☐	☐	☐	☐	☐
Teachers should find it easy to account for any special needs and reasonable adjustments a student has when judging grades.	☐	☐	☐	☐	☐
Teachers will find it difficult to provide objective unbiased judgements of their students' performance.	☐	☐	☐	☐	☐
Appeals against calculated grades are unlikely to be successful as they will only be my opinion against my teachers'.	☐	☐	☐	☐	☐
Employers and universities in the future will treat grades from 2020 differently compared to exam grades taken from other years.	☐	☐	☐	☐	☐

Your application to university

9. **Did you apply to university this academic year (2019/2020)?** ✱ *Required*

- Yes applied to study medicine
- Yes applied but NOT to any medical courses
- No

Woolf K, *et al. BMJ Open* 2021; 11:e044753. doi: 10.1136/bmjopen-2020-044753

Your university choices

10. **Please indicate which universities and courses you have applied to, the response you have had from each, and type in any offers you have received.** For the course, please type in either the UCAS course code (e.g. A100, B900) or the course name (e.g. Standard Entry Medicine, Biomedical Science).

	University name	Course	What response have you had from this university?	Please type in your conditional offer (if applicable):
1st Choice			Please select	
2nd Choice			Please select	
3rd Choice			Please select	
4th Choice			Please select	
5th Choice			Please select	

Communication from universities about changes to selection

For each of your choices: has this university been in touch with you about any changes they are making to their selection processes due to coronavirus/COVID-19?

11. Your Choice 1: has this university told you about any changes they are making to their selection processes due to coronavirus/COVID-19?

11.a. If you selected Other, please specify:

12. Your Choice 2: has this university told you about any changes they are making to their selection processes due to coronavirus/COVID-19?

12.a. If you selected Other, please specify:

13. Your Choice 3: has this university told you about any changes they are making to their selection processes due to coronavirus/COVID-19?

13.a. If you selected Other, please specify:

14. Your Choice 4: has this university told you about any changes they are making to their selection processes due to coronavirus/COVID-19?

Woolf K, *et al. BMJ Open* 2021; 11:e044753. doi: 10.1136/bmjopen-2020-044753

14.a. If you selected Other, please specify:

15. Your Choice 5: has this university told you about any changes they are making to their selection processes due to coronavirus/COVID-19?

15.a. If you selected Other, please specify:

Your education and qualifications

To put into context the answers to the earlier questions it would be helpful if we knew more about your educational qualifications, both those you were due to take this year, and those you have taken in previous years.

16. Which one of the following best describes you? ✱ *Required*

- ○ In Year 13 (S6) of school/college
- ○ Was in Year 13 (S6) of school/college last academic year (2018/2019)
- ○ In the final year of a degree programme
- ○ Have a degree already
- ○ Mature applicant without degree
- ○ Other

16.a. If you selected Other, please specify:

Woolf K, *et al. BMJ Open* 2021; 11:e044753. doi: 10.1136/bmjopen-2020-044753

Your university

17. Please type in the name of your the university and course and subject you are currently studying for or have already gained (e.g. University of Bath, BSc Biomedical Science 2.1). If you have more than one degree, please type each in.

18. If you are currently in the final year of your degree, has your university told you how they will determine your final degree mark (please choose one)? If you are not in your final year please select 'Not applicable'.

- No
- Yes - please give details below
- Uncertain
- Other - please give details below
- Not applicable

19. If you selected Yes or Other to the question above, please give details in the box:

Woolf K, *et al. BMJ Open* 2021; 11:e044753. doi: 10.1136/bmjopen-2020-044753

Your school/college

Please type in the full name and first part of the postcode of the school or college you are currently attending. If you are not at school or college, please type in the details of your last school/college.

20. Name of school/college (e.g. St Mary's C of E School, Kilburn):

20.a. First part of postcode of school/college (e.g. NW6):

Woolf K, *et al. BMJ Open* 2021; 11:e044753. doi: 10.1136/bmjopen-2020-044753

Your qualifications

21. **Please tell us about the A levels, Scottish Highers or equivalent qualifications you are currently taking and/or have already taken (most A level students will have 3 subjects). For each qualification, please indicate the qualification type, subject, grade (predicted or achieved), and the year in which you are expected to achieve/achieved the qualification.**

	Qualification type	Subject	Predicted grade	Achieved grade	Year
1	Please select				
2	Please select				
3	Please select				
4	Please select				
5	Please select				
6	Please select				
7	Please select				
8	Please select				
9	Please select				
10	Please select				

Woolf K, *et al. BMJ Open* 2021; 11:e044753. doi: 10.1136/bmjopen-2020-044753

Admission tests: UCAT

In this section please give us some basic information about the various admissions tests such as UCAT, BMAT and GAMSAT that you may have taken.

If you are not certain of precise scores then put in approximate values.

22. Did you take the UCAT (University Clinical Aptitude Test) in 2019?

- ○ Yes
- ○ No

Woolf K, *et al. BMJ Open* 2021; 11:e044753. doi: 10.1136/bmjopen-2020-044753

UCAT score

23. **What was your total UCAT score and your score on each UCAT subsection (please type)?** *If unsure, type "unsure". If you have taken UCAT more than once, please give your most recent scores. Please estimate if you cannot remember your exact scores.*

	Score
Total UCAT score	
Verbal Reasoning	
Decision Making	
Quantitative Reasoning	
Abstract Reasoning	
Situational Judgement	

Woolf K, *et al. BMJ Open* 2021; 11:e044753. doi: 10.1136/bmjopen-2020-044753

Admissions tests: BMAT

24. Did you take the BMAT (BioMedical Admissions Test) in 2019?

- ○ Yes
- ○ No

Woolf K, *et al. BMJ Open* 2021; 11:e044753. doi: 10.1136/bmjopen-2020-044753

BMAT score

25. **What was your score on each BMAT subsection (please type)?** If you have taken BMAT more than once, please give your most recent scores. If you did not take the test, please type "na". *If unsure or unknown please type "unsure". Please estimate if you cannot remember your exact scores.*

	Score
Section 1	
Section 2	
Section 3	

Woolf K, *et al. BMJ Open* 2021; 11:e044753. doi: 10.1136/bmjopen-2020-044753

Admissions test: GAMSAT

26. Did you take the GAMSAT (Graduate Medical School Admissions Test) in 2018 or 2019?

- Yes
- No

Woolf K, *et al. BMJ Open* 2021; 11:e044753. doi: 10.1136/bmjopen-2020-044753

GAMSAT score

27. **What was your overall GAMSAT score and your score on each GAMSAT section (please type)?** If you have taken GAMSAT more than once, please give your most recent scores. *If you are unsure what your scores were, type "unsure". Please estimate if you cannot remember your exact scores.*

	Score
Overall score	
Section I	
Section II	
Section III	

Woolf K, *et al. BMJ Open* 2021; 11:e044753. doi: 10.1136/bmjopen-2020-044753

Impact of coronavirus/COVID-19 on your day to day life

It would be helpful to understand how applicants' lives and education are being affected by coronavirus/COVID-19 so medical schools can take this into account in selection if necessary. Please be assured all your answers are kept confidential.

If you want support dealing with this difficult time please contact the organisations listed here: https://www.nhs.uk/conditions/stress-anxiety-depression/mental-health-helplines/ or Childline on 0800 1111 https://www.childline.org.uk/.

28. On a typical day in the past five days, about how much time have you spent on the following?

	Time per day						
	Zero mins	1- 10 mins	11-30 mins	31-60 mins	1 to 2 hours	2 to 4 hours	4+ hours
Reading about coronavirus/COVID-19	○	○	○	○	○	○	○
Phoning/texting/video calling your friends	○	○	○	○	○	○	○
Watching TV or videos	○	○	○	○	○	○	○
Gaming online with friends	○	○	○	○	○	○	○
Gaming alone	○	○	○	○	○	○	○
Reading for pleasure	○	○	○	○	○	○	○
Exercising	○	○	○	○	○	○	○
Other hobbies	○	○	○	○	○	○	○
Studying	○	○	○	○	○	○	○
Household chores/shopping for necessities	○	○	○	○	○	○	○
Caring for members of your household	○	○	○	○	○	○	○
Volunteering	○	○	○	○	○	○	○

Your education during the coronavirus outbreak

29. If you are currently in education which of the following is your school/college/uni providing? For each activity, please indicate whether you have used it or not. If you have not used it, please tell us why not. If you are not currently in education, please leave these questions blank.

		If you have not used this, why not?
Online teaching in real time using video (e.g. Zoom, Skype, Teams).	Please select	Please select
Online resources for home learning (e.g. links to activities, apps).	Please select	Please select
Paper resources for home learning (e.g. workbooks).	Please select	Please select
Online exams/assessments that count toward your calculated grades/degree award.	Please select	Please select
Online assessments that don't count towards your calculated grades/degree award (e.g. quizzes).	Please select	Please select
Pastoral support (e.g. asking about mental health, providing mental health resources, checking on home circumstances and suitability for studying).	Please select	Please select

Woolf K, *et al. BMJ Open* 2021; 11:e044753. doi: 10.1136/bmjopen-2020-044753

Support with your university application (e.g. choosing between firm and insurance choices).	Please select	Please select
Other.	Please select	Please select

29.a. If you selected Other reasons then please specify here:

30. Will your school/college/uni formally assess you on any work you do or have done since schools/unis closed? For example, to inform your calculated grades or to determine your degree class, if applicable? If you are not currently in education please select 'Not applicable'.

- Yes
- No
- Uncertain
- Not applicable

Preparing for medical school or university

31. Other than schoolwork or studying for your degree (if relevant) are you doing any preparation for medical school or university? Please select all that apply:

- ☐ Watching online lectures
- ☐ Online private tutoring
- ☐ Reading textbooks
- ☐ Reading other books
- ☐ Volunteering in a clinical environment
- ☐ Volunteering in a non-clinical environment
- ☐ Exam preparation (e.g. timed essays, past papers)
- ☐ Talking to friends
- ☐ Other
- ☐ Not doing any preparation

31.a. If you selected Other, please specify:

32. If you are not doing any preparation, please tell us why not (please select all that apply):

- ☐ I'm not going to university this year
- ☐ Don't have enough time
- ☐ Can't focus because too worried
- ☐ Don't have any resources
- ☐ I'm caring for others
- ☐ I'm unwell
- ☐ It's not necessary
- ☐ Other

Your general approach to life

The following questions are about how you behave and how you see yourself as a person. Please select the response which best describes how you see yourself, where 1 means does not apply to me at all and 7 means applies to me perfectly. Don’t think too hard before answering, just give the first answer that comes to mind.

33. *I see myself as someone who...*

	1=Does not apply to me at all. 7=Applies to me perfectly.						
	1	2	3	4	5	6	7
is sometimes rude to others	○	○	○	○	○	○	○
does a thorough job	○	○	○	○	○	○	○
is talkative	○	○	○	○	○	○	○
worries a lot	○	○	○	○	○	○	○
is original, comes up with new ideas	○	○	○	○	○	○	○
has a forgiving nature	○	○	○	○	○	○	○
tends to be lazy	○	○	○	○	○	○	○
is outgoing, sociable	○	○	○	○	○	○	○
gets nervous easily	○	○	○	○	○	○	○
values artistic, aesthetic experiences	○	○	○	○	○	○	○
is considerate and kind to almost everyone	○	○	○	○	○	○	○
does things efficiently	○	○	○	○	○	○	○
is reserved	○	○	○	○	○	○	○
is relaxed, handles stress well	○	○	○	○	○	○	○
has an active imagination	○	○	○	○	○	○	○

Woolf K, *et al. BMJ Open* 2021; 11:e044753. doi: 10.1136/bmjopen-2020-044753

Your parent/carers' occupations and education

This section has some questions about your parents' or carers' occupation(s) and your household. Your answers will help us understand how family background may affect entry to medical school.

Please select the option(s) that best describes the occupation(s) of your parent(s)/carer(s).

If your parent(s)/carer(s) are not currently working please choose what they were employed as.

If you are no longer living with your parent(s) or carer(s), or are unsure what their occupation is, please still answer these questions as best you can.

34. Occupation of Parent/Carer 1:

34.a. If you selected Other, please specify:

35. Occupation of Parent/Carer 2:

35.a. If you selected Other, please specify:

36. Does one or more of your parent(s)/carer(s) have a university degree?

37. Is one or more of your parent(s)/carer(s) a medical doctor?

Your identification details

Please complete this section to enable the research team to use your survey responses as described in the Information Sheet. Remember, your information is kept confidential and stored securely.

Please type your FULL NAME [first name(s) and surname] in the boxes below.

38. First name:

39. Surname:

40. Please type your **date of birth** using the format DD (day) MM (month) YYYY (year).

(dd/mm/yyyy)

41. Which of the following best describes the gender you identify as?

41.a. If you selected Other, please specify:

Woolf K, *et al. BMJ Open* 2021; 11:e044753. doi: 10.1136/bmjopen-2020-044753

Additional comments

42. Please use this space for any additional comments you wish to make about the questionnaire or selection of medical students:

43. These are unprecedented times. Please tell us about your **hopes for now and the future**, and tell us what **inspires** you.

Thank you for completing this questionnaire.

If you wish to go back and review your answers then please do so now.

When you click on the finish button below, your responses will be submitted to the UKMACS research team.

Woolf K, *et al. BMJ Open* 2021; 11:e044753. doi: 10.1136/bmjopen-2020-044753

Thank You

Thank you for completing this questionnaire for UKMACS.

You can share this questionnaire with other medical applicants: **https://ucl.onlinesurveys.ac.uk/ukmacs-covid19-questionnaire**.

There is also more information on our website:https://ukmacs.wordpress.com/

If you have any questions about the study or this questionnaire, please contact the UKMACS Research Team medsch.choice@ucl.ac.uk

---

Key for selection options

5.1.a -

Strongly disagree
Disagree
Neutral
Agree
Strongly agree

5.2.a -

Strongly disagree
Disagree
Neutral
Agree
Strongly agree

5.3.a -

Strongly disagree
Disagree
Neutral
Agree
Strongly agree

5.4.a -

Strongly disagree
Disagree
Neutral
Agree
Strongly agree

5.5.a -

Strongly disagree
Disagree
Neutral
Agree
Strongly agree

5.6.a -

Strongly disagree
Disagree
Neutral
Agree
Strongly agree

Woolf K, *et al. BMJ Open* 2021; 11:e044753. doi: 10.1136/bmjopen-2020-044753

10.1.c - What response have you had from this university?

Rejected without interview/MMI
Rejected after interview/MMI
Waiting to hear if I will be interviewed
Due to be interviewed
Interviewed and waiting to hear if I have an offer
Conditional offer (please type offer in box on the right)
Unconditional offer
Other

10.2.c - What response have you had from this university?

Rejected without interview/MMI
Rejected after interview/MMI
Waiting to hear if I will be interviewed
Due to be interviewed
Interviewed and waiting to hear if I have an offer
Conditional offer (please type offer in box on the right)
Unconditional offer
Other

10.3.c - What response have you had from this university?

Rejected without interview/MMI
Rejected after interview/MMI
Waiting to hear if I will be interviewed
Due to be interviewed
Interviewed and waiting to hear if I have an offer
Conditional offer (please type offer in box on the right)
Unconditional offer
Other

10.4.c - What response have you had from this university?

Rejected without interview/MMI
Rejected after interview/MMI
Waiting to hear if I will be interviewed
Due to be interviewed
Interviewed and waiting to hear if I have an offer
Conditional offer (please type offer in box on the right)
Unconditional offer
Other

10.5.c - What response have you had from this university?

Rejected without interview/MMI
Rejected after interview/MMI
Waiting to hear if I will be interviewed
Due to be interviewed
Interviewed and waiting to hear if I have an offer
Conditional offer (please type offer in box on the right)
Unconditional offer
Other

11 - Your Choice 1: has this university told you about any changes they are making to their selection processes due to coronavirus/COVID-19?

No
Yes, said changes will be happening but did not specify
Yes, specified changes
Other

Woolf K, *et al. BMJ Open* 2021; 11:e044753. doi: 10.1136/bmjopen-2020-044753

12 - Your Choice 2: has this university told you about any changes they are making to their selection processes due to coronavirus/COVID-19?

No
Yes, said changes will be happening but did not specify
Yes, specified changes
Other

13 - Your Choice 3: has this university told you about any changes they are making to their selection processes due to coronavirus/COVID-19?

No
Yes, said changes will be happening but did not specify
Yes, specified changes
Other

14 - Your Choice 4: has this university told you about any changes they are making to their selection processes due to coronavirus/COVID-19?

No
Yes, said changes will be happening but did not specify
Yes, specified changes
Other

15 - Your Choice 5: has this university told you about any changes they are making to their selection processes due to coronavirus/COVID-19?

No
Yes, said changes will be happening but did not specify
Yes, specified changes
Other

21.1.a - Qualification type

A-Level
AS level
Scottish Higher
Scottish Advanced Higher
International Baccalaureate
Welsh Baccalaureate
Cambridge Pre-U
Irish Leaving Certificate
European Baccalaureate
Access to HE Diploma
BTEC National Diploma
Extended Project Qualification
Degree
Postgraduate qualification
Other

21.2.a - Qualification type

A-Level
AS level
Scottish Higher
Scottish Advanced Higher
International Baccalaureate
Welsh Baccalaureate
Cambridge Pre-U
Irish Leaving Certificate
European Baccalaureate
Access to HE Diploma

BTEC National Diploma
Extended Project Qualification
Degree
Postgraduate qualification
Other

21.3.a - Qualification type

A-Level
AS level
Scottish Higher
Scottish Advanced Higher
International Baccalaureate
Welsh Baccalaureate
Cambridge Pre-U
Irish Leaving Certificate
European Baccalaureate
Access to HE Diploma
BTEC National Diploma
Extended Project Qualification
Degree
Postgraduate qualification
Other

21.4.a - Qualification type

A-Level
AS level
Scottish Higher
Scottish Advanced Higher
International Baccalaureate
Welsh Baccalaureate
Cambridge Pre-U
Irish Leaving Certificate
European Baccalaureate
Access to HE Diploma
BTEC National Diploma
Extended Project Qualification
Degree
Postgraduate qualification
Other

21.5.a - Qualification type

A-Level
AS level
Scottish Higher
Scottish Advanced Higher
International Baccalaureate
Welsh Baccalaureate
Cambridge Pre-U
Irish Leaving Certificate
European Baccalaureate
Access to HE Diploma
BTEC National Diploma
Extended Project Qualification
Degree
Postgraduate qualification
Other

Woolf K, *et al. BMJ Open* 2021; 11:e044753. doi: 10.1136/bmjopen-2020-044753

21.6.a - Qualification type

A-Level
AS level
Scottish Higher
Scottish Advanced Higher
International Baccalaureate
Welsh Baccalaureate
Cambridge Pre-U
Irish Leaving Certificate
European Baccalaureate
Access to HE Diploma
BTEC National Diploma
Extended Project Qualification
Degree
Postgraduate qualification
Other

21.7.a - Qualification type

A-Level
AS level
Scottish Higher
Scottish Advanced Higher
International Baccalaureate
Welsh Baccalaureate
Cambridge Pre-U
Irish Leaving Certificate
European Baccalaureate
Access to HE Diploma
BTEC National Diploma
Extended Project Qualification
Degree
Postgraduate qualification
Other

21.8.a - Qualification type

A-Level
AS level
Scottish Higher
Scottish Advanced Higher
International Baccalaureate
Welsh Baccalaureate
Cambridge Pre-U
Irish Leaving Certificate
European Baccalaureate
Access to HE Diploma
BTEC National Diploma
Extended Project Qualification
Degree
Postgraduate qualification
Other

21.9.a - Qualification type

A-Level
AS level
Scottish Higher
Scottish Advanced Higher
International Baccalaureate

Woolf K, *et al. BMJ Open* 2021; 11:e044753. doi: 10.1136/bmjopen-2020-044753

Welsh Baccalaureate
Cambridge Pre-U
Irish Leaving Certificate
European Baccalaureate
Access to HE Diploma
BTEC National Diploma
Extended Project Qualification
Degree
Postgraduate qualification
Other

21.10.a - Qualification type

A-Level
AS level
Scottish Higher
Scottish Advanced Higher
International Baccalaureate
Welsh Baccalaureate
Cambridge Pre-U
Irish Leaving Certificate
European Baccalaureate
Access to HE Diploma
BTEC National Diploma
Extended Project Qualification
Degree
Postgraduate qualification
Other

29.1.a -

Used
Not used

29.1.b - If you have not used this, why not?

Not available
Not enough private/quiet space
Not enough time
Find it hard to focus
No access to computer/internet
Don't need to do this
Other reason(s)

29.2.a -

Used
Not used

29.2.b - If you have not used this, why not?

Not available
Not enough private/quiet space
Not enough time
Find it hard to focus
No access to computer/internet
Don't need to do this
Other reason(s)

29.3.a -

Woolf K, *et al. BMJ Open* 2021; 11:e044753. doi: 10.1136/bmjopen-2020-044753

Used
Not used

29.3.b - If you have not used this, why not?
Not available
Not enough private/quiet space
Not enough time
Find it hard to focus
No access to computer/internet
Don't need to do this
Other reason(s)

29.4.a -
Used
Not used

29.4.b - If you have not used this, why not?
Not available
Not enough private/quiet space
Not enough time
Find it hard to focus
No access to computer/internet
Don't need to do this
Other reason(s)

29.5.a -
Used
Not used

29.5.b - If you have not used this, why not?
Not available
Not enough private/quiet space
Not enough time
Find it hard to focus
No access to computer/internet
Don't need to do this
Other reason(s)

29.6.a -
Used
Not used

29.6.b - If you have not used this, why not?
Not available
Not enough private/quiet space
Not enough time
Find it hard to focus
No access to computer/internet
Don't need to do this
Other reason(s)

29.7.a -
Used
Not used

Woolf K, *et al. BMJ Open* 2021; 11:e044753. doi: 10.1136/bmjopen-2020-044753

29.7.b - If you have not used this, why not?
Not available
Not enough private/quiet space
Not enough time
Find it hard to focus
No access to computer/internet
Don't need to do this
Other reason(s)

29.8.a -
Used
Not used

29.8.b - If you have not used this, why not?
Not available
Not enough private/quiet space
Not enough time
Find it hard to focus
No access to computer/internet
Don't need to do this
Other reason(s)

34 - Occupation of Parent/Carer 1:
Modern Professional (e.g. teacher, nurse, social worker, artist, police officer (sergeant or above))
Clerical & intermediate (e.g. secretary, call centre agent, nursing auxiliary, nursery nurse)
Senior managers or administrators (e.g. finance manager, chief executive)
Technical & craft (e.g. motor mechanic, plumber, printer, tool maker, gardener, train driver, fitter)
Semi-routine manual & service (e.g. postal / farm worker, security guard, catering/sales assistant)
Routine manual & service (e.g. HGV driver, cleaner, porter, sewing machinist, bar staff, labourer)
Middle or junior managers (e.g. office / retail / bank / restaurant / warehouse manager, publican)
Traditional professional (e.g. accountant, solicitor, medical practitioner, scientist, civil servant)
Never worked
Do not know
Information withheld
Other

35 - Occupation of Parent/Carer 2:
Modern Professional (e.g. teacher, nurse, social worker, artist, police officer (sergeant or above))
Clerical & intermediate (e.g. secretary, call centre agent, nursing auxiliary, nursery nurse)
Senior managers or administrators (e.g. finance manager, chief executive)
Technical & craft (e.g. motor mechanic, plumber, printer, tool maker, gardener, train driver, fitter)
Semi-routine manual & service (e.g. postal / farm worker, security guard, catering/sales assistant)
Routine manual & service (e.g. HGV driver, cleaner, porter, sewing machinist, bar staff, labourer)
Middle or junior managers (e.g. office / retail / bank / restaurant / warehouse manager, publican)
Traditional professional (e.g. accountant, solicitor, medical practitioner, scientist, civil servant)
Never worked
Do not know
Information withheld
Not applicable
Other

36 - Does one or more of your parent(s)/carer(s) have a university degree?
Yes, one has a degree
Yes, both have degrees
No
Rather not say

Woolf K, *et al. BMJ Open* 2021; 11:e044753. doi: 10.1136/bmjopen-2020-044753

37 - Is one or more of your parent(s)/carer(s) a medical doctor?

Yes, one is a medical doctor
Yes, both are medical doctors
No
Rather not say

41 - Which of the following best describes the gender you identify as?

Male
Female
Prefer not to answer
Other

Woolf K, *et al. BMJ Open* 2021; 11:e044753. doi: 10.1136/bmjopen-2020-044753
