# Supplementary material for: The attitudes, perceptions and experiences of medical school applicants following the closure of schools and cancellation of public examinations in 2020 due to the COVID-19 pandemic: a cross-sectional questionnaire study of UK medical applicants
Source: BMJ Open. 2021 Mar 4;11(3):e044753. doi: 10.1136/bmjopen-2020-044753 (PMC7934197; doi:10.1136/bmjopen-2020-044753)
Supplement: Supplementary data [file bmjopen-2020-044753supp002.pdf]

Supplementary file 1: Missing data.

The analysis considered 120 measures in the restricted sample, divided into:

1. *Questionnaire items.* The questionnaire asked about attitudes to 87 different topics concerning medical school entrance. Of 153,076 data points, 10788 (7.2%) were missing. For the individual variables, the median percentage of missing data values was 0.48%, with 75 measures having fewer than 5% of missing values.

2. *Demographic and educational items*. For 12 demographic measures, 462 of 18744 measures were missing (2.5%), with a median of 1.0% per measure, and 11 measures having fewer than 5% missing values. Ethnic origin was not asked about in the present study. The ethnicity of 889 respondents who had reported it in a previous questionnaire were imported into the present dataset; 43.1% of ethnicity measures were therefore missing. IMD_Quintile was obtained from postcodes in England, Wales and Scotland, and was missing in 14.8% of cases.

There were four educational attainment items (grades in the highest-scoring 'top' three predicted A-level grades, UCAT score, BMAT score, and mean GCSE grade). Top three predicted A-level grades were present for all because the sample was based on that criterion. Of the remaining three measures, 1852 out of 4686 (39.5%) were missing: UCAT scores were missing in 13.6% of cases, and BMAT scores in 61.3% of cases, but in both cases missing values were mostly structurally missing, candidates mostly having taken only one aptitude test or the other. Mean GCSE grade was missing in 43.1% of cases, having been imported from a previous UKMACS questionnaire.

Participants self-reported their current or most recent school in the current questionnaire. This question was also present in the Wave 1 UKMACS questionnaire. For schools in England, publicly-available administrative data were available on school type (e.g. independent, voluntary aided) and for state-funded schools there were data on whether the admissions policy was selective or non-selective. These were combined to create a binary variable of School Type (non-selective state schools vs private/selective schools) for 1132 respondents (27.1% missing). A composite variable was created using present responses and the responses in the Wave 1 questionnaire, so data were available for 1158 respondents with values missing in 25.9% of respondents.

Missing values were imputed using the *mice* package.(18) Following the general advice of van Buuren (19) missing values were calculated using *pmm* (predictive mean matching), which as van Buuren says, is a good " all-round method with exceptional properties" (p.84). *pmm* is the default method in the *mice()* function for all scale types (binary, ordinal, numeric) and has the advantage that imputed values are always taken from the existing range of actual values in the data, with *pmm* being robust against mis-specification. The number for the pool of candidate donors, *d*, was set at 5, the default in *mice()*, and the number of imputations, *m*, was set at 25.

Regression analyses on the 25 *mira* datasets were carried out using the *lm()* function within the *with()* function, and separate sets of results in the *mipo* dataset were combined with the *pool()* function. Regression analyses entered all socio-demographic and educational predictor variables into the analysis simultaneously, and results are only reported which were significant with p<.01 after taking all other variables into account, so the analysis is relatively conservative. The nine socio-demographic and educational variables used were: ethnicity, gender, school type, parental higher education, IMD quintile, mean GCSE points, mean top three predicted A-levels, UCAT score, number of medical school offers.

Supplementary file 2: Results for the 665 post-Year 13 respondents excluded from the restricted sample.

This sample includes mature and graduate applicants from the whole of the UK.

Applicant views on admissions

Perceptions of the fairness of methods to select or reject offer holders

As with the restricted sample, no single method was perceived as fair enough to use on its own but many were considered fair enough to use in conjunction with others.

Since this group includes those currently at university and graduate applicants, we have included responses to two additional items: *For those in their final year at university, marks earlier in their course*, which was considered very fair by 35% and quite fair by 45%, and *GAMSAT score (for Graduate Entry students)* which was considered very fair by 17.6% and quite fair by 46.8%.

Compared to those in Year 13, *Predicted grades declared on UCAS form* were considered much less fair and *Personal background (e.g. giving an advantage to students from under-represented groups)* was considered by a majority (52.1%) to be very fair/quite fair.

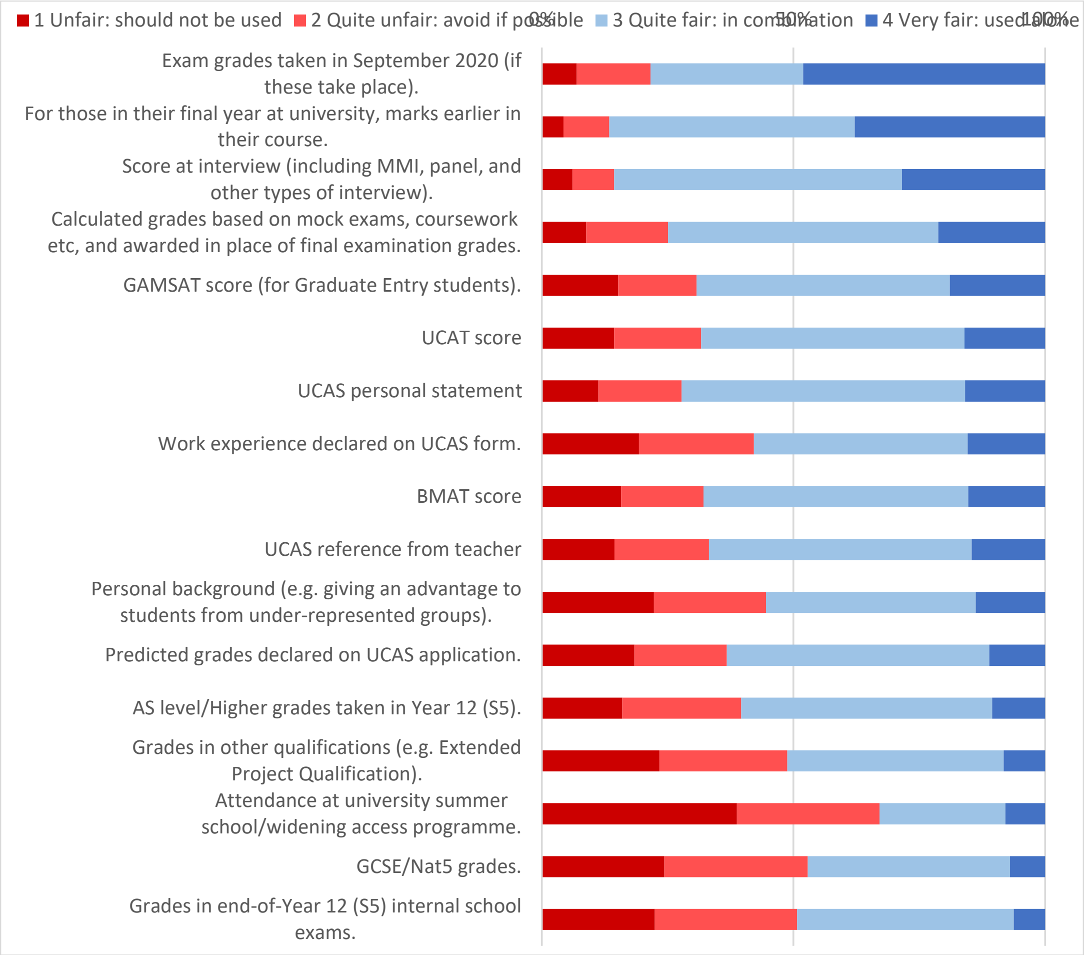

Figure S1: Perceptions of the fairness of methods medical schools could use to decide whether or not to accept applicants who currently hold an offer now that exams have been cancelled. Post-Year13 respondents only.

Acceptability of options for dealing with a situation in which more students meet their offers than there are medical school places

The only option that was rated as slightly or completely acceptable by the majority of respondents (64.6%) was asking for volunteers to defer. Accepting all applicants who meet the conditional offer was the second most acceptable and more acceptable than it was unacceptable.

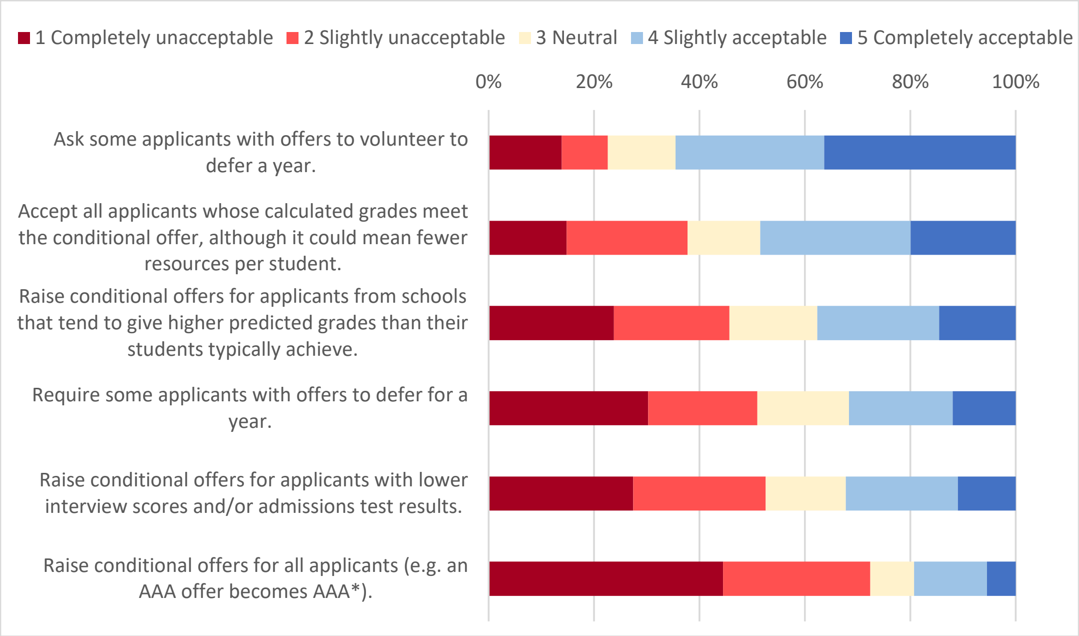

Figure S2: Acceptability of actions medical schools could take if they have more applicants meeting offers than they have places. Post-Year 13 respondents only.

Perceptions of potential impact on admissions for 2021

Respondents were even more divided than in the restricted sample, with about half of respondents (53.8%) agreeing/strongly agreeing that *Applicants rejected this year should be given special consideration when re-applying next year* but 51.5% agreeing/strongly agreeing that *Applicants rejected this year should apply next year in the usual way and be considered with all other applicants*.

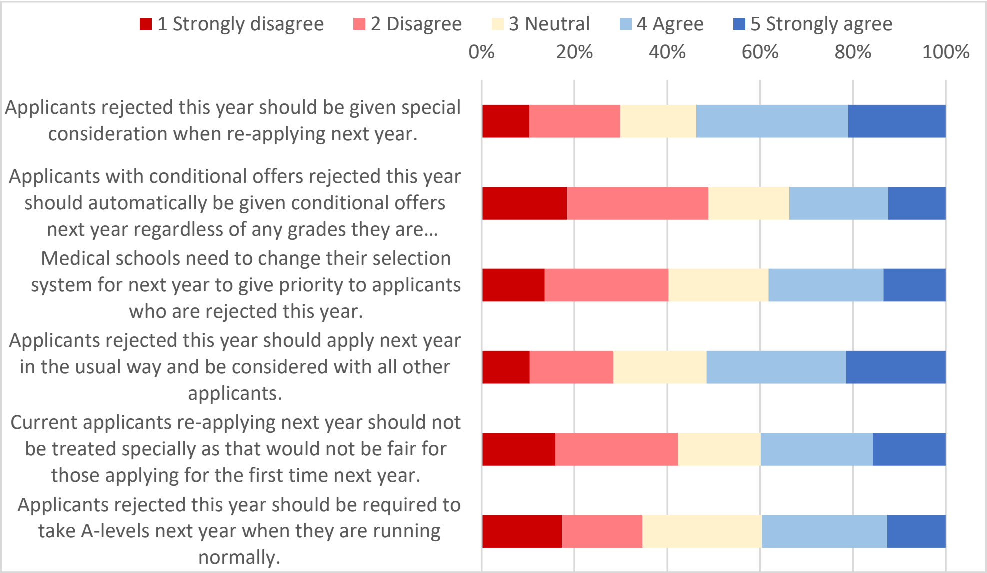

Figure S3: Views on how current applicants should be considered by medical schools if they reapply next year. Post-Year 13 respondents only.

Starting academic year 2020/2021

A majority of respondents (n=375, 56.4%) believed that if necessary, medical schools should *Defer the start of the academic year only when face-to-face teaching is possible* with 285 respondents (42.6.9%) believing that medical schools should *Start the academic year on time using distance learning for as long as is necessary*.

Education and university preparation

Perceptions of process to award calculated grades in lieu of examination grades

Post-Year 13 respondents were generally more negative about calculated grades than respondents in the restricted sample and unsurprisingly there were more “neutral” responses in general and specifically to questions about their own teachers and grades.

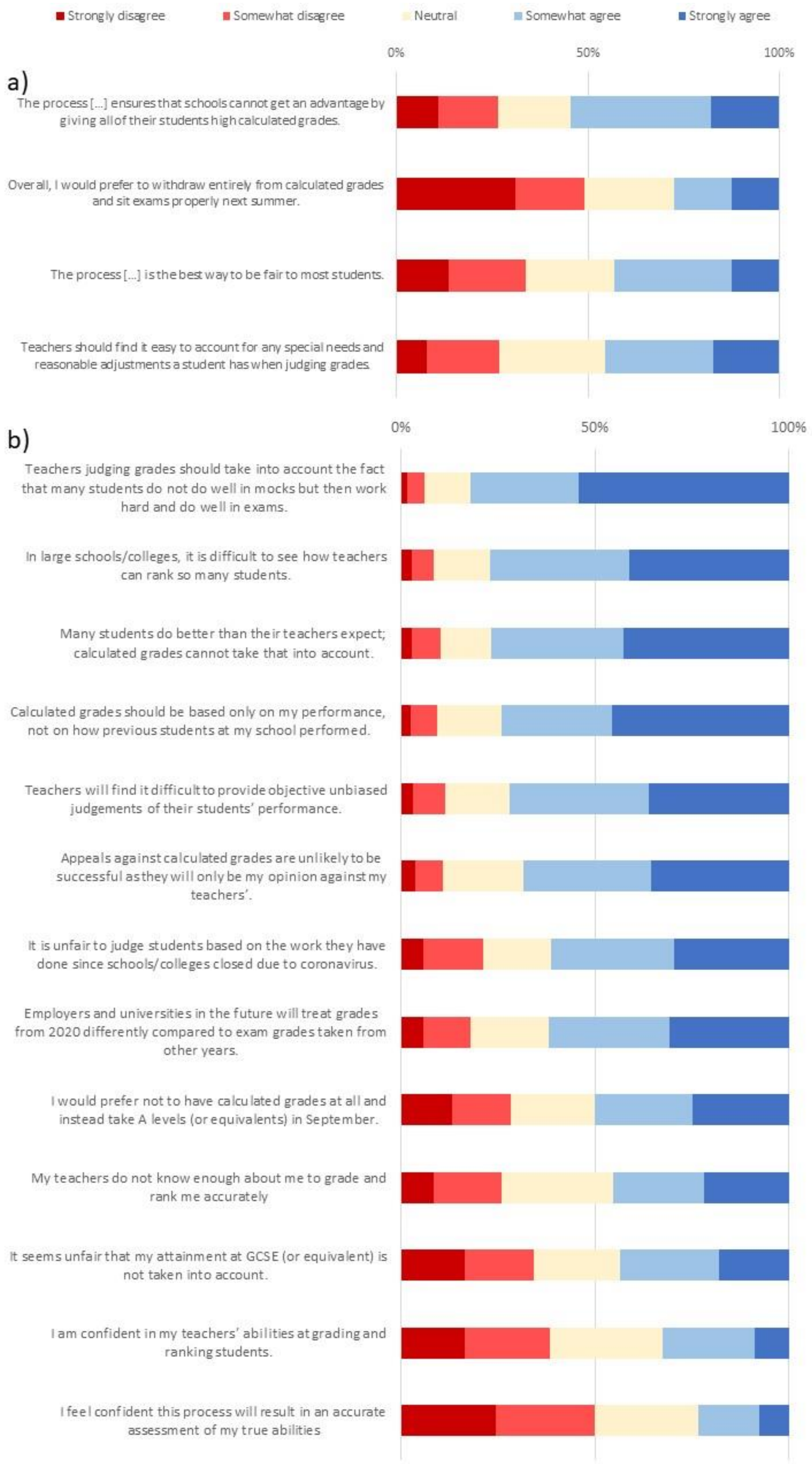

Figure S4: Aspects of calculated grades that respondents were generally more a) positive and b) negative about. Post-Year 13 respondents only.

A number of applicants were re-sitting their examinations and/or were not studying at a school or college but nonetheless were due to take examinations this summer (so-called ‘private candidates’). Several expressed concerns about whether the institution they were due to take their exams with would give them a calculated grade, and if so, what information that grade would be based on:

> *“As a resit student, my previous college which I was registered to retake my exams with this year have decided that they cannot give me calculated grades. I am unsure how to maintain my offers despite not getting grades.”*

> *“I am extremely concerned about how offers made to private candidates who cannot get predicted grades from a school will be treated. Though I had been studying in my lunchtimes/evenings/weekends for over a year, I quit my job 4 days after getting an offer from [redacted] in order to have time to put the work in to get the grades I need. I achieved straight A*s at GCSEs and A-level, so I know how much work it takes to get top grades. I am terrified universities I have offers from will wash their hands of me as I don't have any grades, or forced to defer for a year because universities won't wait for September exam results. Ofqual and exams boards keep saying no student will be disadvantaged, but it appears private candidates like myself may fall through the cracks.”*

> *“I worry that I, as a resitting privately tutored student, will be disadvantaged by the "calculated grades system", as I haven't been in school this year and thus have no exams or schoolwork that could be provided as evidence to support a predicted grade.”*

> *“I’m worried about how they’ll handle resits who have been independently studying as I need to go from a B to an A but am worrying that my old school(exam centre) won’t provide me with a grade even though I’m certain that I’d be able to get an A had I taken the exam. I also can’t afford to take another gap year so I’m hoping unis will take situations like these independently as it would be very unlikely that I’d receive the same grade as last year had I resat.”*

> *“For exam centre who cannot provide grades for resit external students please consider our previous attainment especially if for an applicant like myself has achieved AAB grades from last year and narrowly missed the A grade in Maths by 8 marks. It would be unfair for me to have to take another gap year if I don’t receive a grade this summer.”*

Education since the shutdown

Although participants were post Year-13 many were still in education, whether at school, college or university. The mean number of resources used by participants was 2.9 (SD=1.86).

Like Year 13 respondents, post-Year 13 respondents were using mostly online and paper resources, but 42.8% of post-Year 13 respondents reported having online teaching in real time and nearly half (49.6%) were having online summative tests and; 30.6% reported that their school/college/university would be assessing them formally on work since the closure of schools (although 42.1% reported that this was not applicable to them). See Figure S5.

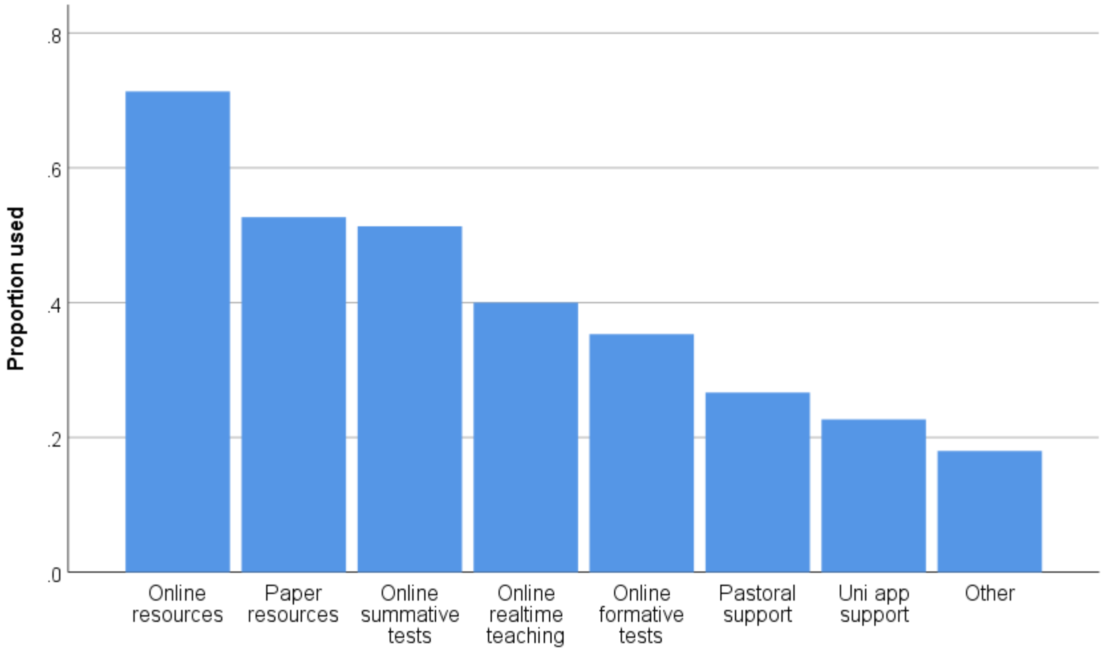

Figure S5: Proportion of post-Year 13 respondents using educational resources since the closure of schools.

Preparation for medical school/university

Post-Year 13 respondents were doing similar sorts of preparation, although they were talking to their friends less. Of the 100 (15.0% of the sample) who said they were not doing any preparation, reasons were different from those in the restricted sample. They were five times more likely to say they did not have time (31.0% vs 6.3%), about half as likely to say they were too worried and not able to focus (26.0% vs 42.5%), and over half as likely to say they did not have resources (15.0% vs 29.5%). A similar percentage selected caring for others as a reason (13.0%), not going to university this year (19.0%), being unwell (6.0%).Respondents could select multiple reasons.

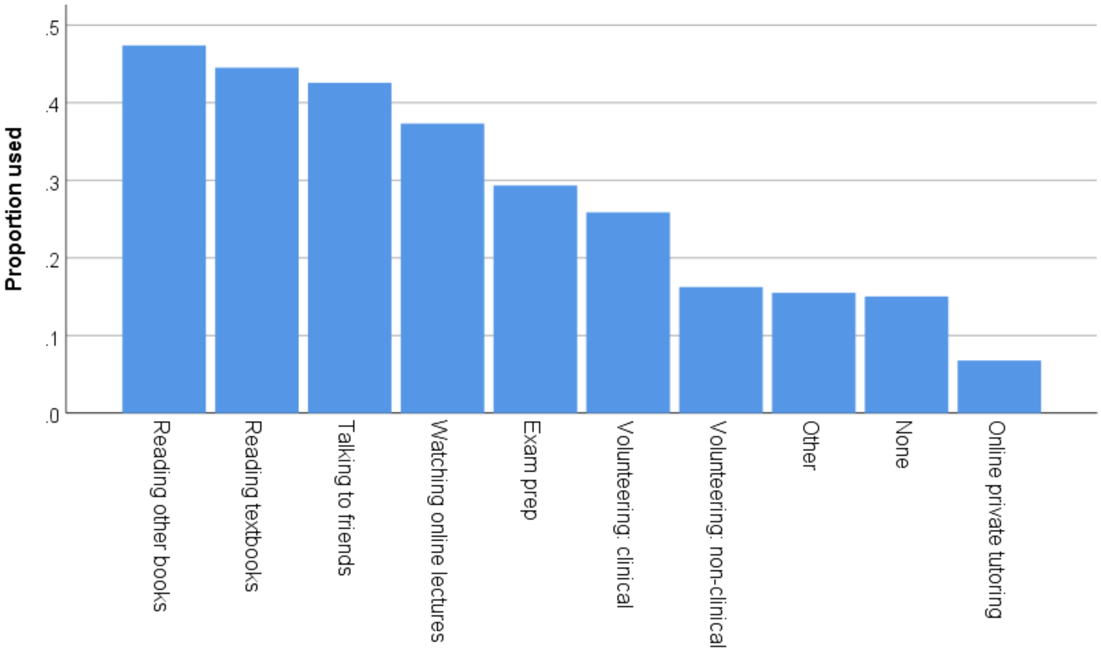

Figure S6: Proportion of respondents undertaking various activities to prepare for medical school or university. Post-Year 13 respondents only.

Time spent during the lockdown

Post-Year 13 respondents were spending broadly similar amounts of time on various activities as those in the restricted sample although they were spending more time volunteering and reading about coronavirus, and less time studying and gaming with friends.

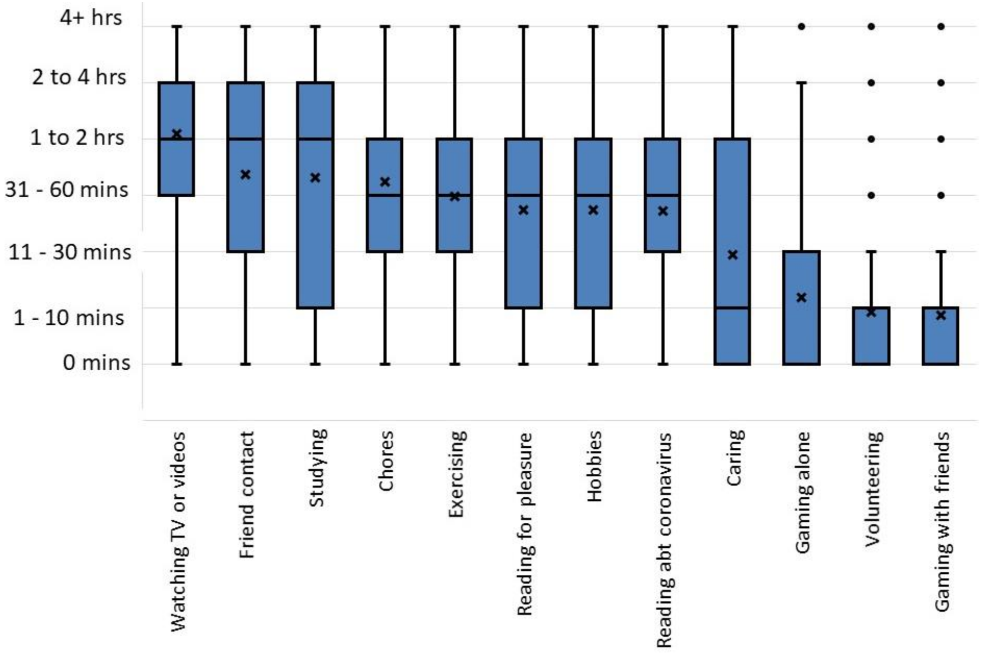

Figure S7: Amount of time respondents reported spending on various activities during the lockdown. Non-Year 13 respondents only.

Supplementary file 3: Results for 125 Scottish S6 respondents excluded from the main analyses

Applicant views on admissions

Perceptions of the fairness of methods to select or reject offer holders

Scottish applicants were similarly uncertain that any measure was fair enough to use alone, however unlike applicants from other UK countries they were more positive about the fairness of using AS level/Higher grades taken in Year 12. This is probably because AS levels are no longer in widespread use whereas Highers are. Scottish applicants were also relatively more positive about the use of calculated grades (83.2% quite or very fair).

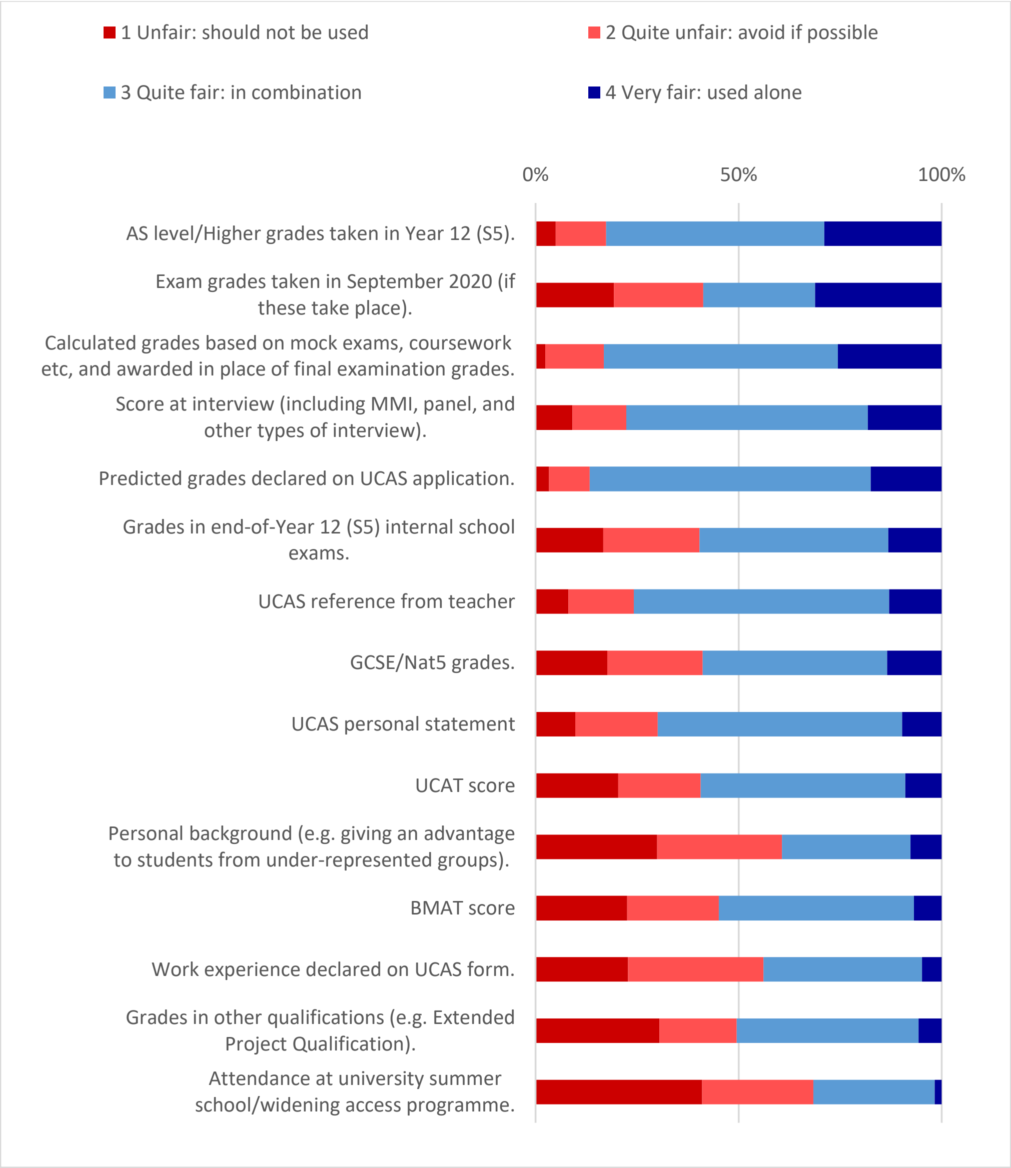

Figure S8: Perceptions of the fairness of methods medical schools could use to decide whether or not to accept applicants who currently hold an offer now that exams have been cancelled. Post-Scottish S6 respondents only.

Acceptability of options for dealing with a situation in which more students meet their offers than there are medical school places

As with other school students, the two acceptable options were accepting all applicants and asking for volunteers to defer.

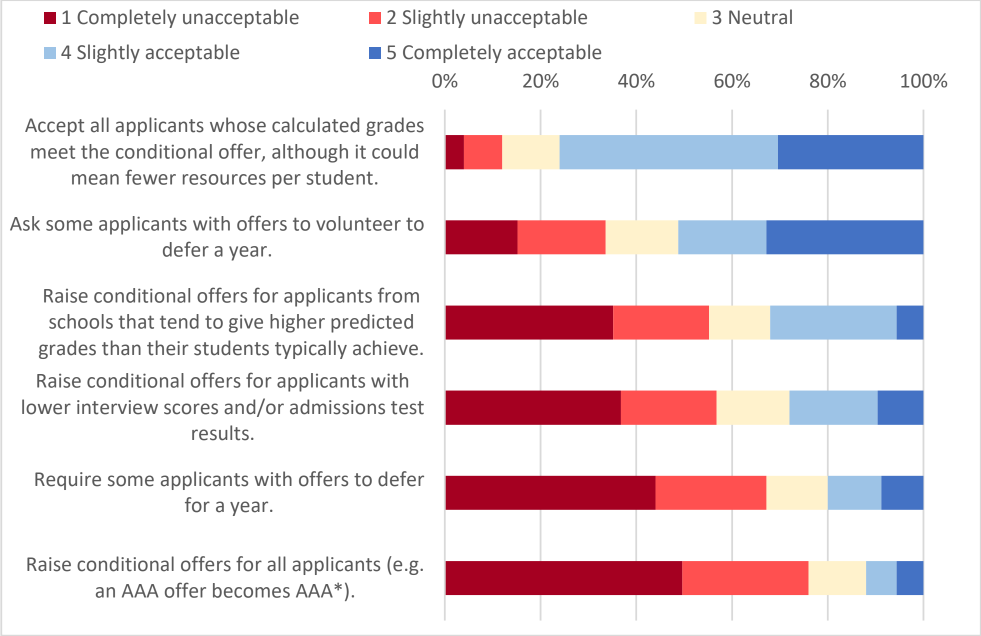

Figure S9: Acceptability of actions medical schools could take if they have more applicants meeting offers than they have places. Scottish S6 respondents only.

Perceptions of potential impact on admissions for 2021

Scottish S6 respondents were even more divided than in other UK countries: half the sample (52.8%) agreed that applicants rejected this year should be given special consideration and half (53.2%) agreeing that they should reapply next year in the usual way and be considered with all other applicants.

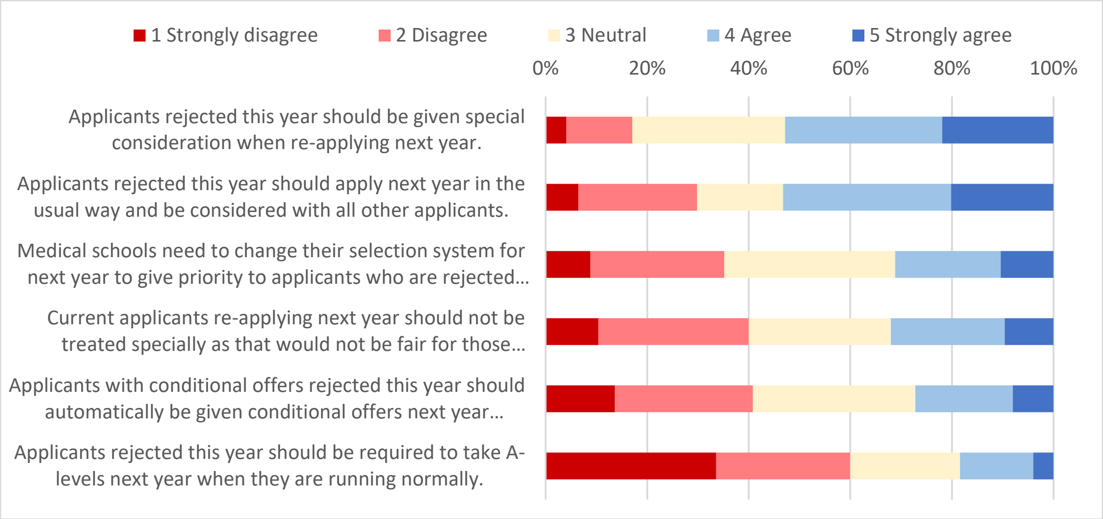

Figure S10: Views on how current applicants should be considered by medical schools if they reapply next year. Scottish S6 respondents only.

Starting academic year 2020/2021

A majority of respondents (n=70; 56.0%) believed that if necessary, medical schools should *Defer the start of the academic year only when face-to-face teaching is possible* with 55 respondents (44.0%) believing that medical schools should *Start the academic year on time using distance learning for as long as is necessary*.

Education and university preparation

Perceptions of process to award calculated grades in lieu of examination grades

Scottish respondents were generally slightly more positive about calculated grades than their equivalents in other UK countries. They were more positive about their teacher's ability to rank and grade students accurately (70.4% agree/strongly agree) and that their teachers knew them well enough to rank and grade them personally (59.2% agree/strongly agree). On the negative side they had similar levels of concern about other aspects of calculated grades as did school students in other UK countries.

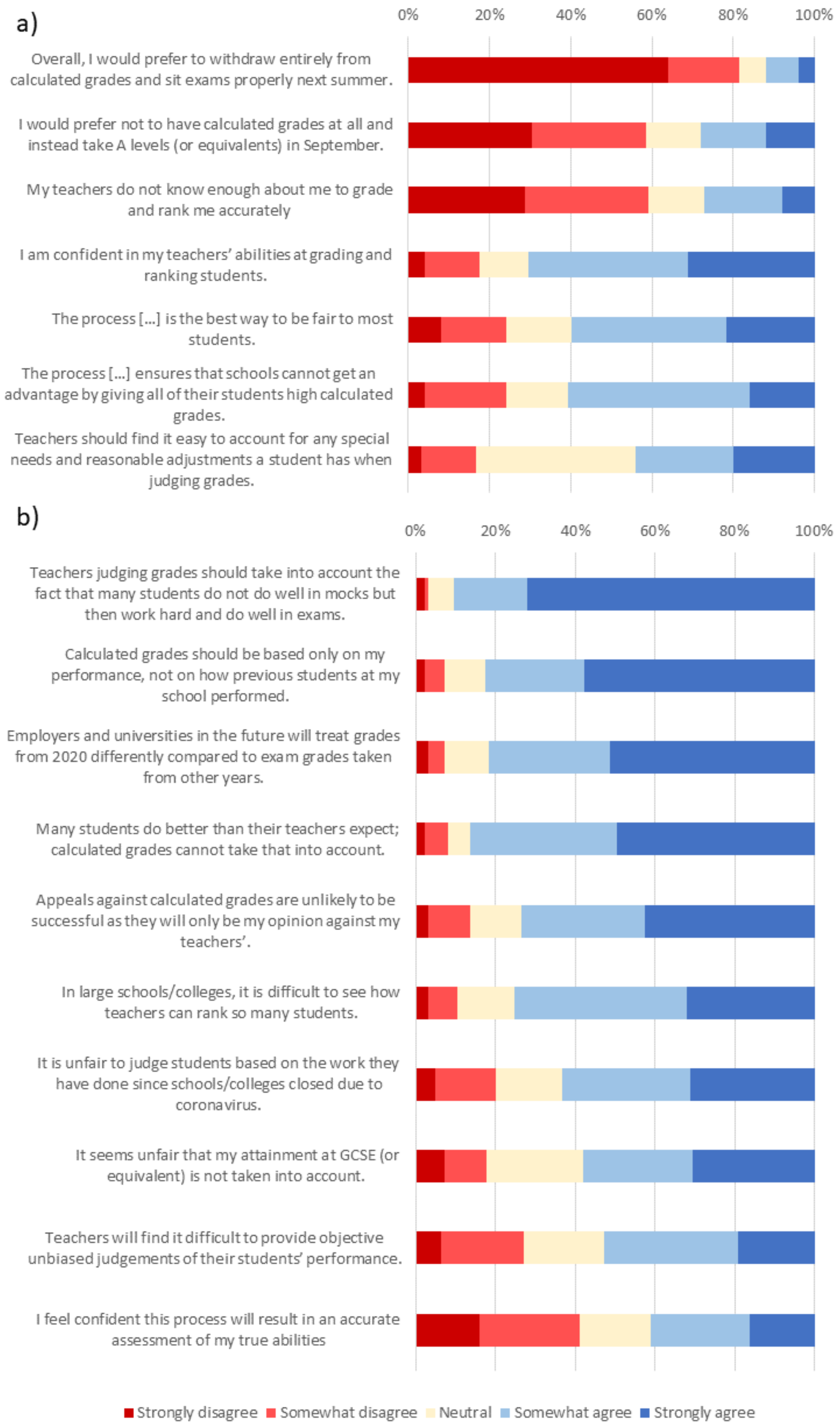

Figure S11: Aspects of calculated grades that respondents were generally more a) positive and b) negative about. Scottish S6 respondents only.

Education since the shutdown

Scottish S6 respondents used on average 2.2 (SD=1.6) educational resources provided by their school, which is fewer than those in the restricted sample. Figure S12 shows Scottish S6 used fewer of all resources compared to the restricted sample, with the exception of summative tests which they were more than twice as likely to use. Scottish S6 students were also more than twice as likely to say their school was assessing them on work since schools closed (n=37; 29.6) with a similar number (n=35; 28.0%) being unsure, and a larger proportion (n=49; 39.2%) saying they were not being assessed.

	Scotland S6	Restricted sample
Online resources	67 (59.8)	781 (71.7)
Paper resources	37 (33.6)	690 (63.9)
Online formative tests	22 (20.0)	447 (41.5)
Pastoral support	32 (29.1)	359 (33.4)
University application support	25 (23.4)	326 (30.4)
Online teaching in real time	31 (27.7)	314 (29.2)
Online summative tests	38 (34.2)	165 (15.4)
Other	<5 (<10)	37 (10.1)

Figure S12: Educational resources provided by schools used in the Scottish and Restricted samples.

Preparation for medical school/university

Scottish applicants were doing similar sorts of preparation as those in the restricted sample; although they were half as likely to be doing examination preparation (n=14; 11.2% vs n=335; 21.4%). Only 19 (15.2%) said they were not doing any preparation which meant numbers were too small to look at reasons for not doing preparation.

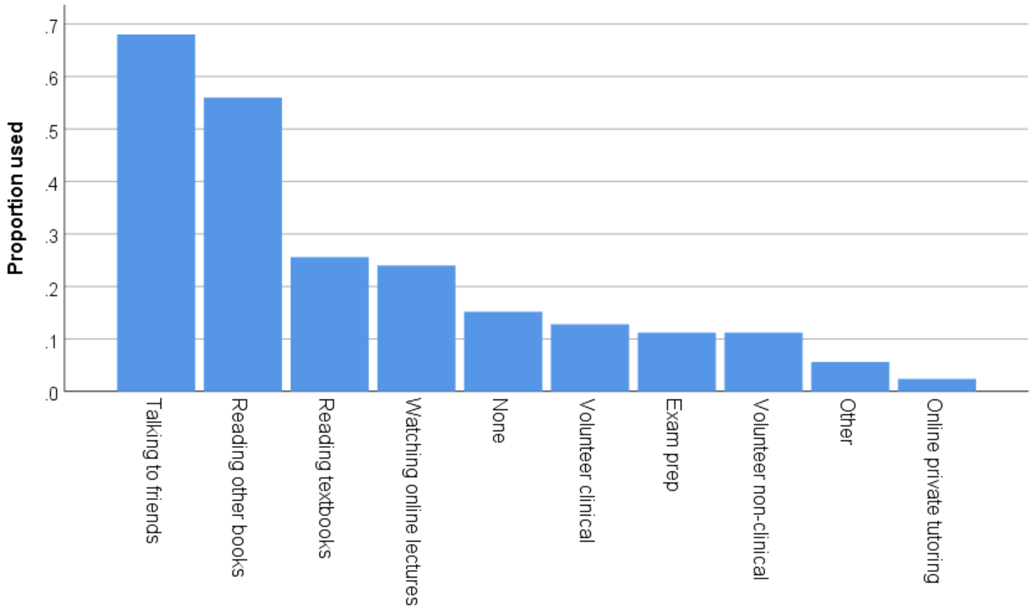

Figure S13: Proportion of respondents undertaking various activities to prepare for medical school or university. Scottish S6 respondents only.

Time spent during the lockdown

The Scottish S6 sample reported similar amounts of time spent on activities as the restricted sample.

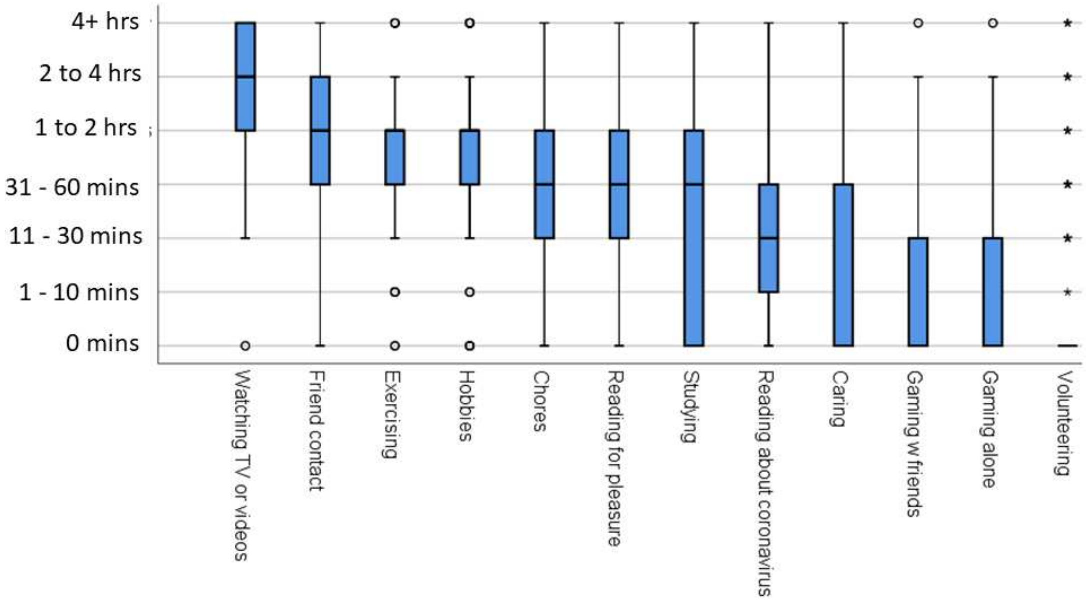

Figure S:14 Amount of time respondents reported spending on various activities during the lockdown. Scottish S6 respondents only.
