# Supplementary material for: The attitudes, perceptions and experiences of medical school applicants following the closure of schools and cancellation of public examinations in 2020 due to the COVID-19 pandemic: a cross-sectional questionnaire study of UK medical applicants
Source: BMJ Open. 2021 Mar 4;11(3):e044753. doi: 10.1136/bmjopen-2020-044753 (PMC7934197; doi:10.1136/bmjopen-2020-044753)
Supplement: Author's manuscript [file bmjopen-2020-044753.draft_revisions.pdf]

BMJ Open is committed to open peer review. As part of this commitment we make the peer review history of every article we publish publicly available.

When an article is published we post the peer reviewers' comments and the authors' responses online. We also post the versions of the paper that were used during peer review. These are the versions that the peer review comments apply to.

The versions of the paper that follow are the versions that were submitted during the peer review process. They are not the versions of record or the final published versions. They should not be cited or distributed as the published version of this manuscript.

BMJ Open is an open access journal and the full, final, typeset and author-corrected version of record of the manuscript is available on our site with no access controls, subscription charges or pay-per-view fees (http://bmjopen.bmj.com).

If you have any questions on BMJ Open's open peer review process please email info.bmjopen@bmj.com

BMJ Open

The attitudes, perceptions and experiences of medical school applicants following the closure of schools and cancellation of public examinations in 2020 due to the COVID-19 pandemic

Journal:	*BMJ Open*
Manuscript ID	bmjopen-2020-044753
Article Type:	Original research
Date Submitted by the Author:	15-Sep-2020

Complete List of Authors:	Woolf, Katherine; University College London Medical School, Research Department for Medical Education<br>Harrison, David; University College London Medical School, Research Department for Medical Education<br>McManus, Chris; University College London Medical School, Research Department of Medical Education

Keywords:	MEDICAL EDUCATION & TRAINING, EDUCATION & TRAINING (see Medical Education & Training), SOCIAL MEDICINE

SCHOLARONE™
Manuscripts

I, the Submitting Author has the right to grant and does grant on behalf of all authors of the Work (as defined in the below author licence), an exclusive licence and/or a non-exclusive licence for contributions from authors who are: i) UK Crown employees; ii) where BMJ has agreed a CC-BY licence shall apply, and/or iii) in accordance with the terms applicable for US Federal Government officers or employees acting as part of their official duties; on a worldwide, perpetual, irrevocable, royalty-free basis to BMJ Publishing Group Ltd ("BMJ") its licensees and where the relevant Journal is co-owned by BMJ to the co-owners of the Journal, to publish the Work in this journal and any other BMJ products and to exploit all rights, as set out in our licence.

The Submitting Author accepts and understands that any supply made under these terms is made by BMJ to the Submitting Author unless you are acting as an employee on behalf of your employer or a postgraduate student of an affiliated institution which is paying any applicable article publishing charge ("APC") for Open Access articles. Where the Submitting Author wishes to make the Work available on an Open Access basis (and intends to pay the relevant APC), the terms of reuse of such Open Access shall be governed by a Creative Commons licence – details of these licences and which Creative Commons licence will apply to this Work are set out in our licence referred to above.

Other than as permitted in any relevant BMJ Author's Self Archiving Policies, I confirm this Work has not been accepted for publication elsewhere, is not being considered for publication elsewhere and does not duplicate material already published. I confirm all authors consent to publication of this Work and authorise the granting of this licence.

For peer review only

The attitudes, perceptions and experiences of medical school applicants following the closure of schools and cancellation of public examinations in 2020 due to the COVID-19 pandemic

Katherine Woolf [a,*]

k.woolf@ucl.ac.uk ORCID 0000- 0003-4915-0715

David Harrison [a]

david.harrison@ucl.ac.uk ORCID 0000- 0002-6639-6752

I C McManus [a]

i.mcmanus@ucl.ac.uk ORCID 0000-0003-3510-4814

[a] Research Department of Medical Education, University College London Medical School, London WC1E 6BT, UK

* Author for correspondence: k.woolf@ucl.ac.uk

For peer review only

Abstract

Objective

Describe the experiences and views of medical applicants from diverse social backgrounds following the closure of schools and universities and the cancellation of public examinations in the United Kingdom (UK) due to COVID-19/coronavirus.

Design

Cross-sectional questionnaire study, part of the longitudinal United Kingdom Medical Applicant Cohort Study (UKMACS).

Setting

UK medical school admissions in 2020.

Participants

2887 participants completed an online questionnaire 8th - 22nd April 2020. Eligible participants had registered to take the University Clinical Admissions Test (UCAT) in 2019 and agreed to be invited to take part, or had completed a previous UKMACS questionnaire, had been seriously considering applying to medicine in the UK for entry in 2020, and were UK residents.

Main outcome measures

Views on calculated grades, views on medical school admissions and teaching in 2020 and 2021, reported experiences of education during the national lockdown.

Results

Respondents were concerned about the calculated grades that replaced A-level examinations: female and Black Asian and Minority Ethnic (BAME) applicants felt teachers would find it difficult to grade and rank students accurately, and applicants from non-selective state schools and living in deprived areas had concerns about the standardisation process. Calculated grades were generally not considered fair enough to use in selection, but were considered fair enough to use in combination with other measures including interview and aptitude test scores. Respondents from non-selective state (public) schools reported less access to educational resources compared to private/selective school pupils, less online teaching in real time, and less time studying during lockdown.

Conclusions

The coronavirus pandemic has and will have significant and long term impacts on the selection, education and performance of our medical workforce. It is important that the views and experiences of applicants from diverse backgrounds are considered in decisions affecting their futures and the future of the profession.

For peer review only

For peer review only - http://bmjopen.bmj.com/site/about/guidelines.xhtml

Article summary: strengths and limitations of this study

- This is the first systematic exploration of medical applicant views on and experiences of the most significant changes to UK education in living memory due to the SARS-COV-2/COVID-19 pandemic.
- It is also the first study we are aware of that looked at university applicant views on calculated grades and the perceived impact on university admissions this year and in 2021.
- The large sample size gathered from around the UK, and the richness of the data provides insight into differences in the experiences and views of different socio-demographic groups, after controlling statistically for educational attainment.
- It is uncertain how representative our sample is of all medical applicants. Medical applicants are not representative of all university applicants in either academic or socio-demographic terms and generalisations from our findings to all applicants should only be done with extreme caution.

For peer review only

Introduction

The UK Medical Applicant Cohort Study (UKMACS) is a study of United Kingdom (UK) medical school admissions. It is primarily a longitudinal questionnaire study of UK residents who in the summer and autumn of 2019 were seriously considering applying to study medicine in the UK for entry in 2020. UKMACS questionnaire data are subsequently linked to administrative data on all UK medical applicants held within the UK Medical Education Database (www.ukmed.ac.uk). Wave 1 data were collected between May and September 2019 and asked how applicants from different backgrounds were choosing which medical schools to apply to. Wave 2 data were collected from November 2019 to January 2020 and asked which medical schools and universities participants had applied to and how they had made their choices.

In March 2020 it was announced that UK schools would close and A-level (and equivalent public examinations) would be cancelled due to the coronavirus/COVID-19 outbreak in the UK. This was one of the most major disruptions ever to affect education and university admissions in the UK and was very significant for the UKMACS cohort, who are mostly in their final year of schooling and were due to sit examinations in the summer of 2020.

We therefore administered an additional unplanned UKMACS questionnaire to understand what medical applicants were experiencing in terms of education, their views on how grades would be awarded following examination cancellations, and their views on how medical schools might respond with regard to admissions policies. We particularly sought to understand how applicants from diverse social backgrounds might differ, with the aim of facilitating the inclusion of applicant perspectives and experiences in discussions about changes to medical school admissions and medical education.(1)

Calculated grades

The absence of A-levels and other equivalent public examinations in March 2020 meant that alternative methods of assessment for candidates had to be found, not least as A-levels are "the single most important bit of information [used in selection]" by universities.(2) On April 3rd *Ofqual* (Office of Qualifications and Examinations Regulation) in England announced that exams under its purview in England would be replaced by calculated grades based on teachers estimation of the grades that their students would have attained, which would then be standardised centrally.(3) The

Scottish Qualification Authority (SQA) and other national bodies also announced similar processes for their examinations.

Performance in A-level examinations has long-term impacts (4, 5), which makes changes to how grades are awarded potentially very significant. The use of calculated grades raises many questions, some of which were summarised in a letter to *The Guardian* by Yasmin Hussein, a GCSE student who said that,

> *"… the … exam hall [is] a level playing field for all abilities, races and genders to get the grades they truly worked hard for and in true anonymity (as the examiners marking don't know you). [… Now we] are being given grades based on mere predictions." Yasmin Hussein, letter to The Guardian, March 29th 2020.(6)*

Among teachers, survey data suggests that there are doubts about the accuracy and fairness of calculated grades, with 39% saying that all students would get a fair deal, 24% saying they would not, and 37% not knowing or not answering. There were also doubts about fairness for students from Black Asian and Minority Ethnic (BAME) backgrounds, about those working hard in the last weeks before an exam being penalised, about teacher 'favouritism', although there were teachers who commented that the process is as fair as possible under the circumstances.(7)

University applicants also have concerns. In a survey carried out by HEPI (Higher Education Policy Institute) before the details of calculated grades were announced, but after it was known that grades would in some way be predicted, 27% thought that their predicted grades were worse than they were likely actually to have attained, compared with 13% thinking their predicted grades were better than they would actually attain.(8)

Another survey of 511 university applicants (including 452 A-level students) conducted for the Sutton Trust found that just under half believed the new A-level grading system would result in their receiving poorer grades but working class respondents were more worried about large negative consequences compared to middle class students. Nearly three quarters believed the new system was less fair than examination grades and this was more of a concern for applicants from *higher* socioeconomic backgrounds. Nearly half of applicants felt the COVID-19 crisis would impede their chances of getting into their first choice university, a more common concern among working class respondents.(9)

The impact on medical school admissions of examination cancellations and their replacement with calculated grades is, at the time of writing, still not completely clear. *Ofqual* states that,

> *"The grades awarded to students will have equal status to the grades awarded in other years and should be treated in this way by universities, colleges and employers. On the results slips and certificates, grades will be reported in the same way as in previous years".(3), p.6.*

The decisions of *Ofqual* in this case are in effect governmental decrees, supported by Ministerial statement, and universities and other bodies will therefore abide by them, as was affirmed by the Medical Schools Council on 5th May 2020.(10) That does not mean however that other factors may not need to be taken into account in some cases, as for instance when applicants do not attain the grades needed for their conditional offers, or for applicants in clearing. Furthermore in guidance updated on 1st May 2020 the Government stated that "if a student does not feel their grade reflects their performance, they will have the opportunity to take an exam in the autumn"(11) with *Ofqual* expanding on 15th May 2020 that "students will be able to use the higher of the two grades for

For peer review only - http://bmjopen.bmj.com/site/about/guidelines.xhtml

future progression."(3) This raises questions for university admissions, as Medical Schools Council acknowledged in their statement of 5th May 2020:

> *"There are a number of issues that the education sector as a whole is yet to resolve. These include how appeals against calculated grades will work across the UK and when students will be able to sit exams if they are unhappy with their calculated grade. The impact of these issues on medical admissions is unclear but medical schools are actively engaging in these discussions and are working hard to develop solutions that are fair to applicants."(10)*

Education during the pandemic

As well as examinations being cancelled, UK schools closed on 20th March 2020 to all except the children of key workers and vulnerable children. While primary schools in England reopened to some year groups on 1st June 2020, this is not the case in other UK countries, and most secondary school and college students will not return full time until September 2020. Similarly in mid-March 2020 many universities suspended face-to-face teaching for the academic year 2019/2020, with much teaching in 2020/21 being online.

The impact of school closures on student learning and outcomes will be significant (12-14) and it may be particularly problematic for those from poorer backgrounds and/or at state-funded schools. The Institute of Fiscal Studies analysed survey data from a weighted sample of over 4000 parents with children aged between four and 15 years old in May 2020 (15). Among secondary school children, those from the richest quintile were spending on average slightly over an hour more per day on learning compared to those in the poorest quintile, amounting to several weeks more learning over the course of the time schools are closed. In particular children in the richest families were spending significantly more on educational activities provided by schools and from private tutors. Even among state school pupils, children from the richest families reported greater access to face-to-face online teaching, which the authors argue is likely to be of higher educational value than other resources that require more parent input, particularly since the poorest parents of secondary school children were less likely to find it easy to support their child's home learning.

The results of the IFS report chime with data from *Teacher Tapp*, an ongoing weighted survey of several thousand teachers in England.(16) At the start of the lockdown (23rd March 2020) private school secondary schools were much more likely than state secondary schools to be using online videoconferencing (27% vs 2%) and online chat (18% vs 3%). The above-mentioned Sutton Trust report (9) also found socioeconomic differences in access to "internet access, devices for learning or a suitable place to study" and differences in the amount of A-level teaching being conducted by teachers at private and state schools.

Among those secondary school pupils who had applied to university, the Sutton Trust report authors argued that students from lower socio-economic backgrounds are also likely face additional disadvantages both with their university applications and when starting university:

> *"Given the uncertainty caused by these changes [to education resulting from COVID-19], university applicants are likely to need more support than ever to navigate the process [of applying to university]. This will be even more important for young people from lower socio-economic backgrounds, who are less likely to be able to draw on the advice of family members with higher education experience themselves. But with schools closed for most pupils, it may be difficult for applicants to get the help they need. Similarly, there's also a danger that this*

For peer review only - http://bmjopen.bmj.com/site/about/guidelines.xhtml

year's applicants will miss out on A level content during the lockdown [...]. For disadvantaged students about to go on to higher education, this could leave them with gaps in their knowledge base, putting them behind their peers before they have even begun at university." [p1. (9)]

The present study

This study aimed to explore and describe perceptions of calculated grades, of student selection more generally, and of educational experiences during school and university closures, in a large group of medical school applicants, who are typically high-attaining students. A range of background factors were assessed to determine how perceptions differed according to demographic and other measures. Data collection took place between April 8th and April 22nd, which was about two and a half weeks after school closures.

Methods

Study design

Cross-sectional questionnaire study, which formed part of the longitudinal UK Medical Applicant Cohort Study.

Eligibility

To be invited to complete the questionnaire, participants had to have registered to take the University Clinical Admissions Test (UCAT) in 2019 and to have agreed to be invited to take part in UKMACS, or they needed to have completed one or more previous UKMACS questionnaires. They also need to have been seriously considering applying to study medicine in the UK for entry in 2020, and be resident in the UK or Islands/Crown Dependencies.

The following groups were excluded from the study and not sent an invite:

- those who had previously requested their data be removed from the UKMACS database;
- previous UKMACS respondents who had not agreed to be contacted for further research;
- previous UKMACS respondents who had previously not consented to having their personal information retained by the research team;
- previous UKMACS respondents who had previously not consented to their personal information being linked with other information for research purposes.

Questionnaire development

During the development of the questionnaire *Ofqual* announced that calculated grades would be awarded. We were therefore able to assess perceptions of how calculated grades would be awarded and used, and perceptions of other possible methods medical schools could use to select or reject offer-holders. We also about potential knock-on effects of calculated grades, including what medical schools should do if they have more applicants meeting their offers than they have medical school places, and how rejected applicants should be treated in the 2021 application cycle.

With uncertainty about whether medical schools and universities would be able to open at the usual time in academic year 2020/2021 we asked applicants whether medical schools should defer opening until teaching could be done face-to-face, or whether they should open online.

For peer review only - http://bmjopen.bmj.com/site/about/guidelines.xhtml

We asked applicants about their use of educational resources provided by schools/colleges/universities, what preparation they were doing for university/medical school, and how much time they were spending on various activities including studying, caring, and volunteering (clinical and non-clinical).

We included self-reported measures of academic attainment and socio-demographic measures we had used in previous UKMACS questionnaires, as well as the 15-item Big Five personality measure used in the national longitudinal cohort study *Understanding Society*. (17)

Most questions were designed specifically for this questionnaire since they asked about unprecedented events and validated items were not available. We constructed the questionnaire with JISC Online Surveys [https://www.onlinesurveys.ac.uk/] and piloted the questionnaire and information sheet with two current applicants to medical school. Amendments were made in response to feedback from the applicants and from Medical Schools Council.

Questionnaire administration

Participants were sent an email invitation and link to the current questionnaire on the afternoon of 8th April 2020. 18,665 invitations were sent, with up to two email reminders and two text message reminders. The questionnaire closing date was 20th April 2020, with responses accepted up to 22nd April 2020.

The questionnaire was administered on the JISC Online Surveys platform. All participants were given the option to withdraw from the study and to request that their contact data be removed from the participant list. Any participant who had responded but then wished for their responses to be removed from the study were able to do this by contacting the UKMACS Research Team by the end of 22nd April 2020. No participants requested that their questionnaire responses be removed.

Statistical analysis

Descriptive and univariate analyses were performed in SPSS v26. Multivariate analyses were performed in R.

Missing values were imputed using the *mice* package.(18) Following the general advice of van Buuren (19) missing values were calculated using *pmm* (predictive mean matching), which as van Buuren says, is a good " all-round method with exceptional properties" (p.84). *pmm* is the default method in the *mice()* function for all scale types (binary, ordinal, numeric) and has the advantage that imputed values are always taken from the existing range of actual values in the data, with *pmm* being robust against mis-specification. The number for the pool of candidate donors, *d*, was set at 5, the default in *mice()*, and the number of imputations, *m*, was set at 25.

Regression analyses on the 25 *mira* datasets were carried out using the *lm()* function within the *with()* function, and separate sets of results in the *mipo* dataset were combined with the *pool()* function. Regression analyses entered all socio-demographic and educational predictor variables into the analysis simultaneously, and results are only reported which were significant with p<.01 after taking all other variables into account, so the analysis is relatively conservative. The nine socio-demographic and educational variables used were: ethnicity, gender, school type, parental higher education, IMD quintile, mean GCSE points, mean top three predicted A-levels, UCAT score, number of medical school offers.

Factor analysis on the 87 attitudinal variables was carried out using the *psych* package (20) with *fa.parallel()* and *nfactors(),* being used to determine the number of factors.

For peer review only - http://bmjopen.bmj.com/site/about/guidelines.xhtml

Freetext question answers

All answers to freetext questions were read by the research team, and illustrative quotes selected to aid understanding of quantitative results.

Results

Participants

3071 participants completed the questionnaire, of whom 2904 stated they were eligible to take part (i.e. seriously considering applying to study medicine in the UK in 2020 and resident in the UK or Islands/Crown Dependencies). After removing 16 respondents who did not consent to have their data analysed and 11 duplicates, there were 2877 valid cases for analysis, which is 15% of those invited. This is subsequently referred to as the full sample.

The main analyses were performed on a restricted sample of 1562 respondents currently in Year 13, who had applied to medicine for entry in 2020, with at least three predicted A-levels and no achieved A-levels. Results are also reported in the Supplementary files for respondent groups excluded from the restricted sample, notably those living in Scotland and those not currently in Year 13 – see Supplementary file.

Missing data

The analysis considered 120 measures in the restricted sample, divided into:

1. *Questionnaire items.* The questionnaire asked about attitudes to 87 different topics concerning medical school entrance. Of 153,076 data points, 10788 (7.2%) were missing. For the individual variables, the median percentage of missing data values was 0.48%, with 75 measures having fewer than 5% of missing values.

2. *Demographic and educational items*. For 12 demographic measures, 462 of 18744 measures were missing (2.5%), with a median of 1.0% per measure, and 11 measures having fewer than 5% missing values. Ethnic origin was not asked about in the present study. The ethnicity of 889 respondents who had reported it in a previous questionnaire were imported into the present dataset; 43.1% of ethnicity measures were therefore missing. IMD_Quintile was obtained from postcodes in England, Wales and Scotland, and was missing in 14.8% of cases.

There were four educational attainment items (grades in the highest-scoring 'top' three predicted A-level grades, UCAT score, BMAT score, and mean GCSE grade). Top three predicted A-level grades were present for all because the sample was based on that criterion. Of the remaining three measures, 1852 out of 4686 (39.5%) were missing: UCAT scores were missing in 13.6% of cases, and BMAT scores in 61.3% of cases, but in both cases missing values were mostly structurally missing, candidates mostly having taken only one aptitude test or the other. Mean GCSE grade was missing in 43.1% of cases, having been imported from a previous UKMACS questionnaire.

Participants self-reported their current or most recent school in the current questionnaire. This question was also present in the Wave 1 UKMACS questionnaire. For schools in England, publicly-available administrative data were available on school type (e.g. independent, voluntary aided) and for state-funded schools there were data on whether the admissions policy was selective or non-selective. These were combined to create a binary variable of School Type (non-selective state schools vs private/selective schools) for 1132 respondents (27.1% missing). A composite variable

For peer review only - http://bmjopen.bmj.com/site/about/guidelines.xhtml

was created using present responses and the responses in the Wave 1 questionnaire, so data were available for 1158 respondents with values missing in 25.9% of respondents.

Demographics

Demographics for the full and restricted samples are reported in **Table 1**.

Education and achievement

Predicted A-levels

A-level grades were scored as A*=12, A=10, B=8 etc, and those reported as being between two grades as intermediate e.g. A*/A =11, A/B = 9, etc. Mean predicted A-level grades were calculated for the top three grades regardless of subject (*Mean top three predicted* A-levels), and for all grades (*Mean predicted* A-levels). *Mean top three predicted* A-levels was 10.89 and *Mean predicted* A-levels was 10.71, both of which are over an A grade.

UCAT, BMAT, GAMSAT

1546 participants (99.1%) reported having taken UCAT; 765 (49.0%) reported having taken BMAT; and none reported having taken GAMSAT. Of the 1350 participants who reported a total UCAT score that was greater than 1799 and less than 3601, the mean score was 2660 (SD=235).

GCSE

GCSE grades can range from 1 to 9. A variable *Mean GCSE* was calculated by dividing the total GCSE points by the number of GCSEs taken, and the mean was 7.91 (SD=0.71).

Relationships between educational measures

UCAT score correlated with *Mean top three predicted* A-levels at 0.418 (p<.001) and with *Mean GCSE* at 0.487 (p<.001). *Mean GCSE* and *Mean top three predicted* A-levels correlated at 0.611 (p<.001).

Participants at non-selective state schools had lower scores on all attainment measures (*Mean GCSE*: difference=0.3 points, p<.001; *Mean top three predicted* A-levels: difference=0.23 points, p<.001; UCAT score: difference=89 points, p<.001).

Medical school offers

1292 (85%) respondents had applied to four medical courses, 1289 (82.5%) had at least one offer, 177 (11.3%) had four offers, and 204 (13%) were waiting to hear from at least one medical school at the time of completing the questionnaire.

Respondents who did not have a parent/carer with a university degree were less likely to have a medical offer (78.1% vs 85.0%; p=0.001).

Applicant views on admissions

Perceptions of the fairness of methods medical schools could consider using in the selection of offer-holders

Participants were asked to rate the fairness of 17 measures, including calculated grades, that medical schools could potentially use to decide to accept or reject offer-holder following exam cancellations. Rating categories were: "Unfair: should not be used" "Quite unfair: avoid if possible" "Quite fair: could be used in combination with other measures" "Very fair: could be used alone", with a freetext question asking for additional comments and suggestion.

For peer review only - http://bmjopen.bmj.com/site/about/guidelines.xhtml

No measure was felt by a majority of participants to be fair enough to use on its own. The measure considered most fair was *Exam grades taken in September 2020 (if these take place)* (32.3% very fair), followed by *Predicted Grades declared on UCAS application* (26.2% very fair), *Calculated grades* (22.6% very fair), *GCSE grades* (20.4% very fair) and *Score at interview* (19.5% very fair).[1]

Several methods were felt by a majority to be fair enough in combination, particularly *Predicted grades* (80.6%), *GCSE grades* (73.8%), and *Score at interview* (73.4%); but only a fifth (20.3%) of participants felt *Attendance at widening participation activities* was quite fair or very fair. See Figure 1.

Multiple regression results showed that after taking account of all other educational and socio-demographic variables, BAME participants were more likely to perceive *Exams taken in September 2020*, *UCAS personal statement*, and *Personal background* as fair to use, and respondents from deprived areas were more likely to perceive *Personal background* and *Attendance at widening access programmes* as fair to use. *Calculated grades based on mock exams, coursework etc, and awarded in place of final examination grades* were perceived as less fair by those with lower predicted A-levels.

There were 154 freetext responses (10%), with participants elaborating on their responses or suggesting alternatives:

> *"A combination of the most objective information that every offer holder will have, ie GCSEs, UCAT or BMAT, interview score, etc"*

> *"A standardised form of assessing all medical applicants would be the best way to allocate existing places. One could argue that most of us have already taken a standardised assessment, the UCAT. Since we do not have standardised A level grades, places should be offered using the UCAT as this is the fairest way of distributing places to the most able students."*

> *"Using interview scores and UCAT scores in combination are independent measures, and are more fair than using calculated grades which have the potential to be biased."*

> *"Anything including personal statement, BMAT or UCAT I would argue are unfair to use as judgement as there will definitely be a bias in terms of how certain students achieved their grade. I believe the fairest way to determine ones overall grade would be to use their GCSE data with a combination of evidence throughout the two years of A levels."*

Other measures participants mentioned included: an additional university assessment (written, viva or project/portfolio-based) now or at the start of the academic year, an additional interview, selection at the end of Year 1/make first year a foundation year, additional reference from teachers/school, reference from work experience, school/college attendance record, distance from university, extenuating circumstances, self-reported use of time during quarantine/lockdown, number of offers received, prioritise those with higher degrees, prioritise those already working in the NHS, extra-curricular achievement (e.g. music, Duke of Edinburgh's Award), school's prior achievement. For example:

[1] See Figure 1 for full item wording

For peer review only - http://bmjopen.bmj.com/site/about/guidelines.xhtml

"NHS experience ie patient facing health professional ie years and grade, other non technical skills, education background ie. science, post graduate achievement ie MSc particularly if in science or medical subject and grade achieved. Also emphasis on the candidates as a whole ie well rounded personality (potential to communicate well) rather than typical A Grade student. Letter of recommendations from medical consultant whom candidates may have worked closely with."

"Another interview possibly over the phone to see what students have done with their time in quarantine (ie, volunteering in a care setting or hospital / working in a hospital / exploring other interests)"

"Each university could form their own selection test similar to UCAT/BMAT with a brief guidance/specification on what will be on the test given out to offer holders so they have some time to revise for it, but this should be used in combination with other details (e.g. if offer holder's calculated grade was only 1 grade below what was required for entry)"

"I think a combination of previous results, any exams that do go ahead (at some point whether that is this summer or later), alongside medical applications, relevant work experience (as per personal statement and any other forms detailing this) and the applicant interview. Also potentially the medical schools could generate online admissions tests for students with conditional offers to generate a clearer view of a students capability and ability to comprehend and withstand the pressures of medical school. But any tests generated by the medical schools must be used alongside the other parts of the applications to ensure fairness."

Participants were asked whether they had heard anything from medical schools/universities they had applied to about how selection might be impacted by examination cancellations; among those holding conditional offers, a minority (n=538; 42%) said they heard from at least one medical school/university they had applied to.

Acceptability of options for dealing with a situation in which more students meet their offers than there are medical school places

Participants were asked to rate the acceptability ("completely unacceptable", "slightly unacceptable", "neutral", "slightly acceptable", "completely acceptable") of a number of options that medical schools could use if they had more students meeting offers than they had places, with a freetext question asking for additional comments and suggestions.

The most acceptable option was *Ask some applicants with offers to volunteer to defer a year*. The only other acceptable option was *Accept all applicants whose calculated grades meet the conditional offer, although it could mean fewer resources per student*. See **Figure 2**.

Multiple regression analyses showed no significant differences by social or demographic group on these items.

There were 187 freetext responses (12%). Several respondents suggested that medical schools should receive more funding to manage larger cohorts and create more doctors, e.g.:

For peer review only - http://bmjopen.bmj.com/site/about/guidelines.xhtml

> *"Deferring of one year should not be taken into consideration as this would damage applications of next year. Ask the government to invest more money on the NHS and allow to have more spaces. All these problems could be solved if exams were taken virtually."*

> *"The government could also provide more funding for medical schools- not only will this allow more people to attend but it will also mean there are more doctors down the line who can work in the NHS."*

There were suggestions that applicants could opt to attend other medical schools they had applied to but which they had not selected as their firm or insurance choice, or that they could be offered places at medical schools they had not applied to:

> *"If some medical schools have a lower numbers of applicants overall, compared to others, redistribute some students to these ones, with permission."*

There were many suggestions of incentives to defer, and some felt that they would welcome a year off before starting:

> *"Incentives to defer like 1 yr free accommodation or £5000 or student ambassador job for gap year"*

> *"Incentive to deferring such as free university accommodation for the first year, organised work experience placements and or organised care assistant jobs for the gap year."*

> *"If people are asked to volunteer to or forcefully defer entry, offering alternatives for work they could do within a healthcare setting for that year. For example, maybe clerical work within the NHS so they're still immersed within the healthcare system.*

> *"Asking students to voluntarily defer a year would be a popular option, I think many people will reevaluate their priorities over the coming months and may appreciate the opportunity."*

> *"The option to defer is definitely an option that should be considered as many people would be happy with the idea of gaining more medical experience in the year out that they would now have."*

There were suggestions medical schools could have multiple cohorts either all starting in October or one cohort starting in October and another cohort starting early 2021.

> *"Create an extra group/year for Covid Students to manage the numbers"*

> *"Maybe consider having staggered starts throughout the year October start January start June starts."*

> *"Stagger the course to offer two presentations and alter the following academic term holidays if possible"*

Respondents also expressed concern as to the impact of the present disruption on next year's admissions cycle and available resources:

For peer review only - http://bmjopen.bmj.com/site/about/guidelines.xhtml

"The selection process should not be biased towards those rejected this year, next year, and should not change for the next cohort."

"I hope that this year's or next year's applicants will not be disadvantaged due to these unprecedented circumstances."

Perceptions of potential impact on admissions for 2021

Participants were asked to rate how much they agreed or disagreed with six options as to how medical schools could deal with the potential impact of the current situation on admissions in 2021. See **Figure 3**.

In general, respondents felt medical schools should give special consideration to current applicants reapplying next year (67.1% agreed/strongly agreed that *Applicants rejected this year should be given special consideration when re-applying next year*) however opinions were divided about what that special consideration should consist of.

Multiple regression analyses showed that after accounting for number of offers, educational, social and demographic factors, BAME respondents were more likely to feel that re-applicants should be given some advantages.

Starting academic year 2020/2021

A majority of respondents (n=952, 61.1%) believed that if necessary, medical schools should *Defer the start of the academic year only when face-to-face teaching is possible* with 605 respondents (38.9%) believing that medical schools should *Start the academic year on time using distance learning for as long as is necessary*. This did not vary significantly by prior attainment, number of offers, or educational/social/demographic background.

Education and university preparation

Calculated grades and the perceptions of process of awarding calculated grades in lieu of examination grades

Participants were generally fairly ambivalent towards calculated grades. On the positive side (see **Figure 4 Error! Reference source not found.a**), the majority of respondents (78.6%) preferred calculated grades to taking examinations next year, and about half (54.9%) preferred calculated grades to taking examinations in September 2020. Over half (59.3%) agreed that schools wouldn't be able game the process to award all their students high grades, and 51.4% felt that the process of awarding calculated grades was the best way to be fair to most students in the circumstances (although 35.0% disagreed). Over half (56.4%) agreed that their teachers were generally able to rank and grade students accurately, however respondents were divided as to whether their own teachers knew them well enough to grade and rank them accurately: 42.0% agreed their teachers did NOT know them well enough whereas 44.6% thought their teachers DID know them well enough.

On the negative side (see **Figure 5 b**), over half of respondents (52.9%) disagreed or strongly disagreed that calculated grades would result in an accurate assessment of their abilities, with 63.4% agreeing that teachers would find it hard to be unbiased, 80.7% agreeing it was difficult to see how teachers in large schools can rank so many students and 85.5% agreeing calculated grades cannot take into account students doing better in exams than their teachers expected. Most agreed it was unfair to judge students on work done since schools/colleges closed (70.4%), that grades should be

For peer review only - http://bmjopen.bmj.com/site/about/guidelines.xhtml

based solely on their performance and not the performance of previous students at their school (69.6%), and that it was unfair their GCSE performance was not taken into account (68.7%).

Mean top three predicted A-level points was a major predictor of perceptions of calculated grades but there were also differences by background after accounting for prior attainment, number of offers and other educational/social/demographic factors: BAME respondents and female respondents were more negative about calculated grades and respondents from non-selective state schools and those from more deprived areas were more likely to agree that calculated grades should not take into account the performance of previous pupils at their school. See **Table 2**.

There were 398 freetext responses to the following request for further comments at the end of the questionnaire: “Please use this space for any additional comments you wish to make about the questionnaire or selection of medical students”. These responses included concerns that calculated grades would be based on work completed early in the academic year and on mock exams created and assessed by the school. It was felt that these measures would not take into consideration the development and academic progress made by pupils over the year, even when teachers gave special consideration to the impact of the disruption. There was also concern that at the time of mock exams in particular, many medicine applicants were more focused on admissions tests (BMAT in particular), submitting applications and preparing for interviews.

> *“Grade calculations took away the chance the students had to prove themselves (final exams) and their control. Basing the final grade on a time when the students weren't aware that they were being truly assessed can hardly be classed as fair.”*

> *“I believe universities should be lenient and realise that if a students calculated grade is below their conditional offer, this is not 100% representative of the students abilities. If they were able to secure an offer in the first place then universities should already know the academic capabilities of said student through their GCSE grades, predicted grades, UCAT/BMAT scores, teacher references, interviews etc. Otherwise, they wouldn't have given the student an offer. Where possible, every offer holder should be given their place at university in this academic year, whenever it resumes and should not be forced to take a year out and spend that year being stressed, lost and demotivated.”*

With teacher submitted grades then being subject to standardisation by the exam boards based on previous achievement from a school was a concern for this student:

> *“I am the only student in my year and the third student in my sixth form's history to ever apply for medicine, and the first to receive all 5 offers. My school historically is one that does not do very well and I fear that my individual success and all the hard work I have had to do on my own as I get no help from my school, will be overshadowed by the bad results from previous years.”*

Education since the shutdown

A minority of respondents said their school was planning on formally assessing them on work done since the shutdown (n=184; 11.8%); nearly half (n=740; 47.5%) said their school would not, and over a third (n=614; 39.4%) were uncertain. Respondents attending a private/selective school were twice as likely to report being assessed on work since the shutdown (14.2% vs 7.6%; p<.001).

For peer review only - http://bmjopen.bmj.com/site/about/guidelines.xhtml

Participants were asked whether they were using educational resources provided by their school/college and if not why not. Nearly all respondents had used at least one resource (n=1346; 91%) and three was the average number used.

Respondents attending private/selective schools were more likely to report having used all educational resources except support for university applications, and those at non-selective state schools used on average two resources compared to the three used by those at private/selective schools. The largest difference was in the use of online teaching in real time, which those at private/selective schools were nearly four times more likely to have used. See **Table 3**.

.

In the multivariate analyses, attendance at a private/selective school was an independent predictor of using online teaching in real time, online resources for home learning, online formative assessments, and paper resources for home learning, even after controlling for prior attainment and socio-demographics. In addition, having at least one parent/carer with a university degree was an independent predictor of using paper resources for home learning, and having lower UCAT/BMAT scores was an independent predictor of using online teaching in real time.

Those who had not used educational resources reported the main reason(s) were either that the resources were not available or that they felt they did not need to use them. Only very few said they had not used a resource because of a lack of private quiet space, lack of time, lack of internet/computer access, or because they were finding it too hard to focus. Those at non-selective state schools were more likely than those at private/selective schools to state lack of availability as a reason, and less likely to state not needing to as a reason– see **Table 4**.

Preparation for medical school/university

Participants were asked what preparation if any they were doing for university or medical school – see **Figure 5**.

Of the 207 (13.3% of the sample) who said they were not doing any preparation, the most common reason was that they were too worried and couldn't focus (n=88; 42.5% of those not doing any preparation), not having resources (35.5%), feeling it wasn't necessary (29.5%), caring for others (13.5%), not going to university this year (14.0%), not having time (6.3%), and being unwell (4.8%). Respondents could select multiple reasons.

Time spent during the lockdown

Participants were asked to state how much time they were spending on various activities in the previous five days – see **Figure 6**. The multivariate analysis showed that respondents from private/selective schools reported spending more time studying, even after controlling for prior attainment and socio-demographic factors.

Personality measures and time spent during lockdown

Personality traits are "relatively enduring styles of thinking, feeling, and acting".(21) It is generally agreed that there are five distinct personal traits or factors: Neuroticism, Extraversion, Openness to Experience, Agreeableness, and Conscientiousness. Correlations between personality and time spent on activities are shown in **Table 5**.

For peer review only - http://bmjopen.bmj.com/site/about/guidelines.xhtml

Factor analysis

Number of factors

The factor analysis included 87 variables which are attitudinal or related to attitudes. The maximum eigenvalue was 6.99, with 27 eigenvalues greater than 1. A scree-plot suggested that there was a break at or around 6 factors (see **Figure 7**). Other criteria were very variable, with *fa.parallel()* in the *psych* package in *R* suggesting there were 19 principal components with eigenvalues greater than the 95% upper confidence interval for randomly generated data. *nfactors()* in *psych* said that VSS complexity 1 suggested 17 factors, VSS complexity 2 suggested 17 factors, Velicer's MAP gve 10 factors, Empirical BIC gave 20 factors, and Sample Size Adjusted BIC gave 20 factors. However the output also reported, "Although the vss.max shows 17 factors, it is probably more reasonable to think about 4 factors". Overall there are probably many small factors corresponding to measures with low communalities and hence mostly unique variance. For present purposes we are particularly interested in aggregating measures to gain more statistical power, and therefore we chose to extract 6 principal factors, which corresponds with the break in the scree slope, and is a little larger than the *nfactors*() recommendation of 4.

Naming of factors

The six factors were named as following, by considering the highest absolute loadings, along with all loadings over 0.35:

1 '*Lack of confidence in calculated grades*'. Positive loadings reflected concerns that teachers will not know students well enough and will find it hard to be objective, preferring not to have calculated grades and take exams in September or next summer, and appeals being unlikely to be successful. Negative loadings reported confidence in the process resulting in an accurate reflection of a student's true ability, and the awarding process being fair to most students. High positive scores therefore represent **a lack of confidence in the process of determining calculated grades**.

2 '*Special treatment next year for rejected applicants*'. High positive loadings were associated with medical schools needing to give higher priority and special consideration next year to students rejected this year, with rejected candidates being automatically given conditional offers next year. Negative loadings suggested that re-applicants next year should be treated in the usual way, and special treatment for rejected applicants this year would not be fair for first year applicants next year. High positive scores therefore suggest that **applicants who are rejected this year should be treated specially next year**.

3 '*Other selection measures to be taken into account*'. A small group of items suggested that selection could take into account aptitude tests such as UCAT, BMAT, and performance at interviews. High scores therefore suggest that where possible, **measures other than calculated grades should be taken into account**.

4 '*Preparing for medical school'*. High positive loadings on this factor reflected applicants who during lockdown were preparing for university by reading (either textbooks or other books), were watching online lectures, as well as talking with friends. Negative loadings reflected applicants who were not doing any preparation, didn't feel preparation was necessary, didn't have any resources, or who couldn't focus because they were too worried. High scores therefore indicate an applicant's **concentration on preparing for medical school or university**.

'*Importance of background and experience*'. All high loadings were positive and indicated that medical schools should take into account work experience, the applicant's personal statement, and

For peer review only - http://bmjopen.bmj.com/site/about/guidelines.xhtml

the teacher's reference on the UCAS form, attendance at university summer schools and widening participation programmes, an applicant's personal background such as being from under-represented groups, and other grades in qualifications such as GCSEs and the Extended Project Qualifications. Overall higher scores indicate that **a wider range of measures should be used to take into account personal background and wider experience.**

'*Resources from school for home study*'. All loadings were positive, and indicated that applicants were being provided with live online teaching, online resources for home learning, paper resources such as workbooks, formative online assessments, and summative online assessments that might count towards calculated grades, doing timed essays or past papers, and spending more time studying. Higher scores therefore indicate **greater support for home schooling from schools and colleges**.

Predictors of factor scores

Predictors of factor scores were assessed using multiple regression. All predictor variables in the set were entered and only those achieving p<.01 are reported. All predictors therefore take into account the effects of others in the set. Set A is the basic set used earlier in the study. Set B is extended by including socioeconomic group (based on parents' jobs), doctor parent(s) and the five Big Five personality factors, and are included on an exploratory basis. See **Table 6.**

Summary and conclusions

Summary of results

No single measure, including calculated grades, was considered fair enough by most applicants to use in the acceptance or rejection of offer-holders; however many applicants considered calculated grades – and many other measures - fair enough to use in combination with other measures such as interview scores or admission test scores. Taking into account personal background or widening participation attendance was considered fairer by BAME applicants, those from deprived areas, and those without degree-educated parents.

Many respondents had concerns about calculated grades, especially BAME and female applicants who felt teachers would find it difficult to grade and rank students accurately, and those from non-selective state schools and living in deprived areas were more concerned about the standardisation process that uses the attainment of previous pupils at a school. Despite this, the majority would rather have calculated grades than forgo calculated grades completely and take examinations in Autumn 2020 or Summer 2021 instead.

Respondents mostly felt that medical schools should admit any applicant who met their conditional offer, even if that meant having to increase the number of places (which would require a legal change and increased government funding), although there was also acceptance of medical schools asking for volunteers to defer but not of requiring deferrals. Respondents were divided as to how rejected applicants should be treated if they were to reapply next year, with some respondents feeling they should be treated no differently and others feeling their 2020 experience should be taken into account. A majority of respondents tended to favour medical schools delaying the start of term until face-to-face teaching were possible.

Applicants from non-selective state schools reported using fewer educational resources than their counterparts at private or selective schools, and in particular they reporting less online teaching in real time, and spending less time studying during the lockdown.

For peer review only - http://bmjopen.bmj.com/site/about/guidelines.xhtml

Comparisons with other research

Our findings show many similarities to other recent UK studies of the effects of the COVID-19 pandemic on education in the UK (8, 9, 15, 16) however it is notable that in this sample of medical applicants ethnicity is more significant than socioeconomic factors in predicting concerns about calculated grades – indeed, to our knowledge, ours is the only survey of applicants that includes a measure of ethnicity. It is known that predicted grades are lower for some minority ethnic groups (22) and indeed, on 2nd April 2020 after the announcement of the cancellation of examinations but before *Ofqual* specified details of calculated grades, the Runnymede Trust and several other race equality organisations wrote to the Secretary of State for Education to urge him to "ensure a fair, transparent and robust system which will more accurately reflect the ability and attainment of students from different backgrounds".(23) Subsequently, on 30th April, the Equality and Human Rights Commission said that,

> *"Using predicted grades in place of this year's summer assessments could deepen the existing inequality in education and put the future of disadvantaged young people at risk if not correctly implemented"* (24)

Our finding that students from private/selective schools were using more educational measures - especially online teaching in real time, which requires significant teacher input and which Andrew et al (15) argue is higher quality that other types of resource - reflects findings from those authors' research with parents of secondary school children (15) and teachers (16); however in our sample students' use of educational resources and time spent studying did not vary by socioeconomic background, including parental higher education, socioeconomic status, or area deprivation. This may be a feature of this particularly high-achieving sample of medical applicants.

Strengths and limitations

This study is, to our knowledge, the first systematic exploration of medical applicant views on and experiences of the most significant changes to UK education in living memory. It is also the first study we are aware of that looked at university applicant views on calculated grades and the impact on university admissions. The large sample size gathered from around the UK, and the richness of the data allowed us to examine important differences in the experiences and views of different socio-demographic groups, after controlling statistically for educational attainment.

The speed at which we were required to develop the questionnaire and the unprecedented nature of the topic under investigation meant we were unable to use validated measures for most questions, nor have we been able to validate the measures ourselves, although we were able to pilot them with current applicants.

It is uncertain how representative our sample is of all medical applicants. Data on applications, offers, acceptances and academic achievement from the current UCAS cycle are not released until early 2021, but it is very likely that offer-holders were over-represented in our sample. Data from the 2019 UCAT testing cycle also show that our sample scored higher than the mean [https://www.ucat.ac.uk/media/1329/2019-test-statistics-oct-2019.pdf]; however not all UCAT test-takers apply to medicine. Demographic data on 2020 medical applicants released by UCAS in November 2019 showed that our restricted sample was similar to all English applicants aged 17 to 19 in terms of ethnicity and deprivation but had more women [https://www.ucas.com/data-and-analysis/undergraduate-statistics-and-reports/ucas-undergraduate-releases/applicant-releases-2020/2020-cycle-applicant-figures-15-october-deadline].

For peer review only - http://bmjopen.bmj.com/site/about/guidelines.xhtml

Medical applicants are not representative of all university applicants in either academic or socio-demographic terms; however the similarity of some of our findings to that of other research, for example that private school pupils are receiving significantly more education than non-selective state school pupils, suggests that the views and experiences of our sample may not be completely different from those of university applicants more generally; however generalisations from our findings to all applicants should only be done with caution.

Implications for policy and practice

The impact of calculated grades on medical admissions was, at the time of writing, uncertain. Our questionnaire closed on 22nd April and on 5th May 2020 the Medical Schools Council announced that medical schools would honour all offers met (something not clear at the time of our questionnaire), while acknowledging that there were still a number of issues that needed resolving.

How calculated grades are likely to work in practice has also been explored by a parallel analysis by our team using UKMED data over the last ten years, comparing predicted A-level grades (which are likely to be similar to calculated grades) with actual, attained A-level grades.(25) Predicted grades were systematically higher in medical school applicants than eventual achieved grades. In addition the predictive validity of predicted grades was only about two-thirds that of achieved A-level grades, both for outcomes five or six years later at the end of medical school, and seven or eight years later in postgraduate examinations. The under-prediction by predicted grades was mitigated in part, although not entirely, by combining predicted grades with UCAT/BMAT scores, which supports the views of some applicants that other measures might be used for selection amongst applicants not meeting the terms of conditional offers.

The likely impacts on medical schools of using calculated grades were at the time of writing uncertain, but our estimates suggested there could in effect be a lowering of entry grade requirements, with possible subsequent increases in medical school drop-out rates, and a somewhat academically weaker cohort with poorer performance in medical school and postgraduate examinations.(5, 26) That is potentially important since very poor postgraduate examination performance itself strongly predicts being sanctioned by the medical regulator.(27)

In the awarding of calculated grades, we predicted that the raw ‘centre assessment grades’ and rankings produced by teachers for *Ofqual* were likely to be similar to predicted grades in being more generous than achieved A-level grades would have been, although the standardisation to be used by examination boards and *Ofqual* are likely to minimise that effect, so that distributions of calculated grades within subjects and centres become similar to actual A-level grades in previous years. As it transpired the centre assessment grades ended up being used without adjustment, and these were significantly higher than previous years’ A-level grades, with the Education Datalab stating “At grades A*-A, there was an increase from 25.2% to 38.1%” (see https://ffteducationdatalab.org.uk/2020/08/gcse-and-a-level-results-2020-how-grades-have-changed-in-every-subject/).

As a result of the awarding of calculated grades an excess of candidates met their conditional offers. Giving their views on what should happen in this regard, applicants in our study suggested that that in light of the shortage of doctors,(28) medical schools might argue for increased places and funding. In the event the Government did indeed lift the cap on medical school places to accommodate the increase in students (see https://www.gov.uk/government/news/action-agreed-to-support-students-into-preferred-universities). The impact of large increases in number on teaching and on predicting through to numbers of places for clinical teaching, foundation training and so on is still

For peer review only - http://bmjopen.bmj.com/site/about/guidelines.xhtml

uncertain. It is worth considering that cohort sizes at many medical schools are already very large, that students tend to be less satisfied at larger schools,(29) and that accommodating extra students into face-to-face teaching that is COVID-secure is likely to be extremely challenging. On the other hand, there is a clear need for more doctors and it is likely that the change to admissions will result in a more socially and demographically diverse cohort.

In this questionnaire many applicants felt it could be fair to using other information such as interview score, UCAT score, or GCSE score to accept or reject offer-holders, and this could include in selecting from amongst 'near-misses'. Overall respondents to our questionnaire demonstrate a lack of confidence in the process of calculated grades. Given the concerns of the Equality and Humans Rights Commission, and the clear concerns also expressed in our study by some disadvantaged groups, there is a clear need to ensure that entrants as far as possible continue to reflect the breadth of those applying to study medicine.

The cancellation of public examinations and the use of calculated grades are not the only problems facing the 2020 application cohort. They are also at risk, particularly those from non-selective state schools, of coming to medical school having had less education over the previous few months,(14) meaning medical schools may need to provide additional teaching and resources to help students catch up. This is likely to be especially challenging for medical schools given the huge constraints on university budgets arising from drops in student numbers(30) and given that many are likely to be unable to open for face-to-face teaching at the start of the academic year, which in itself has unknown consequences.

The 2020 cohort of entrants is likely to face more uncertainty than any cohort of medical student entrants in the past half-century, and our survey makes very visible the many concerns of those applicants.

Conclusions

The global tragedy of the coronavirus pandemic, in addition to its extensive mortality and morbidity, has resulted in huge and sudden disruptions to established ways of life including education and training at all levels. Medical education and training is no exception. The coronavirus pandemic will have significant and long term impacts on the selection, education and performance of our future medical workforce. Understanding how medical education will be affected is therefore important, and in particular how applicants to become the newest entrants to medical careers are being affected. Now more than ever we need medical education, and medical education research, to be prioritised and funded so we can ensure our future doctors are able to be resilient, successful and happy healthcare professionals providing excellent patient care. The present study provides a wide range of insights into the feelings of the 2020 cohort of applicants, only a small proportion of which we have adequately been able to report here.

Acknowledgements

Firstly we are immensely grateful to the several thousand medical school applicants who took the time to respond to survey with a very tight time window, and we particularly thank those who commented that they were pleased that the survey they gave them an opportunity to express their thoughts, feelings and anxieties. We could not include everything that was said, but all comments have been read by the team, and we hope that the current paper summarises some of those many and varied views. We are also grateful to Paul Garrud, Clare Owen, Konstantinos Lulo, and Ewan McNicol for their comments on earlier versions of the questionnaire.

For peer review only - http://bmjopen.bmj.com/site/about/guidelines.xhtml

Contributors

KW, DH and ICM jointly developed the idea for the study, and developed the questionnaire together. DH was responsible for putting the questionnaire online, and for identifying applicants to whom it should be sent, as well as sending text and email reminders. DH and KW cleaned the data, and KW, DH and ICM were all involved in data analysis. The report was written jointly by all three authors, and all authors have read and reviewed the final draft.

Funding

KW is a National Institute for Health Research (NIHR) Career Development Fellow (NIHR CDF-2017-10-008) and is principal investigator for the UKMACS and UKMEDP089 projects supported by the NIHR funding.

DH is funded by NIHR grant CDF-2017-10-008 to KW.

ICM has received no specific funding for this project.

For peer review only

Disclaimers

KW and DH state that this publication presents independent research funded by the National Institute for Health Research (NIHR). The views expressed are those of the authors and not necessarily those of the NHS, the NIHR or the Department of Health and Social Care.

Ethical approval

The study was approved by the UCL Research Ethics Committee Chair on 8th April 2020 as an amendment to the ongoing UKMACS longitudinal questionnaire study (reference: 0511/014).

Funding

Katherine Woolf and David Harrison are funded by a National Institute for Health Research (NIHR) Career Development Fellowship for this research project. This publication presents independent research funded by the National Institute for Health Research (NIHR). The views expressed are those of the author(s) and not necessarily those of the NHS, the NIHR or the Department of Health and Social Care.

Competing interests

All authors have completed the ICMJE uniform disclosure form at http://www.icmje.org/coi_disclosure.pdf : Dr Woolf and Dr Harrison report grants and non-financial support from National Institute for Health Research during the conduct of the study; and Dr Woolf reports personal fees from Transforming Student Access and Outcomes (TASO), outside the submitted work. All authors report no other relationships or activities that could appear to have influenced the submitted work.

Copyright

The Corresponding Author has the right to grant on behalf of all authors and does grant on behalf of all authors, a worldwide licence (http://www.bmj.com/sites/default/files/BMJ%20Author%20Licence%20March%202013.doc) to the Publishers and its licensees in perpetuity, in all forms, formats and media (whether known now or created in the future), to i) publish, reproduce, distribute, display and store the Contribution, ii) translate the Contribution into other languages, create adaptations, reprints, include within

collections and create summaries, extracts and/or, abstracts of the Contribution and convert or allow conversion into any format including without limitation audio, iii) create any other derivative work(s) based in whole or part on the on the Contribution, iv) to exploit all subsidiary rights to exploit all subsidiary rights that currently exist or as may exist in the future in the Contribution, v) the inclusion of electronic links from the Contribution to third party material where-ever it may be located; and, vi) licence any third party to do any or all of the above. All research articles will be made available on an open access basis (with authors being asked to pay an open access fee—see http://www.bmj.com/about-bmj/resources-authors/forms-policies-and-checklists/copyright-open-access-and-permission-reuse). The terms of such open access shall be governed by a Creative Commons licence—details as to which Creative Commons licence will apply to the research article are set out in our worldwide licence referred to above.

Patient and public involvement statement

Patients and the public were consulted in the development of the questionnaire.

Dissemination declaration

We have disseminated the pre-print of this article to those invited to respond.

Data sharing statement

The data will be linked into the UK Medical Education Database **www.ukmed.ac.uk** to which researchers can apply for access.

References

1. Efuribe C, Barre-Hemingway M, Vaghefi E, Ballonoff Suleiman A. Engagement and the Inclusion of Young People in Matters That Affect Their Lives. Journal of Adolescent Health. 2020.
2. McKie A. Scrapped exams may spark UK admissions 'scramble'. Times Higher Education. 2020 26/03/2020.
3. Summer 2020 grades for GCSE, AS and A level, Extended Project Qualification and Advanced Extension Award in maths: Information for Heads of Centre, Heads of Department and teachers on the submission of Centre assessment grades. . Coventry; 2020 03/04/2020. Contract No.: Ofqual/20/6607/1
4. Machin SJ, McNally S, Ruiz-Valenzuela J. Entry Through the Narrow Door: The Costs of Just Failing High Stakes Exams. CESifo Working Paper Series 2018(7008).
5. McManus IC, Woolf K, Dacre J, Paice E, Dewberry C. The Academic Backbone: longitudinal continuities in educational achievement from secondary school and medical school to MRCP(UK) and the specialist register in UK medical students and doctors. BMC Medicine. 2013;11(1):242.
6. Cancellation of GCSE is unfair to some students. The Guardian. 2020 29/03/2020.
7. Lough C. GCSEs: Only 39% teachers think 2020 grades fair for all. Times Educational Supplement. 2020 13/05/2020.
8. (HEPI) HEPI. New poll finds almost one-third of applicants feel less confident they will get into their chosen university since the Coronavirus pandemic. Oxford: Higher Education Policy Institute; 2020 [Available from: https://www.hepi.ac.uk/2020/04/07/new-poll-finds-almost-one-third-of-applicants-feel-less-confident-they-will-get-into-their-chosen-university-since-the-coronavirus-pandemic/;.
9. Montacute R, Holt-White E. COVID-19 and Social Mobility Impact Brief #2: University Access & Student Finance. 2020.

For peer review only - http://bmjopen.bmj.com/site/about/guidelines.xhtml

10. New statement on medical school admissions in 2020: Medical Schools Council; 2020 [updated 05/05/2020. Available from: https://www.medschools.ac.uk/news/new-statement-on-medical-school-admissions-for-2020-entry.
11. Coronavirus (COVID-19): cancellation of GCSEs, AS and A levels in 2020: Department for Education; 2020 [updated 1st May 2020. Available from: https://www.gov.uk/government/publications/coronavirus-covid-19-cancellation-of-gcses-as-and-a-levels-in-2020/coronavirus-covid-19-cancellation-of-gcses-as-and-a-levels-in-2020.
12. Lavy V. Do Differences in Schools' Instruction Time Explain International Achievement Gaps? Evidence from Developed and Developing Countries. The Economic Journal. 2015;125(588):F397-F424.
13. Carlsson M, Dahl GB, Öckert B, Rooth D-O. The Effect of Schooling on Cognitive Skills. The Review of Economics and Statistics. 2015;97(3):533-47.
14. Burgess S, Sievertsen HH. Vox CEPR Policy Portal [Internet]2020. Available from: https://voxeu.org/article/impact-covid-19-education.
15. Andrew A, Cattan S, Costa Dias M, Farquharson C, Kraftman L, Krutikova S, et al. Learning during the lockdown: real-time data on children's experiences during home learning. Institute for Fiscal Studies; 2020.
16. Monitoring COVID-19 Readiness in Schools: Teacher Tapp; 2020 [Available from: https://teachertapp.co.uk/monitoring-covid-19-readiness-in-schools/A
17. Understanding Society: Waves 1-9, 2009-2018 and Harmonised BHPS: Waves 1-18, 1991-2009. [data collection]. 12th Edition. In: University of Essex IfSaER, NatCen Social Research, Kantar Public, editor.: UK Data Service; 2019.
18. van Buuren S, Groothuis-Oudshoorn K. mice: Multivariate Imputation by Chained Equations in R. Journal of Statistical Software 2011;45.
19. Van Buuren S. Flexible imputation of missing data. Second edition ed. New York: CRC Press; 2018.
20. Revelle W. psych: Procedures for Psychological, Psychometric, and Personality Research. Northwestern University, Evanston, Illinois.; 2019.
21. McCrae RC, PT. Personal trait as a human universal. American Psychologist. 1995;52(5).
22. Murphy R, Wyness G. Minority Report: the impact of predicted grades on university admissions of disadvantaged groups. Centre for Education Policy and Equalising Opportunities, UCL Institute of Education; 2020.
23. Lough C. Coronavirus: Williamson warned over grading 'injustice'. Times Educational Supplement 2020 02/04/2020.
24. Predicted grading during COVID-19 could limit young people's futures: Equality and Human Rights Commission; 2020 [Available from: https://www.equalityhumanrights.com/en/our-work/news/predicted-grading-during-covid-19-could-limit-young-peoples-futures.
25. McManus C, Woolf K, Harrison D, Tiffin PA, Paton LW, Yet Fong Cheung K, et al. Calculated grades, predicted grades, forecasted grades and actual A-level grades: Reliability, correlations and predictive validity in medical school applicants, undergraduates, and postgraduates in a time of Covid-19. forthcoming. 2020.
26. Curtis S, Smith D. A comparison of undergraduate outcomes for students from gateway courses and standard entry medicine courses. BMC Medical Education. 2020;20(1):4.
27. Wakeford R, Ludka K, Woolf K, McManus IC. Fitness to practise sanctions in UK doctors are predicted by poor performance at MRCGP and MRCP(UK) assessments: data linkage study. BMC Medicine. 2018;16(1):230.
28. Physicians RCo. Underfunded. Underdoctored. Overstretched. The NHS in 2016. London; 2016.
29. Devine OP, Harborne AC, McManus IC. Assessment at UK medical schools varies substantially in volume, type and intensity and correlates with postgraduate attainment. BMC Medical Education. 2015;15(1):146.

For peer review only - http://bmjopen.bmj.com/site/about/guidelines.xhtml

30. Achieving stability in the higher education sector following COVID-19. Universities UK; 2020 08/04/2020.

For peer review only

Figure captions

Figure 1: Perceptions of the fairness of methods medical schools could use to decide whether or not to accept applicants who currently hold an offer now that exams have been cancelled.

Figure 2: Acceptability of actions medical schools could take if they have more applicants meeting offers than they have places.

Figure 3: Views on how current applicants should be considered by medical schools if they reapply next year.

Figure 4: Aspects of calculated grades that respondents were generally more a) positive and b) negative about

Figure 5: Proportion of respondents undertaking various activities to prepare for medical school or university.

Figure 6: Amount of time respondents reported spending on various activities during the lockdown.

Figure 7: Scree plot for the factor analysis of 87 attitudinal variables.

For peer review only

For peer review only - http://bmjopen.bmj.com/site/about/guidelines.xhtml

Table 1 : Demographics for the full sample and the restricted sample (of those in Year 13, with at least three predicted A-level*s, no achieved* A-level*s, who had applied to study medicine). Rounding to prevent identifying individuals.*

	**Full sample N (%)**	**Restricted sample N (%)**
Female	1968 (68)	1097 (70)
Male	749 (26)	416 (27)
Other	20 (<1%)	<10 (<1)
Missing	140 (5)	Rounded to 40 (3)
White	670 (23)	516 (33)
Asian	301 (11)	228 (15)
Black	79 (3)	58 (4)
Mixed/Other	104 (4)	87 (6)
Missing	1723 (60)	673 (43)
1+ parents with degree	1831 (64)	1046 (67)
First in family	895 (33)	465 (30)
Missing	151 (5)	51 (3)
1+ parents in the highest socioeconomic group	1910 (66)	1097 (70)
No parents in the highest socioeconomic group	1742 (30)	439 (28)
Missing	116 (4)	26 (2)
No parent doctors	2408 (88)	1334 (85)
1+ parents who are doctors	344 (13)	192 (12)
Missing	125 (4)	36 (2)
Non-selective state school	785 (27)	590 (38)
Private or selective school	783 (27)	568 (36)
Missing	1309 (46)	404 (26)
IMD quintile 5 (most deprived – reverse scored)	310 (11)	169 (11)
IMD quintile 4 (reverse scored)	361 (13)	218 (14)
IMD quintile 3 (reverse scored)	410 (14)	236 (15)
IMD quintile 2 (reverse scored)	461 (16)	267 (17)
IMD quintile 1 (least deprived – reverse scored)	704 (25)	441 (28)
Missing	631 (22)	231 (15)
In Year 13/S6	2212 (77)	1562 (100)
One year post-Year 13	179 (6)	0 (0)
Have/studying for a degree	340 (12)	0 (0)
Mature without a degree/other	146 (5)	0 (0)
Missing	0 (0)	0 (0)
England	2003 (70)	1281 (82)
Scotland	170 (6)	<1 (<1)
Wales	78 (3)	50 (3)
Northern Ireland/ Forces/Islands	66 (2)	Rounded to 40 (2)
Other/missing	560 (20)	192 (12)
Total	2877 (100)	1562 (100)

For peer review only - http://bmjopen.bmj.com/site/about/guidelines.xhtml

***Table 2**: Predictors of agreement with statements relating to calculated grades. Predictors are ordered left to right by strength of relationship to the statement. Only statements that showed significant differences by social/demographic group after controlling for prior attainment and the number of offers are shown.*

	**Independent predictors of agreement with statement**			
I would prefer not to have calculated grades at all and instead take A levels (or equivalents) in September.	Lower predicted A level points	BAME	Fewer conditional offers	Female
Overall, I would prefer to withdraw entirely from calculated grades and sit exams properly next summer.	Lower predicted A-level points	BAME	Fewer conditional offers	Female
The process described above is the best way to be fair to most students.	Higher predicted A-level points	White	Higher UCAT/BMAT scores	
I feel confident this process will result in an accurate assessment of my true abilities.	Higher predicted A-level points	White	Male	
Many students do better than their teachers expect; calculated grades cannot take that into account.	Lower predicted A-level points	BAME		
My teachers should take into account the disruption caused by coronavirus when judging grades.	Lower predicted A-level points	BAME		
Calculated grades should be based only on my performance, not on how previous students at my school performed.	Non-Selective State school	Higher deprivation		
I am confident in my teachers' abilities at grading and ranking students.	Higher predicted A-level points	White		
My teachers do not know enough about me to grade and rank me accurately.	Lower predicted A-level points	BAME	Fewer conditional offers	
In large schools/colleges, it is difficult to see how teachers can rank so many students.	BAME	Lower predicted A-level points		
Teachers judging grades should take into account the fact that many students do not do well in mocks but then work hard and do well in exams.	Lower predicted A-level points	Female	Fewer conditional offers	
Employers and universities in the future will treat grades from 2020 differently compared to exam grades taken from other years.	Female	Fewer conditional offers		

For peer review only - http://bmjopen.bmj.com/site/about/guidelines.xhtml

Table 3: School-provided educational resources used by respondents from non-selective state schools and private/selective schools

	N (%) used resource			
	Non selective state school	Private or selective school	Total	p value
Online resources	342 (63.3)	439 (80.0)	781 (71.7)	<.001
Paper resources	315 (58.3)	375 (69.6)	690 (63.9)	<.001
Online formative tests	187 (34.8)	260 (48.2)	447 (41.5)	<.001
Pastoral support	160 (29.7)	199 (37.2)	359 (33.4)	0.009
University application support	152 (28.5)	174 (32.3)	326 (30.4)	0.174
Online teaching in real time	66 (12.4)	248 (45.7)	314 (29.2)	<.001
Online summative tests	70 (13.2)	95 (17.7)	165 (15.4)	0.042
Other	12 (6.3)	25 (14.2)	37 (10.1)	0.011

.

For peer review only

For peer review only - http://bmjopen.bmj.com/site/about/guidelines.xhtml

Table 4: Respondents' main reasons for not using school educational resources during the shutdown by school type

Resource not used	Reason not used	N (%) resource NOT used<br>Non-selective state school	Private or selective school	Total
Online resources	Not available	96 (46.6)	48 (43.2)	144 (45.4)
	Don't need to	80 (38.8)	49 (44.1)	129 (40.7)
Paper resources	Not available	109 (50.5)	74 (46.8)	183 (48.9)
	Don't need to	88 (40.7)	69 (43.7)	157 (42.0)
Online formative test	Not available	206 (60.2)	129 (48.3)	335 (55.0)
	Don't need to	119 (34.8)	116 (43.3)	235 (38.6)
Pastoral support	Not available	161 (42.6)	94 (28.4)	255 (36.0)
	Don't need to	194 (51.3)	205 (61.9)	399 (56.3)
Uni application support	Not available	185 (49.9)	141 (40.5)	326 (45.3)
	Don't need to	155 (41.8)	182 (52.3)	337 (46.9)
Online teaching in real time	Not available	337 (71.7)	189 (63.0)	526 (68.3)
	Don't need to	109 (23.3)	99 (33.0)	208 (27.0)
Online summative test	Not available	289 (65.4)	223 (52.5)	512 (59.1)
	Don't need to	142 (32.1)	177 (41.6)	319 (36.8)
Other	Not available	66 (54.1)	42 (39.3)	108 (47.2)
	Don't need to	47 (38.5)	49 (45.8)	96 (41.9)

.

For peer review only - http://bmjopen.bmj.com/site/about/guidelines.xhtml

Table 5: Simple correlations (Pearson's r) between time spent on various activities during lockdown and big five personality traits. Correlations in bold are significant at p<.001: those in red are negative and those in black are positive.

	Agreeableness	Conscientiousness	Extraversion	Neuroticism	Openness to Experience
Reading about coronavirus	0.056	0.087	0.053	0.033	0.057
Contact with friends	-0.013	-0.005	**0.226**	0.054	-0.025
Watching TV or videos	**-0.160**	**-0.198**	-0.049	**0.195**	-0.067
Gaming with friends	**-0.120**	**-0.141**	-0.043	-0.094	0.020
Gaming alone	**-0.120**	**-0.162**	**-0.153**	-0.010	0.015
Reading for pleasure	0.089	**0.136**	-0.019	-0.020	**0.116**
Exercising	**0.112**	**0.229**	**0.125**	**-0.159**	-0.012
Hobbies	**0.112**	**0.128**	0.020	-0.057	**0.186**
Studying	**0.107**	**0.167**	0.000	-0.054	**0.124**
Chores	**0.124**	0.084	0.021	-0.019	0.064
Caring	**0.139**	0.087	-0.006	0.035	**0.142**
Volunteering	0.022	**0.116**	0.074	-0.043	0.025

For peer review only

Table 6: Predictors of factor scores. Set A includes Number of offers, GCSE points, Predicted A-level points, UCAT/BMAT score, Private/Selective school, Female, BAME, Degree-educated parent(s) and Deprived area. Set B includes Set A plus Highest socioeconomic group, doctor parent(s), and Big5 personality factors Agreeableness, Conscientiousness, Extraversion, Neuroticism and Openness. All predictors reported have p<.01, and are reported in descending order of significance (i.e. most significant at the top).

	**Set A Predictors in order of magnitude**	**Set B Predictors in order of magnitude**
Factor 1: Lack of confidence in calculated grades	Lower predicted A-levels<br>BAME<br>Fewer conditional offers<br>Female	Lower predicted A-levels<br>BAME<br>Fewer conditional offers<br>Higher Openness<br>Lower Conscientiousness<br>Female
Factor 2: Special treatment next year for rejected applicants	Lower predicted A-levels<br>Lower UCAT/BMAT	Lower predicted A-levels<br>Higher Openness<br>Lower UCAT/BMAT<br>Higher Neuroticism<br>Higher Extraversion
Factor 3: Other selection measures to be taken into account	Higher UCAT/BMAT<br>Lower predicted A-levels<br>Male	Higher UCAT/BMAT<br>Lower predicted A-levels<br>Higher Extraversion<br>Male<br>Lower Conscientiousness
Factor 4: Preparing for medical school	White<br>Female	Higher Conscientiousness<br>Lower Neuroticism<br>White<br>Female<br>Higher Agreeableness<br>Higher Openness
Factor 5: Importance of background and experience	Lower UCAT/BMAT<br>BAME<br>Female	Higher Openness<br>Lower UCAT/BMAT<br>Fewer conditional offers<br>BAME
Factor 6: Resources from school for home study	Selective school<br>Lower GCSE<br>Fewer conditional offers	Selective School<br>Lower GCSE<br>Lower Extraversion<br>Higher Openness

For peer review only - http://bmjopen.bmj.com/site/about/guidelines.xhtml

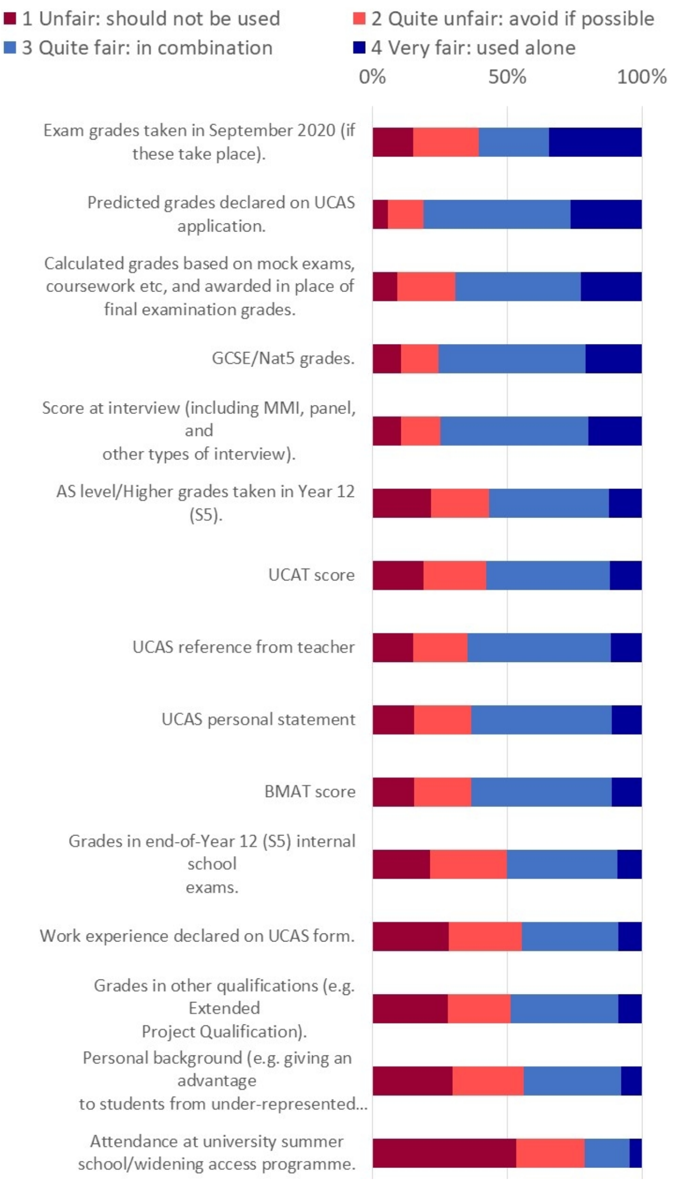

Figure 1: Perceptions of the fairness of methods medical schools could use to decide whether or not to accept applicants who currently hold an offer now that exams have been cancelled.

190x338mm (96 x 96 DPI)

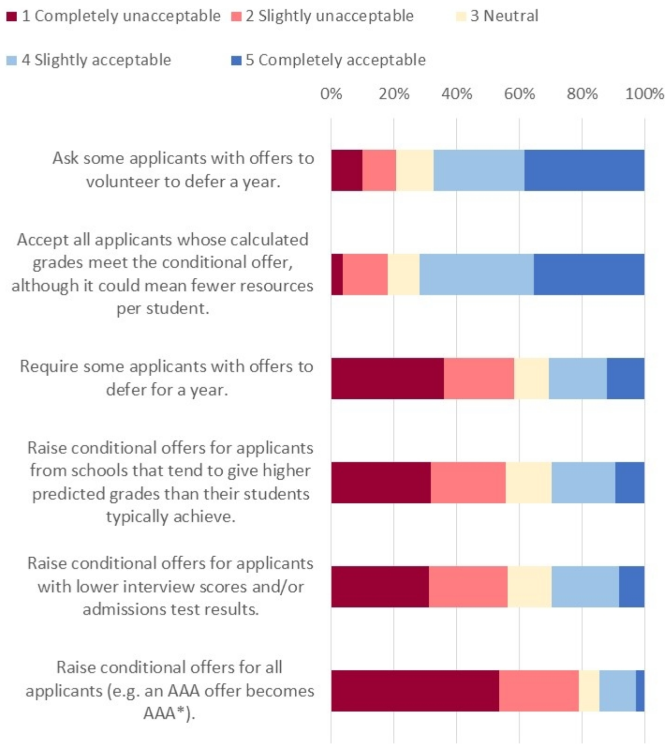

Figure 2: Acceptability of actions medical schools could take if they have more applicants meeting offers than they have places.

190x338mm (96 x 96 DPI)

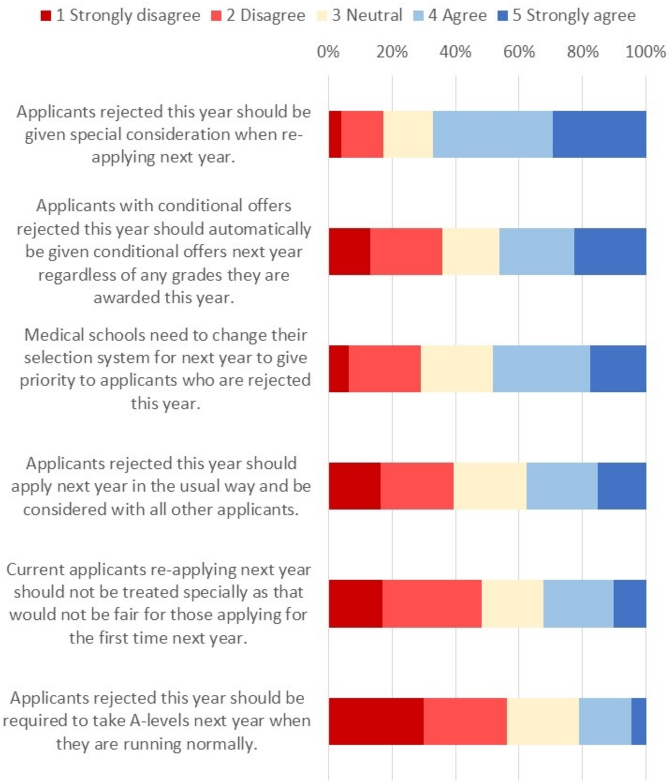

Figure 3: Views on how current applicants should be considered by medical schools if they reapply next year (for entry in 2021).

190x338mm (96 x 96 DPI)

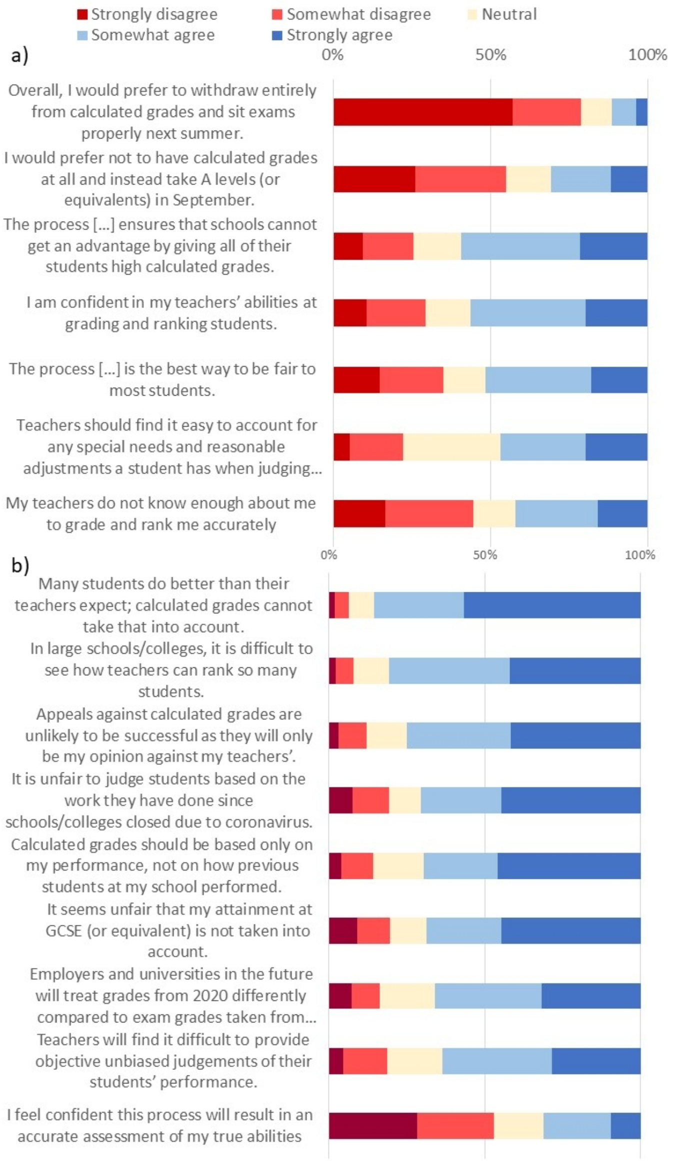

Figure 4: Aspects of calculated grades that respondents were generally more a) positive and b) negative about.

190x338mm (96 x 96 DPI)

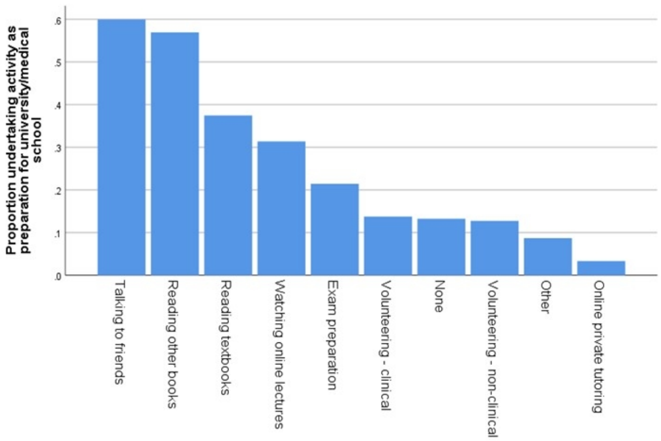

Figure 5: Proportion of respondents undertaking various activities to prepare for medical school or university.

190x338mm (96 x 96 DPI)

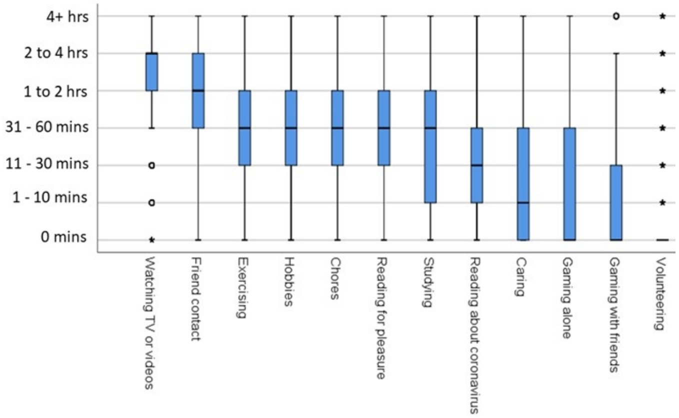

Figure 6: Amount of time respondents reported spending on various activities during the lockdown.

190x338mm (96 x 96 DPI)

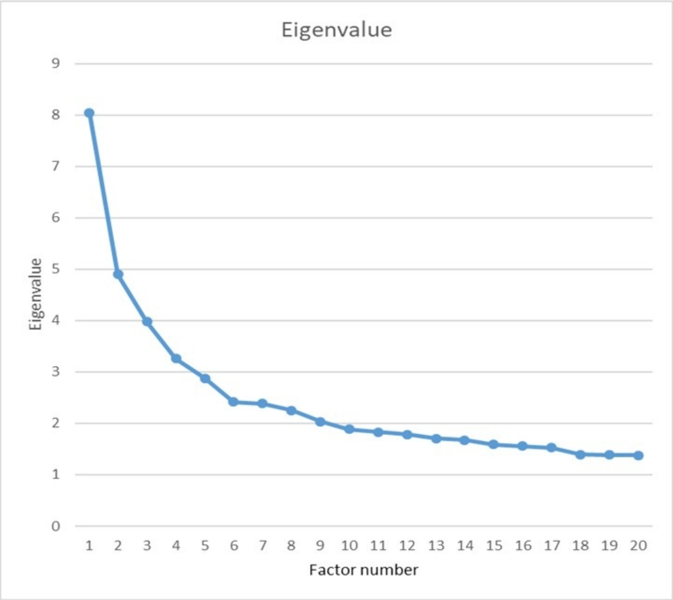

Figure 7: Scree plot for the factor analysis of 87 attitudinal variables.

190x338mm (96 x 96 DPI)

Supplementary file 1: Results for the 665 post-Year 13 respondents excluded from the restricted sample.

This sample includes mature and graduate applicants from the whole of the UK.

Applicant views on admissions

Perceptions of the fairness of methods to select or reject offer holders

As with the restricted sample, no single method was perceived as fair enough to use on its own but many were considered fair enough to use in conjunction with others.

Since this group includes those currently at university and graduate applicants, we have included responses to two additional items: *For those in their final year at university, marks earlier in their course*, which was considered very fair by 35% and quite fair by 45%, and *GAMSAT score (for Graduate Entry students)* which was considered very fair by 17.6% and quite fair by 46.8%.

Compared to those in Year 13, *Predicted grades declared on UCAS form* were considered much less fair and *Personal background (e.g. giving an advantage to students from under-represented groups)* was considered by a majority (52.1%) to be very fair/quite fair.

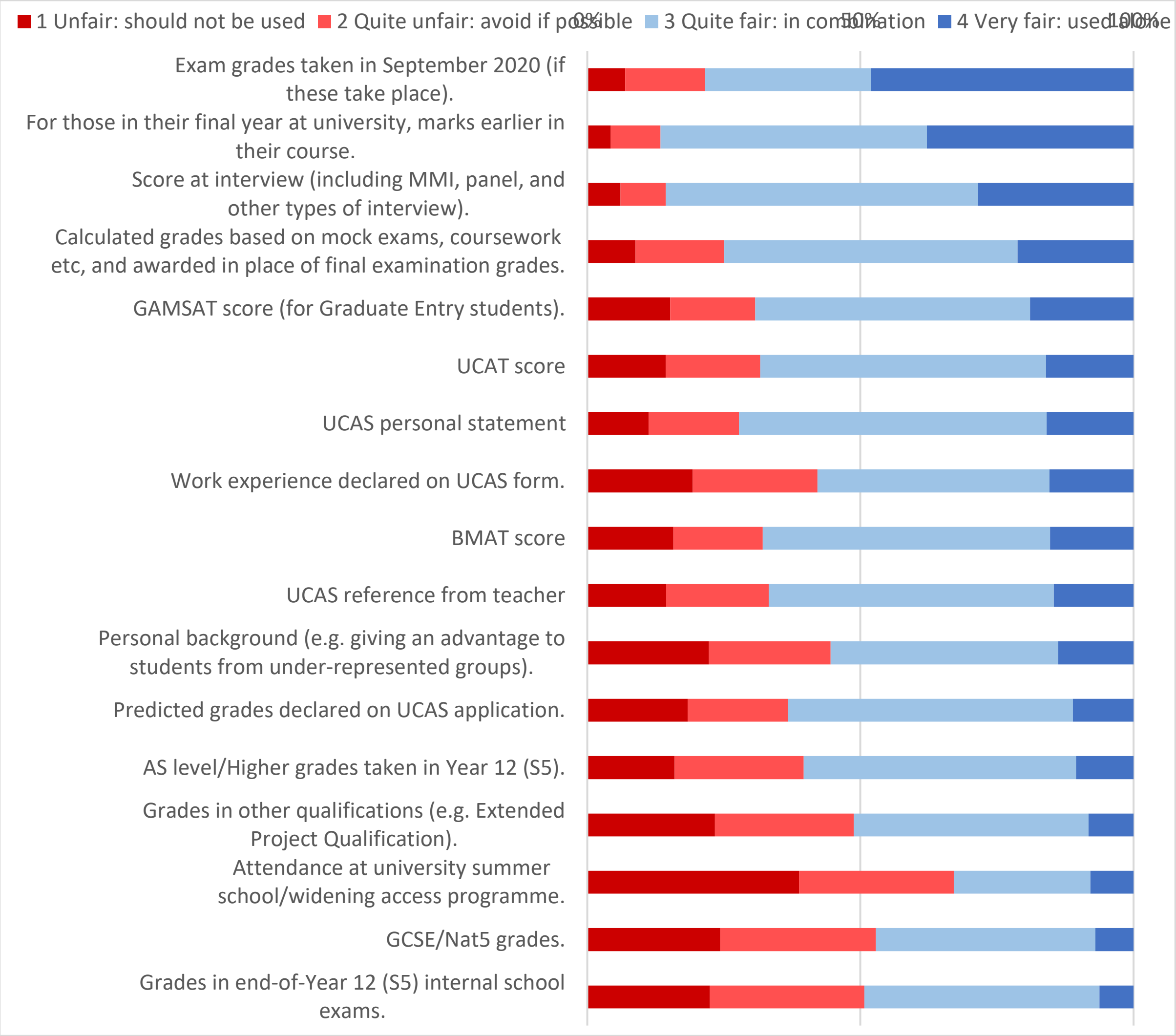

Figure S1: Perceptions of the fairness of methods medical schools could use to decide whether or not to accept applicants who currently hold an offer now that exams have been cancelled. Post-Year13 respondents only.

Acceptability of options for dealing with a situation in which more students meet their offers than there are medical school places

The only option that was rated as slightly or completely acceptable by the majority of respondents (64.6%) was asking for volunteers to defer. Accepting all applicants who meet the conditional offer was the second most acceptable and more acceptable than it was unacceptable.

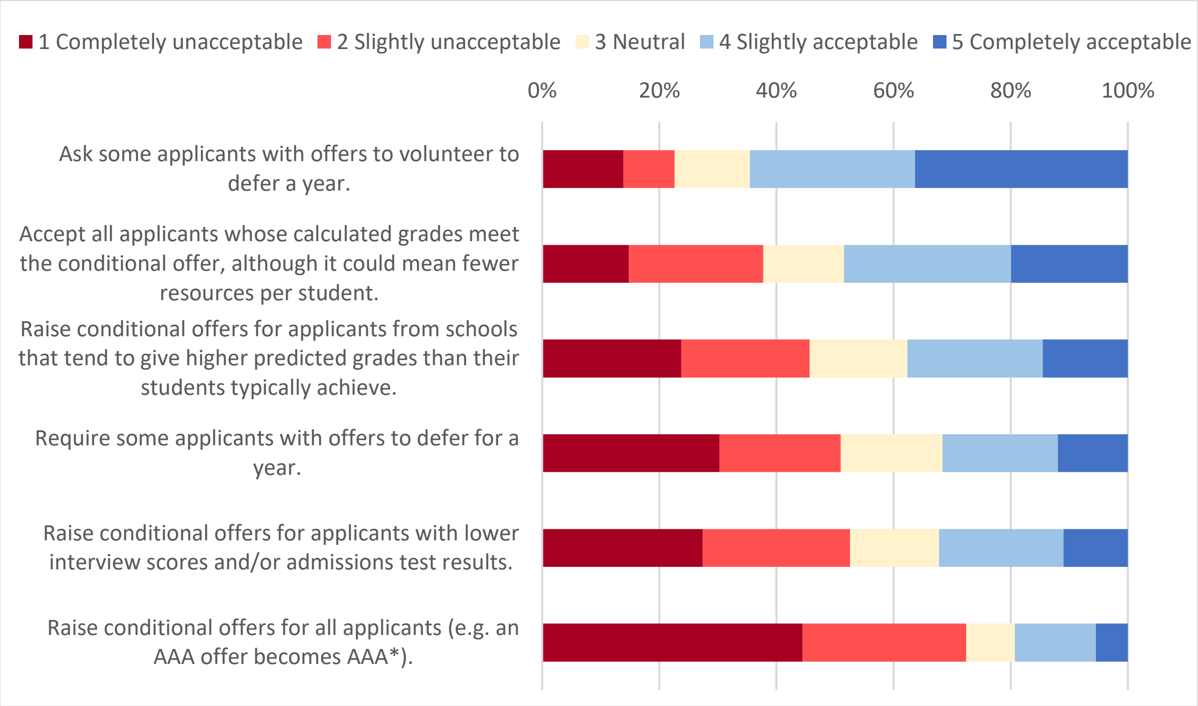

Figure S2: Acceptability of actions medical schools could take if they have more applicants meeting offers than they have places. Post-Year 13 respondents only.

Perceptions of potential impact on admissions for 2021

Respondents were even more divided than in the restricted sample, with about half of respondents (53.8%) agreeing/strongly agreeing that *Applicants rejected this year should be given special consideration when re-applying next year* but 51.5% agreeing/strongly agreeing that *Applicants rejected this year should apply next year in the usual way and be considered with all other applicants*.

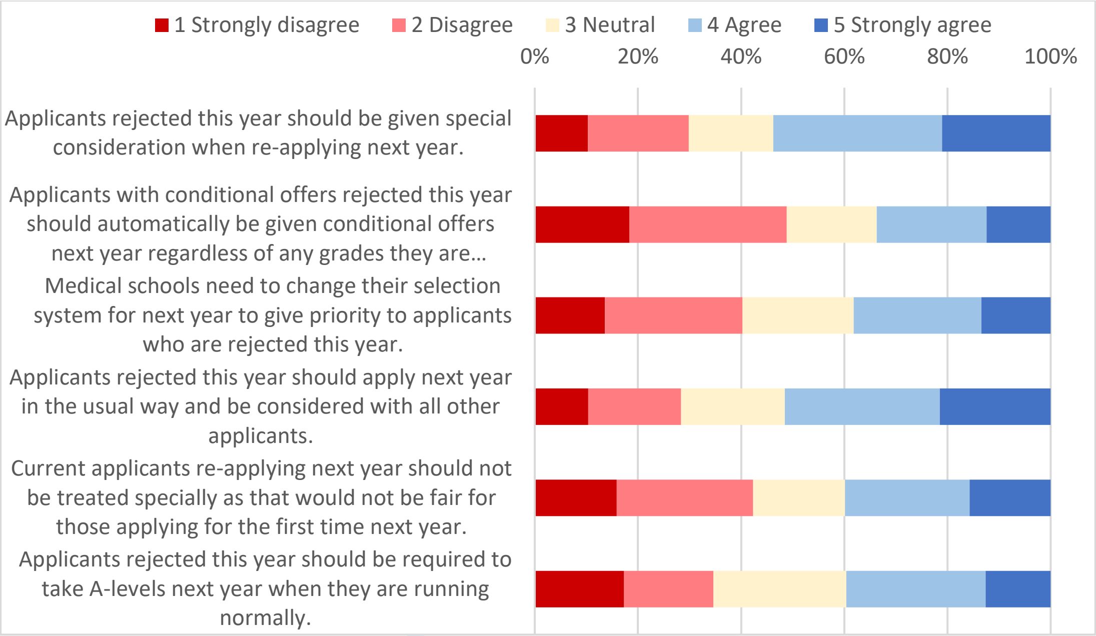

Figure S3: Views on how current applicants should be considered by medical schools if they reapply next year. Post-Year 13 respondents only.

Starting academic year 2020/2021

A majority of respondents (n=375, 56.4%) believed that if necessary, medical schools should *Defer the start of the academic year only when face-to-face teaching is possible* with 285 respondents (42.6.9%) believing that medical schools should *Start the academic year on time using distance learning for as long as is necessary*.

Education and university preparation

Perceptions of process to award calculated grades in lieu of examination grades

Post-Year 13 respondents were generally more negative about calculated grades than respondents in the restricted sample and unsurprisingly there were more “neutral” responses in general and specifically to questions about their own teachers and grades.

For peer review only

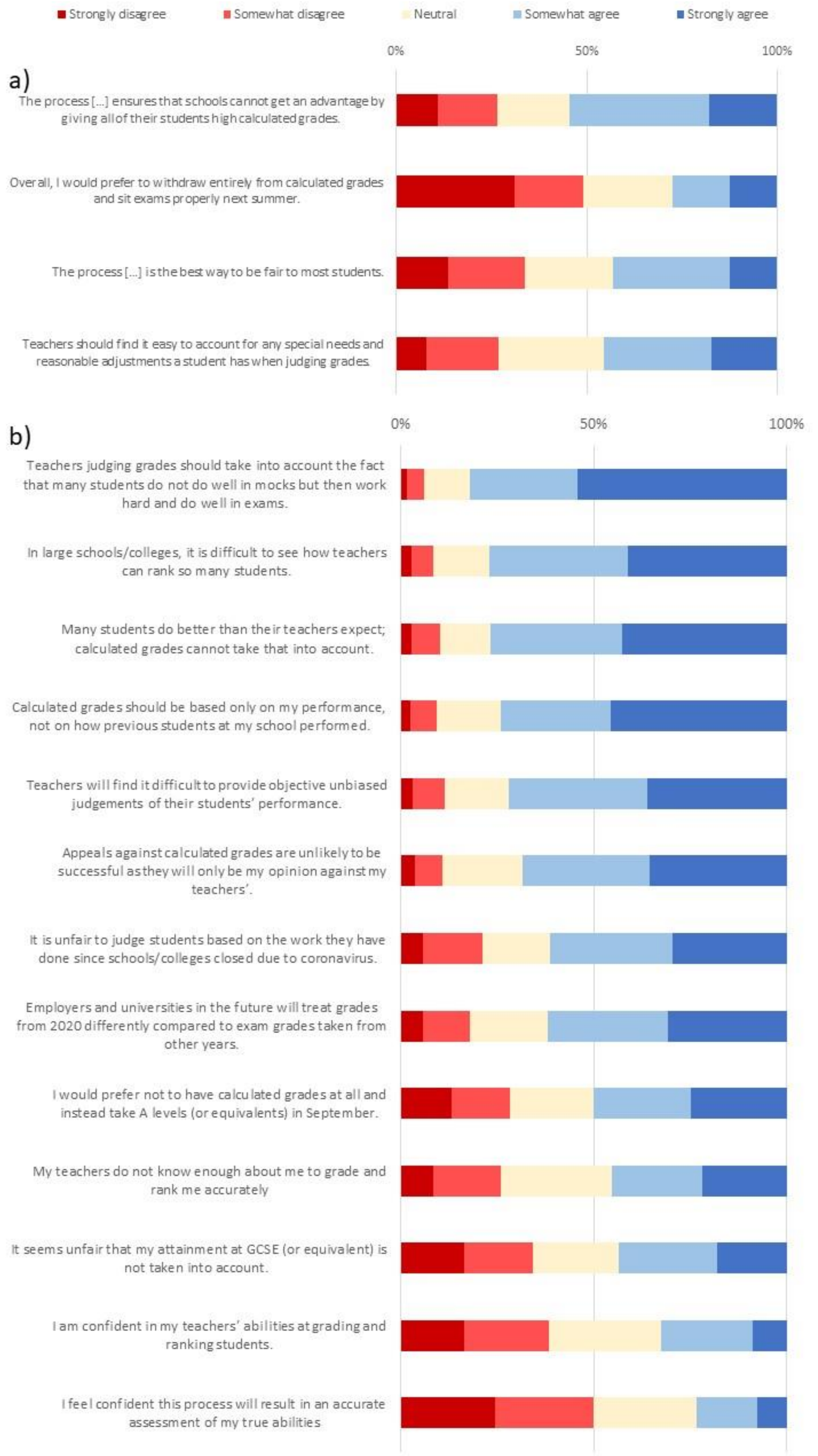

Figure S4: Aspects of calculated grades that respondents were generally more a) positive and b) negative about. Post-Year 13 respondents only.

A number of applicants were re-sitting their examinations and/or were not studying at a school or college but nonetheless were due to take examinations this summer (so-called ‘private candidates’). Several expressed concerns about whether the institution they were due to take their exams with would give them a calculated grade, and if so, what information that grade would be based on:

> *“As a resit student, my previous college which I was registered to retake my exams with this year have decided that they cannot give me calculated grades. I am unsure how to maintain my offers despite not getting grades.”*

> *“I am extremely concerned about how offers made to private candidates who cannot get predicted grades from a school will be treated. Though I had been studying in my lunchtimes/evenings/weekends for over a year, I quit my job 4 days after getting an offer from [redacted] in order to have time to put the work in to get the grades I need. I achieved straight A*s at GCSEs and A-level, so I know how much work it takes to get top grades. I am terrified universities I have offers from will wash their hands of me as I don't have any grades, or forced to defer for a year because universities won't wait for September exam results. Ofqual and exams boards keep saying no student will be disadvantaged, but it appears private candidates like myself may fall through the cracks.”*

> *“I worry that I, as a resitting privately tutored student, will be disadvantaged by the "calculated grades system", as I haven't been in school this year and thus have no exams or schoolwork that could be provided as evidence to support a predicted grade.”*

> *“I’m worried about how they’ll handle resits who have been independently studying as I need to go from a B to an A but am worrying that my old school(exam centre) won’t provide me with a grade even though I’m certain that I’d be able to get an A had I taken the exam. I also can’t afford to take another gap year so I’m hoping unis will take situations like these independently as it would be very unlikely that I’d receive the same grade as last year had I resat.”*

> *“For exam centre who cannot provide grades for resit external students please consider our previous attainment especially if for an applicant like myself has achieved AAB grades from last year and narrowly missed the A grade in Maths by 8 marks. It would be unfair for me to have to take another gap year if I don’t receive a grade this summer.”*

Education since the shutdown

Although participants were post Year-13 many were still in education, whether at school, college or university. The mean number of resources used by participants was 2.9 (SD=1.86).

Like Year 13 respondents, post-Year 13 respondents were using mostly online and paper resources, but 42.8% of post-Year 13 respondents reported having online teaching in real time and nearly half (49.6%) were having online summative tests and; 30.6% reported that their school/college/university would be assessing them formally on work since the closure of schools (although 42.1% reported that this was not applicable to them). See Figure S5.

For peer review only

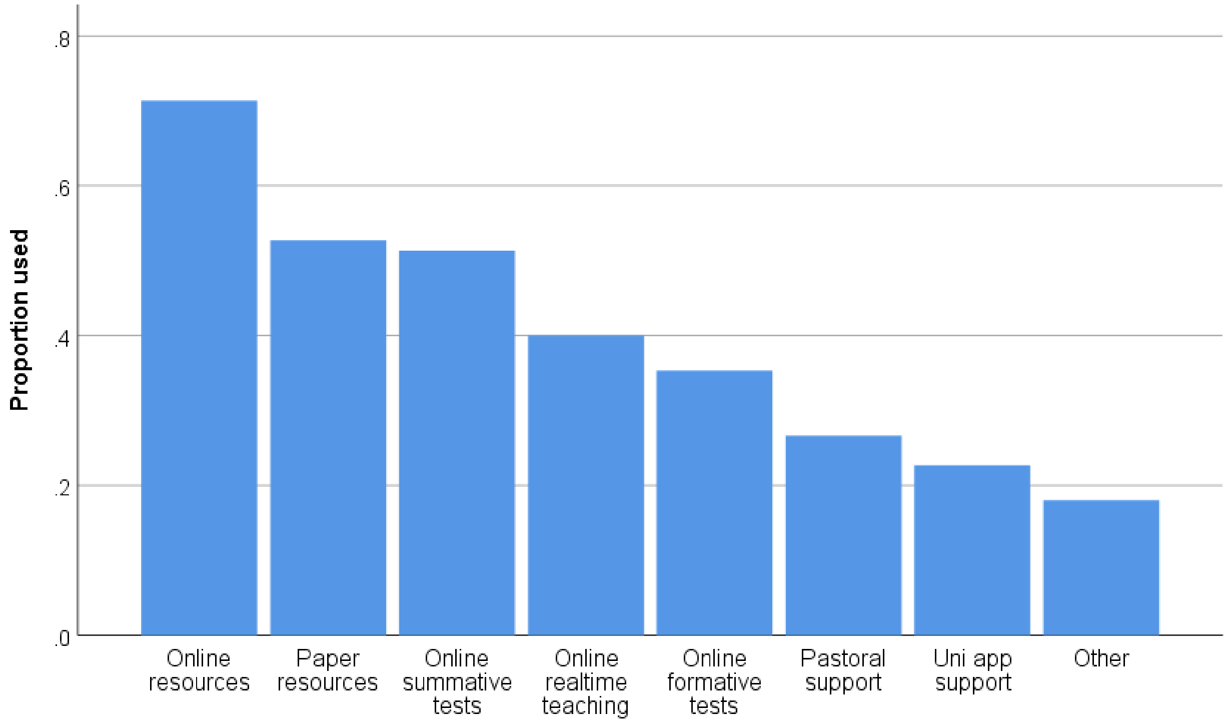

Figure S5: Proportion of post-Year 13 respondents using educational resources since the closure of schools.

Preparation for medical school/university

Post-Year 13 respondents were doing similar sorts of preparation, although they were talking to their friends less. Of the 100 (15.0% of the sample) who said they were not doing any preparation, reasons were different from those in the restricted sample. They were five times more likely to say they did not have time (31.0% vs 6.3%), about half as likely to say they were too worried and not able to focus (26.0% vs 42.5%), and over half as likely to say they did not have resources (15.0% vs 29.5%). A similar percentage selected caring for others as a reason (13.0%), not going to university this year (19.0%), being unwell (6.0%).Respondents could select multiple reasons.

For peer review only

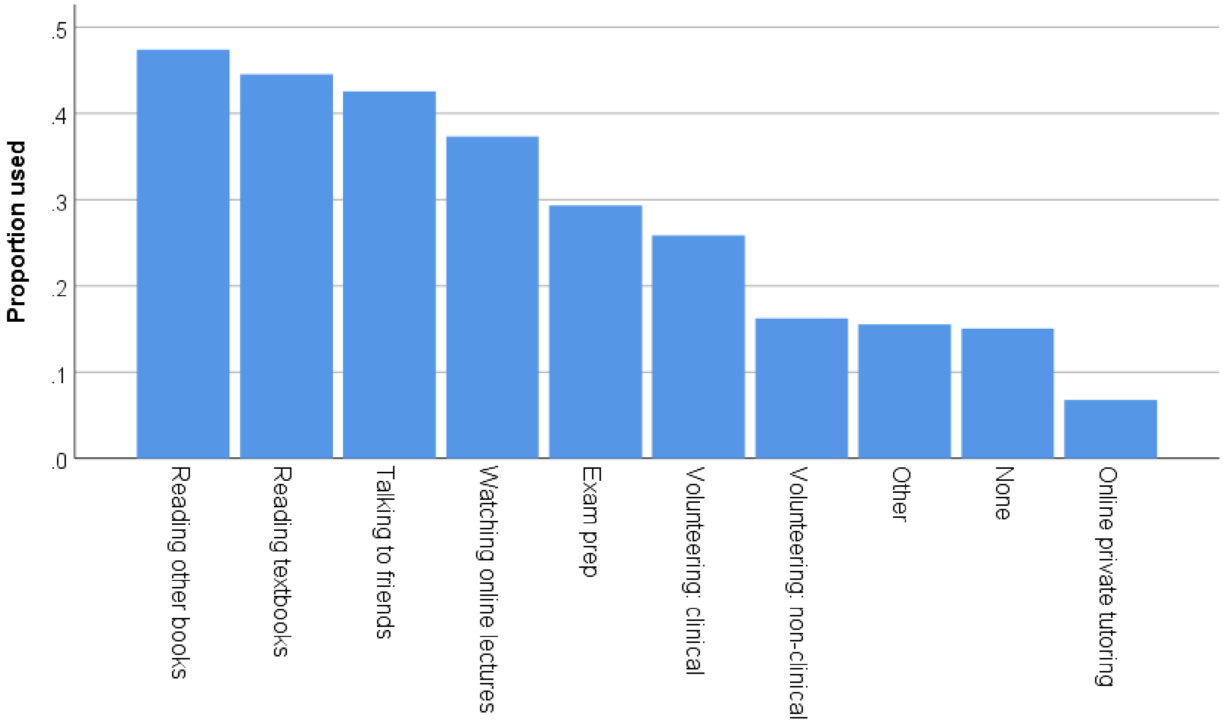

Figure S6: Proportion of respondents undertaking various activities to prepare for medical school or university. Post-Year 13 respondents only.

Time spent during the lockdown

Post-Year 13 respondents were spending broadly similar amounts of time on various activities as those in the restricted sample although they were spending more time volunteering and reading about coronavirus, and less time studying and gaming with friends.

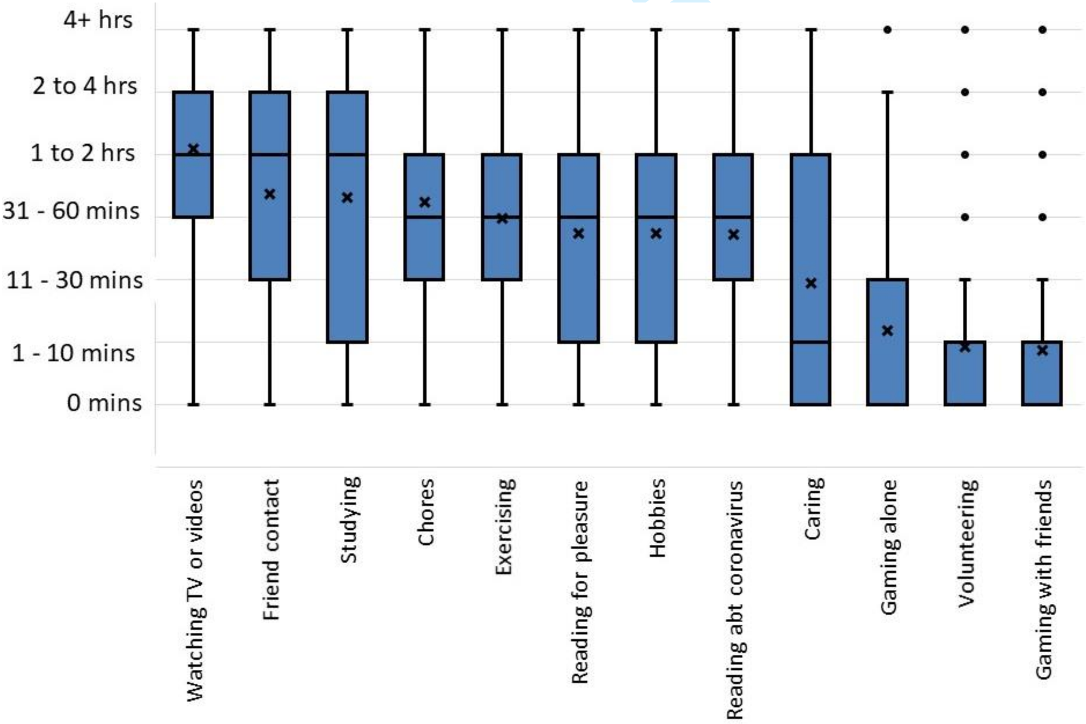

Figure S7: Amount of time respondents reported spending on various activities during the lockdown. Non-Year 13 respondents only.

For peer review only

Supplementary file 2: Results for 125 Scottish S6 respondents excluded from the main analyses

Applicant views on admissions

Perceptions of the fairness of methods to select or reject offer holders

Scottish applicants were similarly uncertain that any measure was fair enough to use alone, however unlike applicants from other UK countries they were more positive about the fairness of using AS level/Higher grades taken in Year 12. This is probably because AS levels are no longer in widespread use whereas Highers are. Scottish applicants were also relatively more positive about the use of calculated grades (83.2% quite or very fair).

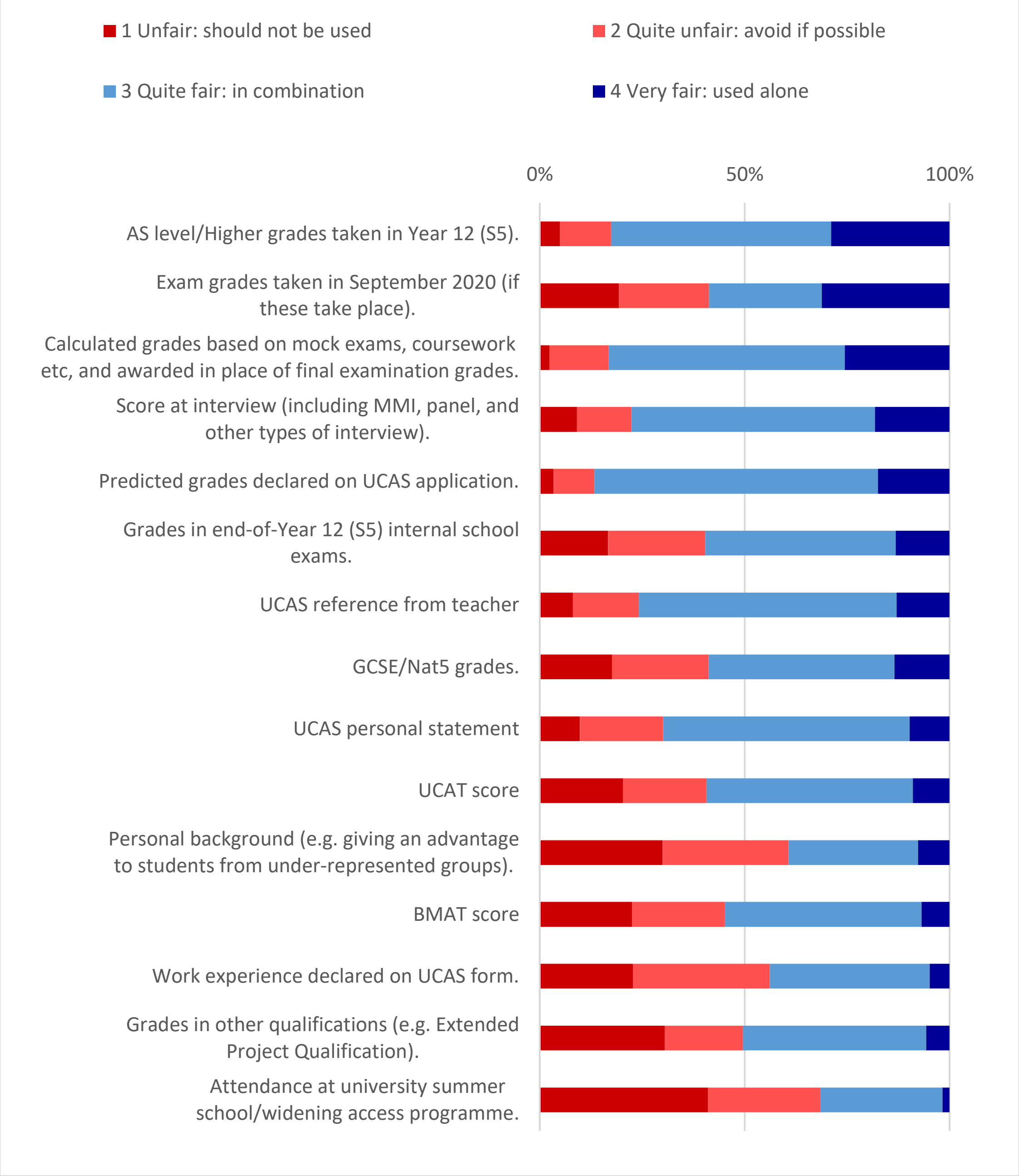

Figure S8: Perceptions of the fairness of methods medical schools could use to decide whether or not to accept applicants who currently hold an offer now that exams have been cancelled. Post-Scottish S6 respondents only.

Acceptability of options for dealing with a situation in which more students meet their offers than there are medical school places

As with other school students, the two acceptable options were accepting all applicants and asking for volunteers to defer.

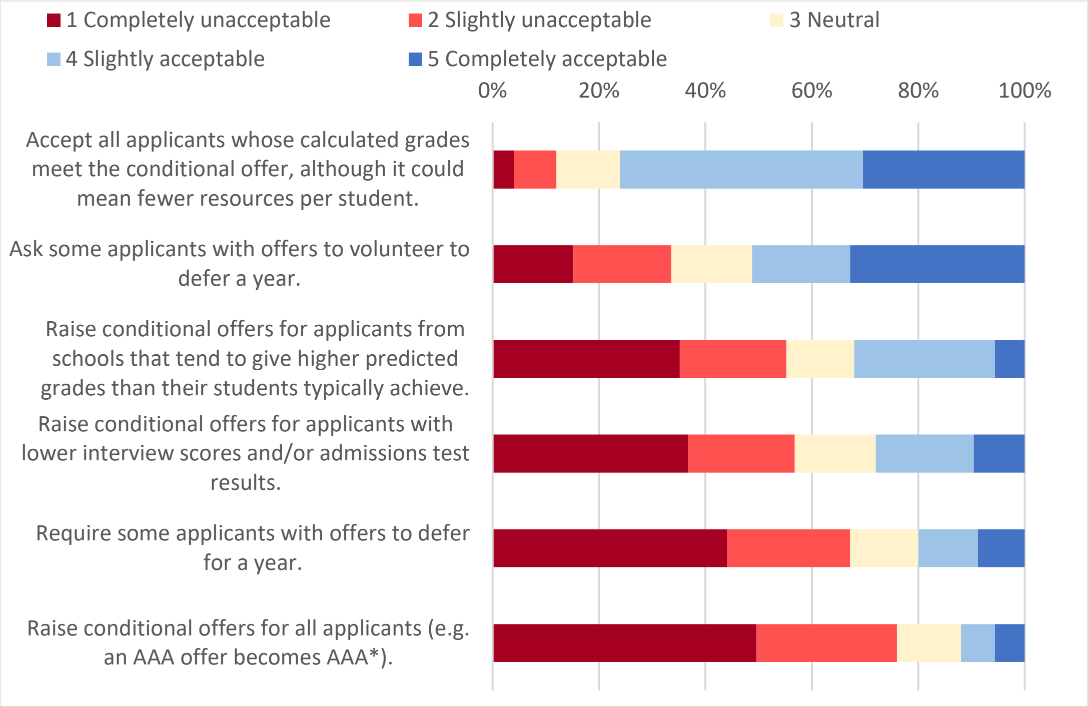

Figure S9: Acceptability of actions medical schools could take if they have more applicants meeting offers than they have places. Scottish S6 respondents only.

Perceptions of potential impact on admissions for 2021

Scottish S6 respondents were even more divided than in other UK countries: half the sample (52.8%) agreed that applicants rejected this year should be given special consideration and half (53.2%) agreeing that they should reapply next year in the usual way and be considered with all other applicants.

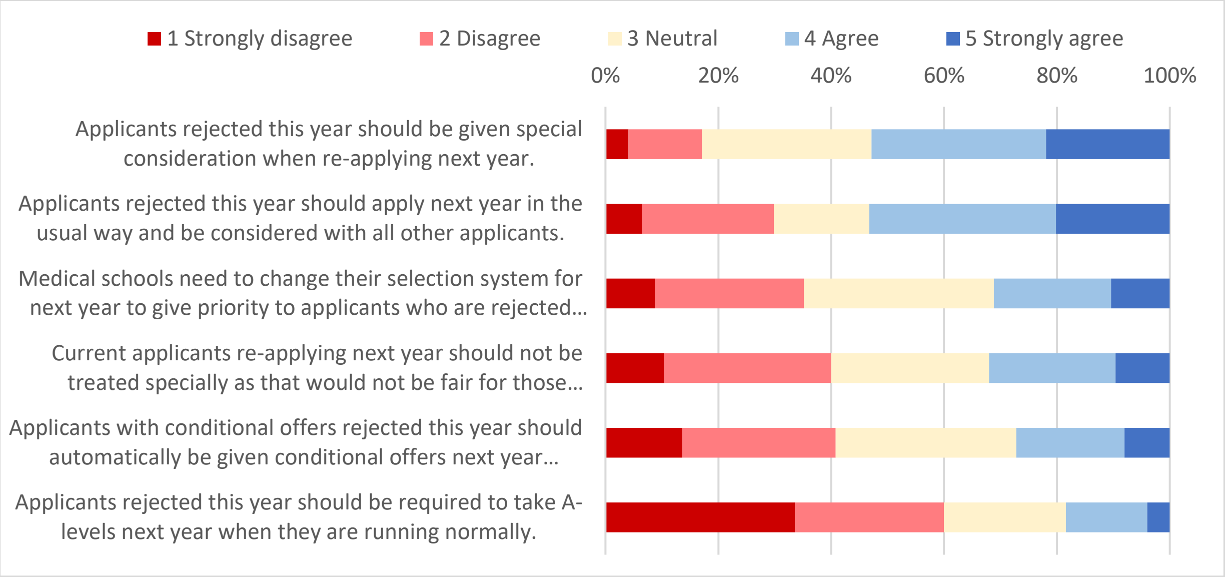

Figure S10: Views on how current applicants should be considered by medical schools if they reapply next year. Scottish S6 respondents only.

Starting academic year 2020/2021

A majority of respondents (n=70; 56.0%) believed that if necessary, medical schools should *Defer the start of the academic year only when face-to-face teaching is possible* with 55 respondents (44.0%) believing that medical schools should *Start the academic year on time using distance learning for as long as is necessary*.

Education and university preparation

Perceptions of process to award calculated grades in lieu of examination grades

Scottish respondents were generally slightly more positive about calculated grades than their equivalents in other UK countries. They were more positive about their teacher's ability to rank and grade students accurately (70.4% agree/strongly agree) and that their teachers knew them well enough to rank and grade them personally (59.2% agree/strongly agree). On the negative side they had similar levels of concern about other aspects of calculated grades as did school students in other UK countries.

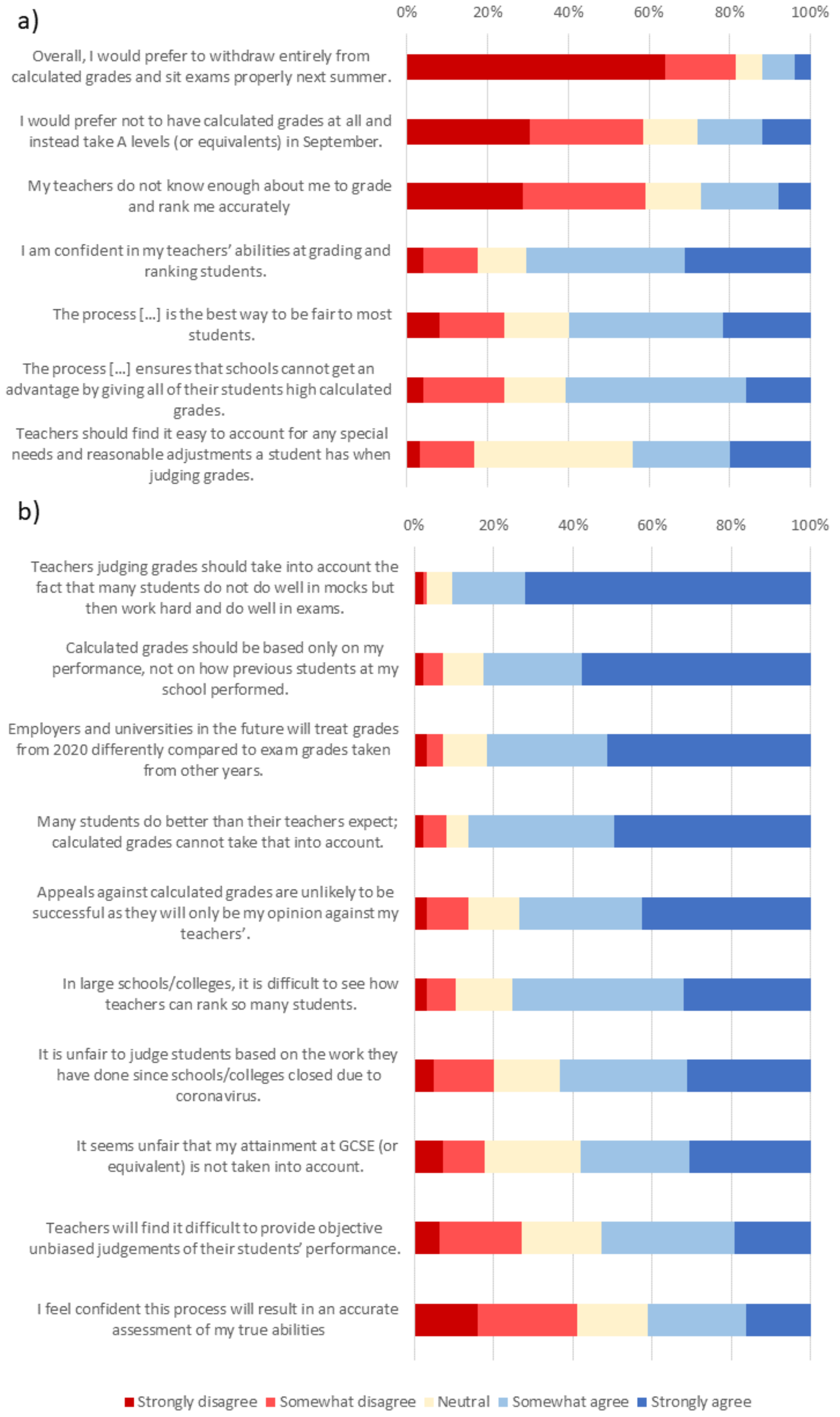

Figure S11: Aspects of calculated grades that respondents were generally more a) positive and b) negative about. Scottish S6 respondents only.

Education since the shutdown

Scottish S6 respondents used on average 2.2 (SD=1.6) educational resources provided by their school, which is fewer than those in the restricted sample. Figure S12 shows Scottish S6 used fewer of all resources compared to the restricted sample, with the exception of summative tests which they were more than twice as likely to use. Scottish S6 students were also more than twice as likely to say their school was assessing them on work since schools closed (n=37; 29.6) with a similar number (n=35; 28.0%) being unsure, and a larger proportion (n=49; 39.2%) saying they were not being assessed.

	Scotland S6	Restricted sample
Online resources	67 (59.8)	781 (71.7)
Paper resources	37 (33.6)	690 (63.9)
Online formative tests	22 (20.0)	447 (41.5)
Pastoral support	32 (29.1)	359 (33.4)
University application support	25 (23.4)	326 (30.4)
Online teaching in real time	31 (27.7)	314 (29.2)
Online summative tests	38 (34.2)	165 (15.4)
Other	<5 (<10)	37 (10.1)

Figure S12: Educational resources provided by schools used in the Scottish and Restricted samples.

Preparation for medical school/university

Scottish applicants were doing similar sorts of preparation as those in the restricted sample; although they were half as likely to be doing examination preparation (n=14; 11.2% vs n=335; 21.4%). Only 19 (15.2%) said they were not doing any preparation which meant numbers were too small to look at reasons for not doing preparation.

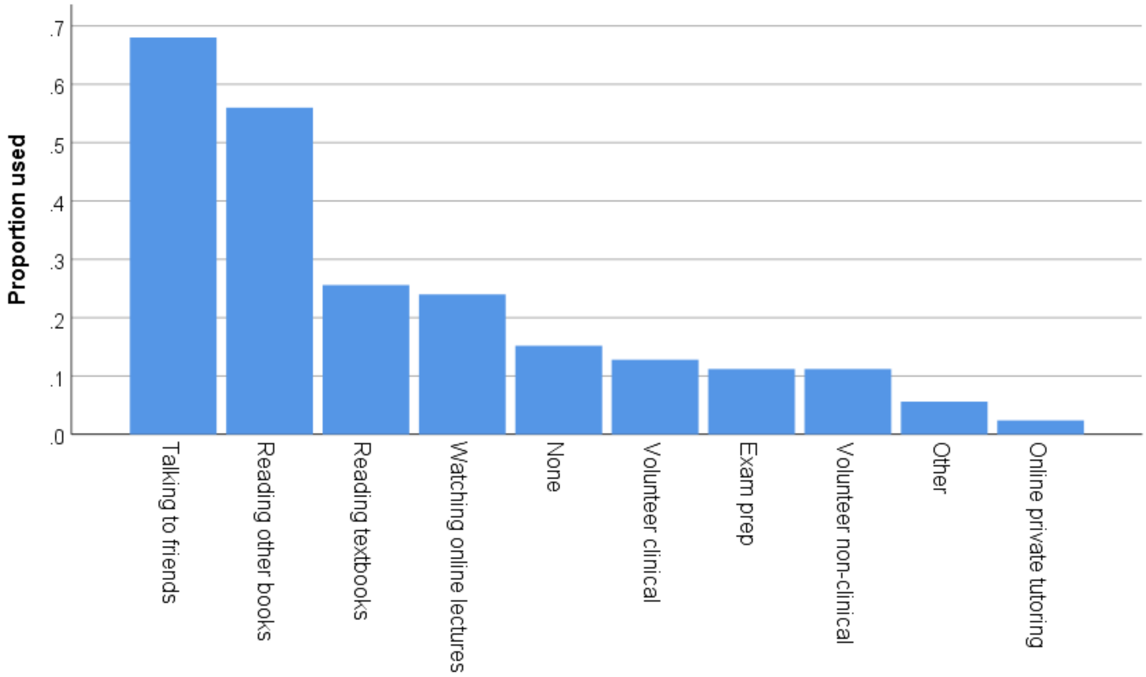

Figure S13: Proportion of respondents undertaking various activities to prepare for medical school or university. Scottish S6 respondents only.

Time spent during the lockdown

The Scottish S6 sample reported similar amounts of time spent on activities as the restricted sample.

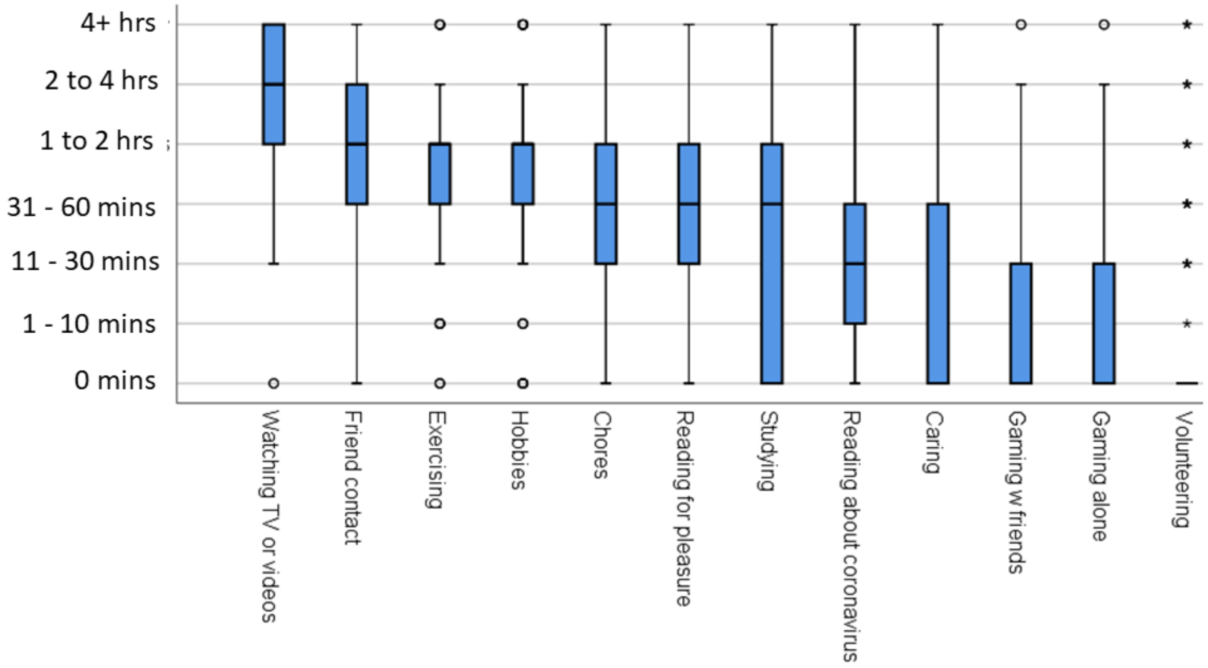

Figure S:14 Amount of time respondents reported spending on various activities during the lockdown. Scottish S6 respondents only.

STROBE Statement—checklist of items that should be included in reports of observational studies

	Item No	Recommendation	Page No.
**Title and abstract**	1	(*a*) Indicate the study's design with a commonly used term in the title or the abstract	2
		(*b*) Provide in the abstract an informative and balanced summary of what was done and what was found	2
**Introduction**			
Background/rationale	2	Explain the scientific background and rationale for the investigation being reported	3-5
Objectives	3	State specific objectives, including any prespecified hypotheses	5
**Methods**			
Study design	4	Present key elements of study design early in the paper	6
Setting	5	Describe the setting, locations, and relevant dates, including periods of recruitment, exposure, follow-up, and data collection	6,7
Participants	6	(*a*) *Cross-sectional study*—Give the eligibility criteria, and the sources and methods of selection of participants	6,7
Variables	7	Clearly define all outcomes, exposures, predictors, potential confounders, and effect modifiers. Give diagnostic criteria, if applicable	8
Data sources/ measurement	8*	For each variable of interest, give sources of data and details of methods of assessment (measurement). Describe comparability of assessment methods if there is more than one group	8
Bias	9	Describe any efforts to address potential sources of bias	8
Study size	10	Explain how the study size was arrived at	7
Quantitative variables	11	Explain how quantitative variables were handled in the analyses. If applicable, describe which groupings were chosen and why	7,8
Statistical methods	12	(*a*) Describe all statistical methods, including those used to control for confounding	8
		(*b*) Describe any methods used to examine subgroups and interactions	8
		(*c*) Explain how missing data were addressed	8
		(*d*) *Cross-sectional study*—If applicable, describe analytical methods taking account of sampling strategy	NA
		(*e*) Describe any sensitivity analyses	NA

Continued on next page

For peer review only - http://bmjopen.bmj.com/site/about/guidelines.xhtml

Results			Page No.
Participants	13*	(a) Report numbers of individuals at each stage of study—eg numbers potentially eligible, examined for eligibility, confirmed eligible, included in the study, completing follow-up, and analysed	7
		(b) Give reasons for non-participation at each stage	NA
		(c) Consider use of a flow diagram	NA
Descriptive data	14*	(a) Give characteristics of study participants (eg demographic, clinical, social) and information on exposures and potential confounders	9
		(b) Indicate number of participants with missing data for each variable of interest	9
		(c) *Cohort study*—Summarise follow-up time (eg, average and total amount)	NA
Outcome data	15*	*Cross-sectional study*—Report numbers of outcome events or summary measures	NA
Main results	16	(*a*) Give unadjusted estimates and, if applicable, confounder-adjusted estimates and their precision (eg, 95% confidence interval). Make clear which confounders were adjusted for and why they were included	10-24
		(*b*) Report category boundaries when continuous variables were categorized	9,10
		(*c*) If relevant, consider translating estimates of relative risk into absolute risk for a meaningful time period	NA
Other analyses	17	Report other analyses done—eg analyses of subgroups and interactions, and sensitivity analyses	7
**Discussion**			
Key results	18	Summarise key results with reference to study objectives	24,25
Limitations	19	Discuss limitations of the study, taking into account sources of potential bias or imprecision. Discuss both direction and magnitude of any potential bias	25,26
Interpretation	20	Give a cautious overall interpretation of results considering objectives, limitations, multiplicity of analyses, results from similar studies, and other relevant evidence	26,27
Generalisability	21	Discuss the generalisability (external validity) of the study results	26
**Other information**			
Funding	22	Give the source of funding and the role of the funders for the present study and, if applicable, for the original study on which the present article is based	7

Note: An Explanation and Elaboration article discusses each checklist item and gives methodological background and published examples of transparent reporting. The STROBE checklist is best used in conjunction with this article (freely available on the Web sites of PLoS Medicine at http://www.plosmedicine.org/, Annals of Internal Medicine at http://www.annals.org/, and Epidemiology at http://www.epidem.com/). Information on the STROBE Initiative is available at www.strobe-statement.org.

For peer review only

For peer review only - http://bmjopen.bmj.com/site/about/guidelines.xhtml

The attitudes, perceptions and experiences of medical school applicants following the closure of schools and cancellation of public examinations in 2020 due to the COVID-19 pandemic: a cross-sectional questionnaire study of UK medical applicants

Journal:	*BMJ Open*
Manuscript ID	bmjopen-2020-044753.R1
Article Type:	Original research
Date Submitted by the Author:	04-Jan-2021
Complete List of Authors:	Woolf, Katherine; University College London Medical School, Research Department for Medical Education<br>Harrison, David; University College London Medical School, Research Department for Medical Education<br>McManus, Chris; University College London Medical School, Research Department of Medical Education
**Primary Subject Heading**:	Medical education and training
Secondary Subject Heading:	Health policy
Keywords:	MEDICAL EDUCATION & TRAINING, EDUCATION & TRAINING (see Medical Education & Training), SOCIAL MEDICINE, COVID-19

SCHOLARONE™
Manuscripts

I, the Submitting Author has the right to grant and does grant on behalf of all authors of the Work (as defined in the below author licence), an exclusive licence and/or a non-exclusive licence for contributions from authors who are: i) UK Crown employees; ii) where BMJ has agreed a CC-BY licence shall apply, and/or iii) in accordance with the terms applicable for US Federal Government officers or employees acting as part of their official duties; on a worldwide, perpetual, irrevocable, royalty-free basis to BMJ Publishing Group Ltd ("BMJ") its licensees and where the relevant Journal is co-owned by BMJ to the co-owners of the Journal, to publish the Work in this journal and any other BMJ products and to exploit all rights, as set out in our licence.

The Submitting Author accepts and understands that any supply made under these terms is made by BMJ to the Submitting Author unless you are acting as an employee on behalf of your employer or a postgraduate student of an affiliated institution which is paying any applicable article publishing charge ("APC") for Open Access articles. Where the Submitting Author wishes to make the Work available on an Open Access basis (and intends to pay the relevant APC), the terms of reuse of such Open Access shall be governed by a Creative Commons licence – details of these licences and which Creative Commons licence will apply to this Work are set out in our licence referred to above.

Other than as permitted in any relevant BMJ Author's Self Archiving Policies, I confirm this Work has not been accepted for publication elsewhere, is not being considered for publication elsewhere and does not duplicate material already published. I confirm all authors consent to publication of this Work and authorise the granting of this licence.

For peer review only

The attitudes, perceptions and experiences of medical school applicants following the closure of schools and cancellation of public examinations in 2020 due to the COVID-19 pandemic: a cross-sectional questionnaire study of UK medical applicants

Katherine Woolf [a,*]

k.woolf@ucl.ac.uk ORCID 0000- 0003-4915-0715

David Harrison [a]

david.harrison@ucl.ac.uk ORCID 0000- 0002-6639-6752

Chris McManus [a]

i.mcmanus@ucl.ac.uk ORCID 0000-0003-3510-4814

[a] Research Department of Medical Education, University College London Medical School, London WC1E 6BT, UK

* Author for correspondence: Dr Katherine Woolf Research Department of Medical Education, University College London Medical School, London WC1E 6BT, UK
k.woolf@ucl.ac.uk 020 3108 9216

For peer review only

Abstract

Objective

Describe the experiences and views of medical applicants from diverse social backgrounds following the closure of schools and universities and the cancellation of public examinations in the United Kingdom (UK) due to COVID-19/coronavirus.

Design

Cross-sectional questionnaire study, part of the longitudinal United Kingdom Medical Applicant Cohort Study (UKMACS).

Setting

UK medical school admissions in 2020.

Participants

2887 participants completed an online questionnaire 8th - 22nd April 2020. Eligible participants had registered to take the University Clinical Admissions Test (UCAT) in 2019 and agreed to be invited to take part, or had completed a previous UKMACS questionnaire, had been seriously considering applying to medicine in the UK for entry in 2020, and were UK residents.

Main outcome measures

Views on calculated grades, views on medical school admissions and teaching in 2020 and 2021, reported experiences of education during the national lockdown.

Results

Respondents were concerned about the calculated grades that replaced A-level examinations: female and Black Asian and Minority Ethnic (BAME) applicants felt teachers would find it difficult to grade and rank students accurately, and applicants from non-selective state schools and living in deprived areas had concerns about the standardisation process. Calculated grades were generally not considered fair enough to use in selection, but were considered fair enough to use in combination with other measures including interview and aptitude test scores. Respondents from non-selective state (public) schools reported less access to educational resources compared to private/selective school pupils, less online teaching in real time, and less time studying during lockdown.

Conclusions

The coronavirus pandemic has and will have significant and long term impacts on the selection, education and performance of our medical workforce. It is important that the views and experiences of applicants from diverse backgrounds are considered in decisions affecting their futures and the future of the profession.

For peer review only

For peer review only - http://bmjopen.bmj.com/site/about/guidelines.xhtml

Article summary: strengths and limitations of this study

- This is the first systematic exploration of medical applicant views on and experiences of the most significant changes to UK education in living memory due to the SARS-COV-2/COVID-19 pandemic.
- It is also the first study we are aware of that looked at university applicant views on calculated grades and the perceived impact on university admissions this year and in 2021.
- The large sample size gathered from around the UK, and the richness of the data provides insight into differences in the experiences and views of different socio-demographic groups, after controlling statistically for educational attainment.
- It is uncertain how representative our sample is of all medical applicants; medical applicants are not representative of all university applicants in either academic or socio-demographic terms and generalisations from our findings to all applicants should only be done with extreme caution.
- At the time of writing it was not possible to include data on participant examination scores or applications and acceptances to medical school; however this follow-up is planned.

Introduction

The UK Medical Applicant Cohort Study (UKMACS) is a study of United Kingdom (UK) medical school admissions. It is primarily a longitudinal questionnaire study of UK residents who in the summer and autumn of 2019 were seriously considering applying to study medicine in the UK for entry in 2020. UKMACS questionnaire data are subsequently linked to administrative data on all UK medical applicants held within the UK Medical Education Database (www.ukmed.ac.uk). Wave 1 data were collected between May and September 2019 and asked how applicants from different backgrounds were choosing which medical schools to apply to. Wave 2 data were collected from November 2019 to January 2020 and asked which medical schools and universities participants had applied to and how they had made their choices.

In March 2020 it was announced that UK schools would close and A-level (and equivalent public examinations) would be cancelled due to the coronavirus/COVID-19 outbreak in the UK. This was one of the most major disruptions ever to affect education and university admissions in the UK and was very significant for the UKMACS cohort, who are mostly in their final year of schooling and were due to sit examinations in the summer of 2020.

We therefore administered an additional unplanned UKMACS questionnaire to understand what medical applicants were experiencing in terms of education, their views on how grades would be awarded following examination cancellations, and their views on how medical schools might respond with regard to admissions policies. We particularly sought to understand how applicants from diverse social backgrounds might differ, with the aim of facilitating the inclusion of applicant perspectives and experiences in discussions about changes to medical school admissions and medical education.(1)

Calculated grades

The absence of A-levels and other equivalent public examinations in March 2020 meant that alternative methods of assessment for candidates had to be found, not least as A-levels are “the single most important bit of information [used in selection]” by universities.(2) On April 3rd *Ofqual* (Office of Qualifications and Examinations Regulation) in England announced that exams under its purview in England would be replaced by calculated grades based on teachers estimation of the

For peer review only - http://bmjopen.bmj.com/site/about/guidelines.xhtml

grades that their students would have attained and the ranking of each student within grades[1], which would then be standardised centrally.(3) The Scottish Qualification Authority (SQA) and other national bodies also announced similar processes for their examinations.

Performance in A-level examinations has long-term impacts (4, 5), which makes changes to how grades are awarded potentially very significant. The use of calculated grades raises many questions, some of which were summarised in a letter to *The Guardian* by Yasmin Hussein, a GCSE student who said that,

> *"… the … exam hall [is] a level playing field for all abilities, races and genders to get the grades they truly worked hard for and in true anonymity (as the examiners marking don't know you). [… Now we] are being given grades based on mere predictions." Yasmin Hussein, letter to The Guardian, March 29th 2020.(6)*

Among teachers, survey data suggests that there are doubts about the accuracy and fairness of calculated grades, with 39% saying that all students would get a fair deal, 24% saying they would not, and 37% not knowing or not answering. There were also doubts about fairness for students from Black Asian and Minority Ethnic (BAME) backgrounds, about those working hard in the last weeks before an exam being penalised, about teacher 'favouritism', although there were teachers who commented that the process is as fair as possible under the circumstances.(7)

University applicants also have concerns. In a survey carried out by HEPI (Higher Education Policy Institute) before the details of calculated grades were announced, but after it was known that grades would in some way be predicted, 27% thought that their predicted grades were worse than they were likely actually to have attained, compared with 13% thinking their predicted grades were better than they would actually attain.(8)

Another survey of 511 university applicants (including 452 A-level students) conducted for the Sutton Trust found that just under half believed the new A-level grading system would result in their receiving poorer grades but working class respondents were more worried about large negative consequences compared to middle class students. Nearly three quarters believed the new system was less fair than examination grades and this was more of a concern for applicants from *higher* socioeconomic backgrounds. Nearly half of applicants felt the COVID-19 crisis would impede their chances of getting into their first choice university, a more common concern among working class respondents.(9)

The impact on medical school admissions of examination cancellations and their replacement with calculated grades is, at the time of writing, still not completely clear. *Ofqual* states that,

> *"The grades awarded to students will have equal status to the grades awarded in other years and should be treated in this way by universities, colleges and employers. On the results slips and certificates, grades will be reported in the same way as in previous years".(3), p.6.*

The decisions of *Ofqual* in this case are in effect governmental decrees, supported by Ministerial statement, and universities and other bodies will therefore abide by them, as was affirmed by the Medical Schools Council on 5th May 2020.(10) That does not mean however that other factors may not need to be taken into account in some cases, as for instance when applicants do not attain the grades needed for their conditional offers, or for applicants in clearing. Furthermore in guidance

---

[1] For example, if a teacher has 30 Chemistry A-level students, they would estimate the grade each student would get. Then the teachers ranks students within grades, so for example if they have 5 students estimated to get an A grade, they rank those 5 students.

For peer review only - http://bmjopen.bmj.com/site/about/guidelines.xhtml

updated on 1st May 2020 the Government stated that "if a student does not feel their grade reflects their performance, they will have the opportunity to take an exam in the autumn"(11) with *Ofqual* expanding on 15th May 2020 that "students will be able to use the higher of the two grades for future progression."(3) This raises questions for university admissions, as Medical Schools Council acknowledged in their statement of 5th May 2020:

> *"There are a number of issues that the education sector as a whole is yet to resolve. These include how appeals against calculated grades will work across the UK and when students will be able to sit exams if they are unhappy with their calculated grade. The impact of these issues on medical admissions is unclear but medical schools are actively engaging in these discussions and are working hard to develop solutions that are fair to applicants."(10)*

Education during the pandemic

As well as examinations being cancelled, UK schools closed on 20th March 2020 to all except the children of key workers and vulnerable children with secondary schools mostly closed until September 2020. Similarly in mid-March 2020 many universities suspended face-to-face teaching for the academic year 2019/2020.

The impact of school closures on student learning and outcomes will be significant (12-14) and it may be particularly problematic for those from poorer backgrounds and/or at state-funded schools. The Institute of Fiscal Studies analysed survey data from a weighted sample of over 4000 parents with children aged between four and 15 years old in May 2020 (15). Among secondary school children, those from the richest quintile were spending on average slightly over an hour more per day on learning compared to those in the poorest quintile, amounting to several weeks more learning over the course of the time schools are closed. In particular children in the richest families were spending significantly more on educational activities provided by schools and from private tutors. Even among state school pupils, children from the richest families reported greater access to face-to-face online teaching, which the authors argue is likely to be of higher educational value than other resources that require more parent input, particularly since the poorest parents of secondary school children were less likely to find it easy to support their child's home learning.

The results of the IFS report chime with data from *Teacher Tapp*, an ongoing weighted survey of several thousand teachers in England.(16) At the start of the lockdown (23rd March 2020) private school secondary schools were much more likely than state secondary schools to be using online videoconferencing (27% vs 2%) and online chat (18% vs 3%). The above-mentioned Sutton Trust report (9) also found socioeconomic differences in access to "internet access, devices for learning or a suitable place to study" and differences in the amount of A-level teaching being conducted by teachers at private and state schools.

Among those secondary school pupils who had applied to university, the Sutton Trust report authors argued that students from lower socio-economic backgrounds are also likely face additional disadvantages both with their university applications and when starting university:

> *"Given the uncertainty caused by these changes [to education resulting from COVID-19], university applicants are likely to need more support than ever to navigate the process [of applying to university]. This will be even more important for young people from lower socio-economic backgrounds, who are less likely to be able to draw on the advice of family members with higher education experience themselves. But with schools closed for most pupils, it may be difficult*

For peer review only - http://bmjopen.bmj.com/site/about/guidelines.xhtml

for applicants to get the help they need. Similarly, there's also a danger that this year's applicants will miss out on A level content during the lockdown […]. For disadvantaged students about to go on to higher education, this could leave them with gaps in their knowledge base, putting them behind their peers before they have even begun at university." [p1. (9)]

The present study

This study aimed to explore and describe perceptions of calculated grades, of student selection more generally, and of educational experiences during school and university closures, in a large group of medical school applicants, who were typically high-attaining students. A range of background factors were assessed to determine how perceptions differed according to demographic and other measures. Data collection took place between April 8th and April 22nd, which was about two and a half weeks after school closures.

Methods

Study design

Cross-sectional questionnaire study, which formed part of the longitudinal UK Medical Applicant Cohort Study.

Eligibility

To be invited to complete the questionnaire, participants had to have registered to take the University Clinical Admissions Test (UCAT) in 2019 and to have agreed to be invited to take part in UKMACS, or they needed to have completed one or more previous UKMACS questionnaires[2]. They also need to have been seriously considering[3] applying to study medicine in the UK for entry in 2020, and be resident in the UK or Islands/Crown Dependencies.

Participants were not invited if they had previously requested their data be removed from the UKMACS database, had asked not to be contacted for further research, or had not consented to having their personal information retained by the research team or linked with other information for research purposes.

Questionnaire development

During the development of the questionnaire *Ofqual* announced that calculated grades would be awarded. We therefore assessed perceptions of how calculated grades would be awarded and used, and of other possible methods medical schools could use to select or reject offer-holders. We also about potential knock-on effects of calculated grades in the 2021 application cycle, and whether medical schools should open online or defer opening until teaching could be done face-to-face. We asked about use of educational resources and preparation for university/medical school, and about the time they were spending on various activities. We included self-reported measures of academic attainment and socio-demographic measures used in previous UKMACS questionnaires, as well as the 15-item Big Five personality measure used in the national longitudinal cohort study *Understanding Society*. (17) Personality traits are "relatively enduring styles of thinking, feeling, and

[2] Wave 1 of the UKMACS questionnaire was administered between May and October 2019; Wave 2 between November 2019 and January 2020.
[3] Participants were thought to be seriously considering applying if they had registered to take UCAT. Wave 1 of the questionnaire also asked them to confirm they were seriously considering applying to study medicine.

For peer review only - http://bmjopen.bmj.com/site/about/guidelines.xhtml

acting".(18) It is generally agreed that there are five distinct personal traits or factors: Neuroticism, Extraversion, Openness to Experience, Agreeableness, and Conscientiousness.

Most questions were designed specifically for this questionnaire since they asked about unprecedented events and validated items were not available. We constructed the questionnaire with JISC Online Surveys [https://www.onlinesurveys.ac.uk/] and piloted the questionnaire and information sheet with two current applicants to medical school. Amendments were made in response to feedback from the applicants and from Medical Schools Council.

Questionnaire administration

Participants were sent an email invitation and link to the current questionnaire on the afternoon of 8th April 2020. 18,665 invitations were sent, with up to two email reminders and two text message reminders. The questionnaire closing date was 20th April 2020, with responses accepted up to 22nd April 2020.

Statistical analysis

Descriptive and univariate analyses were performed in SPSS v26. Imputation of missing data and multivariate analyses were performed in R.

Factor analysis on the 87 attitudinal variables was carried out using the *psych* package in R (21) with *fa.parallel()* and *nfactors(),* being used to determine the number of factors.

Freetext question answers

All answers to freetext questions were read by the research team, and illustrative quotes selected to aid understanding of quantitative results.

Patient and public involvement

Patients and the public were consulted in the development of the questionnaire.

Results

Participants

3071 participants completed the questionnaire, of whom 2904 stated they were eligible to take part (i.e. seriously considering applying to study medicine in the UK in 2020 and resident in the UK or Islands/Crown Dependencies). After removing 16 respondents who did not consent to have their data analysed and 11 duplicates, there were 2877 valid cases for analysis, which is 15% of those invited. This is subsequently referred to as the full sample.

The main analyses were performed on a restricted sample of 1562 respondents currently in Year 13, who had applied to medicine for entry in 2020, with at least three predicted A-levels and no achieved A-levels. Results are also reported in the Supplementary files for respondent groups excluded from the restricted sample, notably those living in Scotland and those not currently in Year 13 – see Supplementary file.

Missing data

The analysis considered 120 measures in the restricted sample. The questionnaire asked about attitudes to 87 different topics concerning medical school entrance. Of 153,076 data points, 10788 (7.2%) were missing. For the individual variables, the median percentage of missing data values was

For peer review only - http://bmjopen.bmj.com/site/about/guidelines.xhtml

0.48%, with 75 measures having fewer than 5% of missing values. The questionnaire also asked about demographic and educational items. For 12 demographic measures, 462 of 18744 measures were missing (2.5%), with a median of 1.0% per measure, and 11 measures having fewer than 5% missing values. For further details on missing data, please see the Supplementary file.

Demographics

Demographics for the full and restricted samples are reported in **Table 1**, where details of categories within demographic variables can also be found.

Education and achievement

Predicted A-levels

A-level grades were scored as A*=12, A=10, B=8 etc, and those reported as being between two grades as intermediate e.g. A*/A =11, A/B = 9, etc. Mean predicted A-level grades were calculated for the top three grades regardless of subject (*Mean top three predicted* A-levels), and for all grades (*Mean predicted* A-levels). *Mean top three predicted* A-levels was 10.89 and *Mean predicted* A-levels was 10.71.

UCAT, BMAT, GAMSAT

1546 participants (99.1%) reported having taken UCAT; 765 (49.0%) reported having taken BMAT; and none reported having taken GAMSAT. Of the 1350 participants who reported a total UCAT score that was greater than 1799 and less than 3601, the mean score was 2660 (SD=235).

GCSE

GCSE grades can range from 1 to 9. A variable *Mean GCSE* was calculated by dividing the total GCSE points by the number of GCSEs taken, and the mean was 7.91 (SD=0.71).

Relationships between educational measures

UCAT score correlated with *Mean top three predicted* A-levels at 0.418 (p<.001) and with *Mean GCSE* at 0.487 (p<.001). *Mean GCSE* and *Mean top three predicted* A-levels correlated at 0.611 (p<.001).

Participants at non-selective state schools had lower scores on all attainment measures (*Mean GCSE*: difference=0.3 points, p<.001; *Mean top three predicted* A-levels: difference=0.23 points, p<.001; UCAT score: difference=89 points, p<.001).

Medical school offers

1292 (85%) respondents had applied to four medical courses, 1289 (82.5%) had at least one offer, 177 (11.3%) had four offers, and 204 (13%) were waiting to hear from at least one medical school at the time of completing the questionnaire.

Respondents who did not have a parent/carer with a university degree were less likely to have a medical offer (78.1% vs 85.0%; p=0.001), as were those without a parent/carer in the highest socioeconomic group (79% vs 85%; p=0.002) Male participants were slightly less likely to have an offer (80% vs 85%; p=0.049).

For peer review only - http://bmjopen.bmj.com/site/about/guidelines.xhtml

Applicant views on admissions

Perceptions of the fairness of methods medical schools could consider using in the selection of offer-holders

Participants were asked to rate the fairness of 17 measures, including calculated grades, that medical schools could potentially use to decide to accept or reject offer-holder following exam cancellations. Rating categories were: "Unfair: should not be used" "Quite unfair: avoid if possible" "Quite fair: could be used in combination with other measures" "Very fair: could be used alone", with a freetext question asking for additional comments and suggestion.

No measure was felt by a majority of participants to be fair enough to use on its own. The measure considered most fair was *Exam grades taken in September 2020 (if these take place)* (32.3% very fair), followed by *Predicted Grades declared on UCAS application* (26.2% very fair), *Calculated grades* (22.6% very fair), *GCSE grades* (20.4% very fair) and *Score at interview* (19.5% very fair).[4]

Several methods were felt by a majority to be fair enough in combination, particularly *Predicted grades* (80.6%), *GCSE grades* (73.8%), and *Score at interview* (73.4%); but only a fifth (20.3%) of participants felt *Attendance at widening participation activities* was quite fair or very fair. See Figure 1.

Multiple regression results showed that after taking account of all other educational and socio-demographic variables, BAME participants were more likely to perceive *Exams taken in September 2020*, *UCAS personal statement*, and *Personal background* as fair to use, and respondents from deprived areas were more likely to perceive *Personal background* and *Attendance at widening access programmes* as fair to use. *Calculated grades based on mock exams, coursework etc, and awarded in place of final examination grades* were perceived as less fair by those with lower predicted A-levels.

There were 154 freetext responses (10%), with participants elaborating on their responses or suggesting alternatives:

> *"A combination of the most objective information that every offer holder will have, ie GCSEs, UCAT or BMAT, interview score, etc"*

> *"A standardised form of assessing all medical applicants would be the best way to allocate existing places. [...] Since we do not have standardised A level grades, places should be offered using the UCAT as this is the fairest way of distributing places to the most able students."*

> *"Using interview scores and UCAT scores in combination are independent measures, and are more fair than using calculated grades which have the potential to be biased."*

> *"Anything including personal statement, BMAT or UCAT I would argue are unfair to use as judgement as there will definitely be a bias in terms of how certain students achieved their grade. I believe the fairest way to determine ones overall*

[4] See Figure 1 for full item wording

For peer review only - http://bmjopen.bmj.com/site/about/guidelines.xhtml

grade would be to use their GCSE data with a combination of evidence throughout the two years of A levels."

Other measures participants mentioned included: an additional university assessment (written, viva or project/portfolio-based) now or at the start of the academic year, an additional interview, selection at the end of Year 1/make first year a foundation year, additional reference from teachers/school, reference from work experience, school/college attendance record, distance from university, extenuating circumstances, self-reported use of time during quarantine/lockdown, number of offers received, prioritise those with higher degrees, prioritise those already working in the NHS, extra-curricular achievement (e.g. music, Duke of Edinburgh's Award), school's prior achievement. For example:

"NHS experience ie patient facing health professional ie years and grade, other non technical skills, education background ie. science, post graduate achievement ie MSc particularly if in science or medical subject and grade achieved. Also emphasis on the candidates as a whole ie well rounded personality (potential to communicate well) rather than typical A Grade student. Letter of recommendations from medical consultant whom candidates may have worked closely with."

"Another interview possibly over the phone to see what students have done with their time in quarantine (ie, volunteering in a care setting or hospital / working in a hospital / exploring other interests)"

"Each university could form their own selection test similar to UCAT/BMAT with a brief guidance/specification on what will be on the test given out to offer holders so they have some time to revise for it, but this should be used in combination with other details (e.g. if offer holder's calculated grade was only 1 grade below what was required for entry)"

"I think a combination of previous results, any exams that do go ahead (at some point whether that is this summer or later), alongside medical applications, relevant work experience (as per personal statement and any other forms detailing this) and the applicant interview. Also potentially the medical schools could generate online admissions tests for students with conditional offers to generate a clearer view of a students capability and ability to comprehend and withstand the pressures of medical school. But any tests generated by the medical schools must be used alongside the other parts of the applications to ensure fairness."

Participants were asked whether they had heard anything from medical schools/universities they had applied to about how selection might be impacted by examination cancellations; among those holding conditional offers, a minority (n=538; 42%) said they had heard from at least one medical school/university they had applied to.

Acceptability of options for dealing with a situation in which more students meet their offers than there are medical school places

Participants were asked to rate the acceptability ("completely unacceptable", "slightly unacceptable", "neutral", "slightly acceptable", "completely acceptable") of a number of options

For peer review only - http://bmjopen.bmj.com/site/about/guidelines.xhtml

that medical schools could use if they had more students meeting offers than they had places, with a freetext question asking for additional comments and suggestions.

The most acceptable option was *Ask some applicants with offers to volunteer to defer a year*. The only other acceptable option was *Accept all applicants whose calculated grades meet the conditional offer, although it could mean fewer resources per student*. See **Figure 2**.

Multiple regression analyses showed no significant differences by social or demographic group on these items.

There were 187 freetext responses (12%). Several respondents suggested that medical schools should receive more funding to manage larger cohorts and create more doctors, e.g.:

> *"Deferring of one year should not be taken into consideration as this would damage applications of next year. Ask the government to invest more money on the NHS and allow to have more spaces. All these problems could be solved if exams were taken virtually."*

> *"The government could also provide more funding for medical schools- not only will this allow more people to attend but it will also mean there are more doctors down the line who can work in the NHS."*

There were suggestions that applicants could opt to attend other medical schools they had applied to but which they had not selected as their firm or insurance choice, or that they could be offered places at medical schools they had not applied to:

> *"If some medical schools have a lower numbers of applicants overall, compared to others, redistribute some students to these ones, with permission."*

There were many suggestions of incentives to defer, and some felt that they would welcome a year off before starting:

> *"Incentives to defer like 1 yr free accommodation or £5000 or student ambassador job for gap year"*

> *"Incentive to deferring such as free university accommodation for the first year, organised work experience placements and or organised care assistant jobs for the gap year."*

> *"If people are asked to volunteer to or forcefully defer entry, offering alternatives for work they could do within a healthcare setting for that year. For example, maybe clerical work within the NHS so they're still immersed within the healthcare system.*

> *"Asking students to voluntarily defer a year would be a popular option, I think many people will reevaluate their priorities over the coming months and may appreciate the opportunity."*

> *"The option to defer is definitely an option that should be considered as many people would be happy with the idea of gaining more medical experience in the year out that they would now have."*

For peer review only - http://bmjopen.bmj.com/site/about/guidelines.xhtml

There were suggestions medical schools could have multiple cohorts either all starting in October or one cohort starting in October and another cohort starting early 2021.

> *"Create an extra group/year for Covid Students to manage the numbers"*

> *"Maybe consider having staggered starts throughout the year October start January start June starts."*

> *"Stagger the course to offer two presentations and alter the following academic term holidays if possible"*

Respondents also expressed concern as to the impact of the present disruption on next year's admissions cycle and available resources:

> *"The selection process should not be biased towards those rejected this year, next year, and should not change for the next cohort."*

> *"I hope that this year's or next year's applicants will not be disadvantaged due to these unprecedented circumstances."*

Perceptions of potential impact on admissions for 2021

Participants were asked to rate how much they agreed or disagreed with six options as to how medical schools could deal with the potential impact of the current situation on admissions in 2021. See **Figure 3**.

In general, respondents felt medical schools should give special consideration to current applicants reapplying next year (67.1% agreed/strongly agreed that *Applicants rejected this year should be given special consideration when re-applying next year*) however opinions were divided about what that special consideration should consist of.

Multiple regression analyses showed that after accounting for number of offers, educational, social and demographic factors, BAME respondents were more likely to feel that re-applicants should be given some advantages.

Starting academic year 2020/2021

A majority of respondents (n=952, 61.1%) believed that if necessary, medical schools should *Defer the start of the academic year only when face-to-face teaching is possible* with 605 respondents (38.9%) believing that medical schools should *Start the academic year on time using distance learning for as long as is necessary*. This did not vary significantly by prior attainment, number of offers, or educational/social/demographic background.

Education and university preparation

Calculated grades and the perceptions of process of awarding calculated grades in lieu of examination grades

Participants were generally fairly ambivalent towards calculated grades. On the positive side (see **Figure 45 Error! Reference source not found.a**), the majority of respondents (78.6%) preferred calculated grades to taking examinations next year, and about half (54.9%) preferred calculated grades to taking examinations in September 2020. Over half (59.3%) agreed that schools wouldn't be able to game the process to award all their students high grades, and 51.4% felt that the process of awarding calculated grades was the best way to be fair to most students in the circumstances

For peer review only - http://bmjopen.bmj.com/site/about/guidelines.xhtml

(although 35.0% disagreed). Over half (56.4%) agreed that their teachers were generally able to rank and grade students accurately, however respondents were divided as to whether their own teachers knew them well enough to grade and rank them accurately: 42.0% agreed their teachers did NOT know them well enough whereas 44.6% thought their teachers DID know them well enough.

On the negative side (see **Figure 5 b**), over half of respondents (52.9%) disagreed or strongly disagreed that calculated grades would result in an accurate assessment of their abilities, with 63.4% agreeing that teachers would find it hard to be unbiased, 80.7% agreeing it was difficult to see how teachers in large schools can rank so many students and 85.5% agreeing calculated grades cannot take into account students doing better in exams than their teachers expected. Most agreed it was unfair to judge students on work done since schools/colleges closed (70.4%), that grades should be based solely on their performance and not the performance of previous students at their school (69.6%), and that it was unfair their GCSE performance was not taken into account (68.7%).

Mean top three predicted A-level points was a major predictor of perceptions of calculated grades but there were also differences by background after accounting for prior attainment, number of offers and other educational/social/demographic factors: BAME respondents and female respondents were more negative about calculated grades and respondents from non-selective state schools and those from more deprived areas were more likely to agree that calculated grades should not take into account the performance of previous pupils at their school. See **Table 2**.

There were 398 freetext responses to the following request for further comments at the end of the questionnaire: "Please use this space for any additional comments you wish to make about the questionnaire or selection of medical students". These responses included concerns that calculated grades would be based on work completed early in the academic year and on mock exams created and assessed by the school. It was felt that these measures would not take into consideration the development and academic progress made by pupils over the year, even when teachers gave special consideration to the impact of the disruption. There was also concern that at the time of mock exams in particular, many medicine applicants were more focused on admissions tests (BMAT in particular), submitting applications and preparing for interviews.

> *"Grade calculations took away the chance the students had to prove themselves (final exams) and their control. Basing the final grade on a time when the students weren't aware that they were being truly assessed can hardly be classed as fair."*

> *"I believe universities should be lenient and realise that if a students calculated grade is below their conditional offer, this is not 100% representative of the students abilities. If they were able to secure an offer in the first place then universities should already know the academic capabilities of said student through their GCSE grades, predicted grades, UCAT/BMAT scores, teacher references, interviews etc. Otherwise, they wouldn't have given the student an offer. Where possible, every offer holder should be given their place at university in this academic year, whenever it resumes and should not be forced to take a year out and spend that year being stressed, lost and demotivated."*

With teacher submitted grades then being subject to standardisation by the exam boards based on previous achievement from a school was a concern for this student:

For peer review only - http://bmjopen.bmj.com/site/about/guidelines.xhtml

"I am the only student in my year and the third student in my sixth form's history to ever apply for medicine, and the first to receive all 5 offers. My school historically is one that does not do very well and I fear that my individual success and all the hard work I have had to do on my own as I get no help from my school, will be overshadowed by the bad results from previous years."

Education since the shutdown

A minority of respondents said their school was planning on formally assessing them on work done since the shutdown (n=184; 11.8%); nearly half (n=740; 47.5%) said their school would not, and over a third (n=614; 39.4%) were uncertain. Respondents attending a private/selective school were twice as likely to report being assessed on work since the shutdown (14.2% vs 7.6%; p<.001).

Participants were asked whether they were using educational resources provided by their school/college and if not why not. Nearly all respondents had used at least one resource (n=1346; 91%) and three was the average number used.

Respondents attending private/selective schools were more likely to report having used all educational resources except support for university applications, and those at non-selective state schools used on average two resources compared to the three used by those at private/selective schools. The largest difference was in the use of online teaching in real time, which those at private/selective schools were nearly four times more likely to have used. See **Table 3**.

In the multivariate analyses, attendance at a private/selective school was an independent predictor of using online teaching in real time, online resources for home learning, online formative assessments, and paper resources for home learning, even after controlling for prior attainment and socio-demographics. In addition, having at least one parent/carer with a university degree was an independent predictor of using paper resources for home learning, and having lower UCAT/BMAT scores was an independent predictor of using online teaching in real time.

Those who had not used educational resources reported the main reason(s) were either that the resources were not available or that they felt they did not need to use them. Only very few said they had not used a resource because of a lack of private quiet space, lack of time, lack of internet/computer access, or because they were finding it too hard to focus. Those at non-selective state schools were more likely than those at private/selective schools to state lack of availability as a reason, and less likely to state not needing to as a reason– see **Table 4**.

Preparation for medical school/university

Participants were asked what preparation if any they were doing for university or medical school – see **Figure 5**.

Of the 207 (13.3% of the sample) who said they were not doing any preparation, the most common reason was that they were too worried and couldn't focus (n=88; 42.5% of those not doing any preparation), not having resources (35.5%), feeling it wasn't necessary (29.5%), caring for others (13.5%), not going to university this year (14.0%), not having time (6.3%), and being unwell (4.8%). Respondents could select multiple reasons.

For peer review only - http://bmjopen.bmj.com/site/about/guidelines.xhtml

Time spent during the lockdown

Participants were asked to state how much time they were spending on various activities in the previous five days – see **Figure 6**. The multivariate analysis showed that respondents from private/selective schools reported spending more time studying, even after controlling for prior attainment and socio-demographic factors.

Factor analysis

Number of factors

The factor analysis included 87 variables which are attitudinal or related to attitudes. The maximum eigenvalue was 6.99, with 27 eigenvalues greater than 1. A scree-plot suggested that there was a break at or around 6 factors (see **Figure 7**). Other criteria were very variable, with *fa.parallel()* in the *psych* package in *R* suggesting there were 19 principal components with eigenvalues greater than the 95% upper confidence interval for randomly generated data. *nfactors()* in *psych* said that VSS complexity 1 suggested 17 factors, VSS complexity 2 suggested 17 factors, Velicer's MAP gve 10 factors, Empirical BIC gave 20 factors, and Sample Size Adjusted BIC gave 20 factors. However the output also reported, "Although the vss.max shows 17 factors, it is probably more reasonable to think about 4 factors". Overall there are probably many small factors corresponding to measures with low communalities and hence mostly unique variance. For present purposes we are particularly interested in aggregating measures to gain more statistical power, and therefore we chose to extract 6 principal factors, which corresponds with the break in the scree slope, and is a little larger than the *nfactors*() recommendation of 4.

Naming of factors

The six factors were named as following, by considering the highest absolute loadings, along with all loadings over 0.35:

1 '*Lack of confidence in calculated grades*'. Positive loadings (n=9 items) reflected concerns that teachers will not know students well enough and will find it hard to be objective, preferring not to have calculated grades and take exams in September or next summer, and appeals being unlikely to be successful. Negative loadings (n=5 items) reported confidence in the process resulting in an accurate reflection of a student's true ability, and the awarding process being fair to most students. High positive scores therefore represent **a lack of confidence in the process of determining calculated grades**.

2 '*Special treatment next year for rejected applicants*'. High positive loadings (n=6 items) were associated with medical schools needing to give higher priority and special consideration next year to students rejected this year, with rejected candidates being automatically given conditional offers next year. Negative loadings (n=4 items) suggested that re-applicants next year should be treated in the usual way, and special treatment for rejected applicants this year would not be fair for first year applicants next year. High positive scores therefore suggest that **applicants who are rejected this year should be treated specially next year**.

3 '*Other selection measures to be taken into account*'. A small group of items (n=3) suggested that selection could take into account aptitude tests such as UCAT, BMAT, and performance at interviews. High scores therefore suggest that where possible, **measures other than calculated grades should be taken into account**.

For peer review only - http://bmjopen.bmj.com/site/about/guidelines.xhtml

4 '*Preparing for medical school*'. High positive loadings (n=4 items) reflected applicants who during lockdown were preparing for university by reading (either textbooks or other books), were watching online lectures, as well as talking with friends. Negative loadings (n=4 items) reflected applicants who were not doing any preparation, didn't feel preparation was necessary, didn't have any resources, or who couldn't focus because they were too worried. High scores therefore indicate an applicant's **concentration on preparing for medical school or university**.

5 '*Importance of background and experience*'. All high loadings (n=8 items) were positive and indicated that medical schools should take into account work experience, the applicant's personal statement, and the teacher's reference on the UCAS form, attendance at university summer schools and widening participation programmes, an applicant's personal background such as being from under-represented groups, and other grades in qualifications such as GCSEs and the Extended Project Qualifications. Overall higher scores indicate that **a wider range of measures should be used to take into account personal background and wider experience.**

6 '*Resources from school for home study*'. All loadings were positive (n=8 items), and indicated that applicants were being provided with live online teaching, online resources for home learning, paper resources such as workbooks, formative online assessments, and summative online assessments that might count towards calculated grades, doing timed essays or past papers, and spending more time studying. Higher scores therefore indicate **having received greater support for home schooling from schools and colleges**.

Predictors of factor scores

Predictors of factor scores were assessed using multiple regression. All predictor variables in the set were entered and only those achieving p<.01 are reported. All predictors therefore take into account the effects of others in the set. Set A is the basic set used earlier in the study. Set B is extended by including socioeconomic group (based on parents' jobs), doctor parent(s) and the five Big Five personality factors, and are included on an exploratory basis. See **Table5.**

Summary and conclusions

Summary of results

No single measure, including calculated grades, was considered fair enough by most applicants to use in the acceptance or rejection of offer-holders; however many applicants considered calculated grades – and many other measures - fair enough to use in combination with other measures such as interview scores or admission test scores. Taking into account personal background or widening participation attendance was considered fairer by BAME applicants, those from deprived areas, and those without degree-educated parents.

Many respondents had concerns about calculated grades, especially BAME and female applicants who felt teachers would find it difficult to grade and rank students accurately, and those from non-selective state schools and living in deprived areas were more concerned about the standardisation process that uses the attainment of previous pupils at a school. Despite this, the majority would rather have calculated grades than forgo calculated grades completely and take examinations in Autumn 2020 or Summer 2021 instead.

Respondents mostly felt that medical schools should admit any applicant who met their conditional offer, even if that meant having to increase the number of places (which would require a legal change and increased government funding), although there was also acceptance of medical schools asking for volunteers to defer but not of requiring deferrals. Respondents were divided as to how

For peer review only - http://bmjopen.bmj.com/site/about/guidelines.xhtml

rejected applicants should be treated if they were to reapply next year, with some respondents feeling they should be treated no differently and others feeling their 2020 experience should be taken into account. A majority of respondents tended to favour medical schools delaying the start of term until face-to-face teaching were possible.

Applicants from non-selective state schools reported using fewer educational resources than their counterparts at private or selective schools, and in particular they reporting less online teaching in real time, and spending less time studying during the lockdown.

Comparisons with other research

Our findings show many similarities to other recent UK studies of the effects of the COVID-19 pandemic on education in the UK (8, 9, 15, 16) however it is notable that in this sample of medical applicants ethnicity is more significant than socioeconomic factors in predicting concerns about calculated grades. A study of A-level students, conducted by Bhopal and Myers between April and August 2020 and published as a report on the OSF open access repository, surveyed an ethnically diverse sample of 583 A-level students in Britain and interviewed 53 students about their views on their education during the pandemic and their exam results. The authors report that 21% of students were glad exams had been cancelled but over twice as many (46%) would prefer to sit exams, which is similar to our finding that exams were considered the fairest method of selection. Similarly to our findings, the authors report that “Many students also raised concerns their ethnicity could influence how teachers assessed their work” quoting a Black student saying “Some of my teachers seem biased […] They always think the Black boys are trouble”, an Irish Traveller student saying “We’re Travellers. The school doesn’t think much of us.” and an Indian student saying “My teachers don’t think I can do that well […] They also have their favourites, we can all see that – those students who they think should do well, are not those who necessarily will do well”. This reflects concerns from the Black Asian and Minority Ethnic participants in our study about teacher bias.
.

It is known that predicted grades are lower for some minority ethnic groups (22) and indeed, on 2nd April 2020 after the announcement of the cancellation of examinations but before *Ofqual* specified details of calculated grades, the Runnymede Trust and several other race equality organisations wrote to the Secretary of State for Education to urge him to “ensure a fair, transparent and robust system which will more accurately reflect the ability and attainment of students from different backgrounds”.(23) Subsequently, on 30th April, the Equality and Human Rights Commission said that,

> *“Using predicted grades in place of this year’s summer assessments could deepen the existing inequality in education and put the future of disadvantaged young people at risk if not correctly implemented”* (24)

Our finding that students from private/selective schools were using more educational measures - especially online teaching in real time, which requires significant teacher input and which Andrew et al (15) argue is higher quality that other types of resource - reflects findings from those authors’ research with parents of secondary school children (15) and teachers (16); however in our sample students’ use of educational resources and time spent studying did not vary by socioeconomic background, including parental higher education, socioeconomic status, or area deprivation. This may be a feature of this particularly high-achieving sample of medical applicants.

Strengths and limitations

This study is, to our knowledge, the first systematic exploration of medical applicant views on and experiences of the most significant changes to UK education in living memory. It is also the first

For peer review only - http://bmjopen.bmj.com/site/about/guidelines.xhtml

study we are aware of that looked at university applicant views on calculated grades and the impact on university admissions. The large sample size gathered from around the UK, and the richness of the data allowed us to examine important differences in the experiences and views of different socio-demographic groups, after controlling statistically for educational attainment.

The speed at which we were required to develop the questionnaire and the unprecedented nature of the topic under investigation meant we were unable to use validated measures for most questions, nor have we been able to validate the measures ourselves, although we were able to pilot them with current applicants. Our data provide a snapshot of applicant views and experiences in April 2020, and it is likely that participants' views and experiences changed after data collection. The fact that participants are part of a longitudinal study however means we will have the chance to follow up participants in 2021 and beyond to discover how the pandemic affected their education. It is uncertain how representative our sample is of all medical applicants. Data on applications, offers, acceptances and academic achievement from the current UCAS cycle are not released until early 2021, but it is very likely that offer-holders were over-represented in our sample. Data from the 2019 UCAT testing cycle also show that our sample scored higher than the mean [https://www.ucat.ac.uk/media/1329/2019-test-statistics-oct-2019.pdf]; however not all UCAT test-takers apply to medicine. Demographic data on 2020 medical applicants released by UCAS in November 2019 showed that our restricted sample was similar to all English applicants aged 17 to 19 in terms of ethnicity and deprivation but had more women [https://www.ucas.com/data-and-analysis/undergraduate-statistics-and-reports/ucas-undergraduate-releases/applicant-releases-2020/2020-cycle-applicant-figures-15-october-deadline].

Medical applicants are not representative of all university applicants in either academic or socio-demographic terms; however the similarity of some of our findings to that of other research, for example that private school pupils are receiving significantly more education than non-selective state school pupils, suggests that the views and experiences of our sample may not be completely different from those of university applicants more generally; however generalisations from our findings to all applicants should only be done with caution.

Implications for policy and practice

The impact of calculated grades on medical admissions was, at the time of writing, uncertain. Our questionnaire closed on 22nd April and on 5th May 2020 the Medical Schools Council announced that medical schools would honour all offers met (something not clear at the time of our questionnaire), while acknowledging that there were still a number of issues that needed resolving.

How calculated grades are likely to work in practice has also been explored by a parallel analysis by our team using UKMED data over the last ten years, comparing predicted A-level grades (which are likely to be similar to calculated grades) with actual, attained A-level grades.(25) Predicted grades were systematically higher in medical school applicants than eventual achieved grades. In addition predicted grades only predicted outcomes about two-thirds as well as achieved A-level grades, both in terms of outcomes five or six years later at the end of medical school, and seven or eight years later in postgraduate examinations. The under-prediction by predicted grades was mitigated in part, although not entirely, by combining predicted grades with UCAT/BMAT scores, which supports the views of some applicants that other measures might be used for selection amongst applicants not meeting the terms of conditional offers.

The likely impacts on medical schools of using calculated grades were at the time of writing uncertain, but our estimates suggested there could in effect be a lowering of entry grade requirements, with possible subsequent increases in medical school drop-out rates, and a somewhat

For peer review only - http://bmjopen.bmj.com/site/about/guidelines.xhtml

academically weaker cohort with poorer performance in medical school and postgraduate examinations.(5, 26) That is potentially important since very poor postgraduate examination performance itself strongly predicts being sanctioned by the medical regulator.(27)

In the awarding of calculated grades, we predicted that the raw 'centre assessment grades' and rankings produced by teachers for *Ofqual* were likely to be similar to predicted grades in being more generous than achieved A-level grades would have been, although the standardisation to be used by examination boards and *Ofqual* are likely to minimise that effect, so that distributions of calculated grades within subjects and centres become similar to actual A-level grades in previous years. As it transpired the centre assessment grades ended up being used without adjustment, and these were significantly higher than previous years' A-level grades, with the Education Datalab stating "At grades A*-A, there was an increase from 25.2% to 38.1%" (see https://ffteducationdatalab.org.uk/2020/08/gcse-and-a-level-results-2020-how-grades-have-changed-in-every-subject/).

As a result of the awarding of calculated grades an excess of candidates met their conditional offers[5]. Giving their views on what should happen in this regard, applicants in our study suggested that that in light of the shortage of doctors,(28) medical schools might argue for increased places and funding. In the event the Government did indeed lift the cap on medical school places to accommodate the increase in students (see https://www.gov.uk/government/news/action-agreed-to-support-students-into-preferred-universities). The impact of large increases in number on teaching and on predicting through to numbers of places for clinical teaching, foundation training and so on is still uncertain. It is worth considering that cohort sizes at many medical schools are already very large, that students tend to be less satisfied at larger schools,(29) and that accommodating extra students into face-to-face teaching that is COVID-secure is likely to be extremely challenging. On the other hand, there is a clear need for more doctors and it is likely that the change to admissions will result in a more socially and demographically diverse cohort.

In this questionnaire many applicants felt it could be fair to using other information such as interview score, UCAT score, or GCSE score to accept or reject offer-holders, and this could include in selecting from amongst 'near-misses'. Overall respondents to our questionnaire demonstrate a lack of confidence in the process of calculated grades. Given the concerns of the Equality and Humans Rights Commission, and the clear concerns also expressed in our study by some disadvantaged groups, there is a clear need to ensure that entrants as far as possible continue to reflect the breadth of those applying to study medicine.

The cancellation of public examinations and the use of calculated grades are not the only problems facing the 2020 application cohort. They are also at risk, particularly those from non-selective state schools, of coming to medical school having had less education over the previous few months,(14) meaning medical schools may need to provide additional teaching and resources to help students catch up. This is likely to be especially challenging for medical schools given the huge constraints on university budgets arising from drops in student numbers(30) and given that many are likely to be unable to open for face-to-face teaching at the start of the academic year, which in itself has unknown consequences. The finding that Black Asian and Minority Ethnic groups were more likely to think teacher-estimated calculated grades could be unfair is concerning, and greater efforts need to

[5] In the UK system, university offers are made before students take their exams. Universities typically give offers that are conditional upon students achieving particular grades. Students meet their offer(s) if they achieve or exceed the grades specified.

For peer review only - http://bmjopen.bmj.com/site/about/guidelines.xhtml

be made to ensure education is fair and perceived as fair by students and all stakeholders. Transparent and independent analysis of the impact of exam cancellations on different sociodemographic groups, once data become available, will also be important.

The 2020 cohort of entrants is likely to face more uncertainty than any cohort of medical student entrants in the past half-century, and our survey makes very visible the many concerns of those applicants.

Conclusions

The global tragedy of the coronavirus pandemic, in addition to its extensive mortality and morbidity, has resulted in huge and sudden disruptions to established ways of life including education and training at all levels. Medical education and training is no exception. The coronavirus pandemic will have significant and long term impacts on the selection, education and performance of our future medical workforce. Understanding how medical education will be affected is therefore important, and in particular how applicants to become the newest entrants to medical careers are being affected. Now more than ever we need medical education, and medical education research, to be prioritised and funded so we can ensure our future doctors are able to be resilient, successful and happy healthcare professionals providing excellent patient care. The present study provides a wide range of insights into the feelings of the 2020 cohort of applicants, only a small proportion of which we have adequately been able to report here.

Acknowledgements

Firstly we are immensely grateful to the several thousand medical school applicants who took the time to respond to survey with a very tight time window, and we particularly thank those who commented that they were pleased that the survey they gave them an opportunity to express their thoughts, feelings and anxieties. We could not include everything that was said, but all comments have been read by the team, and we hope that the current paper summarises some of those many and varied views. We are also grateful to Paul Garrud, Clare Owen, Konstantinos Lulo, and Ewan McNicol for their comments on earlier versions of the questionnaire.

Contributors

KW, DH and CM jointly developed the idea for the study, and developed the questionnaire together. DH was responsible for putting the questionnaire online, and for identifying applicants to whom it should be sent, as well as sending text and email reminders. DH and KW cleaned the data, and KW, DH and CM were all involved in data analysis. The report was written jointly by all three authors, and all authors have read and reviewed the final draft.

Disclaimers

KW and DH state that this publication presents independent research funded by the National Institute for Health Research (NIHR). The views expressed are those of the authors and not necessarily those of the NHS, the NIHR or the Department of Health and Social Care.

Ethical approval

The study was approved by the UCL Research Ethics Committee Chair on 8th April 2020 as an amendment to the ongoing UKMACS longitudinal questionnaire study (reference: 0511/014).

For peer review only

Competing interests

All authors have completed the ICMJE uniform disclosure form at http://www.icmje.org/coi_disclosure.pdf : Dr Woolf and Dr Harrison report grants and non-financial support from National Institute for Health Research during the conduct of the study; and Dr Woolf reports personal fees from Transforming Student Access and Outcomes (TASO), outside the submitted work. All authors report no other relationships or activities that could appear to have influenced the submitted work.

Copyright

The Corresponding Author has the right to grant on behalf of all authors and does grant on behalf of all authors, a worldwide licence (http://www.bmj.com/sites/default/files/BMJ%20Author%20Licence%20March%202013.doc) to the Publishers and its licensees in perpetuity, in all forms, formats and media (whether known now or created in the future), to i) publish, reproduce, distribute, display and store the Contribution, ii) translate the Contribution into other languages, create adaptations, reprints, include within collections and create summaries, extracts and/or, abstracts of the Contribution and convert or allow conversion into any format including without limitation audio, iii) create any other derivative work(s) based in whole or part on the on the Contribution, iv) to exploit all subsidiary rights to exploit all subsidiary rights that currently exist or as may exist in the future in the Contribution, v) the inclusion of electronic links from the Contribution to third party material where-ever it may be located; and, vi) licence any third party to do any or all of the above. All research articles will be made available on an open access basis (with authors being asked to pay an open access fee—see http://www.bmj.com/about-bmj/resources-authors/forms-policies-and-checklists/copyright-open-access-and-permission-reuse). The terms of such open access shall be governed by a Creative Commons licence—details as to which Creative Commons licence will apply to the research article are set out in our worldwide licence referred to above.

Dissemination declaration

We have disseminated the pre-print of this article to those invited to respond.

Data sharing statement

The data will be linked into the UK Medical Education Database **www.ukmed.ac.uk** to which researchers can apply for access.

Funding

KW is a National Institute for Health Research (NIHR) Career Development Fellow (NIHR CDF-2017-10-008) and is principal investigator for the UKMACS and UKMEDP089 projects supported by the NIHR funding.

DH is funded by NIHR grant CDF-2017-10-008 to KW.

CM has received no specific funding for this project.

This publication presents independent research funded by the National Institute for Health Research (NIHR). The views expressed are those of the author(s) and not necessarily those of the NHS, the NIHR or the Department of Health and Social Care.

For peer review only - http://bmjopen.bmj.com/site/about/guidelines.xhtml

References

1. Efuribe C, Barre-Hemingway M, Vaghefi E, Ballonoff Suleiman A. Engagement and the Inclusion of Young People in Matters That Affect Their Lives. Journal of Adolescent Health. 2020.
2. McKie A. Scrapped exams may spark UK admissions 'scramble'. Times Higher Education. 2020 26/03/2020.
3. Summer 2020 grades for GCSE, AS and A level, Extended Project Qualification and Advanced Extension Award in maths: Information for Heads of Centre, Heads of Department and teachers on the submission of Centre assessment grades. Coventry; 2020 03/04/2020. Contract No.: Ofqual/20/6607/1
4. Machin SJ, McNally S, Ruiz-Valenzuela J. Entry Through the Narrow Door: The Costs of Just Failing High Stakes Exams. CESifo Working Paper Series 2018(7008).
5. McManus IC, Woolf K, Dacre J, Paice E, Dewberry C. The Academic Backbone: longitudinal continuities in educational achievement from secondary school and medical school to MRCP(UK) and the specialist register in UK medical students and doctors. BMC Medicine. 2013;11(1):242.
6. Cancellation of GCSE is unfair to some students. The Guardian. 2020 29/03/2020.
7. Lough C. GCSEs: Only 39% teachers think 2020 grades fair for all. Times Educational Supplement. 2020 13/05/2020.
8. (HEPI) HEPI. New poll finds almost one-third of applicants feel less confident they will get into their chosen university since the Coronavirus pandemic. Oxford: Higher Education Policy Institute; 2020 [Available from: https://www.hepi.ac.uk/2020/04/07/new-poll-finds-almost-one-third-of-applicants-feel-less-confident-they-will-get-into-their-chosen-university-since-the-coronavirus-pandemic/;.
9. Montacute R, Holt-White E. COVID-19 and Social Mobility Impact Brief #2: University Access & Student Finance. 2020.
10. New statement on medical school admissions in 2020: Medical Schools Council; 2020 [updated 05/05/2020. Available from: https://www.medschools.ac.uk/news/new-statement-on-medical-school-admissions-for-2020-entry.
11. Coronavirus (COVID-19): cancellation of GCSEs, AS and A levels in 2020: Department for Education; 2020 [updated 1st May 2020. Available from: https://www.gov.uk/government/publications/coronavirus-covid-19-cancellation-of-gcses-as-and-a-levels-in-2020/coronavirus-covid-19-cancellation-of-gcses-as-and-a-levels-in-2020.
12. Lavy V. Do Differences in Schools' Instruction Time Explain International Achievement Gaps? Evidence from Developed and Developing Countries. The Economic Journal. 2015;125(588):F397-F424.
13. Carlsson M, Dahl GB, Öckert B, Rooth D-O. The Effect of Schooling on Cognitive Skills. The Review of Economics and Statistics. 2015;97(3):533-47.
14. Burgess S, Sievertsen HH. Vox CEPR Policy Portal [Internet]2020. Available from: https://voxeu.org/article/impact-covid-19-education.
15. Andrew A, Cattan S, Costa Dias M, Farquharson C, Kraftman L, Krutikova S, et al. Learning during the lockdown: real-time data on children's experiences during home learning. Institute for Fiscal Studies; 2020.
16. Monitoring COVID-19 Readiness in Schools: Teacher Tapp; 2020 [Available from: https://teachertapp.co.uk/monitoring-covid-19-readiness-in-schools/A
17. Understanding Society: Waves 1-9, 2009-2018 and Harmonised BHPS: Waves 1-18, 1991-2009. [data collection]. 12th Edition. In: University of Essex IfSaER, NatCen Social Research, Kantar Public, editor.: UK Data Service; 2019.
18. McCrae RC, PT. Personal trait as a human universal. American Psychologist. 1995;52(5).
19. van Buuren S, Groothuis-Oudshoorn K. mice: Multivariate Imputation by Chained Equations in R. Journal of Statistical Software 2011;45.
20. Van Buuren S. Flexible imputation of missing data. Second edition ed. New York: CRC Press; 2018.

For peer review only - http://bmjopen.bmj.com/site/about/guidelines.xhtml

21. Revelle W. psych: Procedures for Psychological, Psychometric, and Personality Research. Northwestern University, Evanston, Illinois.; 2019.
22. Murphy R, Wyness G. Minority Report: the impact of predicted grades on university admissions of disadvantaged groups. Centre for Education Policy and Equalising Opportunities, UCL Institute of Education; 2020.
23. Lough C. Coronavirus: Williamson warned over grading 'injustice'. Times Educational Supplement 2020 02/04/2020.
24. Predicted grading during COVID-19 could limit young people's futures: Equality and Human Rights Commission; 2020 [Available from: https://www.equalityhumanrights.com/en/our-work/news/predicted-grading-during-covid-19-could-limit-young-peoples-futures.
25. McManus C, Woolf K, Harrison D, Tiffin PA, Paton LW, Yet Fong Cheung K, et al. Calculated grades, predicted grades, forecasted grades and actual A-level grades: Reliability, correlations and predictive validity in medical school applicants, undergraduates, and postgraduates in a time of Covid-19. forthcoming. 2020.
26. Curtis S, Smith D. A comparison of undergraduate outcomes for students from gateway courses and standard entry medicine courses. BMC Medical Education. 2020;20(1):4.
27. Wakeford R, Ludka K, Woolf K, McManus IC. Fitness to practise sanctions in UK doctors are predicted by poor performance at MRCGP and MRCP(UK) assessments: data linkage study. BMC Medicine. 2018;16(1):230.
28. Physicians RCo. Underfunded. Underdoctored. Overstretched. The NHS in 2016. London; 2016.
29. Devine OP, Harborne AC, McManus IC. Assessment at UK medical schools varies substantially in volume, type and intensity and correlates with postgraduate attainment. BMC Medical Education. 2015;15(1):146.
30. Achieving stability in the higher education sector following COVID-19. Universities UK; 2020 08/04/2020.

For peer review only

For peer review only - http://bmjopen.bmj.com/site/about/guidelines.xhtml

Figure captions

Figure 1: Perceptions of the fairness of methods medical schools could use to decide whether or not to accept applicants who currently hold an offer now that exams have been cancelled.

Figure 2: Acceptability of actions medical schools could take if they have more applicants meeting offers than they have places.

Figure 3: Views on how current applicants should be considered by medical schools if they reapply next year.

Figure 4: Aspects of calculated grades that respondents were generally more a) positive and b) negative about

Figure 5: Proportion of respondents undertaking various activities to prepare for medical school or university.

Figure 6: Amount of time respondents reported spending on various activities during the lockdown.

Figure 7: Scree plot for the factor analysis of 87 attitudinal variables.

For peer review only

For peer review only - http://bmjopen.bmj.com/site/about/guidelines.xhtml

Table 1 : Demographics for the full sample and the restricted sample (of those in Year 13, with at least three predicted A-level*s, no achieved* A-level*s, who had applied to study medicine). Rounding to prevent identifying individuals.*

	Full sample N (%)	Restricted sample N (%)
Female	1968 (68)	1097 (70)
Male	749 (26)	416 (27)
Other	20 (<1%)	<10 (<1)
Missing	140 (5)	Rounded to 40 (3)
White	670 (23)	516 (33)
Asian	301 (11)	228 (15)
Black	79 (3)	58 (4)
Mixed/Other	104 (4)	87 (6)
Missing	1723 (60)	673 (43)
1+ parents with degree	1831 (64)	1046 (67)
First in family	895 (33)	465 (30)
Missing	151 (5)	51 (3)
1+ parents in the highest socioeconomic group	1910 (66)	1097 (70)
No parents in the highest socioeconomic group	1742 (30)	439 (28)
Missing	116 (4)	26 (2)
No parent doctors	2408 (88)	1334 (85)
1+ parents who are doctors	344 (13)	192 (12)
Missing	125 (4)	36 (2)
Non-selective state school	785 (27)	590 (38)
Private or selective school	783 (27)	568 (36)
Missing	1309 (46)	404 (26)
IMD quintile 5 (most deprived – reverse scored)	310 (11)	169 (11)
IMD quintile 4 (reverse scored)	361 (13)	218 (14)
IMD quintile 3 (reverse scored)	410 (14)	236 (15)
IMD quintile 2 (reverse scored)	461 (16)	267 (17)
IMD quintile 1 (least deprived – reverse scored)	704 (25)	441 (28)
Missing	631 (22)	231 (15)
In Year 13/S6	2212 (77)	1562 (100)
One year post-Year 13	179 (6)	0 (0)
Have/studying for a degree	340 (12)	0 (0)
Mature without a degree/other	146 (5)	0 (0)
Missing	0 (0)	0 (0)
England	2003 (70)	1281 (82)
Scotland	170 (6)	<1 (<1)
Wales	78 (3)	50 (3)
Northern Ireland/ Forces/Islands	66 (2)	Rounded to 40 (2)
Other/missing	560 (20)	192 (12)
Total	2877 (100)	1562 (100)

For peer review only - http://bmjopen.bmj.com/site/about/guidelines.xhtml

***Table 2**: Predictors of agreement with statements relating to calculated grades. Predictors are ordered left to right by strength of relationship to the statement. Only statements that showed significant differences by social/demographic group after controlling for prior attainment and the number of offers are shown.*

	**Independent predictors of agreement with statement**			
I would prefer not to have calculated grades at all and instead take A levels (or equivalents) in September.	Lower predicted A level points	BAME	Fewer conditional offers	Female
Overall, I would prefer to withdraw entirely from calculated grades and sit exams properly next summer.	Lower predicted A-level points	BAME	Fewer conditional offers	Female
The process described above is the best way to be fair to most students.	Higher predicted A-level points	White	Higher UCAT/BMAT scores	
I feel confident this process will result in an accurate assessment of my true abilities.	Higher predicted A-level points	White	Male	
Many students do better than their teachers expect; calculated grades cannot take that into account.	Lower predicted A-level points	BAME		
My teachers should take into account the disruption caused by coronavirus when judging grades.	Lower predicted A-level points	BAME		
Calculated grades should be based only on my performance, not on how previous students at my school performed.	Non-Selective State school	Higher deprivation		
I am confident in my teachers' abilities at grading and ranking students.	Higher predicted A-level points	White		
My teachers do not know enough about me to grade and rank me accurately.	Lower predicted A-level points	BAME	Fewer conditional offers	
In large schools/colleges, it is difficult to see how teachers can rank so many students.	BAME	Lower predicted A-level points		
Teachers judging grades should take into account the fact that many students do not do well in mocks but then work hard and do well in exams.	Lower predicted A-level points	Female	Fewer conditional offers	
Employers and universities in the future will treat grades from 2020 differently compared to exam grades taken from other years.	Female	Fewer conditional offers		

For peer review only - http://bmjopen.bmj.com/site/about/guidelines.xhtml

Table 3: School-provided educational resources used by respondents from non-selective state schools and private/selective schools

	N (%) used resource			
	Non selective state school	Private or selective school	Total	p value
Online resources	342 (63.3)	439 (80.0)	781 (71.7)	<.001
Paper resources	315 (58.3)	375 (69.6)	690 (63.9)	<.001
Online formative tests	187 (34.8)	260 (48.2)	447 (41.5)	<.001
Pastoral support	160 (29.7)	199 (37.2)	359 (33.4)	0.009
University application support	152 (28.5)	174 (32.3)	326 (30.4)	0.174
Online teaching in real time	66 (12.4)	248 (45.7)	314 (29.2)	<.001
Online summative tests	70 (13.2)	95 (17.7)	165 (15.4)	0.042
Other	12 (6.3)	25 (14.2)	37 (10.1)	0.011

.

For peer review only

For peer review only - http://bmjopen.bmj.com/site/about/guidelines.xhtml

Table 4: Respondents' main reasons for not using school educational resources during the shutdown by school type

Resource not used	Reason not used	N (%) resource NOT used Non-selective state school	Private or selective school	Total
Online resources	Not available	96 (46.6)	48 (43.2)	144 (45.4)
	Don't need to	80 (38.8)	49 (44.1)	129 (40.7)
Paper resources	Not available	109 (50.5)	74 (46.8)	183 (48.9)
	Don't need to	88 (40.7)	69 (43.7)	157 (42.0)
Online formative test	Not available	206 (60.2)	129 (48.3)	335 (55.0)
	Don't need to	119 (34.8)	116 (43.3)	235 (38.6)
Pastoral support	Not available	161 (42.6)	94 (28.4)	255 (36.0)
	Don't need to	194 (51.3)	205 (61.9)	399 (56.3)
Uni application support	Not available	185 (49.9)	141 (40.5)	326 (45.3)
	Don't need to	155 (41.8)	182 (52.3)	337 (46.9)
Online teaching in real time	Not available	337 (71.7)	189 (63.0)	526 (68.3)
	Don't need to	109 (23.3)	99 (33.0)	208 (27.0)
Online summative test	Not available	289 (65.4)	223 (52.5)	512 (59.1)
	Don't need to	142 (32.1)	177 (41.6)	319 (36.8)
Other	Not available	66 (54.1)	42 (39.3)	108 (47.2)
	Don't need to	47 (38.5)	49 (45.8)	96 (41.9)

.

For peer review only

For peer review only

For peer review only - http://bmjopen.bmj.com/site/about/guidelines.xhtml

Table5: Predictors of factor scores. Set A includes Number of offers, GCSE points, Predicted A-level points, UCAT/BMAT score, Private/Selective school, Female, BAME, Degree-educated parent(s) and Deprived area. Set B includes Set A plus Highest socioeconomic group, doctor parent(s), and Big5 personality factors Agreeableness, Conscientiousness, Extraversion, Neuroticism and Openness. All predictors reported have p<.01, and are reported in descending order of significance (i.e. most significant at the top).

	**Set A Predictors in order of magnitude**	**Set B Predictors in order of magnitude**
Factor 1: Lack of confidence in calculated grades	Lower predicted A-levels<br>BAME<br>Fewer conditional offers<br>Female	Lower predicted A-levels<br>BAME<br>Fewer conditional offers<br>Higher Openness<br>Lower Conscientiousness<br>Female
Factor 2: Special treatment next year for rejected applicants	Lower predicted A-levels<br>Lower UCAT/BMAT	Lower predicted A-levels<br>Higher Openness<br>Lower UCAT/BMAT<br>Higher Neuroticism<br>Higher Extraversion
Factor 3: Other selection measures to be taken into account	Higher UCAT/BMAT<br>Lower predicted A-levels<br>Male	Higher UCAT/BMAT<br>Lower predicted A-levels<br>Higher Extraversion<br>Male<br>Lower Conscientiousness
Factor 4: Preparing for medical school	White<br>Female	Higher Conscientiousness<br>Lower Neuroticism<br>White<br>Female<br>Higher Agreeableness<br>Higher Openness
Factor 5: Importance of background and experience	Lower UCAT/BMAT<br>BAME<br>Female	Higher Openness<br>Lower UCAT/BMAT<br>Fewer conditional offers<br>BAME
Factor 6: Resources from school for home study	Selective school<br>Lower GCSE<br>Fewer conditional offers	Selective School<br>Lower GCSE<br>Lower Extraversion<br>Higher Openness

For peer review only - http://bmjopen.bmj.com/site/about/guidelines.xhtml

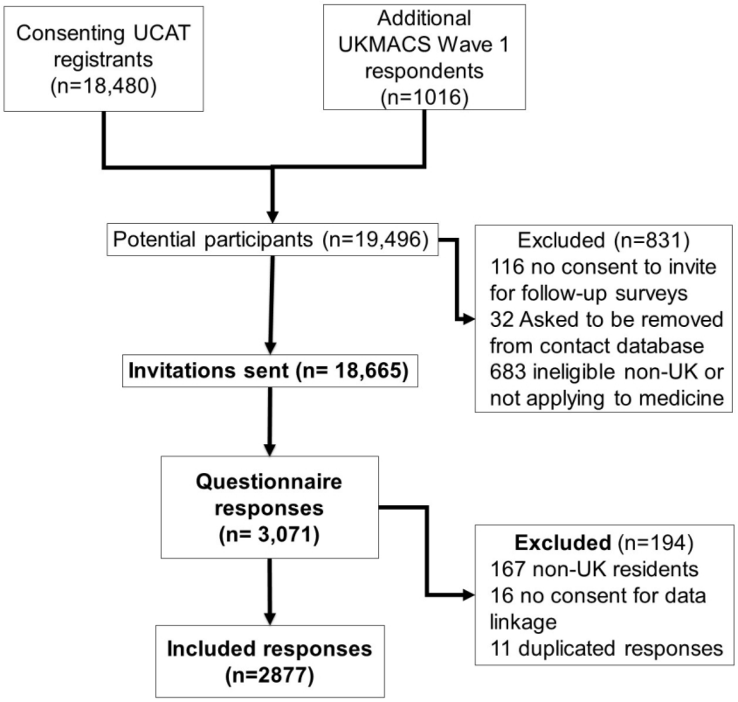

Figure 1: Participant flow diagram.

451x338mm (96 x 96 DPI)

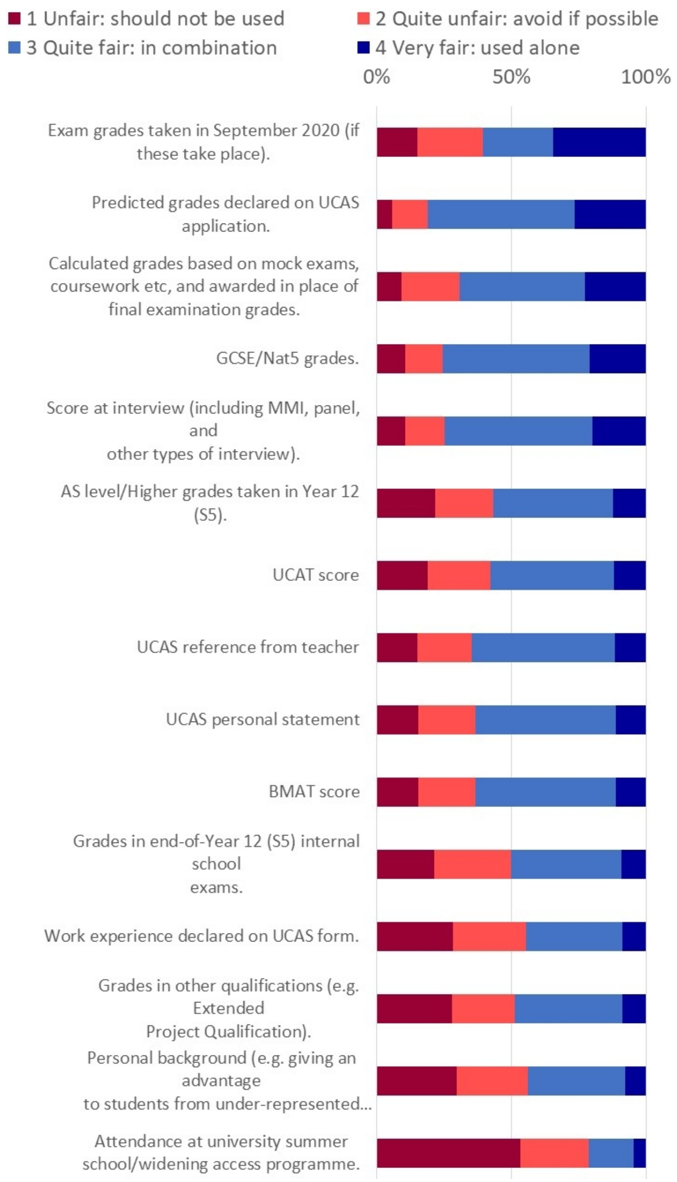

Figure 2: Perceptions of the fairness of methods medical schools could use to decide whether or not to accept applicants who currently hold an offer now that exams have been cancelled.

190x338mm (96 x 96 DPI)

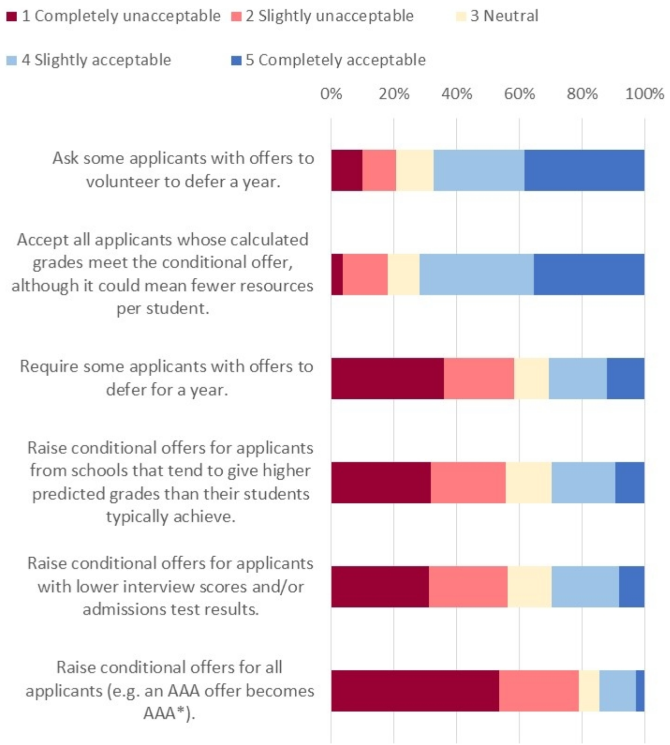

Figure 3: Acceptability of actions medical schools could take if they have more applicants meeting offers than they have places.

190x338mm (96 x 96 DPI)

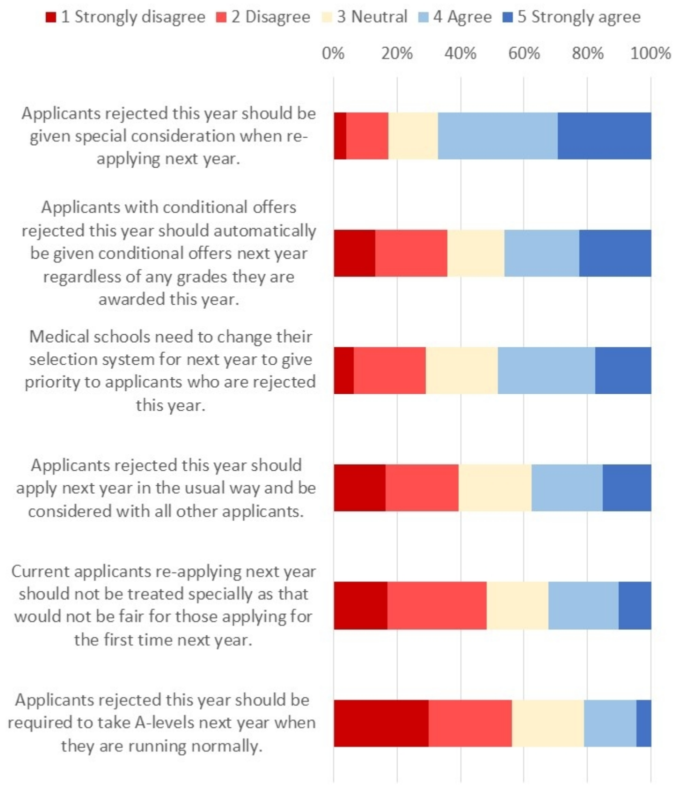

Figure 4: Views on how current applicants should be considered by medical schools if they reapply next year.

190x338mm (96 x 96 DPI)

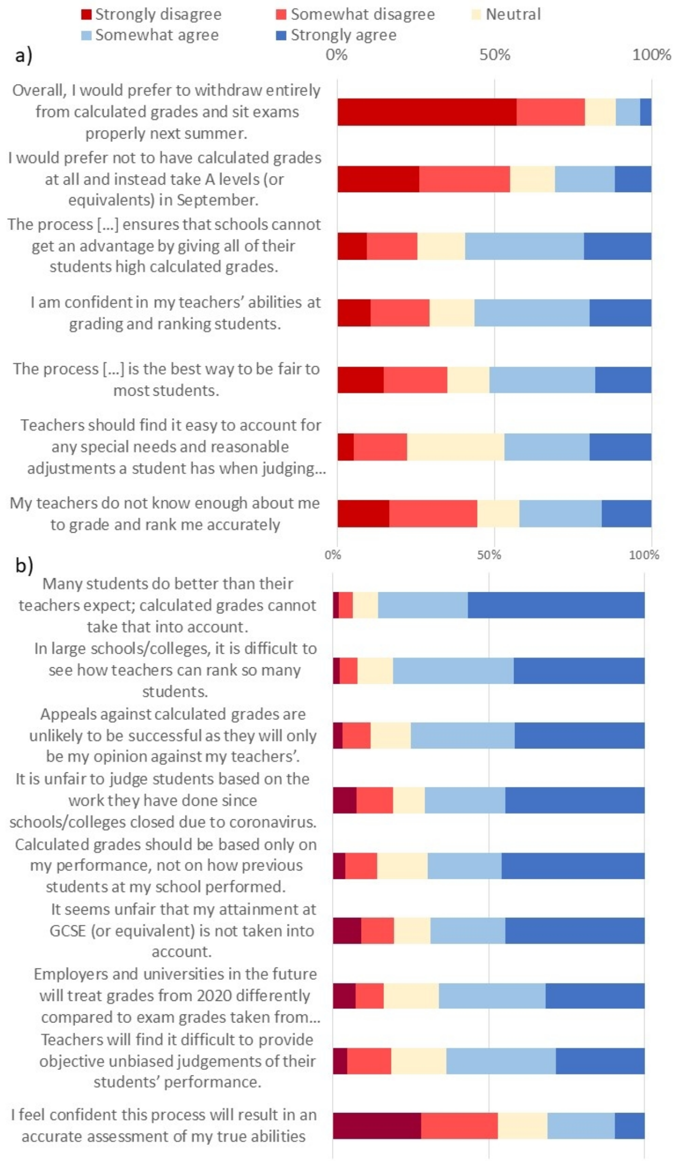

Figure 5: Aspects of calculated grades that respondents were generally more a) positive and b) negative about

190x338mm (96 x 96 DPI)

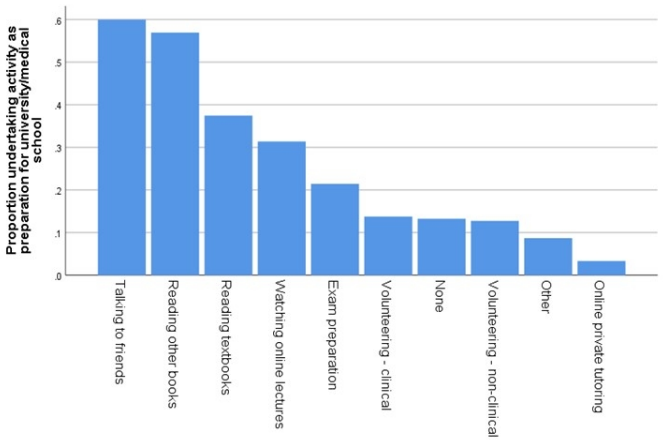

Figure 6: Proportion of respondents undertaking various activities to prepare for medical school or university.

190x338mm (96 x 96 DPI)

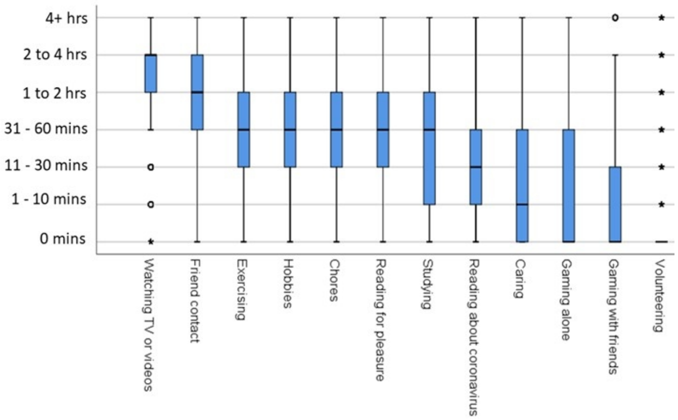

Figure 7: Amount of time respondents reported spending on various activities during the lockdown.

190x338mm (96 x 96 DPI)

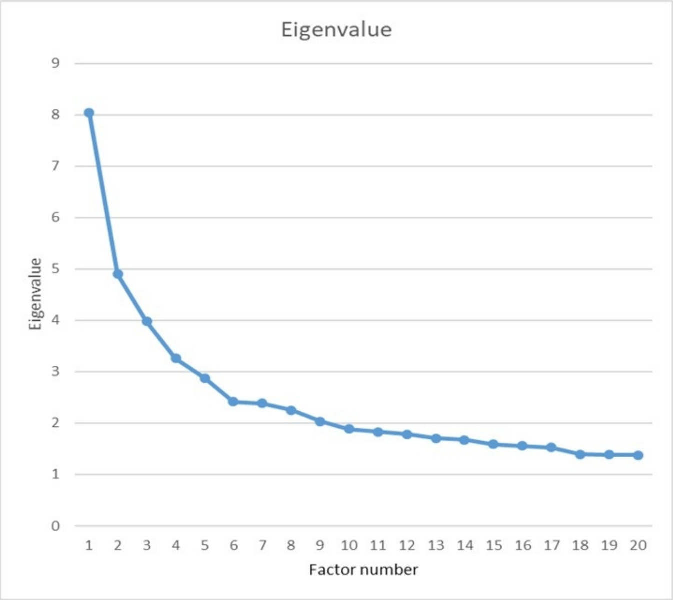

Figure 8: Scree plot for the factor analysis of 87 attitudinal variables.

190x338mm (96 x 96 DPI)

Supplementary file 1: Missing data.

The analysis considered 120 measures in the restricted sample, divided into:

1. *Questionnaire items.* The questionnaire asked about attitudes to 87 different topics concerning medical school entrance. Of 153,076 data points, 10788 (7.2%) were missing. For the individual variables, the median percentage of missing data values was 0.48%, with 75 measures having fewer than 5% of missing values.

2. *Demographic and educational items*. For 12 demographic measures, 462 of 18744 measures were missing (2.5%), with a median of 1.0% per measure, and 11 measures having fewer than 5% missing values. Ethnic origin was not asked about in the present study. The ethnicity of 889 respondents who had reported it in a previous questionnaire were imported into the present dataset; 43.1% of ethnicity measures were therefore missing. IMD_Quintile was obtained from postcodes in England, Wales and Scotland, and was missing in 14.8% of cases.

There were four educational attainment items (grades in the highest-scoring 'top' three predicted A-level grades, UCAT score, BMAT score, and mean GCSE grade). Top three predicted A-level grades were present for all because the sample was based on that criterion. Of the remaining three measures, 1852 out of 4686 (39.5%) were missing: UCAT scores were missing in 13.6% of cases, and BMAT scores in 61.3% of cases, but in both cases missing values were mostly structurally missing, candidates mostly having taken only one aptitude test or the other. Mean GCSE grade was missing in 43.1% of cases, having been imported from a previous UKMACS questionnaire.

Participants self-reported their current or most recent school in the current questionnaire. This question was also present in the Wave 1 UKMACS questionnaire. For schools in England, publicly-available administrative data were available on school type (e.g. independent, voluntary aided) and for state-funded schools there were data on whether the admissions policy was selective or non-selective. These were combined to create a binary variable of School Type (non-selective state schools vs private/selective schools) for 1132 respondents (27.1% missing). A composite variable was created using present responses and the responses in the Wave 1 questionnaire, so data were available for 1158 respondents with values missing in 25.9% of respondents.

Missing values were imputed using the *mice* package.(18) Following the general advice of van Buuren (19) missing values were calculated using *pmm* (predictive mean matching), which as van Buuren says, is a good " all-round method with exceptional properties" (p.84). *pmm* is the default method in the *mice()* function for all scale types (binary, ordinal, numeric) and has the advantage that imputed values are always taken from the existing range of actual values in the data, with *pmm* being robust against mis-specification. The number for the pool of candidate donors, *d*, was set at 5, the default in *mice()*, and the number of imputations, *m*, was set at 25.

Regression analyses on the 25 *mira* datasets were carried out using the *lm()* function within the *with()* function, and separate sets of results in the *mipo* dataset were combined with the *pool()* function. Regression analyses entered all socio-demographic and educational predictor variables into the analysis simultaneously, and results are only reported which were significant with p<.01 after taking all other variables into account, so the analysis is relatively conservative. The nine socio-demographic and educational variables used were: ethnicity, gender, school type, parental higher education, IMD quintile, mean GCSE points, mean top three predicted A-levels, UCAT score, number of medical school offers.

For peer review only

Supplementary file 2: Results for the 665 post-Year 13 respondents excluded from the restricted sample.

This sample includes mature and graduate applicants from the whole of the UK.

Applicant views on admissions

Perceptions of the fairness of methods to select or reject offer holders

As with the restricted sample, no single method was perceived as fair enough to use on its own but many were considered fair enough to use in conjunction with others.

Since this group includes those currently at university and graduate applicants, we have included responses to two additional items: *For those in their final year at university, marks earlier in their course*, which was considered very fair by 35% and quite fair by 45%, and *GAMSAT score (for Graduate Entry students)* which was considered very fair by 17.6% and quite fair by 46.8%.

Compared to those in Year 13, *Predicted grades declared on UCAS form* were considered much less fair and *Personal background (e.g. giving an advantage to students from under-represented groups)* was considered by a majority (52.1%) to be very fair/quite fair.

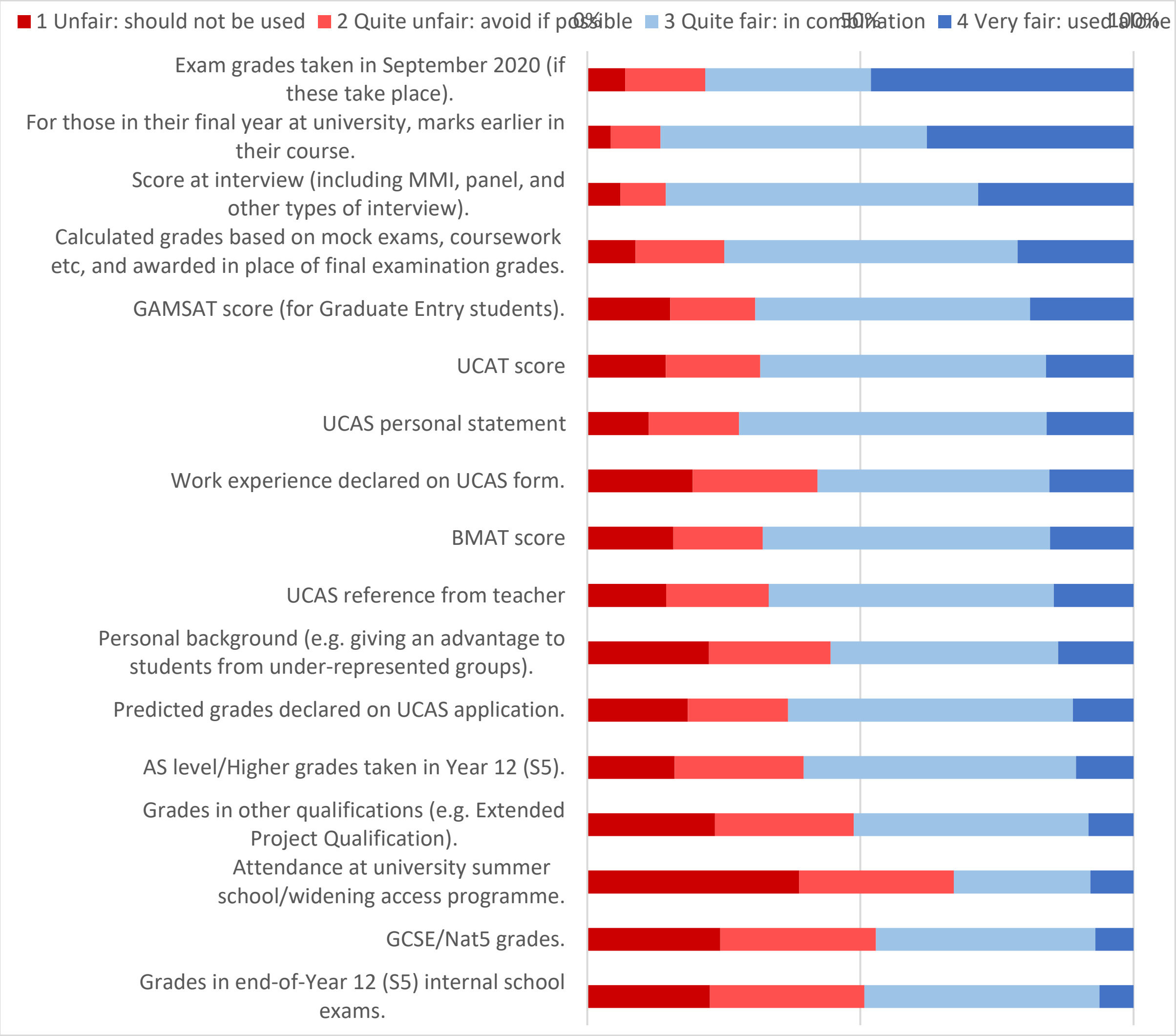

Figure S1: Perceptions of the fairness of methods medical schools could use to decide whether or not to accept applicants who currently hold an offer now that exams have been cancelled. Post-Year13 respondents only.

Acceptability of options for dealing with a situation in which more students meet their offers than there are medical school places

The only option that was rated as slightly or completely acceptable by the majority of respondents (64.6%) was asking for volunteers to defer. Accepting all applicants who meet the conditional offer was the second most acceptable and more acceptable than it was unacceptable.

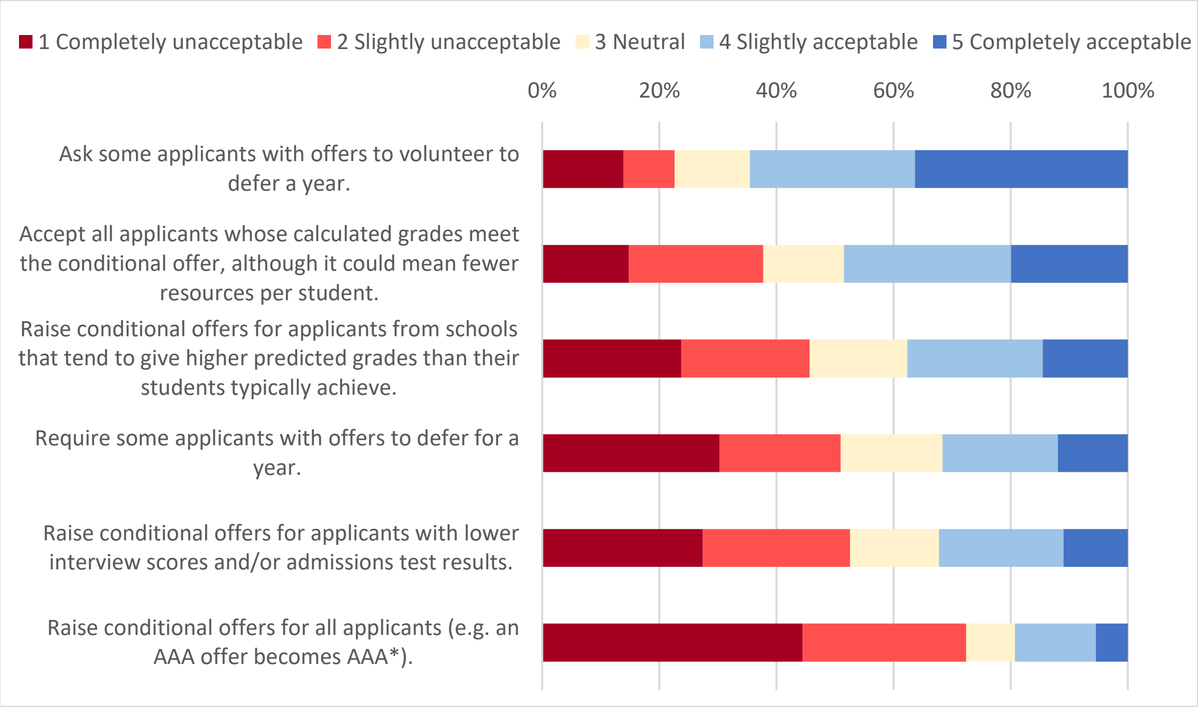

Figure S2: Acceptability of actions medical schools could take if they have more applicants meeting offers than they have places. Post-Year 13 respondents only.

Perceptions of potential impact on admissions for 2021

Respondents were even more divided than in the restricted sample, with about half of respondents (53.8%) agreeing/strongly agreeing that *Applicants rejected this year should be given special consideration when re-applying next year* but 51.5% agreeing/strongly agreeing that *Applicants rejected this year should apply next year in the usual way and be considered with all other applicants*.

For peer review only

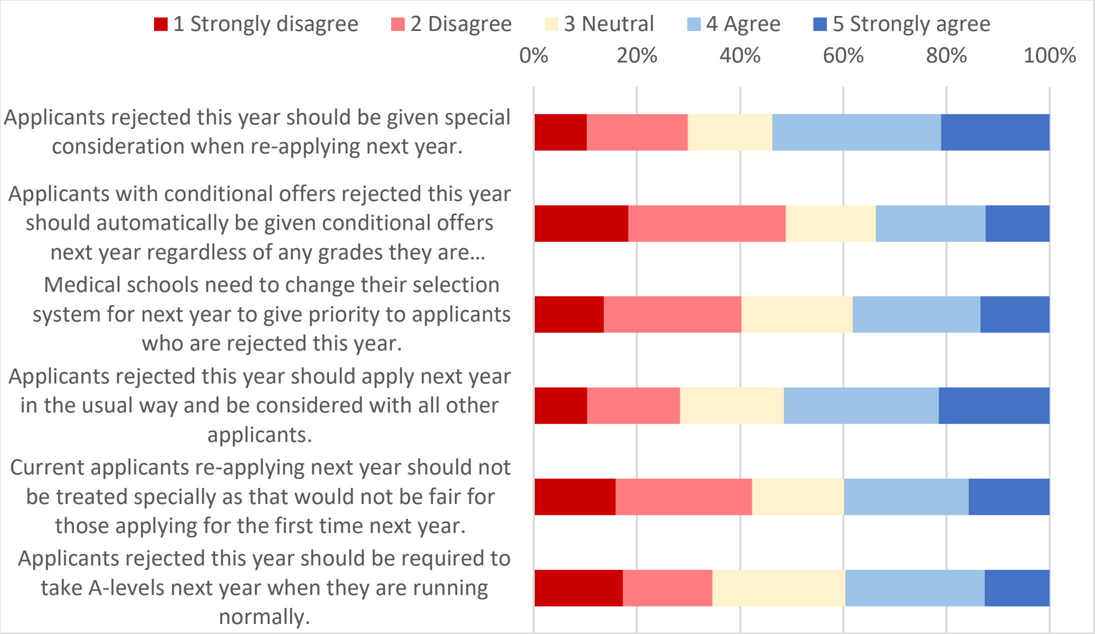

Figure S3: Views on how current applicants should be considered by medical schools if they reapply next year. Post-Year 13 respondents only.

Starting academic year 2020/2021

A majority of respondents (n=375, 56.4%) believed that if necessary, medical schools should *Defer the start of the academic year only when face-to-face teaching is possible* with 285 respondents (42.6.9%) believing that medical schools should *Start the academic year on time using distance learning for as long as is necessary*.

Education and university preparation

Perceptions of process to award calculated grades in lieu of examination grades

Post-Year 13 respondents were generally more negative about calculated grades than respondents in the restricted sample and unsurprisingly there were more "neutral" responses in general and specifically to questions about their own teachers and grades.

For peer review only

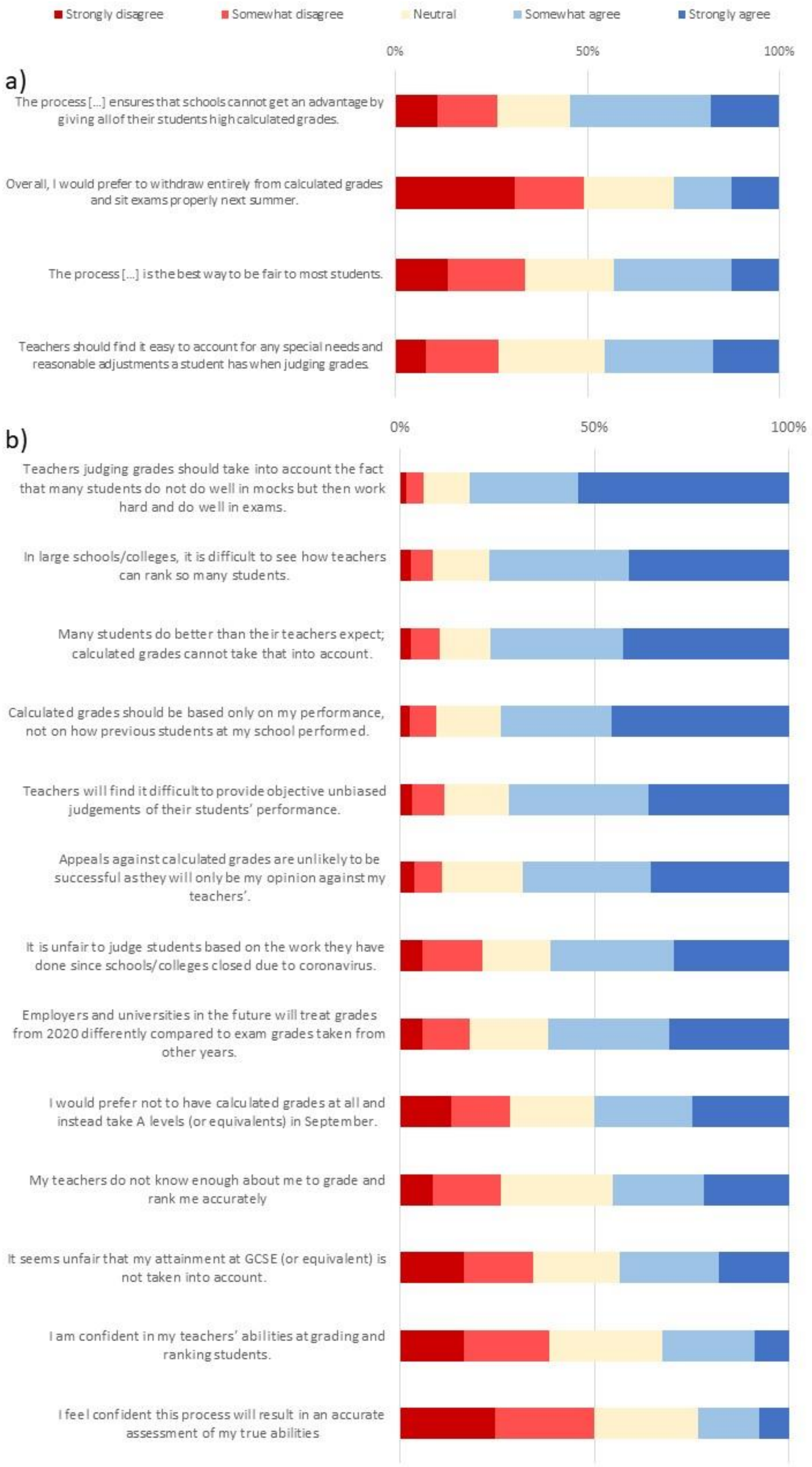

Figure S4: Aspects of calculated grades that respondents were generally more a) positive and b) negative about. Post-Year 13 respondents only.

A number of applicants were re-sitting their examinations and/or were not studying at a school or college but nonetheless were due to take examinations this summer (so-called ‘private candidates’). Several expressed concerns about whether the institution they were due to take their exams with would give them a calculated grade, and if so, what information that grade would be based on:

> *“As a resit student, my previous college which I was registered to retake my exams with this year have decided that they cannot give me calculated grades. I am unsure how to maintain my offers despite not getting grades.”*

> *“I am extremely concerned about how offers made to private candidates who cannot get predicted grades from a school will be treated. Though I had been studying in my lunchtimes/evenings/weekends for over a year, I quit my job 4 days after getting an offer from [redacted] in order to have time to put the work in to get the grades I need. I achieved straight A*s at GCSEs and A-level, so I know how much work it takes to get top grades. I am terrified universities I have offers from will wash their hands of me as I don't have any grades, or forced to defer for a year because universities won't wait for September exam results. Ofqual and exams boards keep saying no student will be disadvantaged, but it appears private candidates like myself may fall through the cracks.”*

> *“I worry that I, as a resitting privately tutored student, will be disadvantaged by the "calculated grades system", as I haven't been in school this year and thus have no exams or schoolwork that could be provided as evidence to support a predicted grade.”*

> *“I’m worried about how they’ll handle resits who have been independently studying as I need to go from a B to an A but am worrying that my old school(exam centre) won’t provide me with a grade even though I’m certain that I’d be able to get an A had I taken the exam. I also can’t afford to take another gap year so I’m hoping unis will take situations like these independently as it would be very unlikely that I’d receive the same grade as last year had I resat.”*

> *“For exam centre who cannot provide grades for resit external students please consider our previous attainment especially if for an applicant like myself has achieved AAB grades from last year and narrowly missed the A grade in Maths by 8 marks. It would be unfair for me to have to take another gap year if I don’t receive a grade this summer.”*

Education since the shutdown

Although participants were post Year-13 many were still in education, whether at school, college or university. The mean number of resources used by participants was 2.9 (SD=1.86).

Like Year 13 respondents, post-Year 13 respondents were using mostly online and paper resources, but 42.8% of post-Year 13 respondents reported having online teaching in real time and nearly half (49.6%) were having online summative tests and; 30.6% reported that their school/college/university would be assessing them formally on work since the closure of schools (although 42.1% reported that this was not applicable to them). See Figure S5.

For peer review only

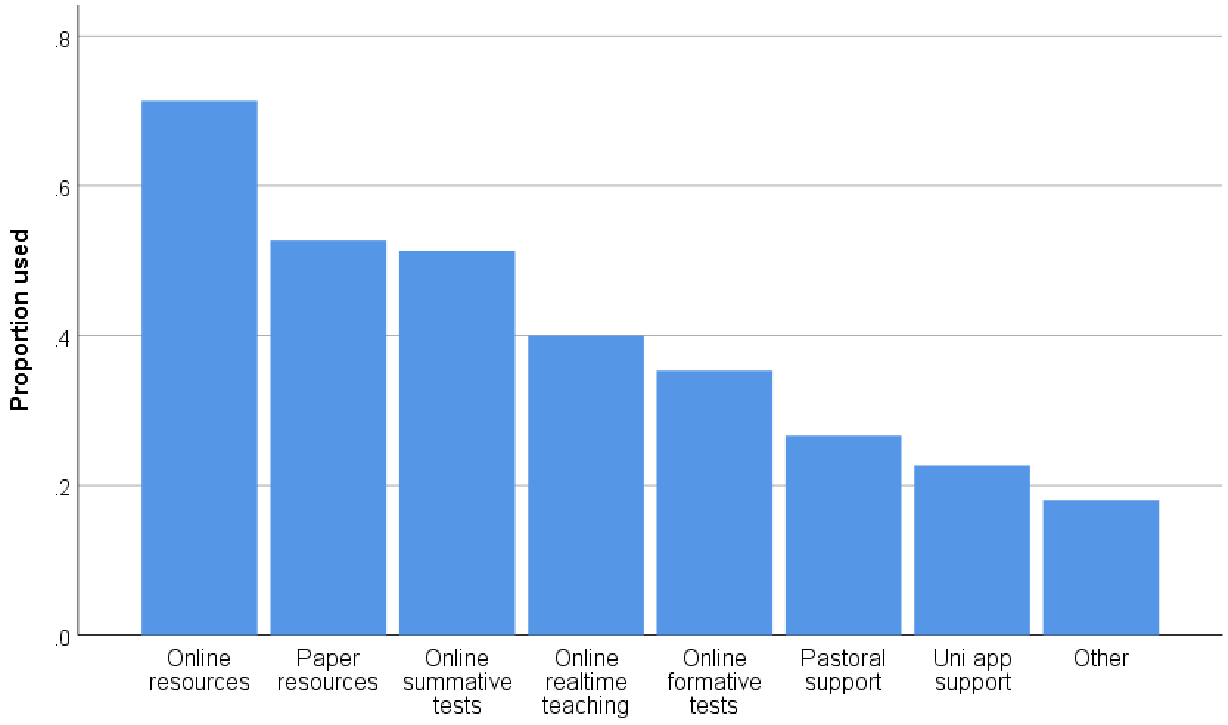

Figure S5: Proportion of post-Year 13 respondents using educational resources since the closure of schools.

Preparation for medical school/university

Post-Year 13 respondents were doing similar sorts of preparation, although they were talking to their friends less. Of the 100 (15.0% of the sample) who said they were not doing any preparation, reasons were different from those in the restricted sample. They were five times more likely to say they did not have time (31.0% vs 6.3%), about half as likely to say they were too worried and not able to focus (26.0% vs 42.5%), and over half as likely to say they did not have resources (15.0% vs 29.5%). A similar percentage selected caring for others as a reason (13.0%), not going to university this year (19.0%), being unwell (6.0%).Respondents could select multiple reasons.

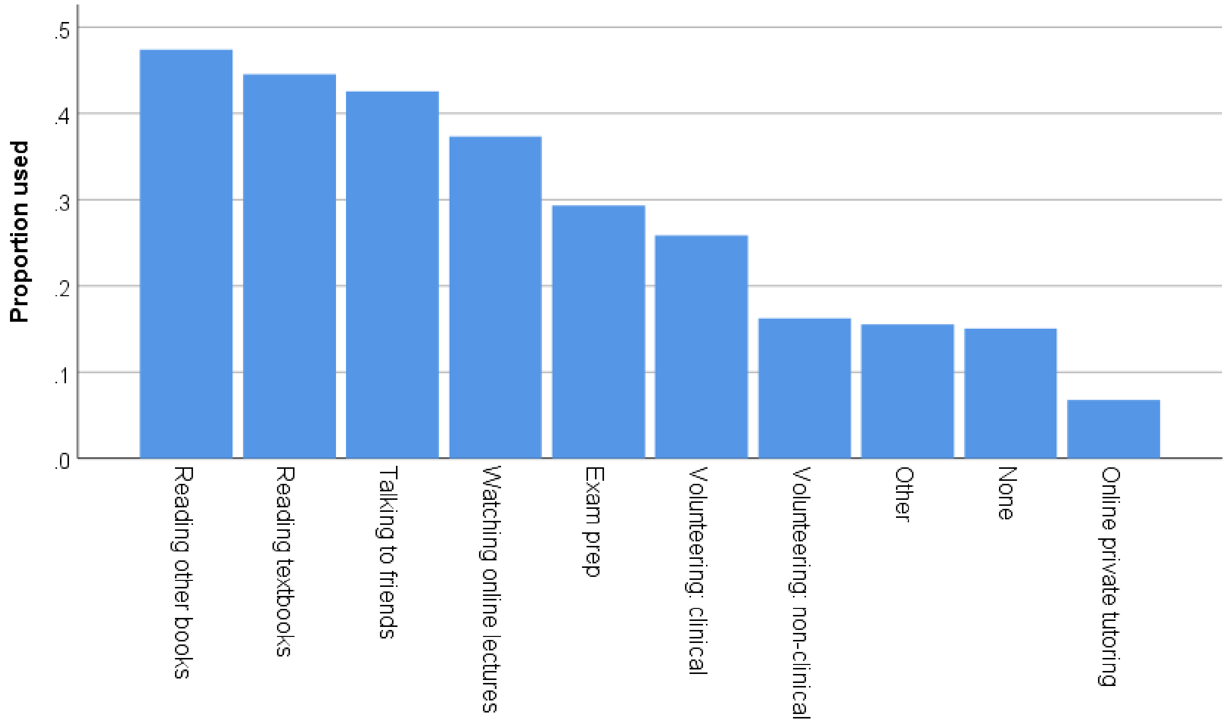

Figure S6: Proportion of respondents undertaking various activities to prepare for medical school or university. Post-Year 13 respondents only.

Time spent during the lockdown

Post-Year 13 respondents were spending broadly similar amounts of time on various activities as those in the restricted sample although they were spending more time volunteering and reading about coronavirus, and less time studying and gaming with friends.

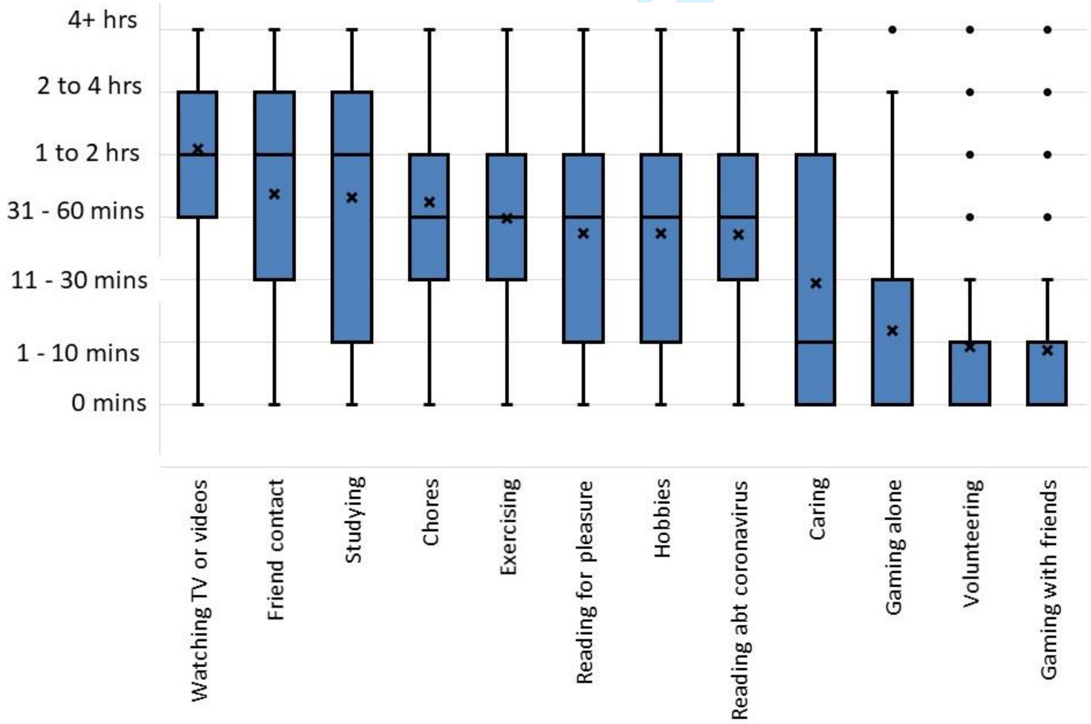

Figure S7: Amount of time respondents reported spending on various activities during the lockdown. Non-Year 13 respondents only.

For peer review only

Supplementary file 3: Results for 125 Scottish S6 respondents excluded from the main analyses

Applicant views on admissions

Perceptions of the fairness of methods to select or reject offer holders

Scottish applicants were similarly uncertain that any measure was fair enough to use alone, however unlike applicants from other UK countries they were more positive about the fairness of using AS level/Higher grades taken in Year 12. This is probably because AS levels are no longer in widespread use whereas Highers are. Scottish applicants were also relatively more positive about the use of calculated grades (83.2% quite or very fair).

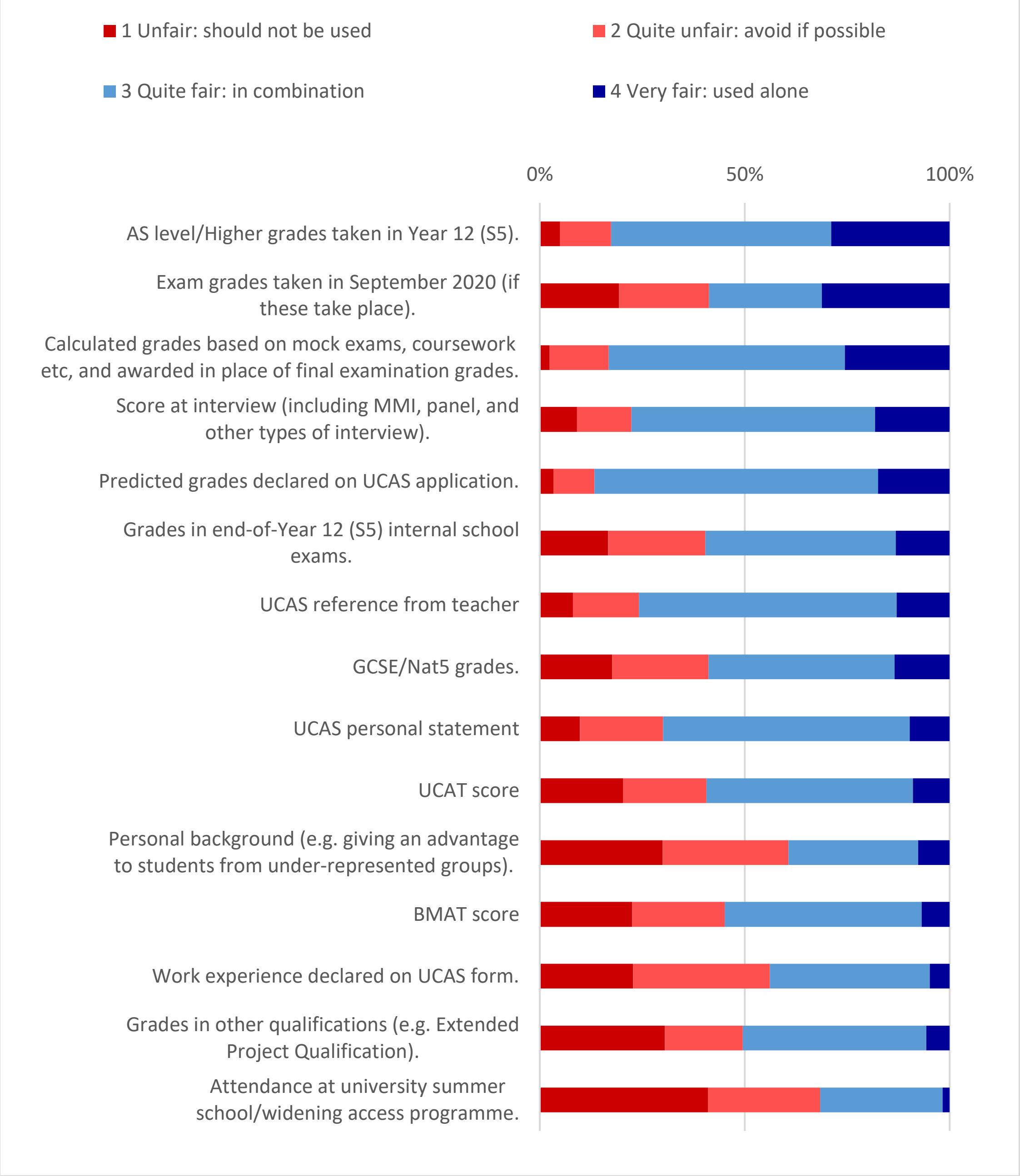

Figure S8: Perceptions of the fairness of methods medical schools could use to decide whether or not to accept applicants who currently hold an offer now that exams have been cancelled. Post-Scottish S6 respondents only.

Acceptability of options for dealing with a situation in which more students meet their offers than there are medical school places

As with other school students, the two acceptable options were accepting all applicants and asking for volunteers to defer.

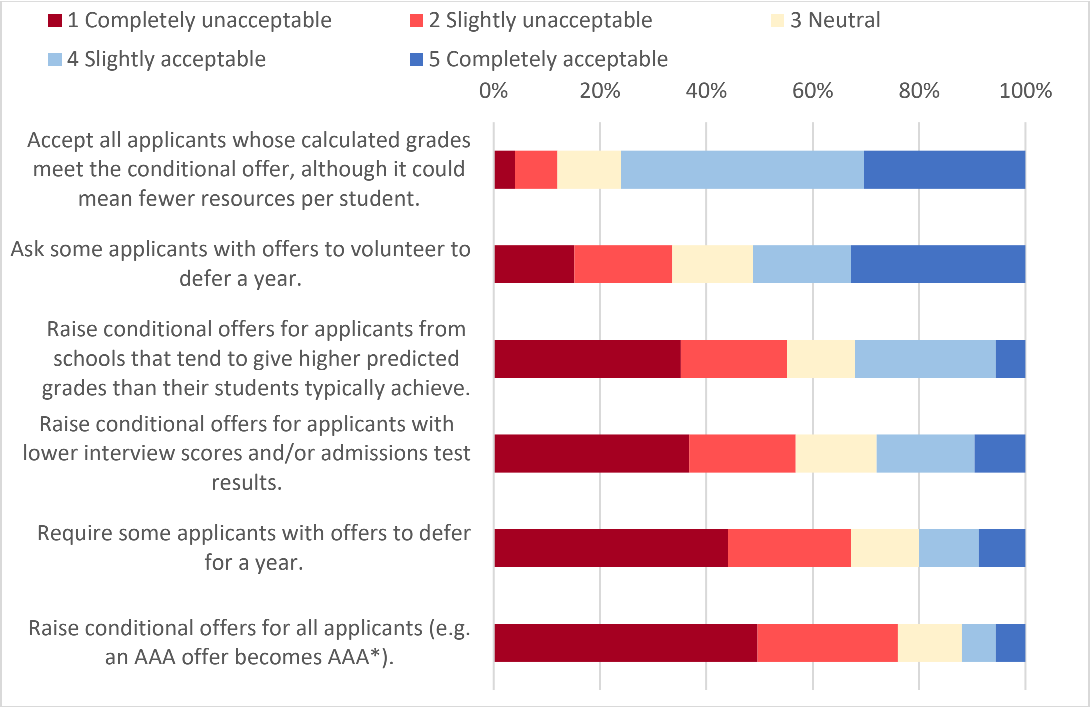

Figure S9: Acceptability of actions medical schools could take if they have more applicants meeting offers than they have places. Scottish S6 respondents only.

Perceptions of potential impact on admissions for 2021

Scottish S6 respondents were even more divided than in other UK countries: half the sample (52.8%) agreed that applicants rejected this year should be given special consideration and half (53.2%) agreeing that they should reapply next year in the usual way and be considered with all other applicants.

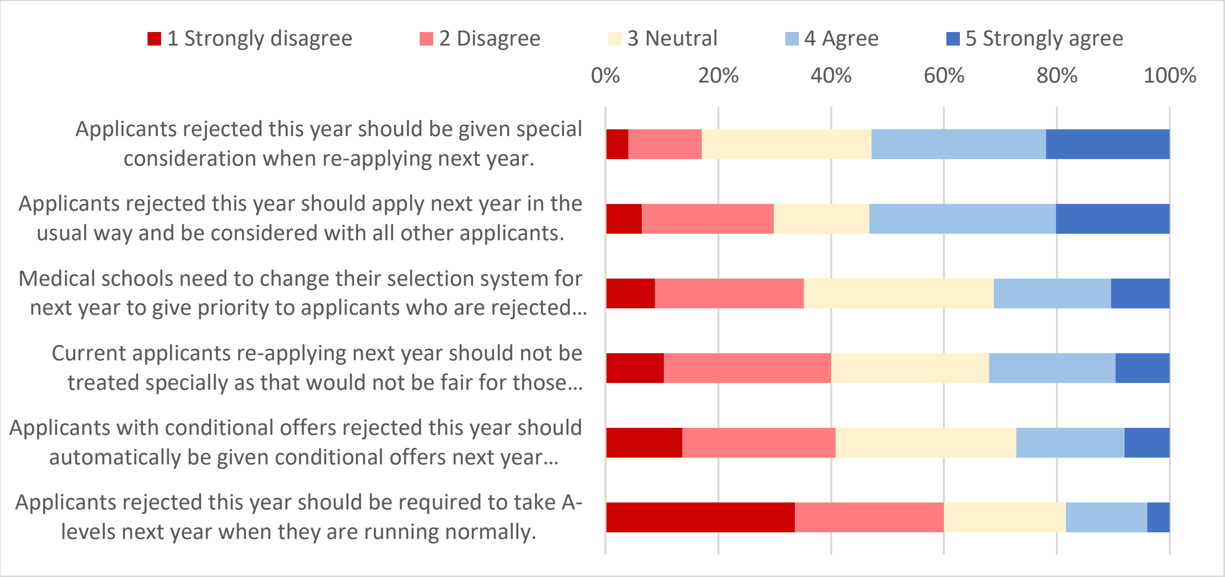

Figure S10: Views on how current applicants should be considered by medical schools if they reapply next year. Scottish S6 respondents only.

Starting academic year 2020/2021

A majority of respondents (n=70; 56.0%) believed that if necessary, medical schools should *Defer the start of the academic year only when face-to-face teaching is possible* with 55 respondents (44.0%) believing that medical schools should *Start the academic year on time using distance learning for as long as is necessary*.

Education and university preparation

Perceptions of process to award calculated grades in lieu of examination grades

Scottish respondents were generally slightly more positive about calculated grades than their equivalents in other UK countries. They were more positive about their teacher's ability to rank and grade students accurately (70.4% agree/strongly agree) and that their teachers knew them well enough to rank and grade them personally (59.2% agree/strongly agree). On the negative side they had similar levels of concern about other aspects of calculated grades as did school students in other UK countries.

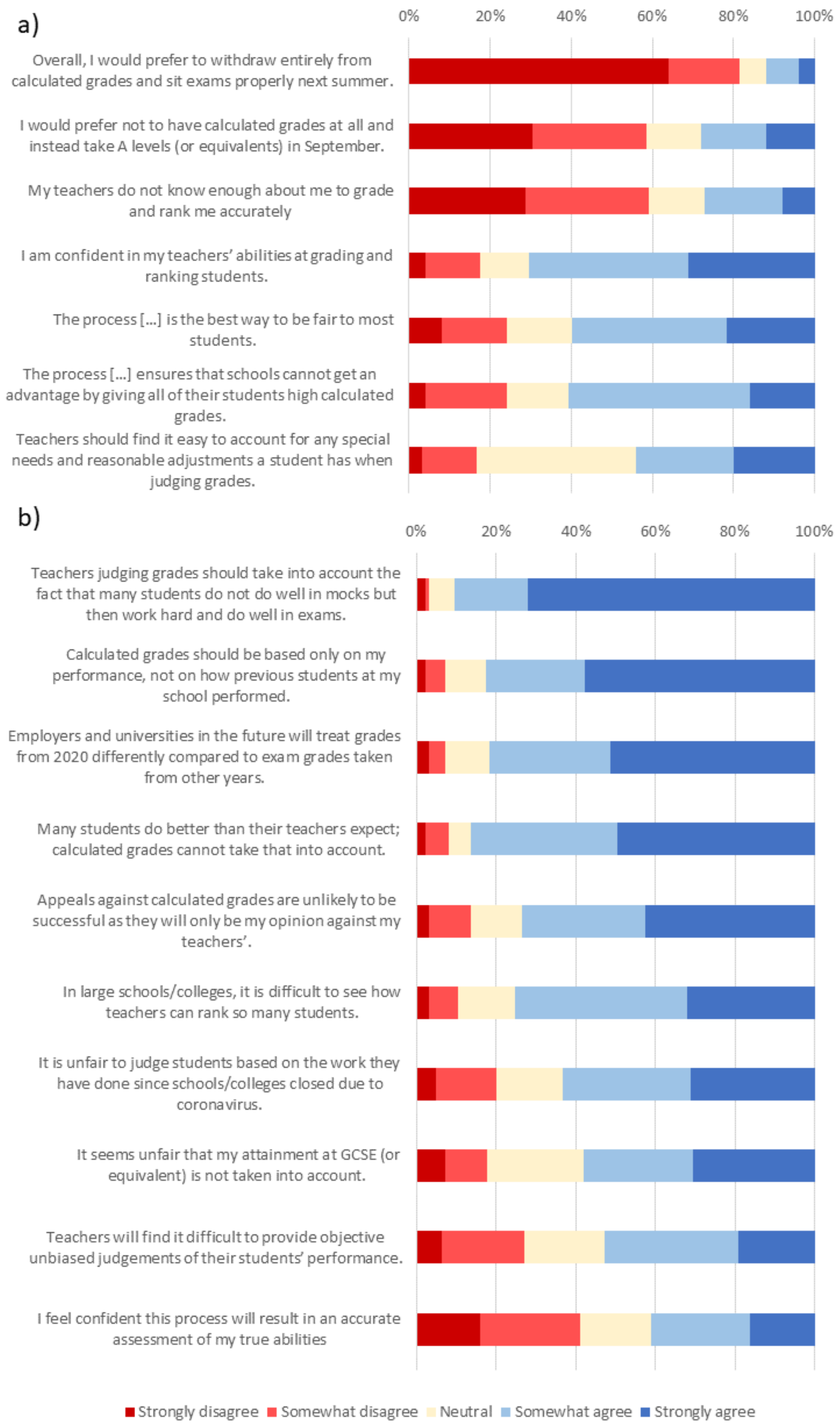

Figure S11: Aspects of calculated grades that respondents were generally more a) positive and b) negative about. Scottish S6 respondents only.

Education since the shutdown

Scottish S6 respondents used on average 2.2 (SD=1.6) educational resources provided by their school, which is fewer than those in the restricted sample. Figure S12 shows Scottish S6 used fewer of all resources compared to the restricted sample, with the exception of summative tests which they were more than twice as likely to use. Scottish S6 students were also more than twice as likely to say their school was assessing them on work since schools closed (n=37; 29.6) with a similar number (n=35; 28.0%) being unsure, and a larger proportion (n=49; 39.2%) saying they were not being assessed.

	Scotland S6	Restricted sample
Online resources	67 (59.8)	781 (71.7)
Paper resources	37 (33.6)	690 (63.9)
Online formative tests	22 (20.0)	447 (41.5)
Pastoral support	32 (29.1)	359 (33.4)
University application support	25 (23.4)	326 (30.4)
Online teaching in real time	31 (27.7)	314 (29.2)
Online summative tests	38 (34.2)	165 (15.4)
Other	<5 (<10)	37 (10.1)

Figure S12: Educational resources provided by schools used in the Scottish and Restricted samples.

Preparation for medical school/university

Scottish applicants were doing similar sorts of preparation as those in the restricted sample; although they were half as likely to be doing examination preparation (n=14; 11.2% vs n=335; 21.4%). Only 19 (15.2%) said they were not doing any preparation which meant numbers were too small to look at reasons for not doing preparation.

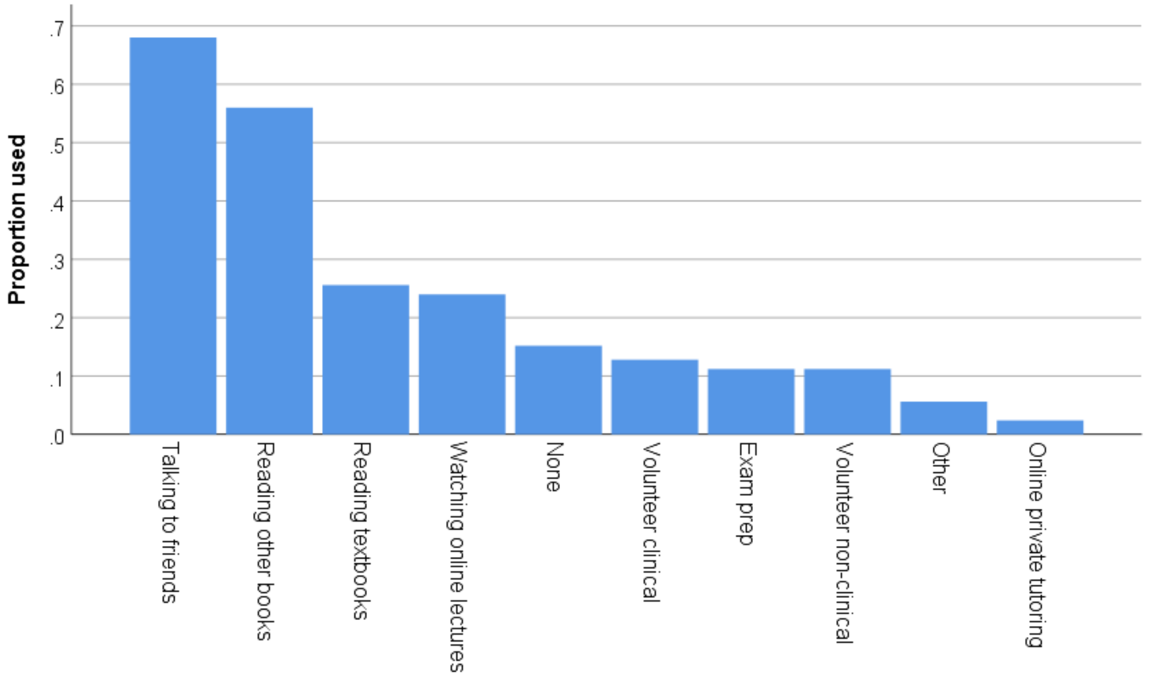

Figure S13: Proportion of respondents undertaking various activities to prepare for medical school or university. Scottish S6 respondents only.

Time spent during the lockdown

The Scottish S6 sample reported similar amounts of time spent on activities as the restricted sample.

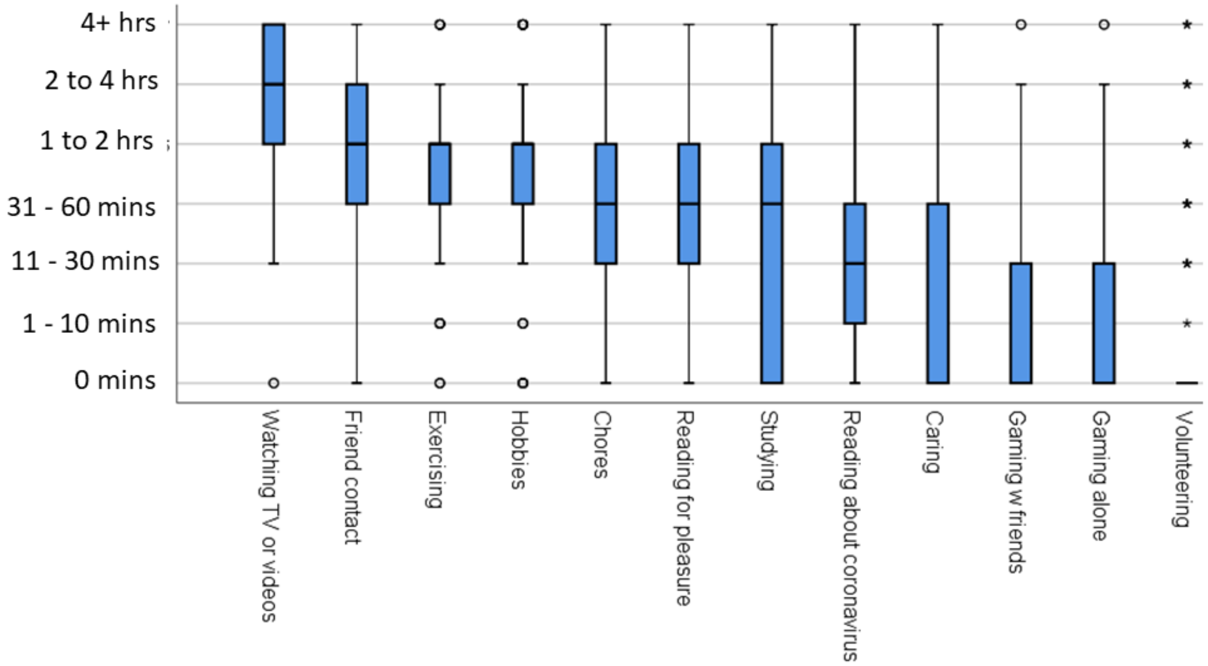

Figure S:14 Amount of time respondents reported spending on various activities during the lockdown. Scottish S6 respondents only.

For peer review only - http://bmjopen.bmj.com/site/about/guidelines.xhtml

STROBE Statement—checklist of items that should be included in reports of observational studies

	Item No	Recommendation	Page No.
**Title and abstract**	1	(*a*) Indicate the study's design with a commonly used term in the title or the abstract	2
		(*b*) Provide in the abstract an informative and balanced summary of what was done and what was found	2
**Introduction**			
Background/rationale	2	Explain the scientific background and rationale for the investigation being reported	3-5
Objectives	3	State specific objectives, including any prespecified hypotheses	5
**Methods**			
Study design	4	Present key elements of study design early in the paper	6
Setting	5	Describe the setting, locations, and relevant dates, including periods of recruitment, exposure, follow-up, and data collection	6,7
Participants	6	(*a*) *Cross-sectional study*—Give the eligibility criteria, and the sources and methods of selection of participants	6,7
Variables	7	Clearly define all outcomes, exposures, predictors, potential confounders, and effect modifiers. Give diagnostic criteria, if applicable	8
Data sources/ measurement	8*	For each variable of interest, give sources of data and details of methods of assessment (measurement). Describe comparability of assessment methods if there is more than one group	8
Bias	9	Describe any efforts to address potential sources of bias	8
Study size	10	Explain how the study size was arrived at	7
Quantitative variables	11	Explain how quantitative variables were handled in the analyses. If applicable, describe which groupings were chosen and why	7,8
Statistical methods	12	(*a*) Describe all statistical methods, including those used to control for confounding	8
		(*b*) Describe any methods used to examine subgroups and interactions	8
		(*c*) Explain how missing data were addressed	8
		(*d) Cross-sectional study*—If applicable, describe analytical methods taking account of sampling strategy	NA
		(*e*) Describe any sensitivity analyses	NA

Continued on next page

For peer review only - http://bmjopen.bmj.com/site/about/guidelines.xhtml

**Results**			**Page No.**
Participants	13*	(a) Report numbers of individuals at each stage of study—eg numbers potentially eligible, examined for eligibility, confirmed eligible, included in the study, completing follow-up, and analysed	7
		(b) Give reasons for non-participation at each stage	NA
		(c) Consider use of a flow diagram	NA
Descriptive data	14*	(a) Give characteristics of study participants (eg demographic, clinical, social) and information on exposures and potential confounders	9
		(b) Indicate number of participants with missing data for each variable of interest	9
		(c) *Cohort study*—Summarise follow-up time (eg, average and total amount)	NA
Outcome data	15*	*Cross-sectional study*—Report numbers of outcome events or summary measures	NA
Main results	16	(*a*) Give unadjusted estimates and, if applicable, confounder-adjusted estimates and their precision (eg, 95% confidence interval). Make clear which confounders were adjusted for and why they were included	10-24
		(*b*) Report category boundaries when continuous variables were categorized	9,10
		(*c*) If relevant, consider translating estimates of relative risk into absolute risk for a meaningful time period	NA
Other analyses	17	Report other analyses done—eg analyses of subgroups and interactions, and sensitivity analyses	7
**Discussion**			
Key results	18	Summarise key results with reference to study objectives	24,25
Limitations	19	Discuss limitations of the study, taking into account sources of potential bias or imprecision. Discuss both direction and magnitude of any potential bias	25,26
Interpretation	20	Give a cautious overall interpretation of results considering objectives, limitations, multiplicity of analyses, results from similar studies, and other relevant evidence	26,27
Generalisability	21	Discuss the generalisability (external validity) of the study results	26
**Other information**			
Funding	22	Give the source of funding and the role of the funders for the present study and, if applicable, for the original study on which the present article is based	7

Note: An Explanation and Elaboration article discusses each checklist item and gives methodological background and published examples of transparent reporting. The STROBE checklist is best used in conjunction with this article (freely available on the Web sites of PLoS Medicine at http://www.plosmedicine.org/, Annals of Internal Medicine at http://www.annals.org/, and Epidemiology at http://www.epidem.com/). Information on the STROBE Initiative is available at www.strobe-statement.org.

For peer review only

For peer review only - http://bmjopen.bmj.com/site/about/guidelines.xhtml

BMJ Open

The attitudes, perceptions and experiences of medical school applicants following the closure of schools and cancellation of public examinations in 2020 due to the COVID-19 pandemic: a cross-sectional questionnaire study of UK medical applicants

Journal:	*BMJ Open*
Manuscript ID	bmjopen-2020-044753.R2
Article Type:	Original research
Date Submitted by the Author:	19-Jan-2021

Complete List of Authors:	Woolf, Katherine; University College London Medical School, Research Department for Medical Education<br>Harrison, David; University College London Medical School, Research Department for Medical Education<br>McManus, Chris; University College London Medical School, Research Department of Medical Education

**Primary Subject Heading**:	Medical education and training
Secondary Subject Heading:	Health policy
Keywords:	MEDICAL EDUCATION & TRAINING, EDUCATION & TRAINING (see Medical Education & Training), SOCIAL MEDICINE, COVID-19

SCHOLARONE™
Manuscripts

I, the Submitting Author has the right to grant and does grant on behalf of all authors of the Work (as defined in the below author licence), an exclusive licence and/or a non-exclusive licence for contributions from authors who are: i) UK Crown employees; ii) where BMJ has agreed a CC-BY licence shall apply, and/or iii) in accordance with the terms applicable for US Federal Government officers or employees acting as part of their official duties; on a worldwide, perpetual, irrevocable, royalty-free basis to BMJ Publishing Group Ltd ("BMJ") its licensees and where the relevant Journal is co-owned by BMJ to the co-owners of the Journal, to publish the Work in this journal and any other BMJ products and to exploit all rights, as set out in our licence.

The Submitting Author accepts and understands that any supply made under these terms is made by BMJ to the Submitting Author unless you are acting as an employee on behalf of your employer or a postgraduate student of an affiliated institution which is paying any applicable article publishing charge ("APC") for Open Access articles. Where the Submitting Author wishes to make the Work available on an Open Access basis (and intends to pay the relevant APC), the terms of reuse of such Open Access shall be governed by a Creative Commons licence – details of these licences and which Creative Commons licence will apply to this Work are set out in our licence referred to above.

Other than as permitted in any relevant BMJ Author's Self Archiving Policies, I confirm this Work has not been accepted for publication elsewhere, is not being considered for publication elsewhere and does not duplicate material already published. I confirm all authors consent to publication of this Work and authorise the granting of this licence.

For peer review only

The attitudes, perceptions and experiences of medical school applicants following the closure of schools and cancellation of public examinations in 2020 due to the COVID-19 pandemic: a cross-sectional questionnaire study of UK medical applicants

Katherine Woolf [a,*]

k.woolf@ucl.ac.uk ORCID 0000- 0003-4915-0715

David Harrison [a]

david.harrison@ucl.ac.uk ORCID 0000- 0002-6639-6752

Chris McManus [a]

i.mcmanus@ucl.ac.uk ORCID 0000-0003-3510-4814

[a] Research Department of Medical Education, University College London Medical School, London WC1E 6BT, UK

* Author for correspondence: Dr Katherine Woolf Research Department of Medical Education, University College London Medical School, London WC1E 6BT, UK
k.woolf@ucl.ac.uk 020 3108 9216

For peer review only - http://bmjopen.bmj.com/site/about/guidelines.xhtml

Abstract

Objective

Describe the experiences and views of medical applicants from diverse social backgrounds following the closure of schools and universities and the cancellation of public examinations in the United Kingdom (UK) due to COVID-19/coronavirus.

Design

Cross-sectional questionnaire study, part of the longitudinal United Kingdom Medical Applicant Cohort Study (UKMACS).

Setting

UK medical school admissions in 2020.

Participants

2887 participants completed an online questionnaire 8th - 22nd April 2020. Eligible participants had registered to take the University Clinical Admissions Test (UCAT) in 2019 and agreed to be invited to take part, or had completed a previous UKMACS questionnaire, had been seriously considering applying to medicine in the UK for entry in 2020, and were UK residents.

Main outcome measures

Views on calculated grades, views on medical school admissions and teaching in 2020 and 2021, reported experiences of education during the national lockdown.

Results

Respondents were concerned about the calculated grades that replaced A-level examinations: female and Black Asian and Minority Ethnic (BAME) applicants felt teachers would find it difficult to grade and rank students accurately, and applicants from non-selective state schools and living in deprived areas had concerns about the standardisation process. Calculated grades were generally not considered fair enough to use in selection, but were considered fair enough to use in combination with other measures including interview and aptitude test scores. Respondents from non-selective state (public) schools reported less access to educational resources compared to private/selective school pupils, less online teaching in real time, and less time studying during lockdown.

Conclusions

The coronavirus pandemic has and will have significant and long term impacts on the selection, education and performance of our medical workforce. It is important that the views and experiences of applicants from diverse backgrounds are considered in decisions affecting their futures and the future of the profession.

For peer review only - http://bmjopen.bmj.com/site/about/guidelines.xhtml

Article summary: strengths and limitations of this study

- This is the first systematic exploration of medical applicant views on and experiences of the most significant changes to UK education in living memory due to the SARS-COV-2/COVID-19 pandemic.
- It is also the first study we are aware of that looked at university applicant views on calculated grades and the perceived impact on university admissions this year and in 2021.
- The large sample size gathered from around the UK, and the richness of the data provides insight into differences in the experiences and views of different socio-demographic groups, after controlling statistically for educational attainment.
- It is uncertain how representative our sample is of all medical applicants; medical applicants are not representative of all university applicants in either academic or socio-demographic terms and generalisations from our findings to all applicants should only be done with extreme caution.
- At the time of writing it was not possible to include data on participant examination scores or applications and acceptances to medical school; however this follow-up is planned.

Introduction

The UK Medical Applicant Cohort Study (UKMACS) is a study of United Kingdom (UK) medical school admissions. It is primarily a longitudinal questionnaire study of UK residents who in the summer and autumn of 2019 were seriously considering applying to study medicine in the UK for entry in 2020. UKMACS questionnaire data are subsequently linked to administrative data on all UK medical applicants held within the UK Medical Education Database (www.ukmed.ac.uk). Wave 1 data were collected between May and September 2019 and asked how applicants from different backgrounds were choosing which medical schools to apply to. Wave 2 data were collected from November 2019 to January 2020 and asked which medical schools and universities participants had applied to and how they had made their choices.

In March 2020 it was announced that UK schools would close and A-level (and equivalent public examinations) would be cancelled due to the coronavirus/COVID-19 outbreak in the UK. This was one of the most major disruptions ever to affect education and university admissions in the UK and was very significant for the UKMACS cohort, who are mostly in their final year of schooling and were due to sit examinations in the summer of 2020.

We therefore administered an additional unplanned UKMACS questionnaire to understand what medical applicants were experiencing in terms of education, their views on how grades would be awarded following examination cancellations, and their views on how medical schools might respond with regard to admissions policies. We particularly sought to understand how applicants from diverse social backgrounds might differ, with the aim of facilitating the inclusion of applicant perspectives and experiences in discussions about changes to medical school admissions and medical education.(1)

Calculated grades

The absence of A-levels and other equivalent public examinations in March 2020 meant that alternative methods of assessment for candidates had to be found, not least as A-levels are "the single most important bit of information [used in selection]" by universities.(2) On April 3rd *Ofqual* (Office of Qualifications and Examinations Regulation) in England announced that exams under its purview in England would be replaced by calculated grades based on teachers estimation of the

For peer review only - http://bmjopen.bmj.com/site/about/guidelines.xhtml

grades that their students would have attained and the ranking of each student within grades[1], which would then be standardised centrally.(3) The Scottish Qualification Authority (SQA) and other national bodies also announced similar processes for their examinations.

Performance in A-level examinations has long-term impacts (4, 5), which makes changes to how grades are awarded potentially very significant. The use of calculated grades raises many questions, some of which were summarised in a letter to *The Guardian* by Yasmin Hussein, a GCSE student who said that,

> *"… the … exam hall [is] a level playing field for all abilities, races and genders to get the grades they truly worked hard for and in true anonymity (as the examiners marking don't know you). [… Now we] are being given grades based on mere predictions." Yasmin Hussein, letter to The Guardian, March 29th 2020.(6)*

Among teachers, survey data suggests that there are doubts about the accuracy and fairness of calculated grades, with 39% saying that all students would get a fair deal, 24% saying they would not, and 37% not knowing or not answering. There were also doubts about fairness for students from Black Asian and Minority Ethnic (BAME) backgrounds, about those working hard in the last weeks before an exam being penalised, about teacher 'favouritism', although there were teachers who commented that the process is as fair as possible under the circumstances.(7)

University applicants also have concerns. In a survey carried out by HEPI (Higher Education Policy Institute) before the details of calculated grades were announced, but after it was known that grades would in some way be predicted, 27% thought that their predicted grades were worse than they were likely actually to have attained, compared with 13% thinking their predicted grades were better than they would actually attain.(8)

Another survey of 511 university applicants (including 452 A-level students) conducted for the Sutton Trust found that just under half believed the new A-level grading system would result in their receiving poorer grades but working class respondents were more worried about large negative consequences compared to middle class students. Nearly three quarters believed the new system was less fair than examination grades and this was more of a concern for applicants from *higher* socioeconomic backgrounds. Nearly half of applicants felt the COVID-19 crisis would impede their chances of getting into their first choice university, a more common concern among working class respondents.(9)

The impact on medical school admissions of examination cancellations and their replacement with calculated grades is, at the time of writing, still not completely clear. *Ofqual* stated that,

> *"The grades awarded to students will have equal status to the grades awarded in other years and should be treated in this way by universities, colleges and employers. On the results slips and certificates, grades will be reported in the same way as in previous years".(3), p.6.*

The decisions of *Ofqual* in this case are in effect governmental decrees, supported by Ministerial statement, and universities and other bodies will therefore abide by them, as was affirmed by the Medical Schools Council on 5th May 2020.(10) That does not mean however that other factors were not needed to be taken into account in some cases, as for instance when applicants did not attain the grades needed for their conditional offers, or for applicants in clearing. Furthermore in guidance

---

[1] For example, if a teacher has 30 Chemistry A-level students, they would estimate the grade each student would get. Then the teachers ranks students within grades, so for example if they have 5 students estimated to get an A grade, they rank those 5 students.

updated on 1st May 2020 the Government stated that “if a student does not feel their grade reflects their performance, they will have the opportunity to take an exam in the autumn”(11) with *Ofqual* expanding on 15th May 2020 that “students will be able to use the higher of the two grades for future progression.”(3) This raises questions for university admissions, as Medical Schools Council acknowledged in their statement of 5th May 2020:

> *“There are a number of issues that the education sector as a whole is yet to resolve. These include how appeals against calculated grades will work across the UK and when students will be able to sit exams if they are unhappy with their calculated grade. The impact of these issues on medical admissions is unclear but medical schools are actively engaging in these discussions and are working hard to develop solutions that are fair to applicants.”(10)*

Education during the pandemic

As well as examinations being cancelled, UK schools closed on 20th March 2020 to all except the children of key workers and vulnerable children with secondary schools mostly closed until September 2020. Similarly in mid-March 2020 many universities suspended face-to-face teaching for the academic year 2019/2020.

The impact of school closures on student learning and outcomes will be significant (12-14) and it may be particularly problematic for those from poorer backgrounds and/or at state-funded schools. The Institute of Fiscal Studies analysed survey data from a weighted sample of over 4000 parents with children aged between four and 15 years old in May 2020 (15). Among secondary school children, those from the richest quintile were spending on average slightly over an hour more per day on learning compared to those in the poorest quintile, amounting to several weeks more learning over the course of the time schools are closed. In particular children in the richest families were spending significantly more on educational activities provided by schools and from private tutors. Even among state school pupils, children from the richest families reported greater access to face-to-face online teaching, which the authors argue is likely to be of higher educational value than other resources that require more parent input, particularly since the poorest parents of secondary school children were less likely to find it easy to support their child’s home learning.

The results of the IFS report chime with data from *Teacher Tapp*, an ongoing weighted survey of several thousand teachers in England.(16) At the start of the lockdown (23rd March 2020) private school secondary schools were much more likely than state secondary schools to be using online videoconferencing (27% vs 2%) and online chat (18% vs 3%). The above-mentioned Sutton Trust report (9) also found socioeconomic differences in access to “internet access, devices for learning or a suitable place to study” and differences in the amount of A-level teaching being conducted by teachers at private and state schools.

Among those secondary school pupils who had applied to university, the Sutton Trust report authors argued that students from lower socio-economic backgrounds are also likely face additional disadvantages both with their university applications and when starting university:

> *“Given the uncertainty caused by these changes [to education resulting from COVID-19], university applicants are likely to need more support than ever to navigate the process [of applying to university]. This will be even more important for young people from lower socio-economic backgrounds, who are less likely to be able to draw on the advice of family members with higher education experience themselves. But with schools closed for most pupils, it may be difficult*

For peer review only - http://bmjopen.bmj.com/site/about/guidelines.xhtml

for applicants to get the help they need. Similarly, there's also a danger that this year's applicants will miss out on A level content during the lockdown [...]. For disadvantaged students about to go on to higher education, this could leave them with gaps in their knowledge base, putting them behind their peers before they have even begun at university." [p1. (9)]

The present study

This study aimed to explore and describe perceptions of calculated grades, of student selection more generally, and of educational experiences during school and university closures, in a large group of medical school applicants, who were typically high-attaining students. A range of background factors were assessed to determine how perceptions differed according to demographic and other measures. Data collection took place between April 8th and April 22nd, which was about two and a half weeks after school closures.

Methods

Study design

Cross-sectional questionnaire study, which formed part of the longitudinal UK Medical Applicant Cohort Study.

Eligibility

To be invited to complete the questionnaire, participants had to have registered to take the University Clinical Admissions Test (UCAT) in 2019 and to have agreed to be invited to take part in UKMACS, or they needed to have completed one or more previous UKMACS questionnaires[2]. They also need to have been seriously considering[3] applying to study medicine in the UK for entry in 2020, and be resident in the UK or Islands/Crown Dependencies.

Participants were not invited if they had previously requested their data be removed from the UKMACS database, had asked not to be contacted for further research, or had not consented to having their personal information retained by the research team or linked with other information for research purposes.

Questionnaire development

During the development of the questionnaire *Ofqual* announced that calculated grades would be awarded. We therefore assessed perceptions of how calculated grades would be awarded and used, and of other possible methods medical schools could use to select or reject offer-holders. We also about potential knock-on effects of calculated grades in the 2021 application cycle, and whether medical schools should open online or defer opening until teaching could be done face-to-face. We asked about use of educational resources and preparation for university/medical school, and about the time they were spending on various activities. We included self-reported measures of academic attainment and socio-demographic measures used in previous UKMACS questionnaires, as well as the 15-item Big Five personality measure used in the national longitudinal cohort study *Understanding Society*. (17) Personality traits are "relatively enduring styles of thinking, feeling, and

---

[2] Wave 1 of the UKMACS questionnaire was administered between May and October 2019; Wave 2 between November 2019 and January 2020.
[3] Participants were thought to be seriously considering applying if they had registered to take UCAT. Wave 1 of the questionnaire also asked them to confirm they were seriously considering applying to study medicine.

For peer review only - http://bmjopen.bmj.com/site/about/guidelines.xhtml

acting".(18) It is generally agreed that there are five distinct personal traits or factors: Neuroticism, Extraversion, Openness to Experience, Agreeableness, and Conscientiousness.

Most questions were designed specifically for this questionnaire since they asked about unprecedented events and validated items were not available. We constructed the questionnaire with JISC Online Surveys [https://www.onlinesurveys.ac.uk/] and piloted the questionnaire and information sheet with two current applicants to medical school. Amendments were made in response to feedback from the applicants and from Medical Schools Council. A copy of the questionnaire is included as Supplementary File 1.

Questionnaire administration

Participants were sent an email invitation and link to the current questionnaire on the afternoon of 8th April 2020. 18,665 invitations were sent, with up to two email reminders and two text message reminders. The questionnaire closing date was 20th April 2020, with responses accepted up to 22nd April 2020.

Statistical analysis

Descriptive and univariate analyses were run on SPSS v26. Imputation of missing data and multivariate analyses were run on R.

Factor analysis on the 87 attitudinal variables was carried out using the *psych* package in R (19) with *fa.parallel()* and *nfactors(),* being used to determine the number of factors.

Freetext question answers

All answers to freetext questions were read by the research team, and illustrative quotes selected to aid understanding of quantitative results.

Patient and public involvement

Patients and the public were consulted in the development of the questionnaire.

Results

Participants

3071 participants completed the questionnaire, of whom 2904 stated they were eligible to take part (i.e. seriously considering applying to study medicine in the UK in 2020 and resident in the UK or Islands/Crown Dependencies). After removing 16 respondents who did not consent to have their data analysed and 11 duplicates, there were 2877 valid cases for analysis, which is 15% of those invited. This is subsequently referred to as the full sample. See Figure 1.

The main analyses were performed on a restricted sample of 1562 respondents currently in Year 13, who had applied to medicine for entry in 2020, with at least three predicted A-levels and no achieved A-levels. Results are also reported in Supplementary file 2 for respondent groups excluded from the restricted sample, notably those living in Scotland and those not currently in Year 13 – see Supplementary file 2.

Missing data

The analysis considered 120 measures in the restricted sample. The questionnaire asked about attitudes to 87 different topics concerning medical school entrance. Of 153,076 data points, 10788

For peer review only - http://bmjopen.bmj.com/site/about/guidelines.xhtml

(7.2%) were missing. For the individual variables, the median percentage of missing data values was 0.48%, with 75 measures having fewer than 5% of missing values. The questionnaire also asked about demographic and educational items. For 12 demographic measures, 462 of 18744 measures were missing (2.5%), with a median of 1.0% per measure, and 11 measures having fewer than 5% missing values. For further details on missing data, please see Supplementary file 2.

Demographics

Demographics for the full and restricted samples are reported in **Table 1**, where details of categories within demographic variables can also be found.

Education and achievement

Predicted A-levels

A-level grades were scored as A*=12, A=10, B=8 etc, and those reported as being between two grades as intermediate e.g. A*/A =11, A/B = 9, etc. Mean predicted A-level grades were calculated for the top three grades regardless of subject (*Mean top three predicted* A-levels), and for all grades (*Mean predicted* A-levels). *Mean top three predicted* A-levels was 10.89 and *Mean predicted* A-levels was 10.71.

UCAT, BMAT, GAMSAT

1546 participants (99.1%) reported having taken UCAT; 765 (49.0%) reported having taken BMAT; and none reported having taken GAMSAT. Of the 1350 participants who reported a total UCAT score that was greater than 1799 and less than 3601, the mean score was 2660 (SD=235).

GCSE

GCSE grades can range from 1 to 9. A variable *Mean GCSE* was calculated by dividing the total GCSE points by the number of GCSEs taken, and the mean was 7.91 (SD=0.71).

Relationships between educational measures

UCAT score correlated with *Mean top three predicted* A-levels at 0.418 (p<.001) and with *Mean GCSE* at 0.487 (p<.001). *Mean GCSE* and *Mean top three predicted* A-levels correlated at 0.611 (p<.001).

Participants at non-selective state schools had lower scores on all attainment measures (*Mean GCSE*: difference=0.3 points, p<.001; *Mean top three predicted* A-levels: difference=0.23 points, p<.001; UCAT score: difference=89 points, p<.001).

Medical school offers

1292 (85%) respondents had applied to four medical courses, 1289 (82.5%) had at least one offer, 177 (11.3%) had four offers, and 204 (13%) were waiting to hear from at least one medical school at the time of completing the questionnaire.

Respondents who did not have a parent/carer with a university degree were less likely to have a medical offer (78.1% vs 85.0%; p=0.001), as were those without a parent/carer in the highest socioeconomic group (79% vs 85%; p=0.002) Male participants were slightly less likely to have an offer (80% vs 85%; p=0.049).

For peer review only - http://bmjopen.bmj.com/site/about/guidelines.xhtml

For peer review only

Applicant views on admissions

Perceptions of the fairness of methods medical schools could consider using in the selection of offer-holders

Participants were asked to rate the fairness of 17 measures, including calculated grades, that medical schools could potentially use to decide to accept or reject offer-holder following exam cancellations. Rating categories were: "Unfair: should not be used" "Quite unfair: avoid if possible" "Quite fair: could be used in combination with other measures" "Very fair: could be used alone", with a freetext question asking for additional comments and suggestion.

No measure was felt by a majority of participants to be fair enough to use on its own. The measure considered most fair was *Exam grades taken in September 2020 (if these take place)* (32.3% very fair), followed by *Predicted Grades declared on UCAS application* (26.2% very fair), *Calculated grades* (22.6% very fair), *GCSE grades* (20.4% very fair) and *Score at interview* (19.5% very fair).[4]

Several methods were felt by a majority to be fair enough in combination, particularly *Predicted grades* (80.6%), *GCSE grades* (73.8%), and *Score at interview* (73.4%); but only a fifth (20.3%) of participants felt *Attendance at widening participation activities* was quite fair or very fair. See Figure 2.

Multiple regression results showed that after taking account of all other educational and socio-demographic variables, BAME participants were more likely to perceive *Exams taken in September 2020*, *UCAS personal statement*, and *Personal background* as fair to use, and respondents from deprived areas were more likely to perceive *Personal background* and *Attendance at widening access programmes* as fair to use. *Calculated grades based on mock exams, coursework etc, and awarded in place of final examination grades* were perceived as less fair by those with lower predicted A-levels.

There were 154 freetext responses (10%), with participants elaborating on their responses or suggesting alternatives:

> *"A combination of the most objective information that every offer holder will have, ie GCSEs, UCAT or BMAT, interview score, etc"*

> *"A standardised form of assessing all medical applicants would be the best way to allocate existing places. […] Since we do not have standardised A level grades, places should be offered using the UCAT as this is the fairest way of distributing places to the most able students."*

> *"Using interview scores and UCAT scores in combination are independent measures, and are more fair than using calculated grades which have the potential to be biased."*

> *"Anything including personal statement, BMAT or UCAT I would argue are unfair to use as judgement as there will definitely be a bias in terms of how certain students achieved their grade. I believe the fairest way to determine ones overall*

[4] See Figure 2 for full item wording

For peer review only - http://bmjopen.bmj.com/site/about/guidelines.xhtml

> *grade would be to use their GCSE data with a combination of evidence throughout the two years of A levels."*

Other measures participants mentioned included: an additional university assessment (written, viva or project/portfolio-based) now or at the start of the academic year, an additional interview, selection at the end of Year 1/make first year a foundation year, additional reference from teachers/school, reference from work experience, school/college attendance record, distance from university, extenuating circumstances, self-reported use of time during quarantine/lockdown, number of offers received, prioritise those with higher degrees, prioritise those already working in the NHS, extra-curricular achievement (e.g. music, Duke of Edinburgh's Award), school's prior achievement. For example:

> *"NHS experience ie patient facing health professional ie years and grade, other non technical skills, education background ie. science, post graduate achievement ie MSc particularly if in science or medical subject and grade achieved. Also emphasis on the candidates as a whole ie well rounded personality (potential to communicate well) rather than typical A Grade student. Letter of recommendations from medical consultant whom candidates may have worked closely with."*

> *"Another interview possibly over the phone to see what students have done with their time in quarantine (ie, volunteering in a care setting or hospital / working in a hospital / exploring other interests)"*

> *"Each university could form their own selection test similar to UCAT/BMAT with a brief guidance/specification on what will be on the test given out to offer holders so they have some time to revise for it, but this should be used in combination with other details (e.g. if offer holder's calculated grade was only 1 grade below what was required for entry)"*

> *"I think a combination of previous results, any exams that do go ahead (at some point whether that is this summer or later), alongside medical applications, relevant work experience (as per personal statement and any other forms detailing this) and the applicant interview. Also potentially the medical schools could generate online admissions tests for students with conditional offers to generate a clearer view of a students capability and ability to comprehend and withstand the pressures of medical school. But any tests generated by the medical schools must be used alongside the other parts of the applications to ensure fairness."*

Participants were asked whether they had heard anything from medical schools/universities they had applied to about how selection might be impacted by examination cancellations; among those holding conditional offers, a minority (n=538; 42%) said they had heard from at least one medical school/university they had applied to.

Acceptability of options for dealing with a situation in which more students meet their offers than there are medical school places

Participants were asked to rate the acceptability ("completely unacceptable", "slightly unacceptable", "neutral", "slightly acceptable", "completely acceptable") of a number of options

For peer review only - http://bmjopen.bmj.com/site/about/guidelines.xhtml

that medical schools could use if they had more students meeting offers than they had places, with a freetext question asking for additional comments and suggestions.

The most acceptable option was *Ask some applicants with offers to volunteer to defer a year*. The only other acceptable option was *Accept all applicants whose calculated grades meet the conditional offer, although it could mean fewer resources per student*. See **Figure** 3.

Multiple regression analyses showed no significant differences by social or demographic group on these items.

There were 187 freetext responses (12%). Several respondents suggested that medical schools should receive more funding to manage larger cohorts and create more doctors, e.g.:

> *"Deferring of one year should not be taken into consideration as this would damage applications of next year. Ask the government to invest more money on the NHS and allow to have more spaces. All these problems could be solved if exams were taken virtually."*

> *"The government could also provide more funding for medical schools- not only will this allow more people to attend but it will also mean there are more doctors down the line who can work in the NHS."*

There were suggestions that applicants could opt to attend other medical schools they had applied to but which they had not selected as their firm or insurance choice, or that they could be offered places at medical schools they had not applied to:

> *"If some medical schools have a lower numbers of applicants overall, compared to others, redistribute some students to these ones, with permission."*

There were many suggestions of incentives to defer, and some felt that they would welcome a year off before starting:

> *"Incentives to defer like 1 yr free accommodation or £5000 or student ambassador job for gap year"*

> *"Incentive to deferring such as free university accommodation for the first year, organised work experience placements and or organised care assistant jobs for the gap year."*

> *"If people are asked to volunteer to or forcefully defer entry, offering alternatives for work they could do within a healthcare setting for that year. For example, maybe clerical work within the NHS so they're still immersed within the healthcare system.*

> *"Asking students to voluntarily defer a year would be a popular option, I think many people will reevaluate their priorities over the coming months and may appreciate the opportunity."*

> *"The option to defer is definitely an option that should be considered as many people would be happy with the idea of gaining more medical experience in the year out that they would now have."*

For peer review only - http://bmjopen.bmj.com/site/about/guidelines.xhtml

For peer review only

There were suggestions medical schools could have multiple cohorts either all starting in October or one cohort starting in October and another cohort starting early 2021.

> *"Create an extra group/year for Covid Students to manage the numbers"*

> *"Maybe consider having staggered starts throughout the year October start January start June starts."*

> *"Stagger the course to offer two presentations and alter the following academic term holidays if possible"*

Respondents also expressed concern as to the impact of the present disruption on next year's admissions cycle and available resources:

> *"The selection process should not be biased towards those rejected this year, next year, and should not change for the next cohort."*

> *"I hope that this year's or next year's applicants will not be disadvantaged due to these unprecedented circumstances."*

Perceptions of potential impact on admissions for 2021

Participants were asked to rate how much they agreed or disagreed with six options as to how medical schools could deal with the potential impact of the current situation on admissions in 2021. See **Figure** 4.

In general, respondents felt medical schools should give special consideration to current applicants reapplying next year (67.1% agreed/strongly agreed that *Applicants rejected this year should be given special consideration when re-applying next year*) however opinions were divided about what that special consideration should consist of.

Multiple regression analyses showed that after accounting for number of offers, educational, social and demographic factors, BAME respondents were more likely to feel that re-applicants should be given some advantages.

Starting academic year 2020/2021

A majority of respondents (n=952, 61.1%) believed that if necessary, medical schools should *Defer the start of the academic year only when face-to-face teaching is possible* with 605 respondents (38.9%) believing that medical schools should *Start the academic year on time using distance learning for as long as is necessary*. This did not vary significantly by prior attainment, number of offers, or educational/social/demographic background.

Education and university preparation

Calculated grades and the perceptions of process of awarding calculated grades in lieu of examination grades

Participants were generally fairly ambivalent towards calculated grades. On the positive side (see **Figure** 5 **Error! Reference source not found.a**), the majority of respondents (78.6%) preferred calculated grades to taking examinations next year, and about half (54.9%) preferred calculated grades to taking examinations in September 2020. Over half (59.3%) agreed that schools wouldn't be able to game the process to award all their students high grades, and 51.4% felt that the process of awarding calculated grades was the best way to be fair to most students in the circumstances

For peer review only - http://bmjopen.bmj.com/site/about/guidelines.xhtml

(although 35.0% disagreed). Over half (56.4%) agreed that their teachers were generally able to rank and grade students accurately, however respondents were divided as to whether their own teachers knew them well enough to grade and rank them accurately: 42.0% agreed their teachers did NOT know them well enough whereas 44.6% thought their teachers DID know them well enough.

On the negative side (see **Figure b**), over half of respondents (52.9%) disagreed or strongly disagreed that calculated grades would result in an accurate assessment of their abilities, with 63.4% agreeing that teachers would find it hard to be unbiased, 80.7% agreeing it was difficult to see how teachers in large schools can rank so many students and 85.5% agreeing calculated grades cannot take into account students doing better in exams than their teachers expected. Most agreed it was unfair to judge students on work done since schools/colleges closed (70.4%), that grades should be based solely on their performance and not the performance of previous students at their school (69.6%), and that it was unfair their GCSE performance was not taken into account (68.7%).

Mean top three predicted A-level points was a major predictor of perceptions of calculated grades but there were also differences by background after accounting for prior attainment, number of offers and other educational/social/demographic factors: BAME respondents and female respondents were more negative about calculated grades and respondents from non-selective state schools and those from more deprived areas were more likely to agree that calculated grades should not take into account the performance of previous pupils at their school. See **Table 2**.

There were 398 freetext responses to the following request for further comments at the end of the questionnaire: "Please use this space for any additional comments you wish to make about the questionnaire or selection of medical students". These responses included concerns that calculated grades would be based on work completed early in the academic year and on mock exams created and assessed by the school. It was felt that these measures would not take into consideration the development and academic progress made by pupils over the year, even when teachers gave special consideration to the impact of the disruption. There was also concern that at the time of mock exams in particular, many medicine applicants were more focused on admissions tests (BMAT in particular), submitting applications and preparing for interviews.

> *"Grade calculations took away the chance the students had to prove themselves (final exams) and their control. Basing the final grade on a time when the students weren't aware that they were being truly assessed can hardly be classed as fair."*

> *"I believe universities should be lenient and realise that if a students calculated grade is below their conditional offer, this is not 100% representative of the students abilities. If they were able to secure an offer in the first place then universities should already know the academic capabilities of said student through their GCSE grades, predicted grades, UCAT/BMAT scores, teacher references, interviews etc. Otherwise, they wouldn't have given the student an offer. Where possible, every offer holder should be given their place at university in this academic year, whenever it resumes and should not be forced to take a year out and spend that year being stressed, lost and demotivated."*

With teacher submitted grades then being subject to standardisation by the exam boards based on previous achievement from a school was a concern for this student:

For peer review only - http://bmjopen.bmj.com/site/about/guidelines.xhtml

"I am the only student in my year and the third student in my sixth form's history to ever apply for medicine, and the first to receive all 5 offers. My school historically is one that does not do very well and I fear that my individual success and all the hard work I have had to do on my own as I get no help from my school, will be overshadowed by the bad results from previous years."

Education since the shutdown

A minority of respondents said their school was planning on formally assessing them on work done since the shutdown (n=184; 11.8%); nearly half (n=740; 47.5%) said their school would not, and over a third (n=614; 39.4%) were uncertain. Respondents attending a private/selective school were twice as likely to report being assessed on work since the shutdown (14.2% vs 7.6%; p<.001).

Participants were asked whether they were using educational resources provided by their school/college and if not why not. Nearly all respondents had used at least one resource (n=1346; 91%) and three was the average number used.

Respondents attending private/selective schools were more likely to report having used all educational resources except support for university applications, and those at non-selective state schools used on average two resources compared to the three used by those at private/selective schools. The largest difference was in the use of online teaching in real time, which those at private/selective schools were nearly four times more likely to have used. See **Table 3**.

In the multivariate analyses, attendance at a private/selective school was an independent predictor of using online teaching in real time, online resources for home learning, online formative assessments, and paper resources for home learning, even after controlling for prior attainment and socio-demographics. In addition, having at least one parent/carer with a university degree was an independent predictor of using paper resources for home learning, and having lower UCAT/BMAT scores was an independent predictor of using online teaching in real time.

Those who had not used educational resources reported the main reason(s) were either that the resources were not available or that they felt they did not need to use them. Only very few said they had not used a resource because of a lack of private quiet space, lack of time, lack of internet/computer access, or because they were finding it too hard to focus. Those at non-selective state schools were more likely than those at private/selective schools to state lack of availability as a reason, and less likely to state not needing to as a reason– see **Table 4**.

Preparation for medical school/university

Participants were asked what preparation if any they were doing for university or medical school – see **Figure** 6.

Of the 207 (13.3% of the sample) who said they were not doing any preparation, the most common reason was that they were too worried and couldn't focus (n=88; 42.5% of those not doing any preparation), not having resources (35.5%), feeling it wasn't necessary (29.5%), caring for others (13.5%), not going to university this year (14.0%), not having time (6.3%), and being unwell (4.8%). Respondents could select multiple reasons.

For peer review only - http://bmjopen.bmj.com/site/about/guidelines.xhtml

Time spent during the lockdown

Participants were asked to state how much time they were spending on various activities in the previous five days – see **Figure** 7. The multivariate analysis showed that respondents from private/selective schools reported spending more time studying, even after controlling for prior attainment and socio-demographic factors.

Factor analysis

Number of factors

The factor analysis included 87 variables which are attitudinal or related to attitudes. The maximum eigenvalue was 6.99, with 27 eigenvalues greater than 1. A scree-plot suggested that there was a break at or around 6 factors (see **Figure** 8). Other criteria were very variable, with *fa.parallel()* in the *psych* package in *R* suggesting there were 19 principal components with eigenvalues greater than the 95% upper confidence interval for randomly generated data. *nfactors()* in *psych* said that VSS complexity 1 suggested 17 factors, VSS complexity 2 suggested 17 factors, Velicer's MAP gve 10 factors, Empirical BIC gave 20 factors, and Sample Size Adjusted BIC gave 20 factors. However the output also reported, "Although the vss.max shows 17 factors, it is probably more reasonable to think about 4 factors". Overall there are probably many small factors corresponding to measures with low communalities and hence mostly unique variance. For present purposes we are particularly interested in aggregating measures to gain more statistical power, and therefore we chose to extract 6 principal factors, which corresponds with the break in the scree slope, and is a little larger than the *nfactors*() recommendation of 4.

Naming of factors

The six factors were named as following, by considering the highest absolute loadings, along with all loadings over 0.35:

1 '*Lack of confidence in calculated grades*'. Positive loadings (n=9 items) reflected concerns that teachers will not know students well enough and will find it hard to be objective, preferring not to have calculated grades and take exams in September or next summer, and appeals being unlikely to be successful. Negative loadings (n=5 items) reported confidence in the process resulting in an accurate reflection of a student's true ability, and the awarding process being fair to most students. High positive scores therefore represent **a lack of confidence in the process of determining calculated grades**.

2 '*Special treatment next year for rejected applicants*'. High positive loadings (n=6 items) were associated with medical schools needing to give higher priority and special consideration next year to students rejected this year, with rejected candidates being automatically given conditional offers next year. Negative loadings (n=4 items) suggested that re-applicants next year should be treated in the usual way, and special treatment for rejected applicants this year would not be fair for first year applicants next year. High positive scores therefore suggest that **applicants who are rejected this year should be treated specially next year**.

3 '*Other selection measures to be taken into account*'. A small group of items (n=3) suggested that selection could take into account aptitude tests such as UCAT, BMAT, and performance at interviews. High scores therefore suggest that where possible, **measures other than calculated grades should be taken into account**.

For peer review only - http://bmjopen.bmj.com/site/about/guidelines.xhtml

4 '*Preparing for medical school*'. High positive loadings (n=4 items) reflected applicants who during lockdown were preparing for university by reading (either textbooks or other books), were watching online lectures, as well as talking with friends. Negative loadings (n=4 items) reflected applicants who were not doing any preparation, didn't feel preparation was necessary, didn't have any resources, or who couldn't focus because they were too worried. High scores therefore indicate an applicant's **concentration on preparing for medical school or university**.

5 '*Importance of background and experience*'. All high loadings (n=8 items) were positive and indicated that medical schools should take into account work experience, the applicant's personal statement, and the teacher's reference on the UCAS form, attendance at university summer schools and widening participation programmes, an applicant's personal background such as being from under-represented groups, and other grades in qualifications such as GCSEs and the Extended Project Qualifications. Overall higher scores indicate that **a wider range of measures should be used to take into account personal background and wider experience.**

6 '*Resources from school for home study*'. All loadings were positive (n=8 items), and indicated that applicants were being provided with live online teaching, online resources for home learning, paper resources such as workbooks, formative online assessments, and summative online assessments that might count towards calculated grades, doing timed essays or past papers, and spending more time studying. Higher scores therefore indicate **having received greater support for home schooling from schools and colleges**.

Predictors of factor scores

Predictors of factor scores were assessed using multiple regression. All predictor variables in the set were entered and only those achieving $p<.01$ are reported. All predictors therefore take into account the effects of others in the set. Set A is the basic set used earlier in the study. Set B is extended by including socioeconomic group (based on parents' jobs), doctor parent(s) and the five Big Five personality factors, and are included on an exploratory basis. See **Table5.**

Summary and conclusions

Summary of results

No single measure, including calculated grades, was considered fair enough by most applicants to use in the acceptance or rejection of offer-holders; however many applicants considered calculated grades – and many other measures - fair enough to use in combination with other measures such as interview scores or admission test scores. Taking into account personal background or widening participation attendance was considered fairer by BAME applicants, those from deprived areas, and those without degree-educated parents.

Many respondents had concerns about calculated grades, especially BAME and female applicants who felt teachers would find it difficult to grade and rank students accurately, and those from non-selective state schools and living in deprived areas were more concerned about the standardisation process that uses the attainment of previous pupils at a school. Despite this, the majority would rather have calculated grades than forgo calculated grades completely and take examinations in Autumn 2020 or Summer 2021 instead.

Respondents mostly felt that medical schools should admit any applicant who met their conditional offer, even if that meant having to increase the number of places (which would require a legal change and increased government funding), although there was also acceptance of medical schools asking for volunteers to defer but not of requiring deferrals. Respondents were divided as to how

For peer review only - http://bmjopen.bmj.com/site/about/guidelines.xhtml

rejected applicants should be treated if they were to reapply next year, with some respondents feeling they should be treated no differently and others feeling their 2020 experience should be taken into account. A majority of respondents tended to favour medical schools delaying the start of term until face-to-face teaching were possible.

Applicants from non-selective state schools reported using fewer educational resources than their counterparts at private or selective schools, and in particular they reporting less online teaching in real time, and spending less time studying during the lockdown.

Comparisons with other research

Our findings show many similarities to other recent UK studies of the effects of the COVID-19 pandemic on education in the UK (8, 9, 15, 16) however it is notable that in this sample of medical applicants ethnicity is more significant than socioeconomic factors in predicting concerns about calculated grades. A study of A-level students, conducted by Bhopal and Myers between April and August 2020 and published as a report on the OSF open access repository, surveyed an ethnically diverse sample of 583 A-level students in Britain and interviewed 53 students about their views on their education during the pandemic and their exam results. The authors report that 21% of students were glad exams had been cancelled but over twice as many (46%) would prefer to sit exams, which is similar to our finding that exams were considered the fairest method of selection. Similarly to our findings, the authors report that "Many students also raised concerns their ethnicity could influence how teachers assessed their work" quoting a Black student saying "Some of my teachers seem biased […] They always think the Black boys are trouble", an Irish Traveller student saying "We're Travellers. The school doesn't think much of us." and an Indian student saying "My teachers don't think I can do that well […] They also have their favourites, we can all see that – those students who they think should do well, are not those who necessarily will do well". This reflects concerns from the Black Asian and Minority Ethnic participants in our study about teacher bias.
.

It is known that predicted grades are lower for some minority ethnic groups (20) and indeed, on 2nd April 2020 after the announcement of the cancellation of examinations but before *Ofqual* specified details of calculated grades, the Runnymede Trust and several other race equality organisations wrote to the Secretary of State for Education to urge him to "ensure a fair, transparent and robust system which will more accurately reflect the ability and attainment of students from different backgrounds".(21) Subsequently, on 30th April, the Equality and Human Rights Commission said that,

> *"Using predicted grades in place of this year's summer assessments could deepen the existing inequality in education and put the future of disadvantaged young people at risk if not correctly implemented"* (22)

Our finding that students from private/selective schools were using more educational measures - especially online teaching in real time, which requires significant teacher input and which Andrew et al (15) argue is higher quality that other types of resource - reflects findings from those authors' research with parents of secondary school children (15) and teachers (16); however in our sample students' use of educational resources and time spent studying did not vary by socioeconomic background, including parental higher education, socioeconomic status, or area deprivation. This may be a feature of this particularly high-achieving sample of medical applicants.

Strengths and limitations

This study is, to our knowledge, the first systematic exploration of medical applicant views on and experiences of the most significant changes to UK education in living memory. It is also the first

For peer review only - http://bmjopen.bmj.com/site/about/guidelines.xhtml

study we are aware of that looked at university applicant views on calculated grades and the impact on university admissions. The large sample size gathered from around the UK, and the richness of the data allowed us to examine important differences in the experiences and views of different socio-demographic groups, after controlling statistically for educational attainment.

The speed at which we were required to develop the questionnaire and the unprecedented nature of the topic under investigation meant we were unable to use validated measures for most questions, nor have we been able to validate the measures ourselves, although we were able to pilot them with current applicants. Our data provide a snapshot of applicant views and experiences in April 2020, and it is possible that participants' views and experiences changed after data collection. The fact that participants are part of a longitudinal study however means we will have the chance to follow up participants in 2021 and beyond to discover how the pandemic affected their education. It is uncertain how representative our sample is of all medical applicants. Data on applications, offers, acceptances and academic achievement from the current UCAS cycle are not released until early 2021, but it is very likely that offer-holders were over-represented in our sample. Data from the 2019 UCAT testing cycle also show that our sample scored higher than the mean [https://www.ucat.ac.uk/media/1329/2019-test-statistics-oct-2019.pdf]; however not all UCAT test-takers apply to medicine. Demographic data on 2020 medical applicants released by UCAS in November 2019 showed that our restricted sample was similar to all English applicants aged 17 to 19 in terms of ethnicity and deprivation but had more women [https://www.ucas.com/data-and-analysis/undergraduate-statistics-and-reports/ucas-undergraduate-releases/applicant-releases-2020/2020-cycle-applicant-figures-15-october-deadline].

Medical applicants are not representative of all university applicants in either academic or socio-demographic terms; however the similarity of some of our findings to that of other research, for example that private school pupils are receiving significantly more education than non-selective state school pupils, suggests that the views and experiences of our sample may not be completely different from those of university applicants more generally; however generalisations from our findings to all applicants should only be done with caution.

Implications for policy and practice

The impact of calculated grades on medical admissions was, at the time of writing, uncertain. Our questionnaire closed on 22nd April and on 5th May 2020 the Medical Schools Council announced that medical schools would honour all offers met (something not clear at the time of our questionnaire), while acknowledging that there were still a number of issues that needed resolving.

How calculated grades are likely to work in practice has also been explored by a parallel analysis by our team using UKMED data over the last ten years, comparing predicted A-level grades (which are likely to be similar to calculated grades) with actual, attained A-level grades.(23) Predicted grades were systematically higher in medical school applicants than eventual achieved grades. In addition predicted grades only predicted outcomes about two-thirds as well as achieved A-level grades, both in terms of outcomes five or six years later at the end of medical school, and seven or eight years later in postgraduate examinations. The under-prediction by predicted grades was mitigated in part, although not entirely, by combining predicted grades with UCAT/BMAT scores, which supports the views of some applicants that other measures might be used for selection amongst applicants not meeting the terms of conditional offers.

The likely impacts on medical schools of using calculated grades were at the time of writing uncertain, but our estimates suggested there could in effect be a lowering of entry grade requirements, with possible subsequent increases in medical school drop-out rates, and a somewhat

For peer review only - http://bmjopen.bmj.com/site/about/guidelines.xhtml

academically weaker cohort with poorer performance in medical school and postgraduate examinations.(5, 24) That is potentially important since very poor postgraduate examination performance itself strongly predicts being sanctioned by the medical regulator.(25)

In the awarding of calculated grades, we predicted that the raw ‘centre assessment grades’ and rankings produced by teachers for *Ofqual* were likely to be similar to predicted grades in being more generous than achieved A-level grades would have been, although the standardisation to be used by examination boards and *Ofqual* are likely to minimise that effect, so that distributions of calculated grades within subjects and centres become similar to actual A-level grades in previous years. As it transpired the centre assessment grades ended up being used without adjustment, and these were significantly higher than previous years’ A-level grades, with the Education Datalab stating “At grades A*-A, there was an increase from 25.2% to 38.1%” (see https://ffteducationdatalab.org.uk/2020/08/gcse-and-a-level-results-2020-how-grades-have-changed-in-every-subject/).

As a result of the awarding of calculated grades an excess of candidates met their conditional offers[5]. Giving their views on what should happen in this regard, applicants in our study suggested that that in light of the shortage of doctors,(26) medical schools might argue for increased places and funding. In the event the Government did indeed lift the cap on medical school places to accommodate the increase in students (see https://www.gov.uk/government/news/action-agreed-to-support-students-into-preferred-universities). The impact of large increases in number on teaching and on predicting through to numbers of places for clinical teaching, foundation training and so on is still uncertain. It is worth considering that cohort sizes at many medical schools are already very large, that students tend to be less satisfied at larger schools,(27) and that accommodating extra students into face-to-face teaching that is COVID-secure is likely to be extremely challenging. On the other hand, there is a clear need for more doctors and it is likely that the change to admissions will result in a more socially and demographically diverse cohort.

In this questionnaire many applicants felt it could be fair to using other information such as interview score, UCAT score, or GCSE score to accept or reject offer-holders, and this could include in selecting from amongst ‘near-misses’. Overall respondents to our questionnaire demonstrate a lack of confidence in the process of calculated grades. Given the concerns of the Equality and Humans Rights Commission, and the clear concerns also expressed in our study by some disadvantaged groups, there is a clear need to ensure that entrants as far as possible continue to reflect the breadth of those applying to study medicine.

The cancellation of public examinations and the use of calculated grades are not the only problems facing the 2020 application cohort. They are also at risk, particularly those from non-selective state schools, of coming to medical school having had less education over the previous few months,(14) meaning medical schools may need to provide additional teaching and resources to help students catch up. This is likely to be especially challenging for medical schools given the huge constraints on university budgets arising from drops in student numbers(28) and given that many are likely to be unable to open for face-to-face teaching at the start of the academic year, which in itself has unknown consequences. The finding that Black Asian and Minority Ethnic groups were more likely to think teacher-estimated calculated grades could be unfair is concerning, and greater efforts need to

[5] In the UK system, university offers are made before students take their exams. Universities typically give offers that are conditional upon students achieving particular grades. Students meet their offer(s) and can be admitted if they achieve or exceed the grades specified.

For peer review only - http://bmjopen.bmj.com/site/about/guidelines.xhtml

be made to ensure education is fair and perceived as fair by students and all stakeholders. Transparent and independent analysis of the impact of exam cancellations on different sociodemographic groups, once data become available, will also be important.

The 2020 cohort of entrants is likely to face more uncertainty than any cohort of medical student entrants in the past half-century, and our survey makes very visible the many concerns of those applicants.

Conclusions

The global tragedy of the coronavirus pandemic, in addition to its extensive mortality and morbidity, has resulted in huge and sudden disruptions to established ways of life including education and training at all levels. Medical education and training is no exception. The coronavirus pandemic will have significant and long term impacts on the selection, education and performance of our future medical workforce. Understanding how medical education will be affected is therefore important, and in particular how applicants to become the newest entrants to medical careers are being affected. Now more than ever we need medical education, and medical education research, to be prioritised and funded so we can ensure our future doctors are able to be resilient, successful and happy healthcare professionals providing excellent patient care. The present study provides a wide range of insights into the feelings of the 2020 cohort of applicants, only a small proportion of which we have adequately been able to report here, but which demonstrate the concerns many have about examination cancelations in 2020 and looking forward to 2021

Acknowledgements

Firstly we are immensely grateful to the several thousand medical school applicants who took the time to respond to survey with a very tight time window, and we particularly thank those who commented that they were pleased that the survey they gave them an opportunity to express their thoughts, feelings and anxieties. We could not include everything that was said, but all comments have been read by the team, and we hope that the current paper summarises some of those many and varied views. We are also grateful to Paul Garrud, Clare Owen, Konstantinos Lulo, and Ewan McNicol for their comments on earlier versions of the questionnaire.

Contributors

KW, DH and CM jointly developed the idea for the study, and developed the questionnaire together. DH was responsible for putting the questionnaire online, and for identifying applicants to whom it should be sent, as well as sending text and email reminders. DH and KW cleaned the data, and KW, DH and CM were all involved in data analysis. The report was written jointly by all three authors, and all authors have read and reviewed the final draft.

Disclaimers

KW and DH state that this publication presents independent research funded by the National Institute for Health Research (NIHR). The views expressed are those of the authors and not necessarily those of the NHS, the NIHR or the Department of Health and Social Care.

Ethical approval

The study was approved by the UCL Research Ethics Committee Chair on 8th April 2020 as an amendment to the ongoing UKMACS longitudinal questionnaire study (reference: 0511/014).

For peer review only - http://bmjopen.bmj.com/site/about/guidelines.xhtml

Competing interests

All authors have completed the ICMJE uniform disclosure form at http://www.icmje.org/coi_disclosure.pdf : Dr Woolf and Dr Harrison report grants and non-financial support from National Institute for Health Research during the conduct of the study; and Dr Woolf reports personal fees from Transforming Student Access and Outcomes (TASO), outside the submitted work. All authors report no other relationships or activities that could appear to have influenced the submitted work.

Copyright

The Corresponding Author has the right to grant on behalf of all authors and does grant on behalf of all authors, a worldwide licence (http://www.bmj.com/sites/default/files/BMJ%20Author%20Licence%20March%202013.doc) to the Publishers and its licensees in perpetuity, in all forms, formats and media (whether known now or created in the future), to i) publish, reproduce, distribute, display and store the Contribution, ii) translate the Contribution into other languages, create adaptations, reprints, include within collections and create summaries, extracts and/or, abstracts of the Contribution and convert or allow conversion into any format including without limitation audio, iii) create any other derivative work(s) based in whole or part on the on the Contribution, iv) to exploit all subsidiary rights to exploit all subsidiary rights that currently exist or as may exist in the future in the Contribution, v) the inclusion of electronic links from the Contribution to third party material where-ever it may be located; and, vi) licence any third party to do any or all of the above. All research articles will be made available on an open access basis (with authors being asked to pay an open access fee—see http://www.bmj.com/about-bmj/resources-authors/forms-policies-and-checklists/copyright-open-access-and-permission-reuse). The terms of such open access shall be governed by a Creative Commons licence—details as to which Creative Commons licence will apply to the research article are set out in our worldwide licence referred to above.

Dissemination declaration

We have disseminated the pre-print of this article to those invited to respond.

Data sharing statement

The data will be linked into the UK Medical Education Database **www.ukmed.ac.uk** to which researchers can apply for access.

Funding

KW is a National Institute for Health Research (NIHR) Career Development Fellow (NIHR CDF-2017-10-008) and is principal investigator for the UKMACS and UKMEDP089 projects supported by the NIHR funding.

DH is funded by NIHR grant CDF-2017-10-008 to KW.

CM has received no specific funding for this project.

This publication presents independent research funded by the National Institute for Health Research (NIHR). The views expressed are those of the author(s) and not necessarily those of the NHS, the NIHR or the Department of Health and Social Care.

For peer review only - http://bmjopen.bmj.com/site/about/guidelines.xhtml

References

1. Efuribe C, Barre-Hemingway M, Vaghefi E, Ballonoff Suleiman A. Engagement and the Inclusion of Young People in Matters That Affect Their Lives. Journal of Adolescent Health. 2020.
2. McKie A. Scrapped exams may spark UK admissions 'scramble'. Times Higher Education. 2020 26/03/2020.
3. Summer 2020 grades for GCSE, AS and A level, Extended Project Qualification and Advanced Extension Award in maths: Information for Heads of Centre, Heads of Department and teachers on the submission of Centre assessment grades. Coventry; 2020 03/04/2020. Contract No.: Ofqual/20/6607/1
4. Machin SJ, McNally S, Ruiz-Valenzuela J. Entry Through the Narrow Door: The Costs of Just Failing High Stakes Exams. CESifo Working Paper Series 2018(7008).
5. McManus IC, Woolf K, Dacre J, Paice E, Dewberry C. The Academic Backbone: longitudinal continuities in educational achievement from secondary school and medical school to MRCP(UK) and the specialist register in UK medical students and doctors. BMC Medicine. 2013;11(1):242.
6. Cancellation of GCSE is unfair to some students. The Guardian. 2020 29/03/2020.
7. Lough C. GCSEs: Only 39% teachers think 2020 grades fair for all. Times Educational Supplement. 2020 13/05/2020.
8. (HEPI) HEPI. New poll finds almost one-third of applicants feel less confident they will get into their chosen university since the Coronavirus pandemic. Oxford: Higher Education Policy Institute; 2020 [Available from: https://www.hepi.ac.uk/2020/04/07/new-poll-finds-almost-one-third-of-applicants-feel-less-confident-they-will-get-into-their-chosen-university-since-the-coronavirus-pandemic/;.
9. Montacute R, Holt-White E. COVID-19 and Social Mobility Impact Brief #2: University Access & Student Finance. 2020.
10. New statement on medical school admissions in 2020: Medical Schools Council; 2020 [updated 05/05/2020. Available from: https://www.medschools.ac.uk/news/new-statement-on-medical-school-admissions-for-2020-entry.
11. Coronavirus (COVID-19): cancellation of GCSEs, AS and A levels in 2020: Department for Education; 2020 [updated 1st May 2020. Available from: https://www.gov.uk/government/publications/coronavirus-covid-19-cancellation-of-gcses-as-and-a-levels-in-2020/coronavirus-covid-19-cancellation-of-gcses-as-and-a-levels-in-2020.
12. Lavy V. Do Differences in Schools' Instruction Time Explain International Achievement Gaps? Evidence from Developed and Developing Countries. The Economic Journal. 2015;125(588):F397-F424.
13. Carlsson M, Dahl GB, Öckert B, Rooth D-O. The Effect of Schooling on Cognitive Skills. The Review of Economics and Statistics. 2015;97(3):533-47.
14. Burgess S, Sievertsen HH. Vox CEPR Policy Portal [Internet]2020. Available from: https://voxeu.org/article/impact-covid-19-education.
15. Andrew A, Cattan S, Costa Dias M, Farquharson C, Kraftman L, Krutikova S, et al. Learning during the lockdown: real-time data on children's experiences during home learning. Institute for Fiscal Studies; 2020.
16. Monitoring COVID-19 Readiness in Schools: Teacher Tapp; 2020 [Available from: https://teachertapp.co.uk/monitoring-covid-19-readiness-in-schools/A
17. Understanding Society: Waves 1-9, 2009-2018 and Harmonised BHPS: Waves 1-18, 1991-2009. [data collection]. 12th Edition. In: University of Essex IfSaER, NatCen Social Research, Kantar Public, editor.: UK Data Service; 2019.
18. McCrae RC, PT. Personal trait as a human universal. American Psychologist. 1995;52(5).
19. Revelle W. psych: Procedures for Psychological, Psychometric, and Personality Research. Northwestern University, Evanston, Illinois.; 2019.

For peer review only - http://bmjopen.bmj.com/site/about/guidelines.xhtml

20. Murphy R, Wyness G. Minority Report: the impact of predicted grades on university admissions of disadvantaged groups. Centre for Education Policy and Equalising Opportunities, UCL Institute of Education; 2020.
21. Lough C. Coronavirus: Williamson warned over grading 'injustice'. Times Educational Supplement 2020 02/04/2020.
22. Predicted grading during COVID-19 could limit young people's futures: Equality and Human Rights Commission; 2020 [Available from: https://www.equalityhumanrights.com/en/our-work/news/predicted-grading-during-covid-19-could-limit-young-peoples-futures.
23. McManus C, Woolf K, Harrison D, Tiffin PA, Paton LW, Yet Fong Cheung K, et al. Calculated grades, predicted grades, forecasted grades and actual A-level grades: Reliability, correlations and predictive validity in medical school applicants, undergraduates, and postgraduates in a time of Covid-19. forthcoming. 2020.
24. Curtis S, Smith D. A comparison of undergraduate outcomes for students from gateway courses and standard entry medicine courses. BMC Medical Education. 2020;20(1):4.
25. Wakeford R, Ludka K, Woolf K, McManus IC. Fitness to practise sanctions in UK doctors are predicted by poor performance at MRCGP and MRCP(UK) assessments: data linkage study. BMC Medicine. 2018;16(1):230.
26. Physicians RCo. Underfunded. Underdoctored. Overstretched. The NHS in 2016. London; 2016.
27. Devine OP, Harborne AC, McManus IC. Assessment at UK medical schools varies substantially in volume, type and intensity and correlates with postgraduate attainment. BMC Medical Education. 2015;15(1):146.
28. Achieving stability in the higher education sector following COVID-19. Universities UK; 2020 08/04/2020.

For peer review only - http://bmjopen.bmj.com/site/about/guidelines.xhtml

Figure captions

Figure 1: Participant flow diagram.

Figure 2: Perceptions of the fairness of methods medical schools could use to decide whether or not to accept applicants who currently hold an offer now that exams have been cancelled.

Figure 3: Acceptability of actions medical schools could take if they have more applicants meeting offers than they have places.

Figure 4: Views on how current applicants should be considered by medical schools if they reapply next year.

Figure 5: Aspects of calculated grades that respondents were generally more a) positive and b) negative about

Figure 6: Proportion of respondents undertaking various activities to prepare for medical school or university.

Figure 7: Amount of time respondents reported spending on various activities during the lockdown.

Figure 8: Scree plot for the factor analysis of 87 attitudinal variables.

For peer review only

For peer review only - http://bmjopen.bmj.com/site/about/guidelines.xhtml

Table 1 : Demographics for the full sample and the restricted sample (of those in Year 13, with at least three predicted A-level*s, no achieved* A-level*s, who had applied to study medicine). Rounding to prevent identifying individuals.*

	Full sample N (%)	Restricted sample N (%)
Female	1968 (68)	1097 (70)
Male	749 (26)	416 (27)
Other	20 (<1%)	<10 (<1)
Missing	140 (5)	Rounded to 40 (3)
White	670 (23)	516 (33)
Asian	301 (11)	228 (15)
Black	79 (3)	58 (4)
Mixed/Other	104 (4)	87 (6)
Missing	1723 (60)	673 (43)
1+ parents with degree	1831 (64)	1046 (67)
First in family	895 (33)	465 (30)
Missing	151 (5)	51 (3)
1+ parents in the highest socioeconomic group	1910 (66)	1097 (70)
No parents in the highest socioeconomic group	1742 (30)	439 (28)
Missing	116 (4)	26 (2)
No parent doctors	2408 (88)	1334 (85)
1+ parents who are doctors	344 (13)	192 (12)
Missing	125 (4)	36 (2)
Non-selective state school	785 (27)	590 (38)
Private or selective school	783 (27)	568 (36)
Missing	1309 (46)	404 (26)
IMD quintile 5 (most deprived – reverse scored)	310 (11)	169 (11)
IMD quintile 4 (reverse scored)	361 (13)	218 (14)
IMD quintile 3 (reverse scored)	410 (14)	236 (15)
IMD quintile 2 (reverse scored)	461 (16)	267 (17)
IMD quintile 1 (least deprived – reverse scored)	704 (25)	441 (28)
Missing	631 (22)	231 (15)
In Year 13/S6	2212 (77)	1562 (100)
One year post-Year 13	179 (6)	0 (0)
Have/studying for a degree	340 (12)	0 (0)
Mature without a degree/other	146 (5)	0 (0)
Missing	0 (0)	0 (0)
England	2003 (70)	1281 (82)
Scotland	170 (6)	<1 (<1)
Wales	78 (3)	50 (3)
Northern Ireland/ Forces/Islands	66 (2)	Rounded to 40 (2)
Other/missing	560 (20)	192 (12)
Total	2877 (100)	1562 (100)

For peer review only - http://bmjopen.bmj.com/site/about/guidelines.xhtml

Table 2: Predictors of agreement with statements relating to calculated grades. Predictors are ordered left to right by strength of relationship to the statement. Only statements that showed significant differences by social/demographic group after controlling for prior attainment and the number of offers are shown.

	Independent predictors of agreement with statement			
I would prefer not to have calculated grades at all and instead take A levels (or equivalents) in September.	Lower predicted A level points	BAME	Fewer conditional offers	Female
Overall, I would prefer to withdraw entirely from calculated grades and sit exams properly next summer.	Lower predicted A-level points	BAME	Fewer conditional offers	Female
The process described above is the best way to be fair to most students.	Higher predicted A-level points	White	Higher UCAT/BMAT scores	
I feel confident this process will result in an accurate assessment of my true abilities.	Higher predicted A-level points	White	Male	
Many students do better than their teachers expect; calculated grades cannot take that into account.	Lower predicted A-level points	BAME		
My teachers should take into account the disruption caused by coronavirus when judging grades.	Lower predicted A-level points	BAME		
Calculated grades should be based only on my performance, not on how previous students at my school performed.	Non-Selective State school	Higher deprivation		
I am confident in my teachers' abilities at grading and ranking students.	Higher predicted A-level points	White		
My teachers do not know enough about me to grade and rank me accurately.	Lower predicted A-level points	BAME	Fewer conditional offers	
In large schools/colleges, it is difficult to see how teachers can rank so many students.	BAME	Lower predicted A-level points		
Teachers judging grades should take into account the fact that many students do not do well in mocks but then work hard and do well in exams.	Lower predicted A-level points	Female	Fewer conditional offers	
Employers and universities in the future will treat grades from 2020 differently compared to exam grades taken from other years.	Female	Fewer conditional offers		

For peer review only - http://bmjopen.bmj.com/site/about/guidelines.xhtml

Table 3: School-provided educational resources used by respondents from non-selective state schools and private/selective schools

	N (%) used resource			
	Non selective state school	Private or selective school	Total	p value
Online resources	342 (63.3)	439 (80.0)	781 (71.7)	<.001
Paper resources	315 (58.3)	375 (69.6)	690 (63.9)	<.001
Online formative tests	187 (34.8)	260 (48.2)	447 (41.5)	<.001
Pastoral support	160 (29.7)	199 (37.2)	359 (33.4)	0.009
University application support	152 (28.5)	174 (32.3)	326 (30.4)	0.174
Online teaching in real time	66 (12.4)	248 (45.7)	314 (29.2)	<.001
Online summative tests	70 (13.2)	95 (17.7)	165 (15.4)	0.042
Other	12 (6.3)	25 (14.2)	37 (10.1)	0.011

.

For peer review only

For peer review only - http://bmjopen.bmj.com/site/about/guidelines.xhtml

***Table 4**: Respondents' main reasons for not using school educational resources during the shutdown by school type*

Resource not used	Reason not used	N (%) resource NOT used Non-selective state school	Private or selective school	Total
Online resources	Not available	96 (46.6)	48 (43.2)	144 (45.4)
	Don't need to	80 (38.8)	49 (44.1)	129 (40.7)
Paper resources	Not available	109 (50.5)	74 (46.8)	183 (48.9)
	Don't need to	88 (40.7)	69 (43.7)	157 (42.0)
Online formative test	Not available	206 (60.2)	129 (48.3)	335 (55.0)
	Don't need to	119 (34.8)	116 (43.3)	235 (38.6)
Pastoral support	Not available	161 (42.6)	94 (28.4)	255 (36.0)
	Don't need to	194 (51.3)	205 (61.9)	399 (56.3)
Uni application support	Not available	185 (49.9)	141 (40.5)	326 (45.3)
	Don't need to	155 (41.8)	182 (52.3)	337 (46.9)
Online teaching in real time	Not available	337 (71.7)	189 (63.0)	526 (68.3)
	Don't need to	109 (23.3)	99 (33.0)	208 (27.0)
Online summative test	Not available	289 (65.4)	223 (52.5)	512 (59.1)
	Don't need to	142 (32.1)	177 (41.6)	319 (36.8)
Other	Not available	66 (54.1)	42 (39.3)	108 (47.2)
	Don't need to	47 (38.5)	49 (45.8)	96 (41.9)

.

For peer review only

For peer review only

Table5: Predictors of factor scores. Set A includes Number of offers, GCSE points, Predicted A-level points, UCAT/BMAT score, Private/Selective school, Female, BAME, Degree-educated parent(s) and Deprived area. Set B includes Set A plus Highest socioeconomic group, doctor parent(s), and Big5 personality factors Agreeableness, Conscientiousness, Extraversion, Neuroticism and Openness. All predictors reported have p<.01, and are reported in descending order of significance (i.e. most significant at the top).

	**Set A Predictors in order of magnitude**	**Set B Predictors in order of magnitude**
Factor 1: Lack of confidence in calculated grades	Lower predicted A-levels<br>BAME<br>Fewer conditional offers<br>Female	Lower predicted A-levels<br>BAME<br>Fewer conditional offers<br>Higher Openness<br>Lower Conscientiousness<br>Female
Factor 2: Special treatment next year for rejected applicants	Lower predicted A-levels<br>Lower UCAT/BMAT	Lower predicted A-levels<br>Higher Openness<br>Lower UCAT/BMAT<br>Higher Neuroticism<br>Higher Extraversion
Factor 3: Other selection measures to be taken into account	Higher UCAT/BMAT<br>Lower predicted A-levels<br>Male	Higher UCAT/BMAT<br>Lower predicted A-levels<br>Higher Extraversion<br>Male<br>Lower Conscientiousness
Factor 4: Preparing for medical school	White<br>Female	Higher Conscientiousness<br>Lower Neuroticism<br>White<br>Female<br>Higher Agreeableness<br>Higher Openness
Factor 5: Importance of background and experience	Lower UCAT/BMAT<br>BAME<br>Female	Higher Openness<br>Lower UCAT/BMAT<br>Fewer conditional offers<br>BAME
Factor 6: Resources from school for home study	Selective school<br>Lower GCSE<br>Fewer conditional offers	Selective School<br>Lower GCSE<br>Lower Extraversion<br>Higher Openness

For peer review only - http://bmjopen.bmj.com/site/about/guidelines.xhtml

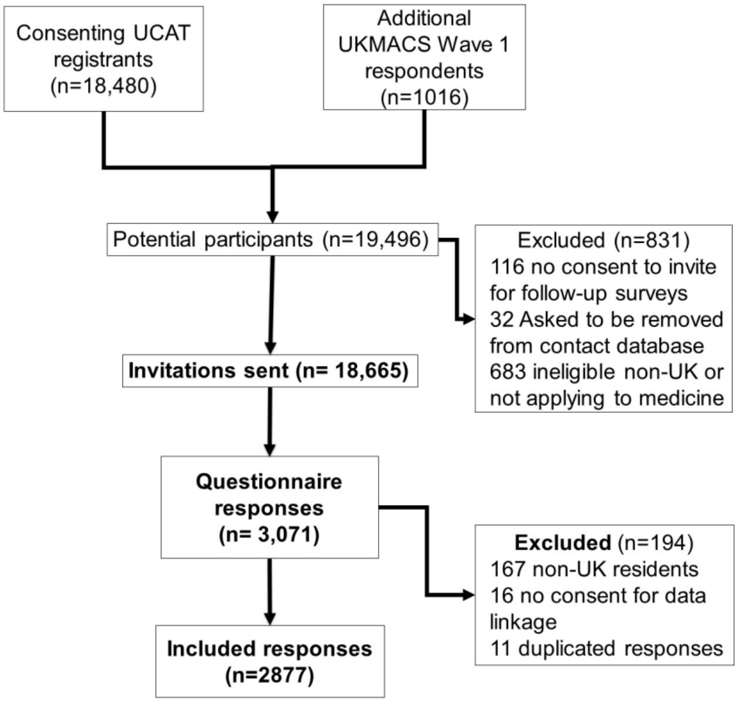

Figure 1: Participant flow diagram.

451x338mm (96 x 96 DPI)

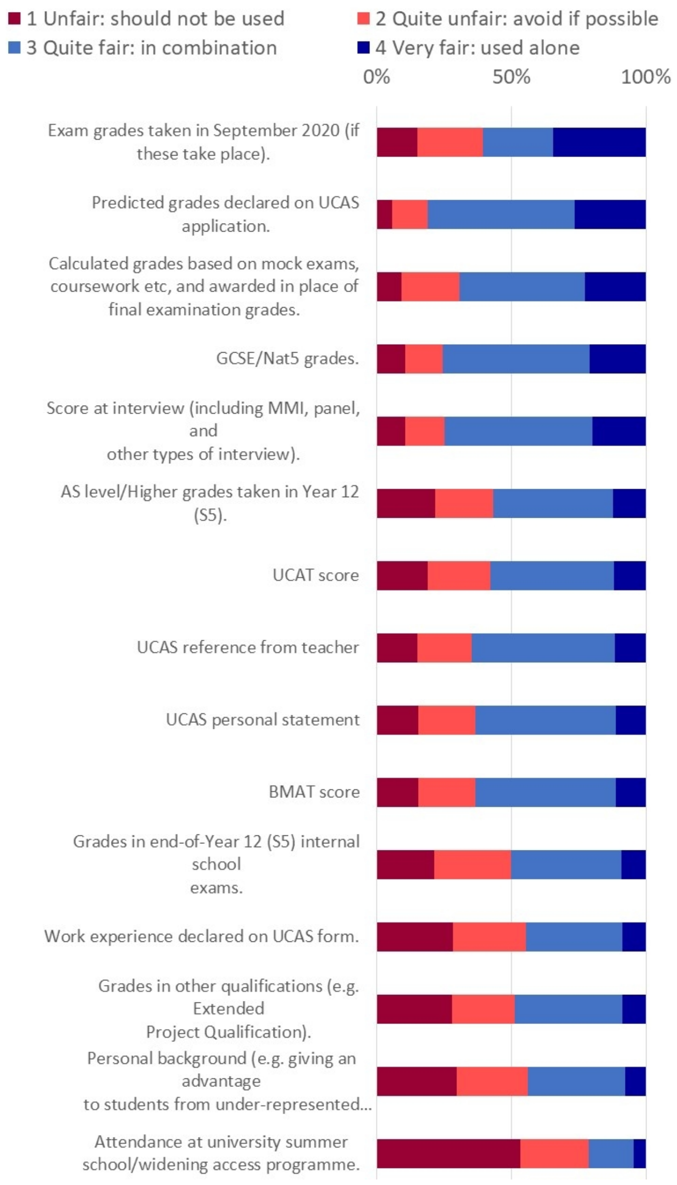

Figure 2: Perceptions of the fairness of methods medical schools could use to decide whether or not to accept applicants who currently hold an offer now that exams have been cancelled.

190x338mm (96 x 96 DPI)

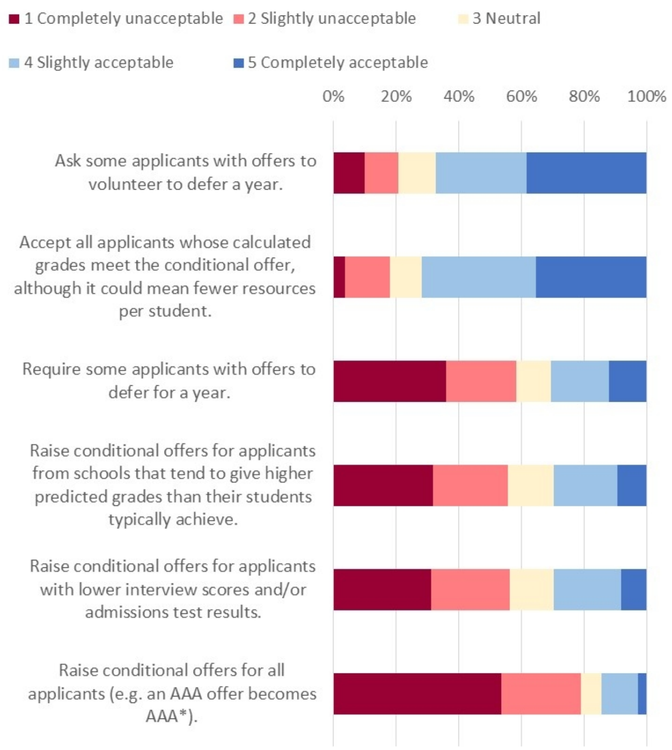

Figure 3: Acceptability of actions medical schools could take if they have more applicants meeting offers than they have places.

190x338mm (96 x 96 DPI)

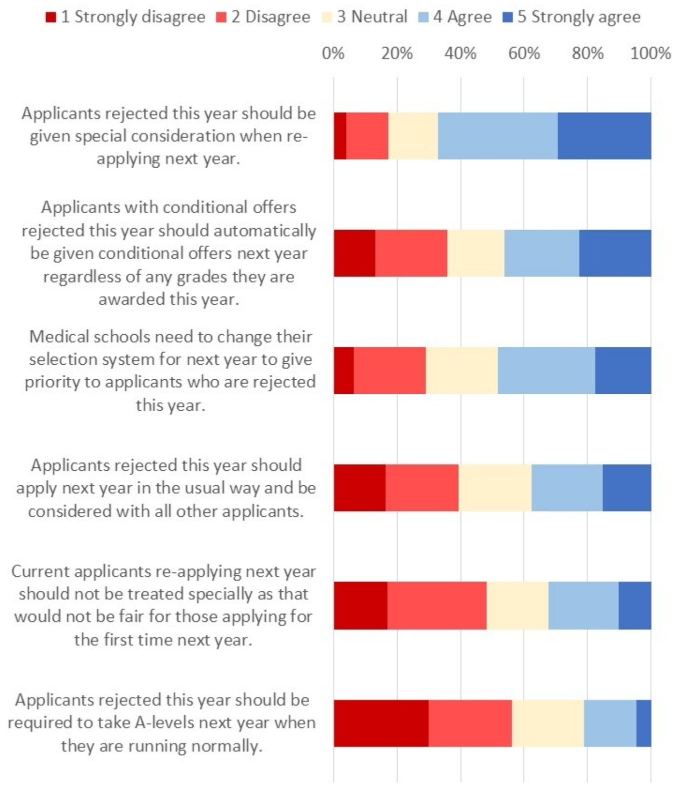

Figure 4: Views on how current applicants should be considered by medical schools if they reapply next year.

190x338mm (96 x 96 DPI)

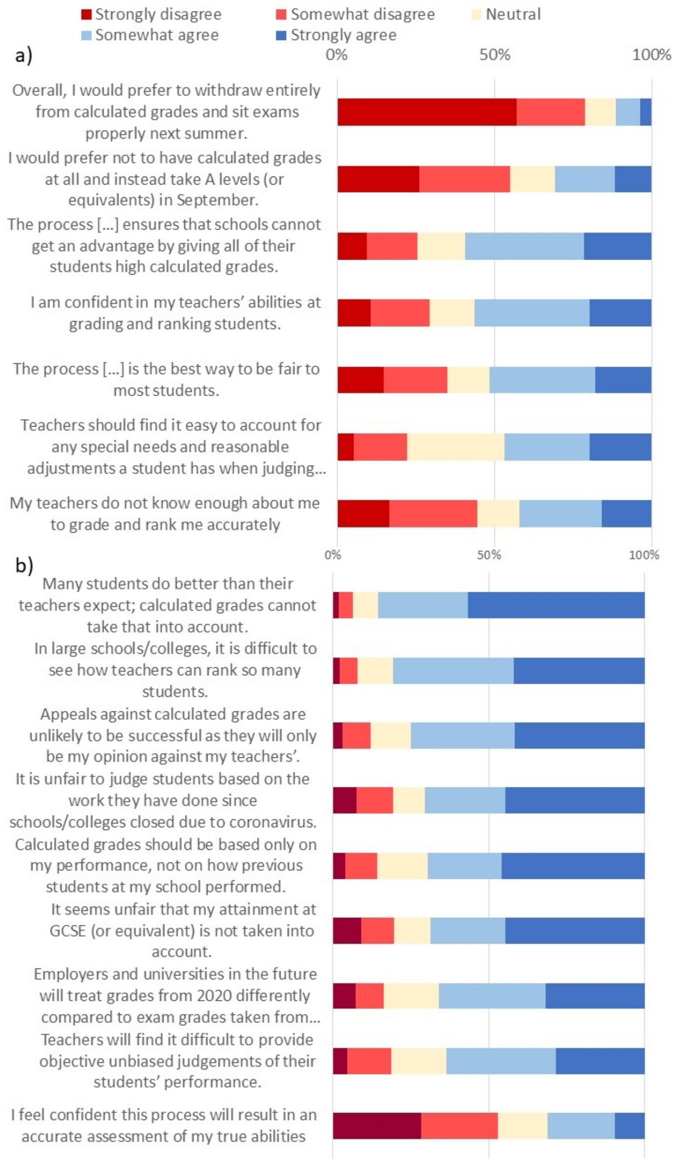

Figure 5: Aspects of calculated grades that respondents were generally more a) positive and b) negative about

190x338mm (96 x 96 DPI)

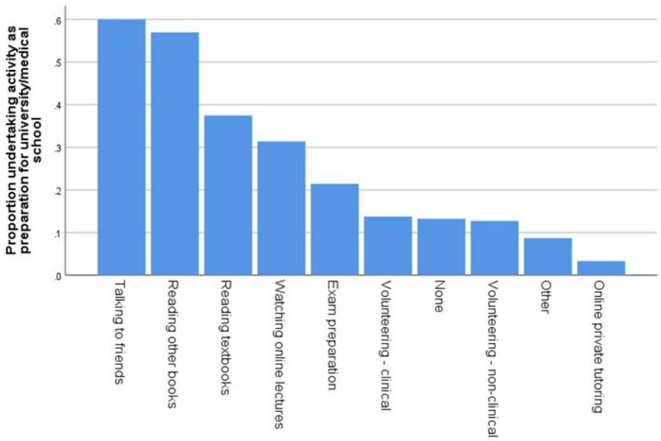

Figure 6: Proportion of respondents undertaking various activities to prepare for medical school or university.

190x338mm (96 x 96 DPI)

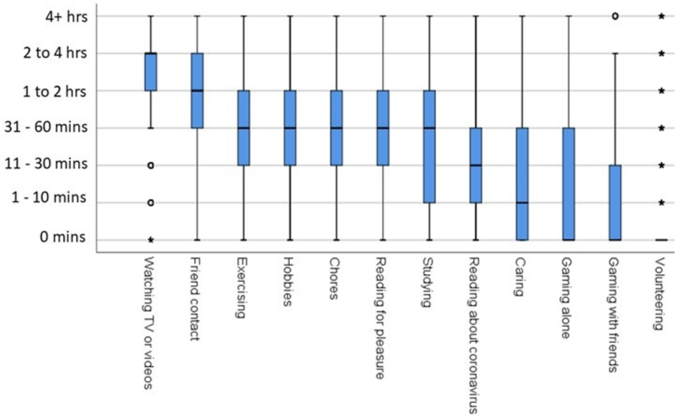

Figure 7: Amount of time respondents reported spending on various activities during the lockdown.

190x338mm (96 x 96 DPI)

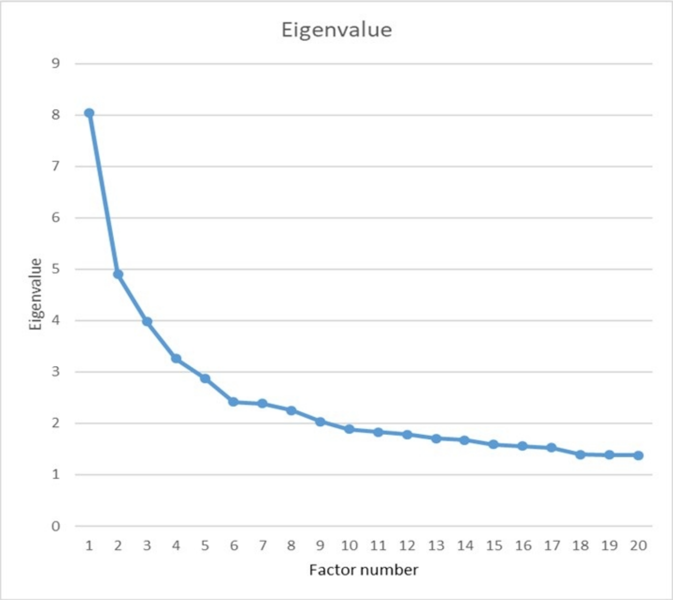

Figure 8: Scree plot for the factor analysis of 87 attitudinal variables.

190x338mm (96 x 96 DPI)

FUNDED BY NIHR | National Institute for Health Research

the NHS needs YOU

UK Medical Applicant Cohort Study

UKMACS-COVID19

UKMACS COVID-19 questionnaire

Due to the coronavirus/COVID-19 outbreak, UK school examinations have been cancelled and alternatives for selecting medical students are being considered.

The UK Medical Applicant Cohort Study (UKMACS) is an academic research study about applying to medical school. We want to find out how you, the applicants, are reacting to these changes and what you think medical schools should do to select applicants fairly.

This questionnaire needs to be answered by **20th April 2020**.
The turnaround is very tight as we want the questionnaire to assist medical schools in making decisions on selection.

We keep your information private and confidential.

No medical school, school or university will be able to identify you from the information you give us.

The information you give us will NOT be used to make any decisions about you personally that will affect your education or career.

You are being invited to complete this questionnaire because you agreed to be contacted by us when you registered to take UCAT in 2019 and/or because you have already responded to one or more of our questionnaires.

Please note that we are interested in responses from ALL applicants to medical school, whether or not you completed any of our previous questionnaires, whether or not you have been offered a place, and whether or not you were due to take A levels/Highers/equivalents this year.

Before you proceed to the questionnaire, please download the study Information Sheet and read it so you understand what the study is about and what taking part entails.

Contact study lead Dr Katherine Woolf and the UKMACS research team on medsch.choice@ucl.ac.uk if you have any questions.

The study is led by Dr Katherine Woolf at University College London (UCL), and funded by the National Institute for Health Research (NIHR) Career Development Fellowship (Grant Reference Number CDF-2017-10-008). The views expressed are those of the authors and not necessarily those of the NIHR or the Department of Health and Social Care.

For peer review only - http://bmjopen.bmj.com/site/about/guidelines.xhtml

Eligibility to take part in this study

This study is for people aged 16 or over, resident in the UK or Crown Dependencies, who applied or were considering applying to study medicine in the UK for entry in 2020.

1. Please select "Yes" to indicate you are aged 16 or older, resident in the UK (or Channel Islands/Isle of Man). If you select "No" you cannot take part in this study. ✱ *Required*

- (•) Yes
- ( ) No

For peer review only

Consent to take part in this study

2. Please select “I consent” to each statement to show you understand and agree to taking part in the study. If you select "I do NOT consent" you may not be able to take part in the study.

	I consent	I do NOT consent
I understand my personal information will be kept private by the research team on password-protected computers or encrypted files at UCL, and will not be passed on to any commercial organisations. [‘Personal information’ means: name, questionnaire answers, or any other information of yours we have such as contact details, UCAT or other test registration ID number.]	◉	○
I agree my questionnaire answers can be linked with other personal information about me and used only for academic research, as explained in the Information Sheet.	◉	○
I understand that taking part in this study is voluntary, and I can withdraw by contacting the research team by the 22nd April 2020.	◉	○
I understand the potential risks of taking part in the study and the support I can get if needed, including who to contact with questions or to make a complaint (see Information Sheet).	◉	○

For peer review only - http://bmjopen.bmj.com/site/about/guidelines.xhtml

How medical schools should select applicants in the absence of exam grades

Medical schools are currently discussing how to select applicants without examination grades. The following questions ask about a range of ways being discussed as possibilities. Please let us know how fair or unfair you think each option is. None of these options has yet been decided upon. Your responses may have an influence upon what medical schools decide to do.

Some of these questions are specific to applicants still at secondary school and others are more relevant to mature/graduate entrants. Please answer all questions as far as possible. Later on in the questionnaire we will ask about your educational background so we can factor that into our analyses.

3. Here is a list of different measures that medical schools could use to decide whether or not to accept **applicants who currently hold an offer**. For each method, please indicate how fair it is to use to accept or reject current offer holders now that exams have been cancelled. ["Calculated grades' are grades produced by examination boards based on estimated grades and rankings produced teachers using information on a student's progress. The method has been described by Ofqual for England and is likely to be broadly similar elsewhere].

More info

	Unfair: should not be used	Quite unfair: avoid if possible	Quite fair: could be used in combination with other measures	Very fair: could be used alone	Uncertain
Predicted grades declared on UCAS application.	○	○	○	○	○
Calculated grades based on mock exams, coursework etc, and awarded in place of final examination grades.	○	○	○	○	○
Exam grades taken in September 2020 (if these take place).	○	○	○	○	○
AS level/Higher grades taken in Year 12 (S5).	○	○	○	○	○
Grades in end-of-Year 12 (S5) internal school exams.	○	○	○	○	○
GCSE/Nat5 grades.	○	○	○	○	○
Grades in other qualifications (e.g. Extended Project Qualification).	○	○	○	○	○
For those in their final year at university, marks earlier in their course.	○	○	○	○	○
Score at interview (including MMI, panel, and other types of interview).	○	○	○	○	○
UCAT score.	○	○	○	○	○
BMAT score.	○	○	○	○	○
GAMSAT score (for Graduate Entry students).	○	○	○	○	○
UCAS personal statement.	○	○	○	○	○
UCAS reference from teacher.	○	○	○	○	○
Work experience declared on UCAS form.	○	○	○	○	○
Attendance at university summer school/widening access programme.	○	○	○	○	○
Personal background (e.g. giving an advantage to students from under-represented groups).	○	○	○	○	○

For peer review only - http://bmjopen.bmj.com/site/about/guidelines.xhtml

3.a. If you think there are other measures that could be used by medical schools to select applicants, please explain by typing in the box:

4. The cancellation of exams could potentially result in medical schools having many more students than they had planned for. In that situation, how acceptable or unacceptable are each of the following for medical schools to do:

	Completely unacceptable	Slightly unacceptable	Neutral	Slight acceptable	Completely acceptable
Accept all applicants whose calculated grades meet the conditional offer, although it could mean fewer resources per student.	○	○	○	○	○
Raise conditional offers for all applicants (e.g. an AAA offer becomes AAA*).	○	○	○	○	○
Raise conditional offers for applicants with lower interview scores and/or admissions test results.	○	○	○	○	○
Raise conditional offers for applicants from schools that tend to give higher predicted grades than their students typically achieve.	○	○	○	○	○
Ask some applicants with offers to volunteer to defer a year.	○	○	○	○	○
Require some applicants with offers to defer for a year.	○	○	○	○	○

4.a. If you have any other comments about how medical schools should cope with having many more students than places, please type it into the box:

For peer review only - http://bmjopen.bmj.com/site/about/guidelines.xhtml

Looking to academic year 2020/2021

5. The coronavirus outbreak could have a knock-on effect for medical school selection in 2021. Please indicate how much you agree or disagree with the following statements about what medical schools should do in selection next year:

Current applicants re-applying next year should not be treated specially as that would not be fair for those applying for the first time next year.	Please select
Medical schools need to change their selection system for next year to give priority to applicants who are rejected this year.	Please select
Applicants with conditional offers rejected this year should automatically be given conditional offers next year regardless of any grades they are awarded this year.	Please select
Applicants rejected this year should be given special consideration when re-applying next year.	Please select
Applicants rejected this year should be required to take A-levels next year when they are running normally.	Please select
Applicants rejected this year should apply next year in the usual way and be considered with all other applicants.	Please select

6. It is possible that universities will not start the next academic year at the usual time in October 2020. In that case, which of the following should universities do?

- Start the academic year on time using distance learning for as long as is necessary.
- Defer the start of the academic year only when face-to-face teaching is possible.

For peer review only - http://bmjopen.bmj.com/site/about/guidelines.xhtml

Calculated Grades

Now that examinations have been cancelled, students will be awarded ‘calculated grades’. We are interested in your opinion on this. Before giving us your opinion, please read the information below about how calculated grades will be awarded.

For each examination a student takes:

- Teachers will judge the grade a student would have got had they had taken the examination (for example A*, A, B, etc); AND where that student ranks compared to other students also getting that grade. For example, a student judged to get a B in Chemistry will be compared to all others also judged to get a B in Chemistry, with all students being ranked in order (1st, 2nd, 3rd etc). Teachers will do this after 29th May 2020.
    - Judgements will NOT be based on predicted grades submitted to UCAS. Instead they will be based on evidence such as classwork, homework, mock examinations, coursework etc.
    - Judgements are not told to students and are passed confidentially to exam boards and Ofqual (the English exam regulator).
- A final grade is then awarded by exam boards and Ofqual, based on the teacher judgement and other information about the school/college a student attends, such as how students at that school performed in previous years.

There will be an appeals process and it is possible students will be able to sit the examinations when schools open again, although that is still uncertain.

Full details of the process here https://www.gov.uk/government/publications/gcses-as-and-a-level-awarding-summer-2020. Please note the process may differ outside of England. However if you are outside of England please answer the questions as if the same arrangements were to apply to the exams you are taking.

7. Please rate how much you *agree* or *disagree* with each of the following statements about using calculated grades:

More info

	Strongly disagree	Somewhat disagree	Neutral	Somewhat agree	Strongly agree
I would prefer not to have calculated grades at all and instead take A levels (or equivalents) in September.	☐	☐	☐	☐	☐
Overall, I would prefer to withdraw entirely from calculated grades and sit exams properly next summer.	☐	☐	☐	☐	☐
The process described above is the best way to be fair to most students.	☐	☐	☐	☐	☐
The process described above ensures that schools cannot get an advantage by giving all of their students high calculated grades.	☐	☐	☐	☐	☐
I feel confident this process will result in an accurate assessment of my true abilities.	☐	☐	☐	☐	☐
It is unfair to judge students based on the work they have done since schools/colleges closed due to coronavirus.	☐	☐	☐	☐	☐
It seems unfair that my attainment at GCSE (or equivalent) is not taken into account.	☐	☐	☐	☐	☐
Many students do better than their teachers expect; calculated grades cannot take that into account.	☐	☐	☐	☐	☐
My teachers should take into account the disruption caused by coronavirus when judging grades.	☐	☐	☐	☐	☐
Calculated grades should be based only on my performance, not on how previous students at my school performed.	☐	☐	☐	☐	☐

For peer review only - http://bmjopen.bmj.com/site/about/guidelines.xhtml

8. Please rate how much you *agree* or *disagree* with each of the following statements about calculated grades:

	Strongly disagree	Somewhat disagree	Neutral	Somewhat agree	Strongly agree
I am confident in my teachers' abilities at grading and ranking students.	☐	☐	☐	☐	☐
My teachers do not know enough about me to grade and rank me accurately.	☐	☐	☐	☐	☐
In large schools/colleges, it is difficult to see how teachers can rank so many students.	☐	☐	☐	☐	☐
Teachers judging grades should take into account the fact that many students do not do well in mocks but then work hard and do well in exams.	☐	☐	☐	☐	☐
Teachers should find it easy to account for any special needs and reasonable adjustments a student has when judging grades.	☐	☐	☐	☐	☐
Teachers will find it difficult to provide objective unbiased judgements of their students' performance.	☐	☐	☐	☐	☐
Appeals against calculated grades are unlikely to be successful as they will only be my opinion against my teachers'.	☐	☐	☐	☐	☐
Employers and universities in the future will treat grades from 2020 differently compared to exam grades taken from other years.	☐	☐	☐	☐	☐

For peer review only - http://bmjopen.bmj.com/site/about/guidelines.xhtml

Your application to university

9. **Did you apply to university this academic year (2019/2020)?** ✱ *Required*

- ○ Yes applied to study medicine
- ○ Yes applied but NOT to any medical courses
- ○ No

For peer review only

For peer review only - http://bmjopen.bmj.com/site/about/guidelines.xhtml

Your university choices

10. **Please indicate which universities and courses you have applied to, the response you have had from each, and type in any offers you have received.** For the course, please type in either the UCAS course code (e.g. A100, B900) or the course name (e.g. Standard Entry Medicine, Biomedical Science).

	University name	Course	What response have you had from this university?	Please type in your conditional offer (if applicable):
1st Choice			Please select	
2nd Choice			Please select	
3rd Choice			Please select	
4th Choice			Please select	
5th Choice			Please select	

peer review only

For peer review only - http://bmjopen.bmj.com/site/about/guidelines.xhtml

Communication from universities about changes to selection

For each of your choices: has this university been in touch with you about any changes they are making to their selection processes due to coronavirus/COVID-19?

11. Your Choice 1: has this university told you about any changes they are making to their selection processes due to coronavirus/COVID-19?

11.a. If you selected Other, please specify:

12. Your Choice 2: has this university told you about any changes they are making to their selection processes due to coronavirus/COVID-19?

12.a. If you selected Other, please specify:

13. Your Choice 3: has this university told you about any changes they are making to their selection processes due to coronavirus/COVID-19?

13.a. If you selected Other, please specify:

14. Your Choice 4: has this university told you about any changes they are making to their selection processes due to coronavirus/COVID-19?

For peer review only - http://bmjopen.bmj.com/site/about/guidelines.xhtml

14.a. If you selected Other, please specify:

15. Your Choice 5: has this university told you about any changes they are making to their selection processes due to coronavirus/COVID-19?

15.a. If you selected Other, please specify:

For peer review only - http://bmjopen.bmj.com/site/about/guidelines.xhtml

Your education and qualifications

To put into context the answers to the earlier questions it would be helpful if we knew more about your educational qualifications, both those you were due to take this year, and those you have taken in previous years.

16. Which one of the following best describes you? ✱ *Required*

- ○ In Year 13 (S6) of school/college
- ○ Was in Year 13 (S6) of school/college last academic year (2018/2019)
- ○ In the final year of a degree programme
- ○ Have a degree already
- ○ Mature applicant without degree
- ○ Other

16.a. If you selected Other, please specify:

For peer review only - http://bmjopen.bmj.com/site/about/guidelines.xhtml

Your university

17. Please type in the name of your the university and course and subject you are currently studying for or have already gained (e.g. University of Bath, BSc Biomedical Science 2.1). If you have more than one degree, please type each in.

18. If you are currently in the final year of your degree, has your university told you how they will determine your final degree mark (please choose one)? If you are not in your final year please select 'Not applicable'.

- No
- Yes - please give details below
- Uncertain
- Other - please give details below
- Not applicable

19. If you selected Yes or Other to the question above, please give details in the box:

For peer review only - http://bmjopen.bmj.com/site/about/guidelines.xhtml

Your school/college

Please type in the full name and first part of the postcode of the school or college you are currently attending. If you are not at school or college, please type in the details of your last school/college.

20. Name of school/college (e.g. St Mary's C of E School, Kilburn):

20.a. First part of postcode of school/college (e.g. NW6):

For peer review only

Your qualifications

21. **Please tell us about the A levels, Scottish Highers or equivalent qualifications you are currently taking and/or have already taken (most A level students will have 3 subjects). For each qualification, please indicate the qualification type, subject, grade (predicted or achieved), and the year in which you are expected to achieve/achieved the qualification.**

	Qualification type	Subject	Predicted grade	Achieved grade	Year
1	Please select				
2	Please select				
3	Please select				
4	Please select				
5	Please select				
6	Please select				
7	Please select				
8	Please select				
9	Please select				
10	Please select				

For peer review only - http://bmjopen.bmj.com/site/about/guidelines.xhtml

Admission tests: UCAT

In this section please give us some basic information about the various admissions tests such as UCAT, BMAT and GAMSAT that you may have taken.

If you are not certain of precise scores then put in approximate values.

22. Did you take the UCAT (University Clinical Aptitude Test) in 2019?

- ○ Yes
- ○ No

For peer review only

UCAT score

23. **What was your total UCAT score and your score on each UCAT subsection (please type)?** *If unsure, type "unsure". If you have taken UCAT more than once, please give your most recent scores. Please estimate if you cannot remember your exact scores.*

	Score
Total UCAT score	
Verbal Reasoning	
Decision Making	
Quantitative Reasoning	
Abstract Reasoning	
Situational Judgement	

For peer review only

Admissions tests: BMAT

24. Did you take the BMAT (BioMedical Admissions Test) in 2019?

- Yes
- No

For peer review only

BMAT score

25. **What was your score on each BMAT subsection (please type)?** If you have taken BMAT more than once, please give your most recent scores. If you did not take the test, please type "na". *If unsure or unknown please type "unsure". Please estimate if you cannot remember your exact scores.*

	Score
Section 1	
Section 2	
Section 3	

For peer review only

Admissions test: GAMSAT

26. Did you take the GAMSAT (Graduate Medical School Admissions Test) in 2018 or 2019?

- Yes
- No

For peer review only

GAMSAT score

27. **What was your overall GAMSAT score and your score on each GAMSAT section (please type)?** If you have taken GAMSAT more than once, please give your most recent scores. *If you are unsure what your scores were, type "unsure". Please estimate if you cannot remember your exact scores.*

	Score
Overall score	
Section I	
Section II	
Section III	

For peer review only

For peer review only - http://bmjopen.bmj.com/site/about/guidelines.xhtml

Impact of coronavirus/COVID-19 on your day to day life

It would be helpful to understand how applicants' lives and education are being affected by coronavirus/COVID-19 so medical schools can take this into account in selection if necessary. Please be assured all your answers are kept confidential.

If you want support dealing with this difficult time please contact the organisations listed here: https://www.nhs.uk/conditions/stress-anxiety-depression/mental-health-helplines/ or Childline on 0800 1111 https://www.childline.org.uk/.

28. On a typical day in the past five days, about how much time have you spent on the following?

	Time per day						
	Zero mins	1- 10 mins	11-30 mins	31-60 mins	1 to 2 hours	2 to 4 hours	4+ hours
Reading about coronavirus/COVID-19	○	○	○	○	○	○	○
Phoning/texting/video calling your friends	○	○	○	○	○	○	○
Watching TV or videos	○	○	○	○	○	○	○
Gaming online with friends	○	○	○	○	○	○	○
Gaming alone	○	○	○	○	○	○	○
Reading for pleasure	○	○	○	○	○	○	○
Exercising	○	○	○	○	○	○	○
Other hobbies	○	○	○	○	○	○	○
Studying	○	○	○	○	○	○	○
Household chores/shopping for necessities	○	○	○	○	○	○	○
Caring for members of your household	○	○	○	○	○	○	○
Volunteering	○	○	○	○	○	○	○

Your education during the coronavirus outbreak

29. If you are currently in education which of the following is your school/college/uni providing? For each activity, please indicate whether you have used it or not. If you have not used it, please tell us why not. If you are not currently in education, please leave these questions blank.

		If you have not used this, why not?
Online teaching in real time using video (e.g. Zoom, Skype, Teams).	Please select	Please select
Online resources for home learning (e.g. links to activities, apps).	Please select	Please select
Paper resources for home learning (e.g. workbooks).	Please select	Please select
Online exams/assessments that count toward your calculated grades/degree award.	Please select	Please select
Online assessments that don't count towards your calculated grades/degree award (e.g. quizzes).	Please select	Please select
Pastoral support (e.g. asking about mental health, providing mental health resources, checking on home circumstances and suitability for studying).	Please select	Please select

Support with your university application (e.g. choosing between firm and insurance choices).	Please select	Please select
Other.	Please select	Please select

29.a. If you selected Other reasons then please specify here:

30. Will your school/college/uni formally assess you on any work you do or have done since schools/unis closed? For example, to inform your calculated grades or to determine your degree class, if applicable? If you are not currently in education please select 'Not applicable'.

- Yes
- No
- Uncertain
- Not applicable

For peer review only - http://bmjopen.bmj.com/site/about/guidelines.xhtml

Preparing for medical school or university

31. Other than schoolwork or studying for your degree (if relevant) are you doing any preparation for medical school or university? Please select all that apply:

- ☐ Watching online lectures
- ☐ Online private tutoring
- ☐ Reading textbooks
- ☐ Reading other books
- ☐ Volunteering in a clinical environment
- ☐ Volunteering in a non-clinical environment
- ☐ Exam preparation (e.g. timed essays, past papers)
- ☐ Talking to friends
- ☐ Other
- ☐ Not doing any preparation

31.a. If you selected Other, please specify:

32. If you are not doing any preparation, please tell us why not (please select all that apply):

- ☐ I'm not going to university this year
- ☐ Don't have enough time
- ☐ Can't focus because too worried
- ☐ Don't have any resources
- ☐ I'm caring for others
- ☐ I'm unwell
- ☐ It's not necessary
- ☐ Other

For peer review only - http://bmjopen.bmj.com/site/about/guidelines.xhtml

Your general approach to life

The following questions are about how you behave and how you see yourself as a person. Please select the response which best describes how you see yourself, where 1 means does not apply to me at all and 7 means applies to me perfectly. Don't think too hard before answering, just give the first answer that comes to mind.

33. *I see myself as someone who...*

	1=Does not apply to me at all. 7=Applies to me perfectly.						
	1	2	3	4	5	6	7
is sometimes rude to others	○	○	○	○	○	○	○
does a thorough job	○	○	○	○	○	○	○
is talkative	○	○	○	○	○	○	○
worries a lot	○	○	○	○	○	○	○
is original, comes up with new ideas	○	○	○	○	○	○	○
has a forgiving nature	○	○	○	○	○	○	○
tends to be lazy	○	○	○	○	○	○	○
is outgoing, sociable	○	○	○	○	○	○	○
gets nervous easily	○	○	○	○	○	○	○
values artistic, aesthetic experiences	○	○	○	○	○	○	○
is considerate and kind to almost everyone	○	○	○	○	○	○	○
does things efficiently	○	○	○	○	○	○	○
is reserved	○	○	○	○	○	○	○
is relaxed, handles stress well	○	○	○	○	○	○	○
has an active imagination	○	○	○	○	○	○	○

For peer review only - http://bmjopen.bmj.com/site/about/guidelines.xhtml

Your parent/carers' occupations and education

This section has some questions about your parents' or carers' occupation(s) and your household. Your answers will help us understand how family background may affect entry to medical school.

Please select the option(s) that best describes the occupation(s) of your parent(s)/carer(s).

If your parent(s)/carer(s) are not currently working please choose what they were employed as.

If you are no longer living with your parent(s) or carer(s), or are unsure what their occupation is, please still answer these questions as best you can.

34. Occupation of Parent/Carer 1:

34.a. If you selected Other, please specify:

35. Occupation of Parent/Carer 2:

35.a. If you selected Other, please specify:

36. Does one or more of your parent(s)/carer(s) have a university degree?

37. Is one or more of your parent(s)/carer(s) a medical doctor?

Your identification details

Please complete this section to enable the research team to use your survey responses as described in the Information Sheet. Remember, your information is kept confidential and stored securely.

Please type your FULL NAME [first name(s) and surname] in the boxes below.

38. First name:

39. Surname:

40. Please type your **date of birth** using the format DD (day) MM (month) YYYY (year).

(dd/mm/yyyy)

41. Which of the following best describes the gender you identify as?

41.a. If you selected Other, please specify:

For peer review only - http://bmjopen.bmj.com/site/about/guidelines.xhtml

Additional comments

42. Please use this space for any additional comments you wish to make about the questionnaire or selection of medical students:

43. These are unprecedented times. Please tell us about your **hopes for now and the future**, and tell us what **inspires** you.

Thank you for completing this questionnaire.

If you wish to go back and review your answers then please do so now.

When you click on the finish button below, your responses will be submitted to the UKMACS research team.

For peer review only - http://bmjopen.bmj.com/site/about/guidelines.xhtml

Thank You

Thank you for completing this questionnaire for UKMACS.

You can share this questionnaire with other medical applicants: **https://ucl.onlinesurveys.ac.uk/ukmacs-covid19-questionnaire**.

There is also more information on our website:https://ukmacs.wordpress.com/

If you have any questions about the study or this questionnaire, please contact the UKMACS Research Team medsch.choice@ucl.ac.uk

---

Key for selection options

5.1.a -
- Strongly disagree
- Disagree
- Neutral
- Agree
- Strongly agree

5.2.a -
- Strongly disagree
- Disagree
- Neutral
- Agree
- Strongly agree

5.3.a -
- Strongly disagree
- Disagree
- Neutral
- Agree
- Strongly agree

5.4.a -
- Strongly disagree
- Disagree
- Neutral
- Agree
- Strongly agree

5.5.a -
- Strongly disagree
- Disagree
- Neutral
- Agree
- Strongly agree

5.6.a -
- Strongly disagree
- Disagree
- Neutral
- Agree
- Strongly agree

For peer review only - http://bmjopen.bmj.com/site/about/guidelines.xhtml

10.1.c - What response have you had from this university?

- Rejected without interview/MMI
- Rejected after interview/MMI
- Waiting to hear if I will be interviewed
- Due to be interviewed
- Interviewed and waiting to hear if I have an offer
- Conditional offer (please type offer in box on the right)
- Unconditional offer
- Other

10.2.c - What response have you had from this university?

- Rejected without interview/MMI
- Rejected after interview/MMI
- Waiting to hear if I will be interviewed
- Due to be interviewed
- Interviewed and waiting to hear if I have an offer
- Conditional offer (please type offer in box on the right)
- Unconditional offer
- Other

10.3.c - What response have you had from this university?

- Rejected without interview/MMI
- Rejected after interview/MMI
- Waiting to hear if I will be interviewed
- Due to be interviewed
- Interviewed and waiting to hear if I have an offer
- Conditional offer (please type offer in box on the right)
- Unconditional offer
- Other

10.4.c - What response have you had from this university?

- Rejected without interview/MMI
- Rejected after interview/MMI
- Waiting to hear if I will be interviewed
- Due to be interviewed
- Interviewed and waiting to hear if I have an offer
- Conditional offer (please type offer in box on the right)
- Unconditional offer
- Other

10.5.c - What response have you had from this university?

- Rejected without interview/MMI
- Rejected after interview/MMI
- Waiting to hear if I will be interviewed
- Due to be interviewed
- Interviewed and waiting to hear if I have an offer
- Conditional offer (please type offer in box on the right)
- Unconditional offer
- Other

11 - Your Choice 1: has this university told you about any changes they are making to their selection processes due to coronavirus/COVID-19?

- No
- Yes, said changes will be happening but did not specify
- Yes, specified changes
- Other

For peer review only - http://bmjopen.bmj.com/site/about/guidelines.xhtml

12 - Your Choice 2: has this university told you about any changes they are making to their selection processes due to coronavirus/COVID-19?

No
Yes, said changes will be happening but did not specify
Yes, specified changes
Other

13 - Your Choice 3: has this university told you about any changes they are making to their selection processes due to coronavirus/COVID-19?

No
Yes, said changes will be happening but did not specify
Yes, specified changes
Other

14 - Your Choice 4: has this university told you about any changes they are making to their selection processes due to coronavirus/COVID-19?

No
Yes, said changes will be happening but did not specify
Yes, specified changes
Other

15 - Your Choice 5: has this university told you about any changes they are making to their selection processes due to coronavirus/COVID-19?

No
Yes, said changes will be happening but did not specify
Yes, specified changes
Other

21.1.a - Qualification type

A-Level
AS level
Scottish Higher
Scottish Advanced Higher
International Baccalaureate
Welsh Baccalaureate
Cambridge Pre-U
Irish Leaving Certificate
European Baccalaureate
Access to HE Diploma
BTEC National Diploma
Extended Project Qualification
Degree
Postgraduate qualification
Other

21.2.a - Qualification type

A-Level
AS level
Scottish Higher
Scottish Advanced Higher
International Baccalaureate
Welsh Baccalaureate
Cambridge Pre-U
Irish Leaving Certificate
European Baccalaureate
Access to HE Diploma

For peer review only - http://bmjopen.bmj.com/site/about/guidelines.xhtml

BTEC National Diploma
Extended Project Qualification
Degree
Postgraduate qualification
Other

21.3.a - Qualification type

A-Level
AS level
Scottish Higher
Scottish Advanced Higher
International Baccalaureate
Welsh Baccalaureate
Cambridge Pre-U
Irish Leaving Certificate
European Baccalaureate
Access to HE Diploma
BTEC National Diploma
Extended Project Qualification
Degree
Postgraduate qualification
Other

21.4.a - Qualification type

A-Level
AS level
Scottish Higher
Scottish Advanced Higher
International Baccalaureate
Welsh Baccalaureate
Cambridge Pre-U
Irish Leaving Certificate
European Baccalaureate
Access to HE Diploma
BTEC National Diploma
Extended Project Qualification
Degree
Postgraduate qualification
Other

21.5.a - Qualification type

A-Level
AS level
Scottish Higher
Scottish Advanced Higher
International Baccalaureate
Welsh Baccalaureate
Cambridge Pre-U
Irish Leaving Certificate
European Baccalaureate
Access to HE Diploma
BTEC National Diploma
Extended Project Qualification
Degree
Postgraduate qualification
Other

For peer review only - http://bmjopen.bmj.com/site/about/guidelines.xhtml

21.6.a - Qualification type

A-Level
AS level
Scottish Higher
Scottish Advanced Higher
International Baccalaureate
Welsh Baccalaureate
Cambridge Pre-U
Irish Leaving Certificate
European Baccalaureate
Access to HE Diploma
BTEC National Diploma
Extended Project Qualification
Degree
Postgraduate qualification
Other

21.7.a - Qualification type

A-Level
AS level
Scottish Higher
Scottish Advanced Higher
International Baccalaureate
Welsh Baccalaureate
Cambridge Pre-U
Irish Leaving Certificate
European Baccalaureate
Access to HE Diploma
BTEC National Diploma
Extended Project Qualification
Degree
Postgraduate qualification
Other

21.8.a - Qualification type

A-Level
AS level
Scottish Higher
Scottish Advanced Higher
International Baccalaureate
Welsh Baccalaureate
Cambridge Pre-U
Irish Leaving Certificate
European Baccalaureate
Access to HE Diploma
BTEC National Diploma
Extended Project Qualification
Degree
Postgraduate qualification
Other

21.9.a - Qualification type

A-Level
AS level
Scottish Higher
Scottish Advanced Higher
International Baccalaureate

For peer review only - http://bmjopen.bmj.com/site/about/guidelines.xhtml

Welsh Baccalaureate
Cambridge Pre-U
Irish Leaving Certificate
European Baccalaureate
Access to HE Diploma
BTEC National Diploma
Extended Project Qualification
Degree
Postgraduate qualification
Other

21.10.a - Qualification type
A-Level
AS level
Scottish Higher
Scottish Advanced Higher
International Baccalaureate
Welsh Baccalaureate
Cambridge Pre-U
Irish Leaving Certificate
European Baccalaureate
Access to HE Diploma
BTEC National Diploma
Extended Project Qualification
Degree
Postgraduate qualification
Other

29.1.a -
Used
Not used

29.1.b - If you have not used this, why not?
Not available
Not enough private/quiet space
Not enough time
Find it hard to focus
No access to computer/internet
Don't need to do this
Other reason(s)

29.2.a -
Used
Not used

29.2.b - If you have not used this, why not?
Not available
Not enough private/quiet space
Not enough time
Find it hard to focus
No access to computer/internet
Don't need to do this
Other reason(s)

29.3.a -

For peer review only - http://bmjopen.bmj.com/site/about/guidelines.xhtml

Used
Not used

29.3.b - If you have not used this, why not?
Not available
Not enough private/quiet space
Not enough time
Find it hard to focus
No access to computer/internet
Don't need to do this
Other reason(s)

29.4.a -
Used
Not used

29.4.b - If you have not used this, why not?
Not available
Not enough private/quiet space
Not enough time
Find it hard to focus
No access to computer/internet
Don't need to do this
Other reason(s)

29.5.a -
Used
Not used

29.5.b - If you have not used this, why not?
Not available
Not enough private/quiet space
Not enough time
Find it hard to focus
No access to computer/internet
Don't need to do this
Other reason(s)

29.6.a -
Used
Not used

29.6.b - If you have not used this, why not?
Not available
Not enough private/quiet space
Not enough time
Find it hard to focus
No access to computer/internet
Don't need to do this
Other reason(s)

29.7.a -
Used
Not used

For peer review only - http://bmjopen.bmj.com/site/about/guidelines.xhtml

29.7.b - If you have not used this, why not?

Not available
Not enough private/quiet space
Not enough time
Find it hard to focus
No access to computer/internet
Don't need to do this
Other reason(s)

29.8.a -

Used
Not used

29.8.b - If you have not used this, why not?

Not available
Not enough private/quiet space
Not enough time
Find it hard to focus
No access to computer/internet
Don't need to do this
Other reason(s)

34 - Occupation of Parent/Carer 1:

Modern Professional (e.g. teacher, nurse, social worker, artist, police officer (sergeant or above))
Clerical & intermediate (e.g. secretary, call centre agent, nursing auxiliary, nursery nurse)
Senior managers or administrators (e.g. finance manager, chief executive)
Technical & craft (e.g. motor mechanic, plumber, printer, tool maker, gardener, train driver, fitter)
Semi-routine manual & service (e.g. postal / farm worker, security guard, catering/sales assistant)
Routine manual & service (e.g. HGV driver, cleaner, porter, sewing machinist, bar staff, labourer)
Middle or junior managers (e.g. office / retail / bank / restaurant / warehouse manager, publican)
Traditional professional (e.g. accountant, solicitor, medical practitioner, scientist, civil servant)
Never worked
Do not know
Information withheld
Other

35 - Occupation of Parent/Carer 2:

Modern Professional (e.g. teacher, nurse, social worker, artist, police officer (sergeant or above))
Clerical & intermediate (e.g. secretary, call centre agent, nursing auxiliary, nursery nurse)
Senior managers or administrators (e.g. finance manager, chief executive)
Technical & craft (e.g. motor mechanic, plumber, printer, tool maker, gardener, train driver, fitter)
Semi-routine manual & service (e.g. postal / farm worker, security guard, catering/sales assistant)
Routine manual & service (e.g. HGV driver, cleaner, porter, sewing machinist, bar staff, labourer)
Middle or junior managers (e.g. office / retail / bank / restaurant / warehouse manager, publican)
Traditional professional (e.g. accountant, solicitor, medical practitioner, scientist, civil servant)
Never worked
Do not know
Information withheld
Not applicable
Other

36 - Does one or more of your parent(s)/carer(s) have a university degree?

Yes, one has a degree
Yes, both have degrees
No
Rather not say

For peer review only - http://bmjopen.bmj.com/site/about/guidelines.xhtml

37 - Is one or more of your parent(s)/carer(s) a medical doctor?

Yes, one is a medical doctor

Yes, both are medical doctors

No

Rather not say

41 - Which of the following best describes the gender you identify as?

Male

Female

Prefer not to answer

Other

For peer review only

For peer review only - http://bmjopen.bmj.com/site/about/guidelines.xhtml

Supplementary file 1: Missing data.

The analysis considered 120 measures in the restricted sample, divided into:

1. *Questionnaire items.* The questionnaire asked about attitudes to 87 different topics concerning medical school entrance. Of 153,076 data points, 10788 (7.2%) were missing. For the individual variables, the median percentage of missing data values was 0.48%, with 75 measures having fewer than 5% of missing values.

2. *Demographic and educational items*. For 12 demographic measures, 462 of 18744 measures were missing (2.5%), with a median of 1.0% per measure, and 11 measures having fewer than 5% missing values. Ethnic origin was not asked about in the present study. The ethnicity of 889 respondents who had reported it in a previous questionnaire were imported into the present dataset; 43.1% of ethnicity measures were therefore missing. IMD_Quintile was obtained from postcodes in England, Wales and Scotland, and was missing in 14.8% of cases.

There were four educational attainment items (grades in the highest-scoring 'top' three predicted A-level grades, UCAT score, BMAT score, and mean GCSE grade). Top three predicted A-level grades were present for all because the sample was based on that criterion. Of the remaining three measures, 1852 out of 4686 (39.5%) were missing: UCAT scores were missing in 13.6% of cases, and BMAT scores in 61.3% of cases, but in both cases missing values were mostly structurally missing, candidates mostly having taken only one aptitude test or the other. Mean GCSE grade was missing in 43.1% of cases, having been imported from a previous UKMACS questionnaire.

Participants self-reported their current or most recent school in the current questionnaire. This question was also present in the Wave 1 UKMACS questionnaire. For schools in England, publicly-available administrative data were available on school type (e.g. independent, voluntary aided) and for state-funded schools there were data on whether the admissions policy was selective or non-selective. These were combined to create a binary variable of School Type (non-selective state schools vs private/selective schools) for 1132 respondents (27.1% missing). A composite variable was created using present responses and the responses in the Wave 1 questionnaire, so data were available for 1158 respondents with values missing in 25.9% of respondents.

Missing values were imputed using the *mice* package.(18) Following the general advice of van Buuren (19) missing values were calculated using *pmm* (predictive mean matching), which as van Buuren says, is a good " all-round method with exceptional properties" (p.84). *pmm* is the default method in the *mice()* function for all scale types (binary, ordinal, numeric) and has the advantage that imputed values are always taken from the existing range of actual values in the data, with *pmm* being robust against mis-specification. The number for the pool of candidate donors, *d*, was set at 5, the default in *mice()*, and the number of imputations, *m*, was set at 25.

Regression analyses on the 25 *mira* datasets were carried out using the *lm()* function within the *with()* function, and separate sets of results in the *mipo* dataset were combined with the *pool()* function. Regression analyses entered all socio-demographic and educational predictor variables into the analysis simultaneously, and results are only reported which were significant with p<.01 after taking all other variables into account, so the analysis is relatively conservative. The nine socio-demographic and educational variables used were: ethnicity, gender, school type, parental higher education, IMD quintile, mean GCSE points, mean top three predicted A-levels, UCAT score, number of medical school offers.

For peer review only

Supplementary file 2: Results for the 665 post-Year 13 respondents excluded from the restricted sample.

This sample includes mature and graduate applicants from the whole of the UK.

Applicant views on admissions

Perceptions of the fairness of methods to select or reject offer holders

As with the restricted sample, no single method was perceived as fair enough to use on its own but many were considered fair enough to use in conjunction with others.

Since this group includes those currently at university and graduate applicants, we have included responses to two additional items: *For those in their final year at university, marks earlier in their course*, which was considered very fair by 35% and quite fair by 45%, and *GAMSAT score (for Graduate Entry students)* which was considered very fair by 17.6% and quite fair by 46.8%.

Compared to those in Year 13, *Predicted grades declared on UCAS form* were considered much less fair and *Personal background (e.g. giving an advantage to students from under-represented groups)* was considered by a majority (52.1%) to be very fair/quite fair.

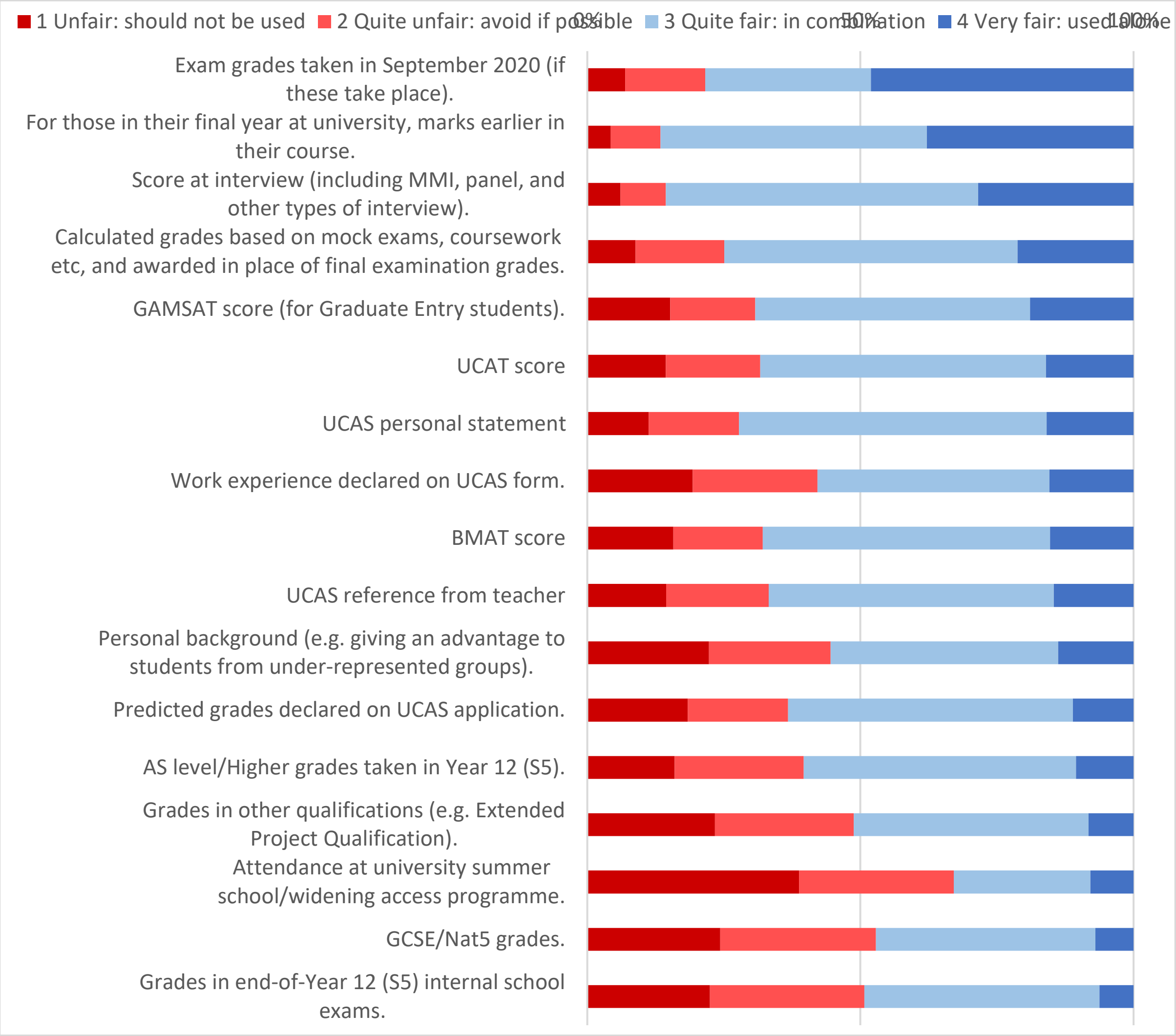

Figure S1: Perceptions of the fairness of methods medical schools could use to decide whether or not to accept applicants who currently hold an offer now that exams have been cancelled. Post-Year13 respondents only.

Acceptability of options for dealing with a situation in which more students meet their offers than there are medical school places

The only option that was rated as slightly or completely acceptable by the majority of respondents (64.6%) was asking for volunteers to defer. Accepting all applicants who meet the conditional offer was the second most acceptable and more acceptable than it was unacceptable.

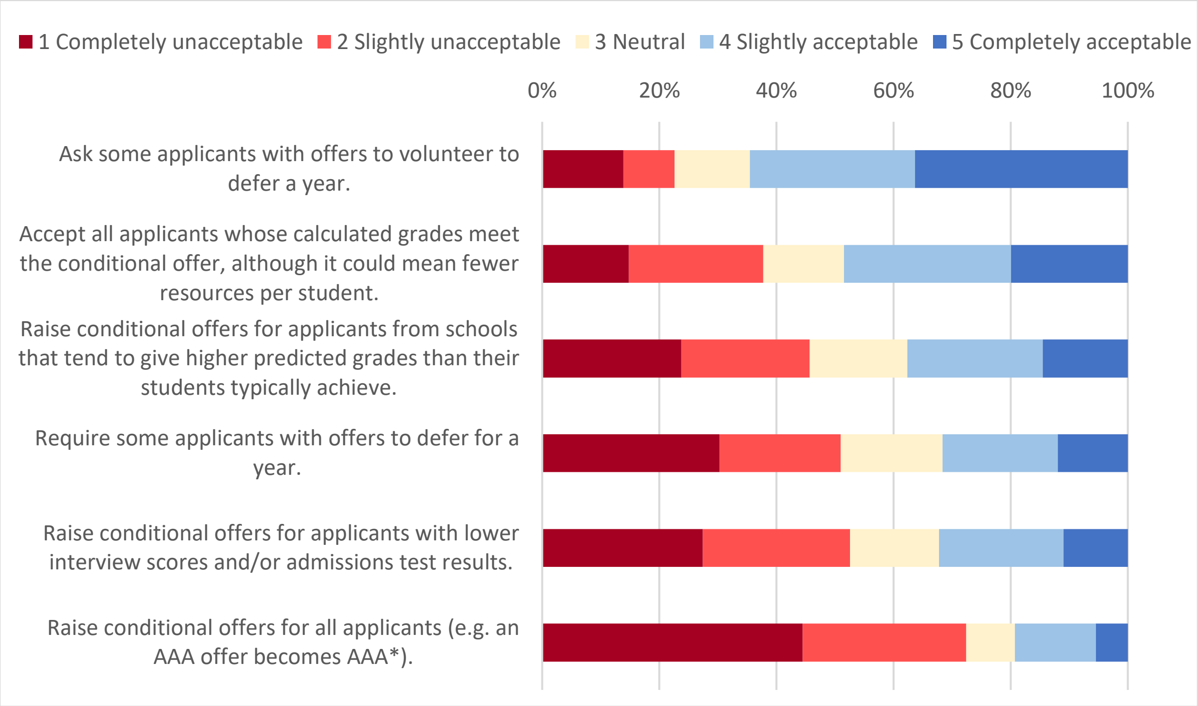

Figure S2: Acceptability of actions medical schools could take if they have more applicants meeting offers than they have places. Post-Year 13 respondents only.

Perceptions of potential impact on admissions for 2021

Respondents were even more divided than in the restricted sample, with about half of respondents (53.8%) agreeing/strongly agreeing that *Applicants rejected this year should be given special consideration when re-applying next year* but 51.5% agreeing/strongly agreeing that *Applicants rejected this year should apply next year in the usual way and be considered with all other applicants*.

For peer review only

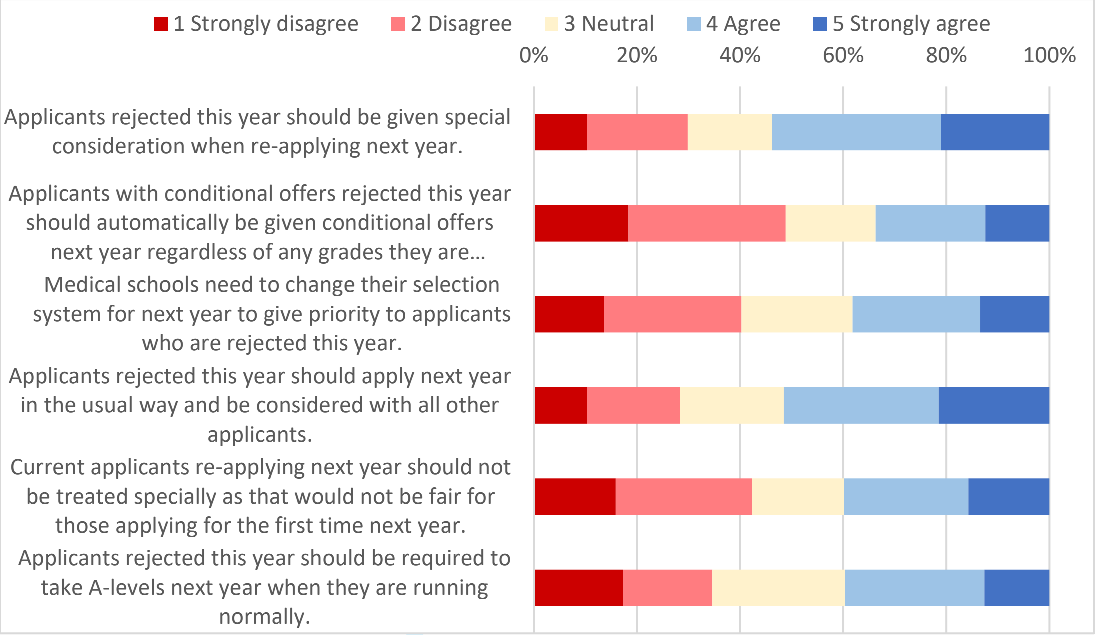

Figure S3: Views on how current applicants should be considered by medical schools if they reapply next year. Post-Year 13 respondents only.

Starting academic year 2020/2021

A majority of respondents (n=375, 56.4%) believed that if necessary, medical schools should *Defer the start of the academic year only when face-to-face teaching is possible* with 285 respondents (42.6.9%) believing that medical schools should *Start the academic year on time using distance learning for as long as is necessary*.

Education and university preparation

Perceptions of process to award calculated grades in lieu of examination grades

Post-Year 13 respondents were generally more negative about calculated grades than respondents in the restricted sample and unsurprisingly there were more "neutral" responses in general and specifically to questions about their own teachers and grades.

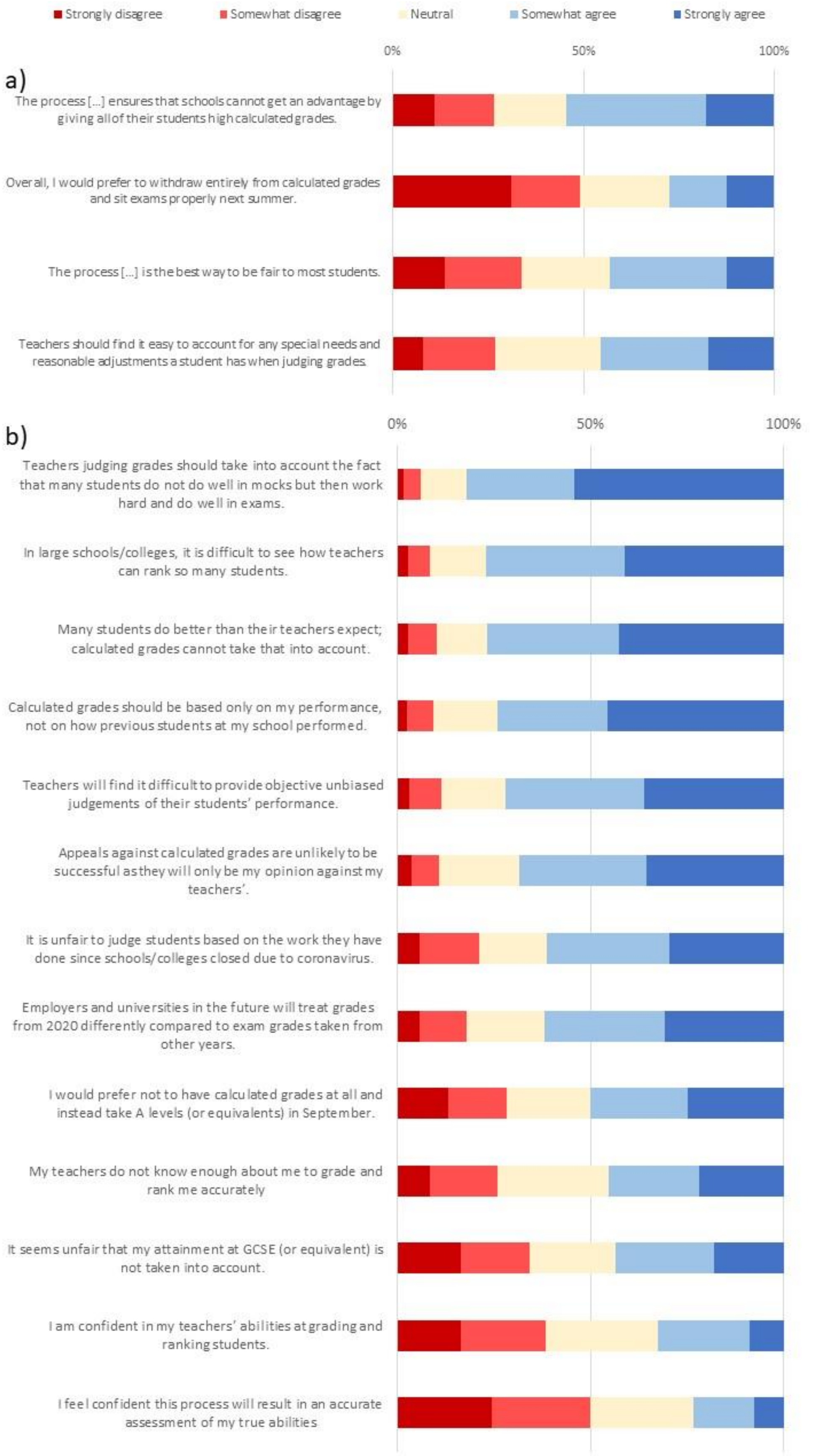

Figure S4: Aspects of calculated grades that respondents were generally more a) positive and b) negative about. Post-Year 13 respondents only.

A number of applicants were re-sitting their examinations and/or were not studying at a school or college but nonetheless were due to take examinations this summer (so-called 'private candidates'). Several expressed concerns about whether the institution they were due to take their exams with would give them a calculated grade, and if so, what information that grade would be based on:

> *"As a resit student, my previous college which I was registered to retake my exams with this year have decided that they cannot give me calculated grades. I am unsure how to maintain my offers despite not getting grades."*

> *"I am extremely concerned about how offers made to private candidates who cannot get predicted grades from a school will be treated. Though I had been studying in my lunchtimes/evenings/weekends for over a year, I quit my job 4 days after getting an offer from [redacted] in order to have time to put the work in to get the grades I need. I achieved straight A*s at GCSEs and A-level, so I know how much work it takes to get top grades. I am terrified universities I have offers from will wash their hands of me as I don't have any grades, or forced to defer for a year because universities won't wait for September exam results. Ofqual and exams boards keep saying no student will be disadvantaged, but it appears private candidates like myself may fall through the cracks."*

> *"I worry that I, as a resitting privately tutored student, will be disadvantaged by the "calculated grades system", as I haven't been in school this year and thus have no exams or schoolwork that could be provided as evidence to support a predicted grade."*

> *"I'm worried about how they'll handle resits who have been independently studying as I need to go from a B to an A but am worrying that my old school(exam centre) won't provide me with a grade even though I'm certain that I'd be able to get an A had I taken the exam. I also can't afford to take another gap year so I'm hoping unis will take situations like these independently as it would be very unlikely that I'd receive the same grade as last year had I resat."*

> *"For exam centre who cannot provide grades for resit external students please consider our previous attainment especially if for an applicant like myself has achieved AAB grades from last year and narrowly missed the A grade in Maths by 8 marks. It would be unfair for me to have to take another gap year if I don't receive a grade this summer."*

Education since the shutdown

Although participants were post Year-13 many were still in education, whether at school, college or university. The mean number of resources used by participants was 2.9 (SD=1.86).

Like Year 13 respondents, post-Year 13 respondents were using mostly online and paper resources, but 42.8% of post-Year 13 respondents reported having online teaching in real time and nearly half (49.6%) were having online summative tests and; 30.6% reported that their school/college/university would be assessing them formally on work since the closure of schools (although 42.1% reported that this was not applicable to them). See Figure S5.

For peer review only

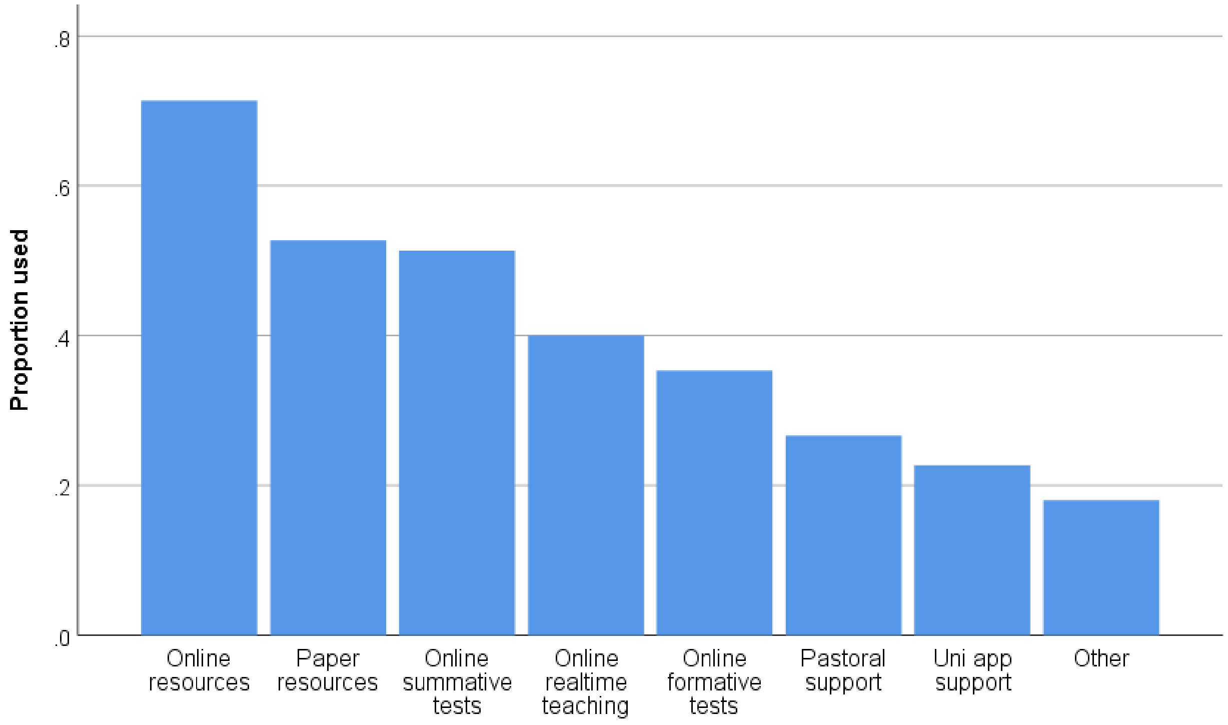

Figure S5: Proportion of post-Year 13 respondents using educational resources since the closure of schools.

Preparation for medical school/university

Post-Year 13 respondents were doing similar sorts of preparation, although they were talking to their friends less. Of the 100 (15.0% of the sample) who said they were not doing any preparation, reasons were different from those in the restricted sample. They were five times more likely to say they did not have time (31.0% vs 6.3%), about half as likely to say they were too worried and not able to focus (26.0% vs 42.5%), and over half as likely to say they did not have resources (15.0% vs 29.5%). A similar percentage selected caring for others as a reason (13.0%), not going to university this year (19.0%), being unwell (6.0%).Respondents could select multiple reasons.

For peer review only

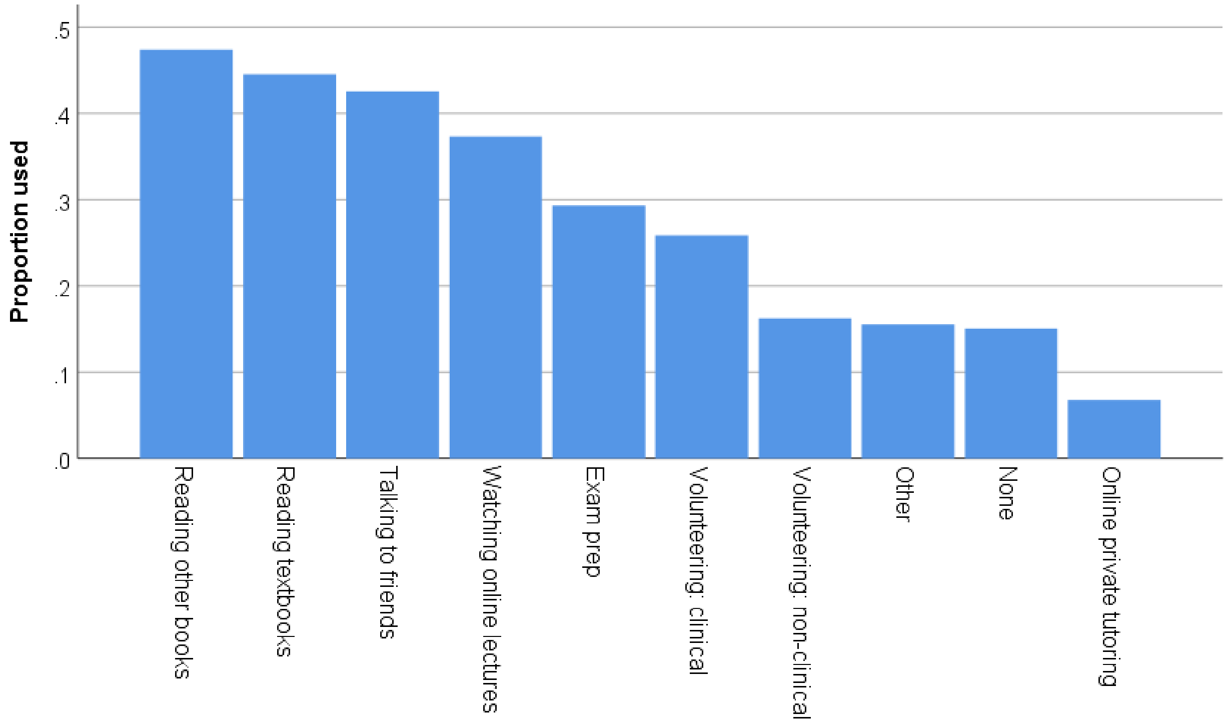

Figure S6: Proportion of respondents undertaking various activities to prepare for medical school or university. Post-Year 13 respondents only.

Time spent during the lockdown

Post-Year 13 respondents were spending broadly similar amounts of time on various activities as those in the restricted sample although they were spending more time volunteering and reading about coronavirus, and less time studying and gaming with friends.

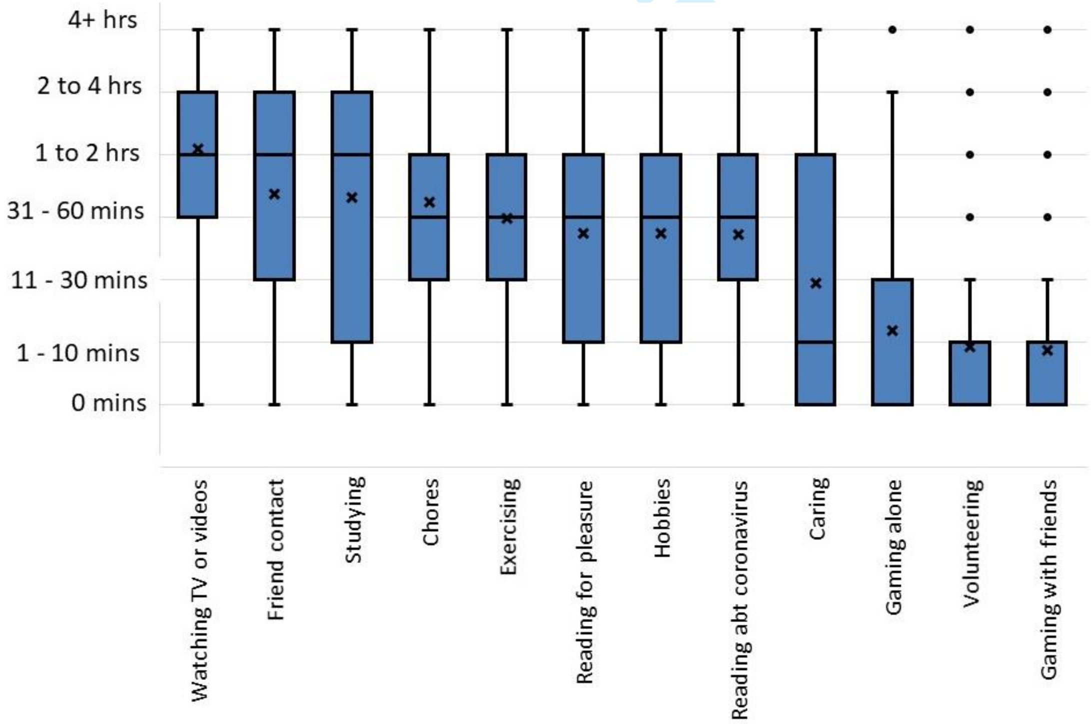

Figure S7: Amount of time respondents reported spending on various activities during the lockdown. Non-Year 13 respondents only.

For peer review only

Supplementary file 3: Results for 125 Scottish S6 respondents excluded from the main analyses

Applicant views on admissions

Perceptions of the fairness of methods to select or reject offer holders

Scottish applicants were similarly uncertain that any measure was fair enough to use alone, however unlike applicants from other UK countries they were more positive about the fairness of using AS level/Higher grades taken in Year 12. This is probably because AS levels are no longer in widespread use whereas Highers are. Scottish applicants were also relatively more positive about the use of calculated grades (83.2% quite or very fair).

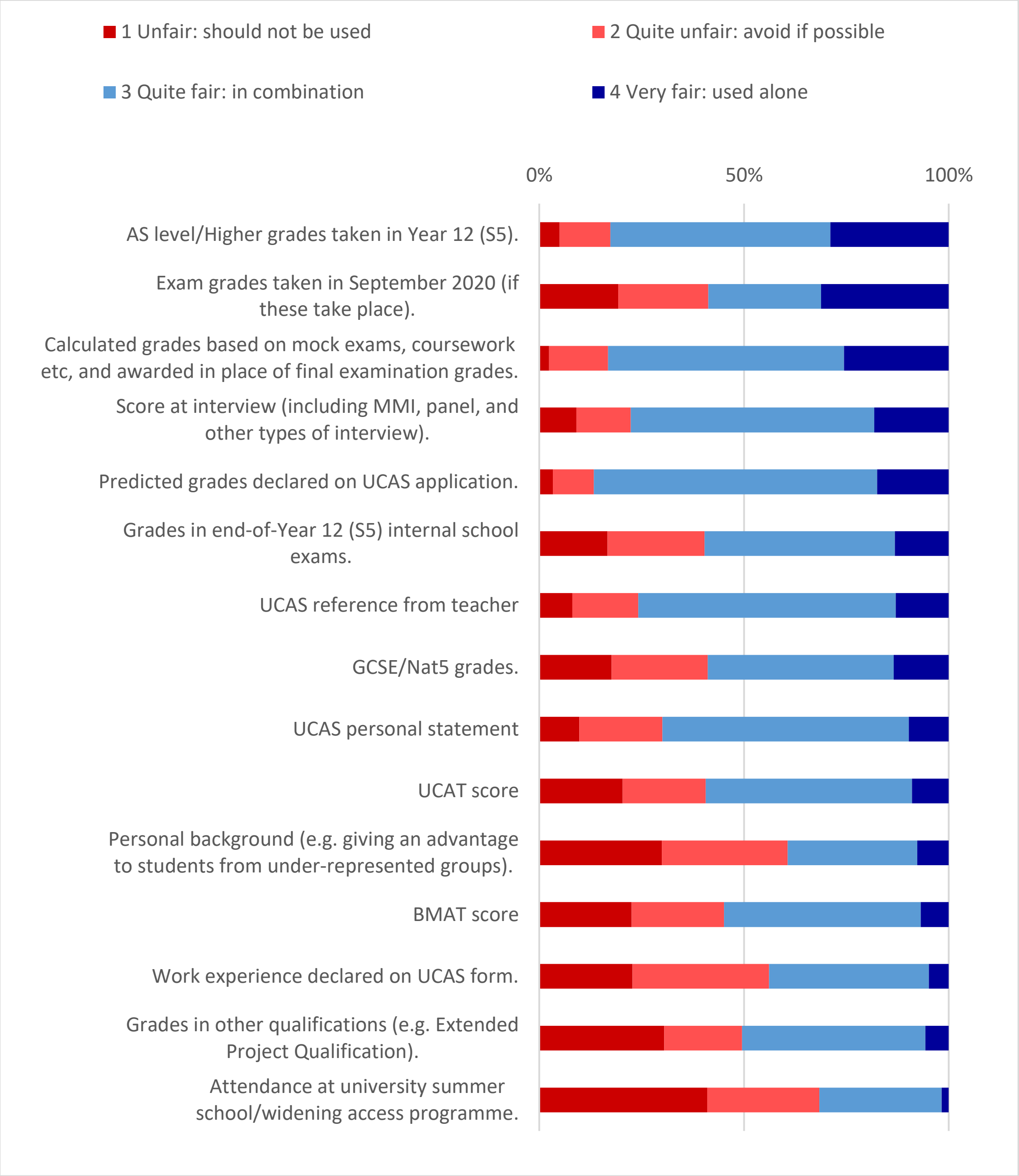

Figure S8: Perceptions of the fairness of methods medical schools could use to decide whether or not to accept applicants who currently hold an offer now that exams have been cancelled. Post-Scottish S6 respondents only.

Acceptability of options for dealing with a situation in which more students meet their offers than there are medical school places

As with other school students, the two acceptable options were accepting all applicants and asking for volunteers to defer.

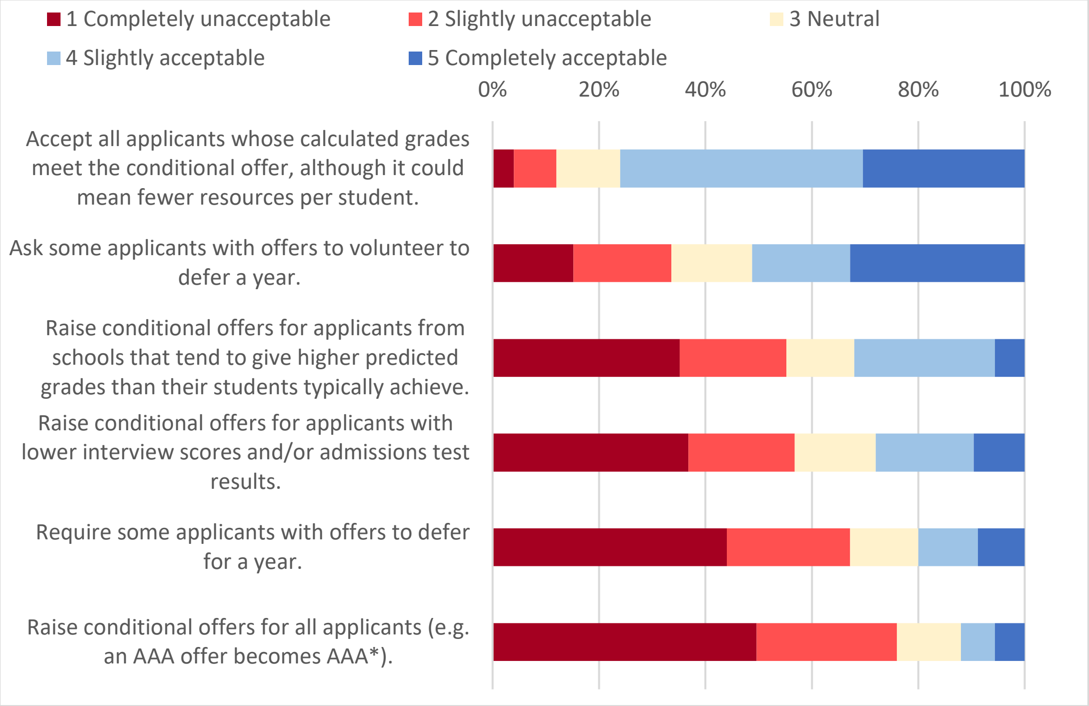

Figure S9: Acceptability of actions medical schools could take if they have more applicants meeting offers than they have places. Scottish S6 respondents only.

Perceptions of potential impact on admissions for 2021

Scottish S6 respondents were even more divided than in other UK countries: half the sample (52.8%) agreed that applicants rejected this year should be given special consideration and half (53.2%) agreeing that they should reapply next year in the usual way and be considered with all other applicants.

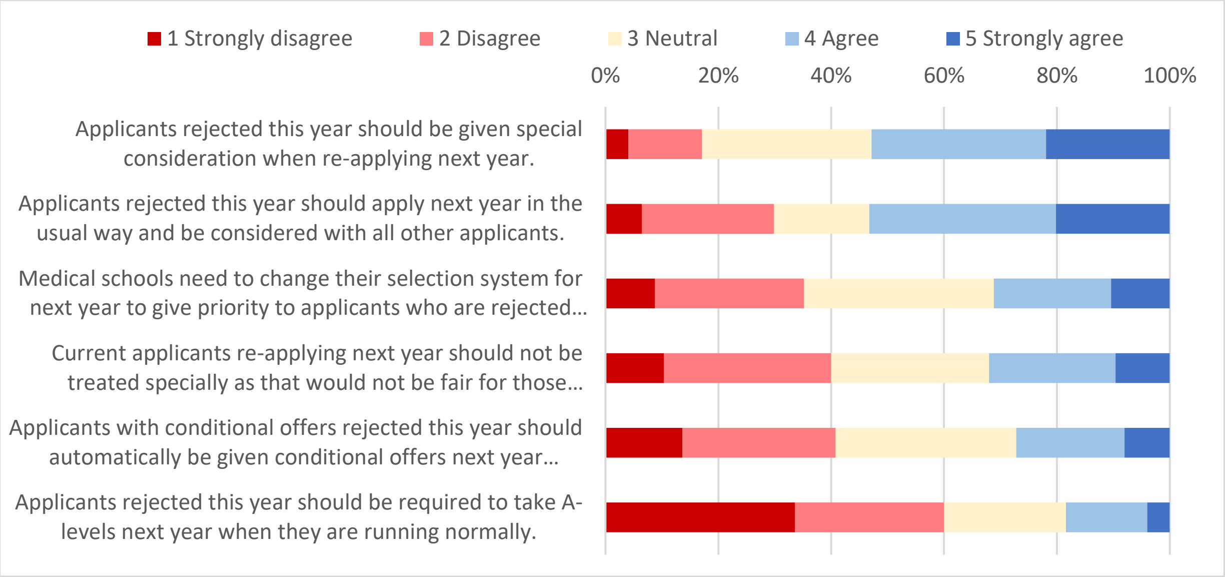

Figure S10: Views on how current applicants should be considered by medical schools if they reapply next year. Scottish S6 respondents only.

Starting academic year 2020/2021

A majority of respondents (n=70; 56.0%) believed that if necessary, medical schools should *Defer the start of the academic year only when face-to-face teaching is possible* with 55 respondents (44.0%) believing that medical schools should *Start the academic year on time using distance learning for as long as is necessary*.

Education and university preparation

Perceptions of process to award calculated grades in lieu of examination grades

Scottish respondents were generally slightly more positive about calculated grades than their equivalents in other UK countries. They were more positive about their teacher's ability to rank and grade students accurately (70.4% agree/strongly agree) and that their teachers knew them well enough to rank and grade them personally (59.2% agree/strongly agree). On the negative side they had similar levels of concern about other aspects of calculated grades as did school students in other UK countries.

For peer review only

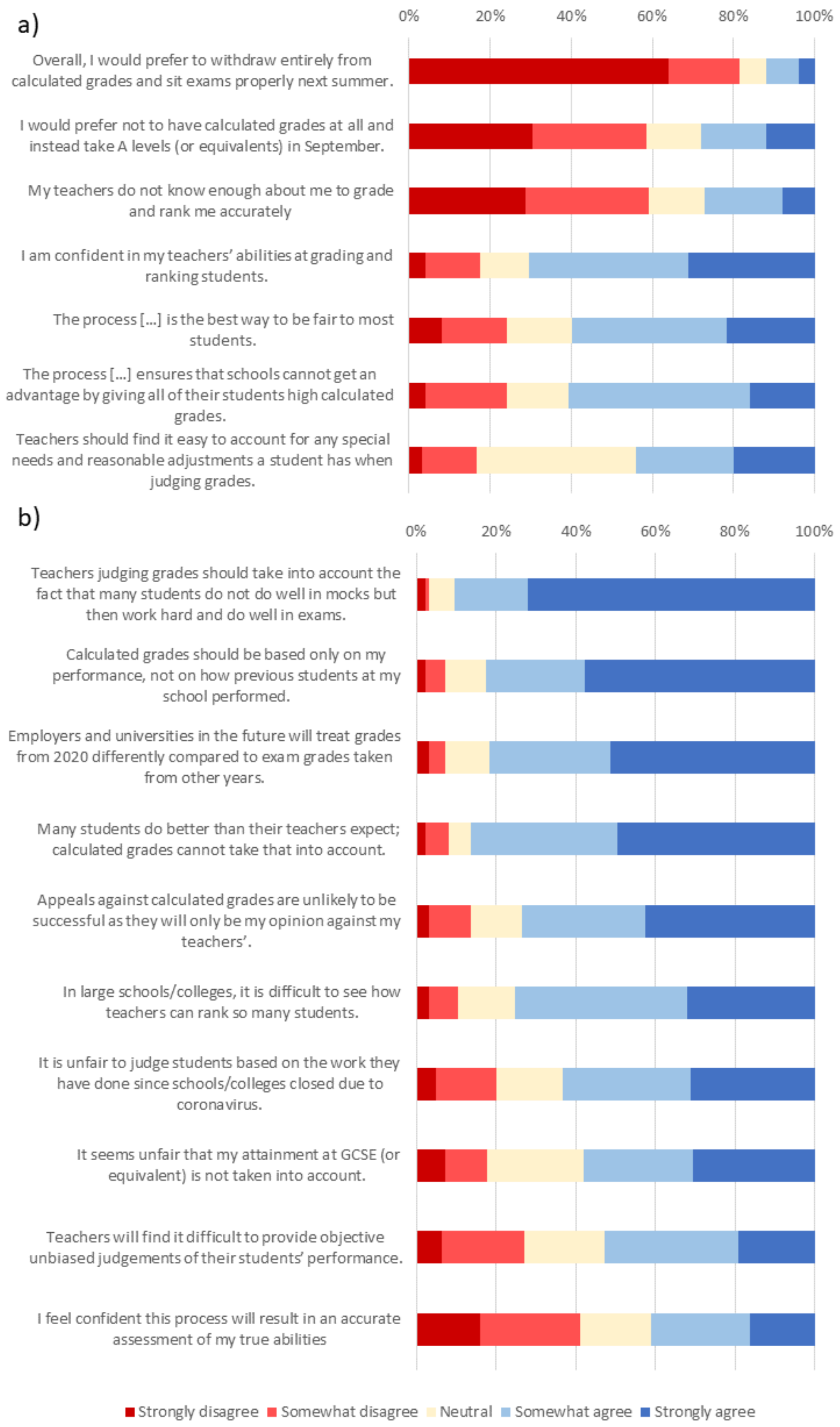

Figure S11: Aspects of calculated grades that respondents were generally more a) positive and b) negative about. Scottish S6 respondents only.

Education since the shutdown

Scottish S6 respondents used on average 2.2 (SD=1.6) educational resources provided by their school, which is fewer than those in the restricted sample. Figure S12 shows Scottish S6 used fewer of all resources compared to the restricted sample, with the exception of summative tests which they were more than twice as likely to use. Scottish S6 students were also more than twice as likely to say their school was assessing them on work since schools closed (n=37; 29.6) with a similar number (n=35; 28.0%) being unsure, and a larger proportion (n=49; 39.2%) saying they were not being assessed.

	Scotland S6	Restricted sample
Online resources	67 (59.8)	781 (71.7)
Paper resources	37 (33.6)	690 (63.9)
Online formative tests	22 (20.0)	447 (41.5)
Pastoral support	32 (29.1)	359 (33.4)
University application support	25 (23.4)	326 (30.4)
Online teaching in real time	31 (27.7)	314 (29.2)
Online summative tests	38 (34.2)	165 (15.4)
Other	<5 (<10)	37 (10.1)

Figure S12: Educational resources provided by schools used in the Scottish and Restricted samples.

Preparation for medical school/university

Scottish applicants were doing similar sorts of preparation as those in the restricted sample; although they were half as likely to be doing examination preparation (n=14; 11.2% vs n=335; 21.4%). Only 19 (15.2%) said they were not doing any preparation which meant numbers were too small to look at reasons for not doing preparation.

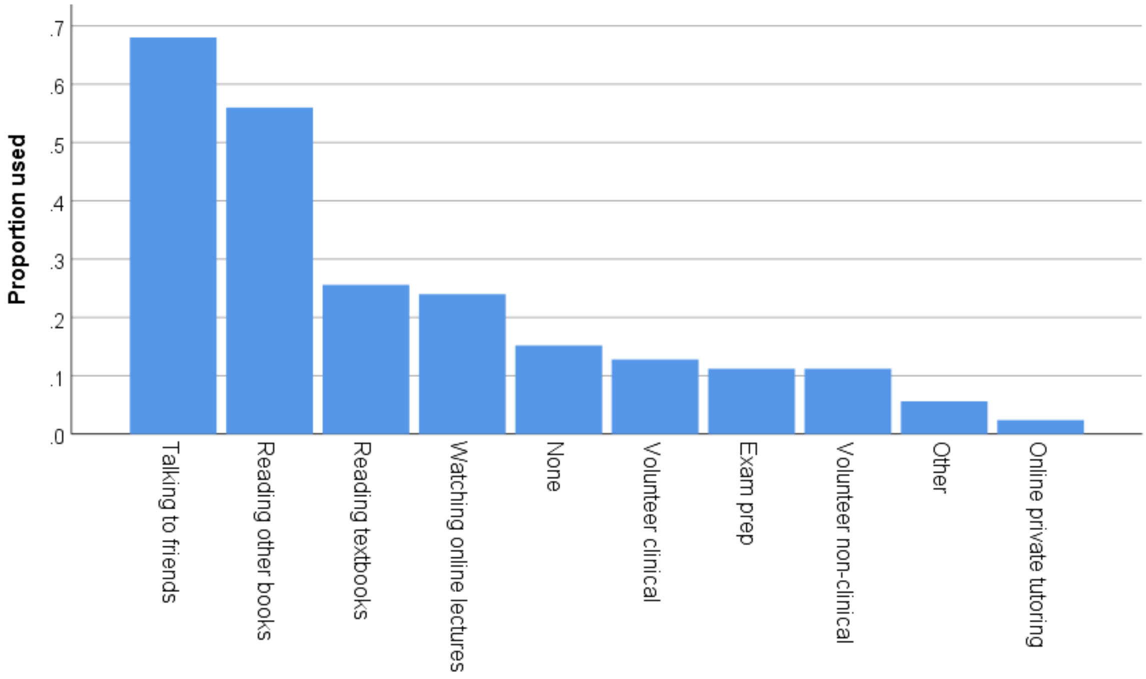

Figure S13: Proportion of respondents undertaking various activities to prepare for medical school or university. Scottish S6 respondents only.

Time spent during the lockdown

The Scottish S6 sample reported similar amounts of time spent on activities as the restricted sample.

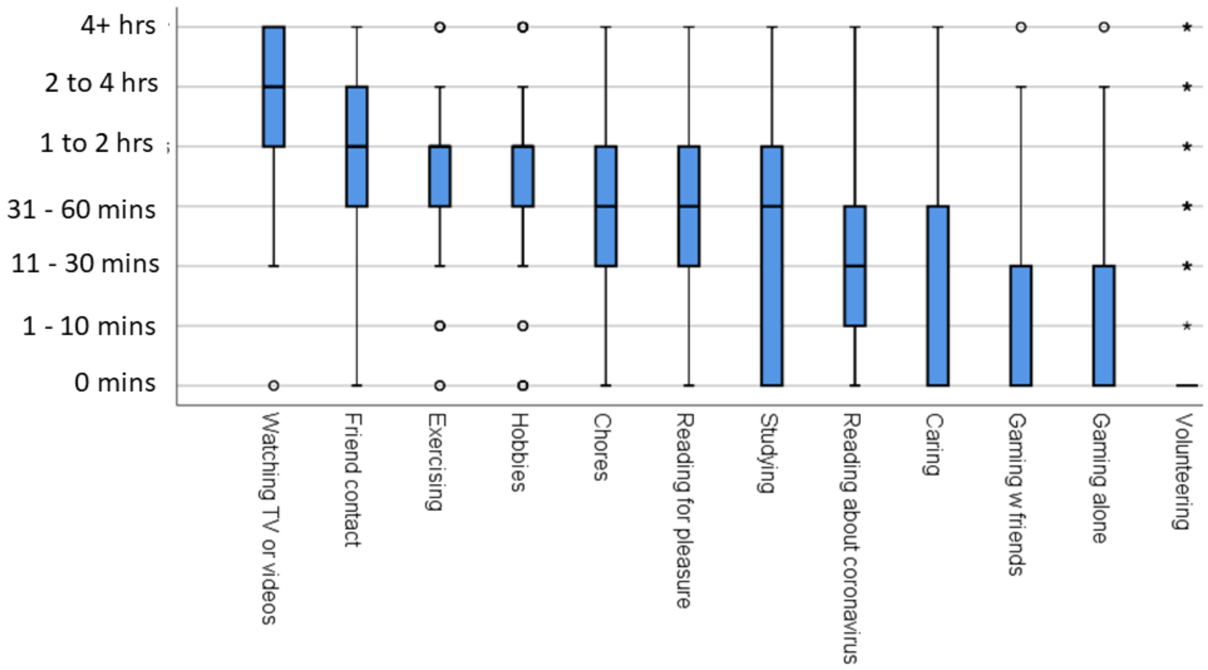

Figure S:14 Amount of time respondents reported spending on various activities during the lockdown. Scottish S6 respondents only.

For peer review only

STROBE Statement—checklist of items that should be included in reports of observational studies

	Item No	Recommendation	Page No.
**Title and abstract**	1	(*a*) Indicate the study's design with a commonly used term in the title or the abstract	2
		(*b*) Provide in the abstract an informative and balanced summary of what was done and what was found	2
**Introduction**			
Background/rationale	2	Explain the scientific background and rationale for the investigation being reported	3-5
Objectives	3	State specific objectives, including any prespecified hypotheses	5
**Methods**			
Study design	4	Present key elements of study design early in the paper	6
Setting	5	Describe the setting, locations, and relevant dates, including periods of recruitment, exposure, follow-up, and data collection	6,7
Participants	6	(*a*) *Cross-sectional study*—Give the eligibility criteria, and the sources and methods of selection of participants	6,7
Variables	7	Clearly define all outcomes, exposures, predictors, potential confounders, and effect modifiers. Give diagnostic criteria, if applicable	8
Data sources/ measurement	8*	For each variable of interest, give sources of data and details of methods of assessment (measurement). Describe comparability of assessment methods if there is more than one group	8
Bias	9	Describe any efforts to address potential sources of bias	8
Study size	10	Explain how the study size was arrived at	7
Quantitative variables	11	Explain how quantitative variables were handled in the analyses. If applicable, describe which groupings were chosen and why	7,8
Statistical methods	12	(*a*) Describe all statistical methods, including those used to control for confounding	8
		(*b*) Describe any methods used to examine subgroups and interactions	8
		(*c*) Explain how missing data were addressed	8
		(*d*) *Cross-sectional study*—If applicable, describe analytical methods taking account of sampling strategy	NA
		(*e*) Describe any sensitivity analyses	NA

Continued on next page

For peer review only - http://bmjopen.bmj.com/site/about/guidelines.xhtml

Results			Page No.
Participants	13*	(a) Report numbers of individuals at each stage of study—eg numbers potentially eligible, examined for eligibility, confirmed eligible, included in the study, completing follow-up, and analysed	7
		(b) Give reasons for non-participation at each stage	NA
		(c) Consider use of a flow diagram	NA
Descriptive data	14*	(a) Give characteristics of study participants (eg demographic, clinical, social) and information on exposures and potential confounders	9
		(b) Indicate number of participants with missing data for each variable of interest	9
		(c) *Cohort study*—Summarise follow-up time (eg, average and total amount)	NA
Outcome data	15*	*Cross-sectional study*—Report numbers of outcome events or summary measures	NA
Main results	16	(*a*) Give unadjusted estimates and, if applicable, confounder-adjusted estimates and their precision (eg, 95% confidence interval). Make clear which confounders were adjusted for and why they were included	10-24
		(*b*) Report category boundaries when continuous variables were categorized	9,10
		(*c*) If relevant, consider translating estimates of relative risk into absolute risk for a meaningful time period	NA
Other analyses	17	Report other analyses done—eg analyses of subgroups and interactions, and sensitivity analyses	7
**Discussion**			
Key results	18	Summarise key results with reference to study objectives	24,25
Limitations	19	Discuss limitations of the study, taking into account sources of potential bias or imprecision. Discuss both direction and magnitude of any potential bias	25,26
Interpretation	20	Give a cautious overall interpretation of results considering objectives, limitations, multiplicity of analyses, results from similar studies, and other relevant evidence	26,27
Generalisability	21	Discuss the generalisability (external validity) of the study results	26
**Other information**			
Funding	22	Give the source of funding and the role of the funders for the present study and, if applicable, for the original study on which the present article is based	7

Note: An Explanation and Elaboration article discusses each checklist item and gives methodological background and published examples of transparent reporting. The STROBE checklist is best used in conjunction with this article (freely available on the Web sites of PLoS Medicine at http://www.plosmedicine.org/, Annals of Internal Medicine at http://www.annals.org/, and Epidemiology at http://www.epidem.com/). Information on the STROBE Initiative is available at www.strobe-statement.org.

For peer review only
